# Psoraleae Fructus Ethanol Extract Induced Hepatotoxicity via Impaired Lipid Metabolism Caused by Disruption of Fatty Acid *β*-Oxidation

**DOI:** 10.1155/2023/4202861

**Published:** 2023-01-07

**Authors:** Zhaojuan Guo, Yuanyuan Shi, Bingqian Jiang, Xiyi Peng, Lin Zhang, Can Tu, Ting Wang

**Affiliations:** ^1^Beijing Research Institute of Chinese Medicine, Beijing University of Chinese Medicine, Beijing, China; ^2^NMPA Key Laboratory for Research and Evaluation of Traditional Chinese Medicine, Beijing University of Chinese Medicine, Beijing, China; ^3^Beijing International Science and Technology Cooperation Base for TCM Hepatotoxicity and New Drug Research and Development, Beijing University of Chinese Medicine, Beijing, China; ^4^School of Life Sciences, Beijing University of Chinese Medicine, Beijing, China

## Abstract

Herb-induced liver injury (HILI) is gradually increasing, and Psoraleae Fructus (PF) has been reported to induce hepatotoxicity. However, its underlying toxicity mechanism has been only poorly revealed. In this paper, we attempted to explore the liver injury and mechanism caused by Psoraleae Fructus ethanol extract (PFE). First, we administered PFE to mice for 4 weeks and evaluated their serum liver function indices. H&E staining was performed to observe the pathological changes of the livers. Oil red O staining was used to visualize hepatic lipids. Serum-untargeted metabolomics and liver proteomics were used to explore the mechanism of PF hepatotoxicity, and transmission electron microscopy was determined to assess mitochondria and western blot to determine potential target proteins expression. The results showed that PFE caused abnormal liver biochemical indicators and liver tissue injury in mice, and there was substantial fat accumulation in liver tissue in this group. Furthermore, metabolomic analysis showed that PFE changed bile acid synthesis, lipid metabolism, etc., and eight metabolites, including linoleic acid, which could be used as potential biomarkers of PFE hepatotoxicity. Proteomic analysis revealed that differential proteins were clustered in the mitochondrial transmembrane transport, the long-chain fatty acid metabolic process and purine ribonucleotide metabolic process. Multiomics analysis showed that eight pathways were enriched in both metabolomics and proteomics, such as bile secretion, unsaturated fatty acid biosynthesis, and linoleic acid metabolism. The downregulation of SLC27A5, CPT1A, NDUFB5, and COX6A1 and upregulation of cytochrome C and ABCC3 expressions also confirmed the impaired fatty acid oxidative catabolism. Altogether, this study revealed that PFE induced hepatotoxicity by damaging mitochondria, reducing fatty acid *β*-oxidation levels, and inhibiting fatty acids ingested by bile acids.

## 1. Introduction

In recent years, herbal and dietary supplements (HDS) are commonly used around the world, and there has been increasing recognition worldwide of the efficacy of herbs and their extracts in treating and preventing diseases [1–4]. However, herb medicines also have negative side effects like pharmaceutical medications. In recent years, it has been proven that several herb medicines and their products may induce liver injury. HDS is increasingly used in the US, and herb-induced liver injury (HILI) has proportionally increased. In a prospective study of drug-induced liver injury (DILI) from the NIH-funded Drug-Induced Liver Injury Network, HDS accounted for 16% of overall cases [5]. HILI has developed into a significant public health issue and should not be disregarded.

Psoraleae Fructus (PF) refers to the ripe dried seeds of *Psoralea corylifolia L.* (Leguminosae). It treats kidney- and spleen-related yang deficiencies, vitiligo, bone fracture, osteoporosis, and other conditions [6]. However, according to reports, there was a risk of liver damage after PF therapy. [7–10]. The objective nature of hepatotoxicity caused by PF has been confirmed by several studies [11–13]. Our previous research showed that, in the clinical literature, 64% of patients with liver injuries induced by PF experienced liver injury within two months of taking medicine, and most of the liver injury types were hepatocellular injuries. The risk factors for liver injury included the process, extraction technology, dosage and treatment period, and drug combination [14]. In our previous toxicity studies, PF ethanol extract (PFE) showed more significant hepatotoxicity in mice than PF water extract (PFW). Notably, steatosis and hepatocyte hypertrophy were observed in the central hepatic lobule zone of PEE-treated mice [15], which is consistent with the type of liver injury in the above clinical study. Overall, PF is a traditional Chinese herb with potential hepatotoxicity, whose underlying mechanisms remain obscure.

A frequent pathophysiology for DILI is mitochondrial failure, the production of reactive metabolites, and oxidative stress [16]. Another study discovered that bavachin triggered HepG2 cells to undergo apoptosis, with reactive oxygen species (ROS) serving as a crucial signal that caused endoplasmic reticulum stress and mitochondrial malfunction [17, 18]. Other studies have shown that bakuchiol induced hepatotoxicity by causing oxidative stress, mitochondrial damage, abnormal CYP450 enzyme activity, and the disturbance of bile acid transporter expression [19–22]. However, TCM is a complex of multiple chemical components. Different chemical components have different targets, levels, and pathways of action, and the complexity of the components in the herb determines the diversity of mechanisms that cause hepatotoxicity. Therefore, it is difficult for a single component to represent the whole TCM to elucidate its mechanism.

Recently, the emergence of multiomics studies based on systems biology provided a powerful tool for the study of Chinese medicine (CM) toxicology. The advantage of proteomics for studying CM toxicity is that they can rapidly and comprehensively identify molecular mechanisms by analyzing changes in protein expression in poisoned cells, tissues, and organs and identifying the specific expression of differential proteins and markers of toxicity or detoxification. Metabolomics directly reflects abnormalities in cell and tissue functions, and their changes over time are due to metabolic damage in the organism. Its holistic and global advantages coincide with the CM characteristics, with multiple pathways, components, and targets [23]. Proteomics and metabolomics now are widely used in hepatotoxicity studies of CM, such as Radix Rehmanniae [24], Scutellariae [25], and Polygoni Multiflori Radix [26, 27]. Therefore, this research verified PF-induced hepatotoxicity to examine the mechanism of PF-induced hepatotoxicity using metabolomics and proteomics techniques.

## 2. Materials and Methods

### 2.1. Drugs

Tong Ren Tang Chinese Pharmaceutical Co. Ltd. (Beijing, China) provided the PF samples. PF was identified as *Psoralea corylifolia L.*

### 2.2. Preparation of PFE

General Rule 0213 of the National Pharmacopoeia committee (2020 edition) was referenced to obtain salt PF. The extracts were combined, filtered, and concentrated. The produced samples were kept at 4°C for subsequent analysis, and the yield ratio was 65.40% (w/w, PFE).

### 2.3. Animals

Twenty male Kun Ming mice (23 ± 2 *g*) were purchased from Beijing Vital River Laboratory Animal Technology Co., Ltd. (Permission No. SCXK (Beijing) 2016-0011) and kept in an environment-controlled breeding room for five days with free food and water access at a temperature of 23-26°C. The mice were then grouped randomly into control and PFE groups (*n* = 10 each). CMC-Na was used to dissolve the PFE. Mice in the PFE group were given PFE extract (5.14 g/kg, approximately two times the clinical dose), and control group mice were intragastrically administered with CMC-Na. After four weeks of gavage administration, mice were sacrificed, and blood, liver, and brain were retained. The Beijing University of Chinese Medicine's Ethical Committee on Animal Research and the Guidelines for the Care and Use of Laboratory Animals authorized this experiment (No. BUCM-4-2019120302-4074).

### 2.4. Biochemical Assays and Histological Determination

Blood and liver tissues were collected 24 h following the last administration. Blood was centrifuged to obtain serum for detecting liver function indices such as alanine aminotransferase (ALT), aspartate aminotransferase (AST), and metabonomic analysis. The livers of each group of mice were quickly removed, and the left lateral lobe was divided into three parts for hematoxylin-eosin (H&E) staining, oil red O staining, and transmission electron microscopy, separately. Proteomics and western blot assays were performed on the liver's left medial lobe. The remaining liver specimens were instantly frozen and kept at -80°C.

### 2.5. Oil Red O Staining

The fatty content of the liver was determined using the oil red O staining kit (Solarbio Life Science, Beijing, China). In brief, liver tissues were fixed on a frozen section machine, the trimming thickness was adjusted to 15 *μ*m, and the sections were placed in PBS for washing three times for 5 min/time. After washing, sections were rinsed three times with distilled water for 5 minutes each time after being stained dropwise for 10 minutes at room temperature with an adequate amount of oil red O staining solution. The slices were then stained with hematoxylin for 3 minutes more before being rinsed three times with distilled water for 5 minutes each. Glycerin jelly was used as an aqueous mounting medium for mounting livers, and light microscopy was used to evaluate it.

### 2.6. Preparation of Serum Metabolism Samples

In brief, serum samples were thawed at 4°C. A total of 200 *μ*L of serum was added to 600 *μ*L of extraction mixture (methanol/acetonitrile = 1/1, v/v), homogenized by rapid vortexing for 30 s, incubated at 4°C for 30 min, and then centrifuged at 4°C for 20 min at 12000 rpm. After vortex shaking, the serums were centrifuged at 4°C f at 12000 rpm for 10 min. Finally, 10 *μ*L of each sample's supernatant was aspirated for quality control (QC) samples, and 200 *μ*L of each sample's supernatant was transferred to glass vials for nontargeted metabolomics analysis.

### 2.7. Metabonomic Analysis

Nontargeted metabolomics analysis was performed using UHPLC-Q-Exactive MS. For the analysis, we used a Dionex Ultimate 3000 UHPLC plus-focused ultraperformance liquid chromatograph coupled to a Thermo Scientific Orbitrap Elite mass spectrometer. The Waters ACQUITY UPLC HSS T3 (2.1 mm × 100 mm, 1.7 *μ*m) chromatographic column was used. The mobile phase consisted of (a) acetonitrile and (b) a solution of 0.1% formic acid. The flow rate was 0.3 mL/min, the column temperature was 35°C, and the injection volume was 3 *μ*L. The chromatographic gradient was optimized as follows: 0-12 min, 70%-15% B; 12.1-14 min, 15% B; 14.1-16 min, 15%-70% B; 16.1-17 min, 70% B. The system's post time was set to 10 minutes to equalize the system.

Mass spectrometry (MS) evaluations were conducted with UHPLC-Q-Exactive MS (Thermo Fisher Scientific, MA, USA). The following were the parameters for the positive ion mode: HESI served as the ion source, and other conditions included a 350°C temperature, high-purity nitrogen (purity >99.99%) for the sheath and auxiliary gases, a 30 arb sheath gas flow rate, a 10 arb auxiliary gas flow rate, a 3 kV ionization source voltage, a 35 V capillary voltage, and a 110 V tube lens voltage. HESI served as the ion source, the temperature was 350°C, and the sheath (30 arb) and auxiliary (10 arb) gases were both high-purity nitrogen (>99.99%) for the negative ion mode. The tube lens voltage was 110 V, while the capillary voltages and ionization source were adjusted to 35.0 V and 3.0 kV, respectively.

### 2.8. Metabonomic Data Extraction

ProteoWizard converted the raw data into mzML format, and the XCMS program was employed for retention time correction, peak extraction, and alignment. Each sample group's peaks with >50% missing data were filtered out using the “SVR” method after the peak areas were corrected [28]. Information on identifying metabolites was found by searching the lab's database and integrating metDNA and public library methods.

### 2.9. Preparation of Liver Proteomic Samples

Enzymatically digested protein samples in equal amounts were added with standard protein, mixed with TCA of a final concentration of 20%, vortexed for 30 s, and precipitated for 2 h at 4°C. After centrifuging the samples at 4500 g for 5 minutes, the precipitates were washed 2-3 times with acetone. The precipitate was then broken up by ultrasonic before being mixed with TEAB at a final concentration of 200 mM, trypsin at a 1 : 50 (protease: protein, m/m) ratio, followed by overnight enzymatic digestion. After adding a final concentration of 5 mM dithiothreitol (DTT), the temperature was decreased to 56°C for 30 min. The mixture was then incubated at room temperature for 15 min with iodoacetamide (IAA) at a final concentration of 11 mM.

### 2.10. TMT Labeling and Fractionation

With the aid of a Strata X C18 SPE column (Phenomenex), the trypsin-digested peptide was desalted and vacuum-dried. Following the manufacturer's instructions for the TMT kit, the peptide was processed after being reconstituted in 0.5 M TEAB. High-pH reverse-phase HPLC was used to separate the tryptic peptides into fractions using a Thermo BetaSil C18 column (5 *μ*m particles, 4.6 mm ID, and 250 mm length). First, peptides were divided into 60 fractions over 60 min using a gradient of 8%-32% acetonitrile (pH 9.0). The peptides were combined into 6 fractions and dried by vacuum centrifuging.

### 2.11. LC-MS and Proteomic Analysis

The EASY-nLC 1000 ultra-high-performance liquid phase system was used to separate the tryptic peptides. In the mobile phase, 2% acetonitrile and 0.1% formic acid (A) were mixed with 90% acetonitrile and 0.1% formic acid (B). The following procedures were used to elute the B gradient: 0-38 min, 8%-23% B; 38-52 min, 23%-35% B; 52-56 min, 35%-80% B; 56-59 min, 80% B. A constant flow rate of 550.00 nL/min was used. Following the NSI source, the peptides were exposed to tandem mass spectrometry (MS/MS) in Q ExactiveTM Plus (Thermo Fisher Scientific, MA, USA), which was coupled to the UPLC online. A high-resolution Orbitrap identified and analyzed peptide parent ions and secondary fragments at an ion source voltage of 2.2 kV. The m/z scan range for the full scan was 400-1500, and intact peptides were identified in the Orbitrap with a resolution of 70,000. The automatic gain control (AGC) was set to 5E4, the signal threshold was 3.8E4 ions/s, the maximum injection duration was 50 ms, and the dynamic exclusion period was 30 s for tandem mass spectrometry scans to avoid repeat scanning of the parent ion.

### 2.12. Database Search and Bioinformatics Analysis

MaxQuant search engine was used to process the generated MS/MS data (v.1.5.2.8). In the initial and main searches, the precursor ion mass tolerance was set to 20 ppm and 5 ppm, respectively, whereas the fragment ion mass tolerance was set to 0.02 Da.

The enrichment of differentially expressed protein with all identified proteins was examined using a two-tailed Fisher's exact test. The GO was deemed significant when the *P* value was corrected to < 0.05. The KAAS online service tool from the Kyoto Encyclopedia of Genes and Genomes (KEGG) was used to annotate the proteins. The KEGG mapper then matched the annotated proteins to the corresponding pathways in the database.

### 2.13. Transmission Electron Microscope Observation

Liver tissues were fixed with 4% glutaraldehyde at 4°C for 24 h, followed by 1% osmium for 2 h. The tissue was rinsed times for 5 min each with PBS, and then dehydrated twice using the following gradients: 50% acetone for 15 min, 70% acetone for 15 min, 90% acetone for 15 min, and 100% acetone for 10 min. After embedding with acetone and drying, 70 nm sections were performed with an ultrathin sectioning machine and attached to the copper grid. The sections were stained for 30 min with uranyl acetate solution and citric acid stain before being examined with transmission electron microscopy.

### 2.14. Western Blotting

The PMSF was added to the liver tissues and placed on a swinging tissue crusher for 1 min. Following the manufacturer's instructions for the BCA kit, the supernatant was collected and the protein concentration was calculated. Proteins were separated using 12% sodium dodecyl sulfate-polyacrylamide gel electrophoresis (SDS-PAGE), transferred to polyvinylidene fluoride (PVDF) membranes, and then immersed in a solution of 5% TBST skim milk powder at room temperature for 60 min to achieve nonspecific closure. The following primary antibodies were then incubated with the PVDF membranes for 12 h at 4°C: anti-SLC27A5 antibody (MA5-17175, Thermo Fisher; 1 : 2000), anti-CPT1A antibody (ab128568, Abcam; 1 : 1000), anti-NDUFB5 antibody (ab230215, Abcam; 1: 2000), anti-COX6A1 antibody (ab110265, Abcam; 1 : 1000), anti-cytochrome C antibody (ab230215, Abcam; 1 : 1000), and anti-ABCC3 antibody (39909 s, Cell Signaling Technology; 1 : 1000). After being washed three times with TBST, the PVDF membranes were incubated with secondary antibodies for one hour at room temperature. The membranes were then rinsed three times with TBST. Amersham Imager 680 UV was used to perform the exposures and analyze the grayscale values.

### 2.15. Statistical Analysis

In the metabonomic analysis, the statistical analyses were performed with the R program and included univariate statistical analysis such as Student's *t*-test and a multiplicative analysis of variance, multivariate statistical analysis such as principal component analysis (PCA), and orthogonal partial least squares discriminant analysis (OPLS-DA). Metabolites VIP > 1 and *P* value < 0.05 were saved.

According to the relative and absolute quantification ratios of the protein isobaric tags, the fold changes in proteins in the control and PFE groups were calculated to have a mean value for proteomic analysis. SPSS 25.0 Student's *t*-tests were used to evaluate if the differential proteins were substantially different (*P* < 0.05) across groups. As a result, proteins with a *P* value < 0.05 and a fold change >1.3 were selected as differentially expressed biomarkers.

The data were expressed as mean ± SD. GraphPad Prism version 9.0 (GraphPad Prism Software, CA, USA) was used to analyze quantitative data. The significance of the differences (variations) in means was evaluated using a one-way ANOVA. *P* < 0.05, *P* < 0.01, or *P* < 0.001 denote statistical significance levels.

## 3. Result

### 3.1. Analysis of the Compounds in PFE

The UHPLC-Q-Exactive MS method was used to analyze the chemical components in PFE. Over 50 compounds were identified from the extract, including coumarins (15 compounds), flavonoids (23 compounds), phenols (7 compounds), and glycosides (5 compounds) (Table S1). The potential hepatotoxic toxic components of PF, such as psoralidin, bakuchiol, neobavaisoflavone, bavachin, and bavachinin, were included [29].

### 3.2. Liver Injury Induced by PFE

**Figure 1:**
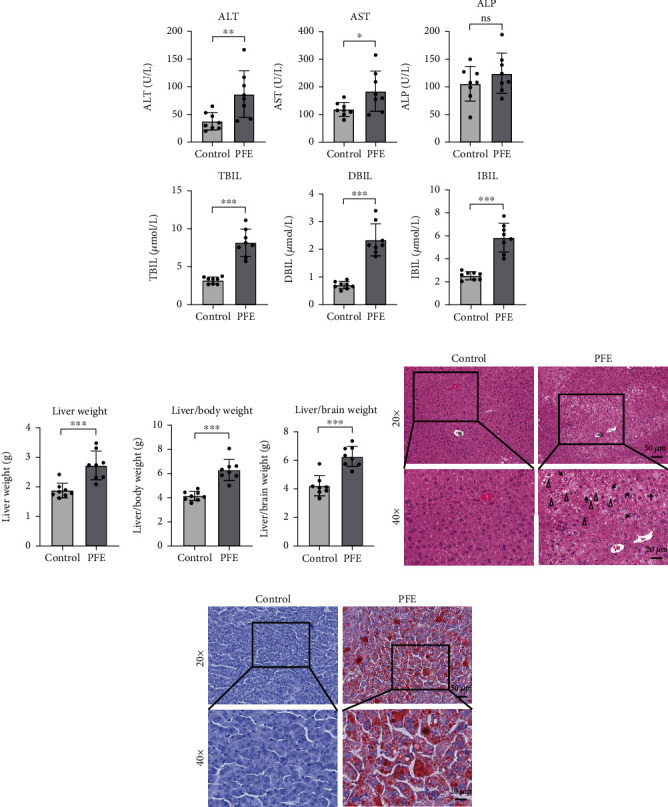
PFE-induced liver injury. (a) ALT, AST, ALP, TBIL, DBIL, and IBIL in all groups. (b) Liver weight, liver/body weight ratio, and liver/brain weight ratio in all groups. (c) Representative histopathological microphotographs of mice liver stained with H&E at magnifications of ×20 (top line) and ×40 (bottom line). (d) Representative histopathological microphotographs of mice liver stained with oil red O staining at magnifications of ×20 (top line) and ×40 (bottom line). The hepatic lobular vein is indicated with an asterisk. Hepatocyte hypertrophy is denoted with an arrow. Triangle denotes vacuolation. The data are expressed as mean ± SD, *n* = 10. ^∗^*P* < 0.05, ^∗∗^*P* < 0.01, and ^∗∗∗^*P* < 0.001 versus the control group.

Mice in the PFE group showed significantly (*P* < 0.05 and *P* < 0.01) higher levels of ALT and AST than mice in the control group (Figure 1(a)). Additionally, the liver weight in the PFE group mice was considerably higher (*P* < 0.01) (Figure 1(b)), as were the liver/brain weight and liver/body weight ratios, (*P* < 0.001 and *P* < 0.001) (Figure 1(b)). The results of liver histopathology showed that mice in the PFE group all had varying degrees of hepatocellular steatosis; hepatocellular hypertrophy was also observed in all mice (Figure 1(c)). These results suggested that PFE caused severe hepatotoxicity in mice. The hepatic lipid deposition was assessed using oil red O staining. The findings revealed that the livers of mice in the PFE group had a higher number of red lipid aggregates than that in the control group (Figure 1(d)). It is suggested that PFE may lead to disorders of lipid metabolism.

### 3.3. Metabolomic Profile Analysis of PFE-Induced Liver Injury

**Figure 2:**
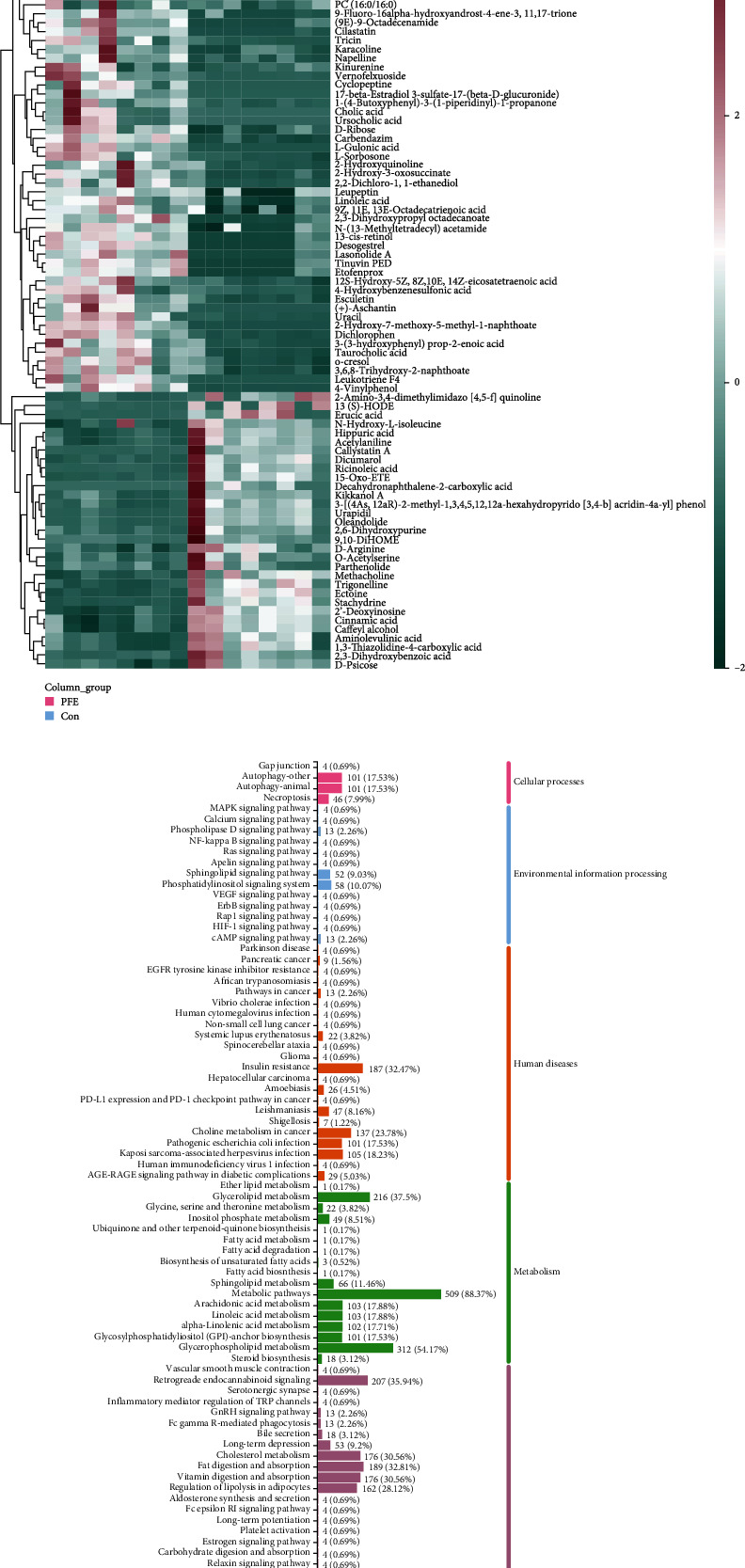
Metabolomic analysis of mice liver tissues after PEE administration. (a) PCA scores in positive and negative modes. (b) OPLS-DA scores in positive and negative modes. (c) Volcano plot. Red and blue dots indicate significantly increased and decreased proteins, respectively. *P* < 0.05, VIP > 1, and fold change > 2 or < 0.5. (d) The heat map shows 92 metabolites that were substantially altered in the liver were clustered in the control and PEE group. The red and blue represent increased and decreased metabolite content, respectively. (e) The KEGG pathway classification of differential proteins in the PFE group.

All serum samples were analyzed using UHPLC-Q-Exactive MS in both positive and negative ion modes. Multivariate analyses revealed a remarkable difference between the PEE and control groups. The control and PFE groups' profiles and the PCA model both indicated a trend toward separation (Figure 2(a)). The score matrix projection graph's central distribution and clustering of the QC samples show that the model is stable and reliable and effectively predicted grouping.

The score plot of OPLS-DA showed that the control and PFE groups could be discriminated in both positive and negative modes, which was utilized further to determine the differences in metabolites between the two groups (Figure 2(b)). We identified these metabolites using the results of MS/MS and information from an online database. PFE administration induced alterations in 92 differential metabolites of MS 2-level when the variable VIP > 1, *P* value < 0.05, and fold change > 2 or < 0.5. Then, we conducted a univariate analysis of metabolites and found that the administration of PFE induced changes in 92 differential metabolites relative to the control group when the variable VIP > 1, *P* value < 0.05, and fold change > 2 or < 0.5 (Figure S1). 31 metabolites were upregulated, such as benzene, acrylic acid, hippuric acid, and D-arginine, and 61 metabolites were significantly downregulated, such as leukotriene F4, taurocholic acid, and linoleic acid (Figure 2(c)). The retention time, metabolite formula, *P* value, and fold change of serum differential metabolites are listed in Supplementary Table S2. The heat map results showed that the samples of the PFE and control groups were in the same cluster, and the levels of the different metabolites in the PFE group were clearly distinguished from those in the control group. This also suggests that the 70% ethanolic extract of psoralen significantly alters metabolite levels in mice (Figure 2(d)). Based on the information from the KEGG database, we performed topology and pathway enrichment analyses to identify the metabolomic pathways that were involved. The identified biomarkers and related pathways are shown in Figure 2(e). Our findings mainly pointed to bile acid synthesis, lipid metabolism, glucose metabolism, amino acid metabolism, and degradation of aromatic compounds. Bile secretion, biosynthesis of primary bile acids, biosynthesis of secondary bile acids, biosynthesis of unsaturated fatty acids, cholesterol metabolism, linoleic acid metabolism, metabolism of xenobiotics by cytochrome P450, and degradation of aromatic compounds were significantly related to hepatotoxicity.

### 3.4. Comprehensive Proteomics Characterization of PFE-Related Hepatotoxicity

**Figure 3:**
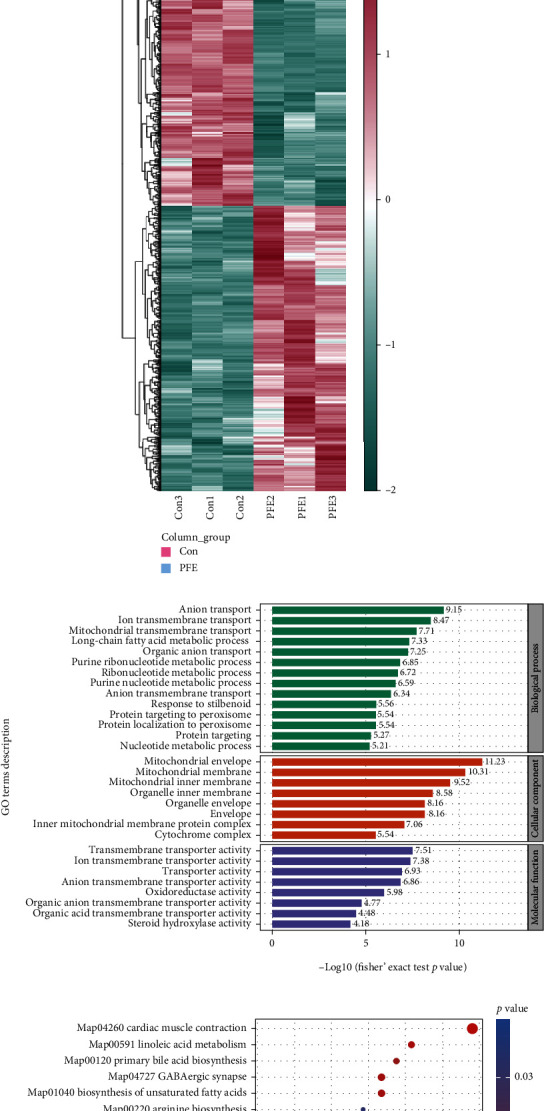
Proteomic analysis of mice liver tissues after PEE administration. (a) PCA scores. (b) Volcano plot. Blue and red dots represent significantly decreased and increased proteins, respectively. *P* < 0.05 and fold change > 1.3 or< 0.8. (c) The heat map shows that 762 proteins that were substantially altered in the liver were clustered in the control and PEE groups. The red and blue represent increased and decreased metabolite content, respectively. (d) GO analysis of differential proteins in the PFE group. (e) KEGG pathway analysis of differential proteins changed in the PFE group.

The proteomic profiles of PFE-induced liver injury in mice were examined by means of UHPLC-Q-Exactive MS. To study and visualize the discrepancies between PFE and control, PCA was applied to assess the data of the liver tissues, showing an obvious separation of PFE and control proteomic profiling and suggesting a significant difference between the protein patterns of the two groups (Figure 3(a)). Volcano plots were applied to show the differential proteins with the variable *P* < 0.05 and fold change > 1.3 or < 0.8. As a result, 762 proteins were detected, with 374 upregulated and 388 downregulated compared to the control group (Figure 3(b)). All the quantitative data on differential proteins were summarized in Supplementary Table S3. Then, we performed a clustering analysis of the proteins. As a result, the control and PFE samples were separated (Figure 3(c)). GO analysis revealed that differential proteins were clustered in mitochondrial transmembrane transport, long-chain fatty acid metabolic processes, purine ribonucleotide metabolic processes, and protein targeting to the peroxisome (Figure 3(d)).

The biological pathways linked to PFE-induced liver injury in mice were also identified using KEGG analysis. As shown in Figure 3(e), the identified proteins participated in different pathways, such as linoleic acid metabolism, oxidative phosphorylation, nonalcoholic fatty liver disease (NAFLD), unsaturated fatty acids biosynthesis, primary bile acid biosynthesis, and bile secretion.

### 3.5. Multiomics Analysis of Metabolomics and Proteomics of PFE-Related Hepatotoxicity

**Figure 4:**
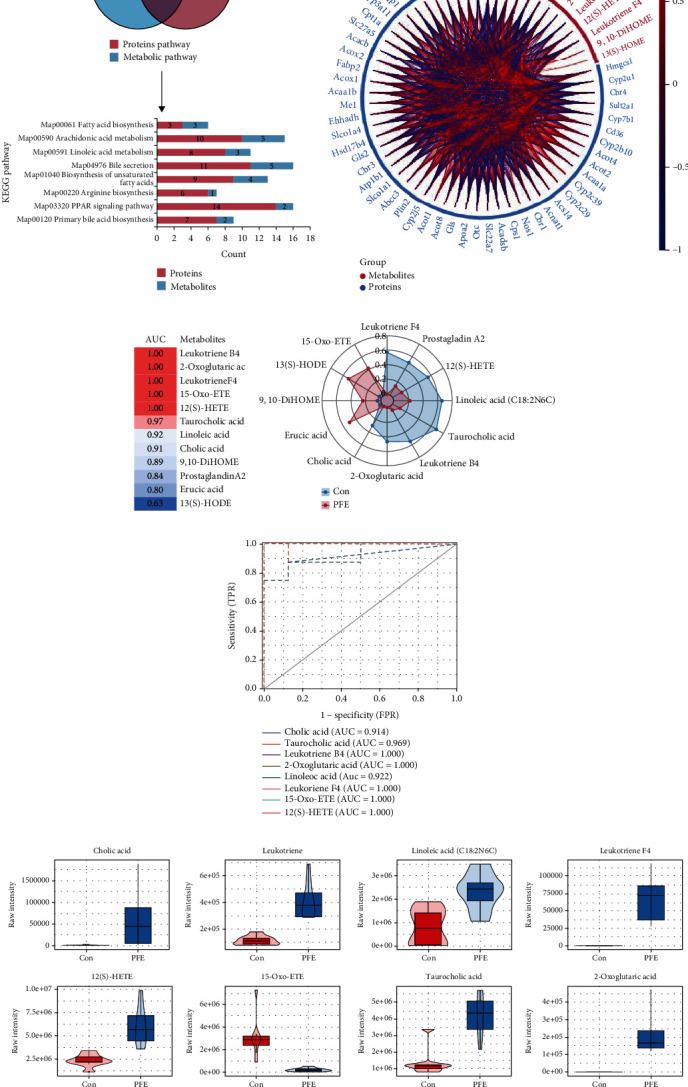
Association analysis of metabolomics and proteomics. (a) KEGG pathway in both metabolomics and proteomics. Pathways were present in both metabolomic and proteomic KEGG pathways, and *P* < 0.05. (b) Chord diagram for metabolites and proteins association analysis. Metabolites and proteins in the pathway of (a) diagram. (c) Heat map of AUC values of 12 differential metabolites. (d) Spider plots of 12 differential metabolites between the control and PEE groups. (e) ROC curves of differential metabolites with top 8 serum AUC values. (f) Quantitative analysis of 8 metabolites.

We conducted multiomics analyses for differential proteins and differential metabolites, respectively, to delve into any potential relationships between differential proteins and differential metabolites in the pathway. The results revealed 22 pathways enriched in both metabolomics and proteomics and 8 pathways with *P* < 0.05, such as bile secretion, unsaturated fatty acid biosynthesis, and linoleic acid metabolism (Figure 4(a)). There were complex associations between the differential metabolites and proteins enriched in these pathways (Figure 4(b)). We found that leukotriene F4, prostaglandin A2, 12(S)-HETE,14Z-eicosatetraenoic acid, linoleic acid, taurocholic acid leukotriene B4, 2-oxoglutaric acid, and cholic acid expression was significantly decreased after PFE exposure, and erucic acid, 9,10-DiHOME, 13(S)-HODE, and 15-oxo-ETE expression was significantly increased (Figure 4(c)). Eight serum metabolites with AUC values >0.9—leukotriene B4, 2-oxoglutaric acid, leukotriene F4, 15-oxo-ETE, 12(S)-HETE,14Z-eicosatetraenoic acid, taurocholic acid, linoleic acid, and cholic acid—may be biomarkers of PEE hepatotoxicity (Figures 4(d)–(f)).

### 3.6. PFE-Induced Liver Injury Associated with Lipid Metabolism Disorders

**Figure 5:**
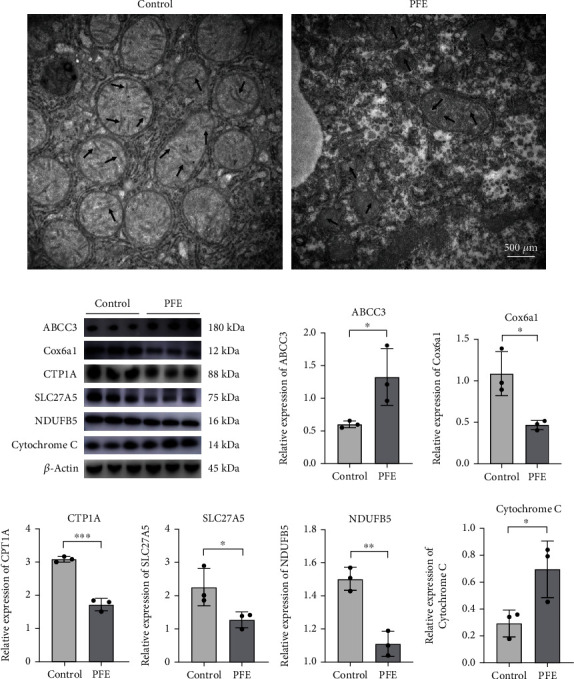
PFE interferes with lipid metabolism in mice's liver. (a) The ultrastructural changes of the liver mitochondria by transmission electron microscope, scale bars = 500 *μ*m. Arrows indicate mitochondrial cristae. (b) Western blot analysis of ABCC3, COX6A1, CPT1A, SLC27A5, NDUFB5, and cytochrome C expressions in the liver of mice. The values were expressed as mean ± SD, *n* = 3. ^∗^*P* < 0.05 and ^∗∗^*P* < 0.01 compared to the control group.

**Figure 6:**
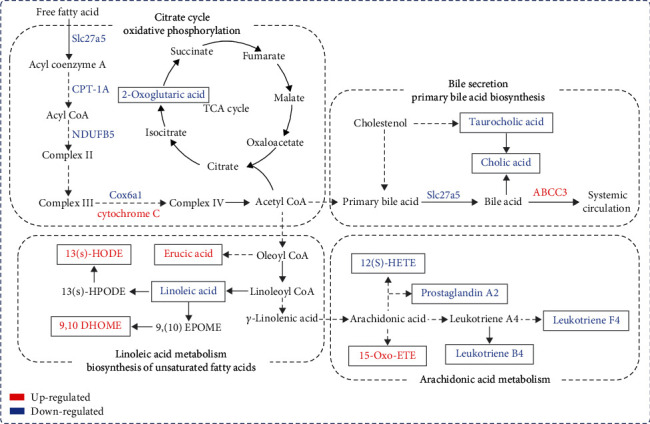
PFE caused hepatotoxicity by damaging mitochondria and causing lipid metabolism disorders in the liver of mice.

Mitochondria are the main sites of fat metabolism. To verify whether PFE induced liver injury by damaging the mitochondria and causing lipid metabolism disorders in the livers of mice, mitochondrial morphology was detected, and the typical indicators related to lipid metabolism screened by proteomics were determined, including ABCC3, COX6A1, CPT1A, SLC27A5, NDUFB5, and cytochrome C. Transmission electron microscope analysis showed that, at the ultrastructural level, the mitochondrial number was reduced, and mitochondrial cristae were shortened, broken, or disappeared in the PFE group of mice (Figure 5(a)). In addition, western blot assay showed that PFE exposure significantly suppressed COX6A1 and CPT1A expression in mice liver and inhibited SLC27A5 and NDUFB5 levels. Additionally, ABCC3 and cytochrome C were upregulated (Figure 5(b)). Hence, our data indicated that PFE induced hepatotoxicity by damaging mitochondria, reducing fatty acid *β*-oxidation levels, and inhibiting fatty acids ingested by bile acids (Figure 6).

## 4. Discussion

This study evaluated PFE hepatotoxicity and investigated its mechanism using multiomics analysis. This study demonstrates that PFE inhibited the expression of solute carrier family SLC27A5, CPT1A, NDUFB5, and COX6A1 and upregulated the cytochrome C and ABCC3 levels, and it indicated that PFE induced hepatotoxicity by damaging mitochondria, reducing fatty acid *β*-oxidation levels, and inhibiting fatty acids ingested by bile acids which were the potential toxicity mechanism of PFE hepatotoxicity (Figure 6).

The widespread use of CM, including Polygoni Multiflori Radix, Cortex Dictamni, and PF, has recently spurred research on HILI [30–34]. PF is frequently used in clinical practice. It was first mentioned in Treatise on the Preparation and Broiling of Materia Medica (Leigong Paozhi Lun) by Lei Gong. There is no report on PF's hepatotoxicity in ancient herbal books; however, current research has shown that PF is linked to liver damage [7–10, 35, 36]. This suggests that PF confers a risk of liver injury and is not in the category of nontoxic herbs.

Herb medicines are used in two forms in the clinic; one is in the form of compound Chinese traditional medicine, which is applied using water extraction in clinical applications. The other is in the form of Chinese patent medicines to serve the clinic. However, herb medicines are extracted from ethanol in some Chinese patent medicines. The extraction of some Chinese patent medicines involving PF, such as Xianniu Jiangu granules [37], and Bufei Huoxue capsule [38] was reported to induce serious liver damage and death. Our previous research demonstrated that the ethanol extract of PF was significantly more toxic than the water extract to rats and mice [15]. In this study, we again confirmed the hepatotoxicity of the ethanolic extraction of psoralen, which damaged hepatocytes, cytoplasm, and mitochondria and lead to the release of excessive ALT and AST. Meanwhile, liver histopathology showed that the livers of PFE mice showed hepatocellular steatosis, while oil red staining revealed the presence of a high number of fat droplet aggregates in the hepatocytes. Our previous study on the clinical characteristics of patients with PF-induced liver injury found that the injury type of most patients was hepatocellular injury [14]. The findings of this study were in agreement with those of our earlier investigation.

Lipids are a general group of essential components in living cells; studies have shown that drugs that considerably damage mitochondrial *β*-oxidation can cause microvesicular steatosis by accumulating free fatty acids and triglycerides. More serious microvesicular steatosis causes a diffuse accumulation of tiny fat droplets as its primary symptom [39, 40]. In this study, the livers of mice in the PFE group displayed a significant level of microvesicular steatosis. Then, the oil red O results supported the initial finding that the mice in the PFE group had a significant accumulation of small lipid droplets in their livers. In addition, our previous study suggested that the major hepatotoxic components of PF, including psoralidin, bakuchiol, neobavaisoflavone, bavachin, and bavachinin, caused hepatocyte injury, including lipid accumulation and cell apoptosis, by damaging mitochondria damage due to an abnormally elevated ROS level [29]. Therefore, we thought that the mechanism of PFE hepatotoxicity was related to abnormal lipid metabolism due to mitochondrial dysfunction.

The development of multiomics technology can solve the problem of unclear targets and unknown mechanisms in TCM to a certain extent. This study is aimed at performing a thorough analysis of endogenous metabolites in mice after drug administration using nontargeted metabolomics and to explore the mechanism of PF hepatotoxicity by analyzing the alteration of PF metabolic processes in mice. In mice, PFE induced significant alterations in the pathways regulating linoleic acid metabolism, unsaturated fatty acid production, and primary bile acid biosynthesis, as revealed by metabolomic and proteomic analyses. According to research, the four broad basic mechanisms linked to the induction of steatosis—increased fatty acid synthesis, increased fatty acid mobilization, and uptake decreased fatty acid *β*-oxidation and decreased lipoprotein exportation—could be aligned with the drug mechanisms that cause steatosis or intracellular lipid accumulation in drug-induced steatohepatitis [40]. Our histopathological results showed the presence of massive steatosis in the mice hepatocytes, and the oil red O results indicated that a large amount of fat accumulation was visible in the liver tissue. One mechanism that prevents excessive fat accumulation in the liver is increased mitochondrial oxidation of free fatty acids (FFAs), which only becomes faulty when respiration is severely compromised [41]. There might be a threshold above which fat infiltration leads to hepatocyte injury. Furthermore, inhibition of carnitine palmitoyltransferase I (CPT-1), inhibition of the mitochondrial respiratory chain, and disruption of mitochondrial DNA can all result in indirect inhibition of *β*-oxidation [37, 42–44]. The role of CPT1A, which is found in the outer mitochondrial membrane, is to transform lipid acyl-coenzyme A into lipoyl carnitine and coenzyme A, a key component of lipid metabolism that catalyzes the initial stage of fatty acid oxidation [45, 46]. SLC27A2 and SLC27A5 are the fatty acid transport proteins in hepatocytes [47]. SLC27A proteins (or fatty acid transport proteins, FATPs) have been proposed to function in fatty acid transport and activation either alone or in combination with a long-chain acyl CoA synthetase (Acsl: acyl CoA synthetase (ACS) of long-chain fatty acids) in fatty acid transport and activation [48]. In the first stage of the fatty acid *β*-oxidation pathway, SLC27A5 transforms fatty acids into acyl-coenzyme A. Our research found that PFE inhibited the expression of SLC27A5 and CPT1A, which suggested that there was a reduced fatty acid entrance into mitochondria.

Under aerobic conditions, most normal differentiated cells generate energy by mitochondrial oxidative phosphorylation (OXPHOS) and metabolize glucose to carbon dioxide through the mitochondrial tricarboxylic acid (TCA) cycle [49]. NDUFB5 is a mitochondrial membrane respiratory chain NADH dehydrogenase (Complex I) that helps transfer electrons from NADH to the respiratory chain [50]. In mice from the PFE group, NDUFB5 expression was inhibited, resulting in electron loss from the respiratory chain. COX6A1 is a cytochrome C oxidase component and the last enzyme in the mitochondrial electron transport chain, which powers oxidative phosphorylation. Cytochrome C represents the terminal step in the ETC; it is essential to energy metabolism because it regulates redox interactions with its homologs in the mitochondrial respiratory chain [51]. Cytochrome C is released into the cytoplasm as a result of mitochondrial damage. In this study, the decreased expression of COX6A1 and elevated cytochrome C suggested that the electron transport chain ends were blocked and ATP synthesis was impaired in mitochondria. The elevated cytochrome C also suggested that mitochondria were damaged, leading to the inhibition of fatty acid oxidative phosphorylation (Figure 5(b)). In addition, our transmission electron microscope analysis showed that the mitochondrial number was reduced and mitochondrial cristae were shortened, broken, or disappeared in the PFE group of mice. In addition, our previous study showed that the main toxic components of PF, such as bakuchiol, could lead to mitochondrial damage, as evidenced by the reduced mitochondrial membrane potential and increased ROS; these data confirmed that PFE caused mitochondrial dysfunction which led to the leaking of respiratory chain electron transport and inhibited fatty acid oxidative catabolism [29].

As a free unsaturated fatty acid, linoleic acid undergoes beta-oxidation during the metabolic process. Regulating the expression of genes involved in lipid synthesis and oxidation affects lipid metabolism [47, 52, 53]. In this study, we discovered that the level of linoleic acid metabolism in the liver of mice was reduced (Figure 6). This result was consistent with reduced levels of fatty acid *β*-oxidation, leading to lipid denaturation [54]. Linoleic acid can also be transformed into *γ*-linolenic acid, which was then converted to arachidonic acid to participate in arachidonic acid metabolism. In this report, in the PFE-treated mice, the decreased levels of phospholipase A2, leukotriene B4, and leukotriene F4 produced by arachidonic acid metabolism may be due to the reduced levels of linoleic acid metabolism.

In our study, we found that PFE's administration to mice also interfered with bile secretion and primary bile acid biosynthesis (Figure 6). Cholic acid is one of the components of bile acid, a crucial signaling molecule responsible for regulating energy, glucose, lipid metabolism, drug metabolism, and immune response modulation. According to our results, taurocholic acid and cholic acid levels were downregulated in the PFE group, and bile secretion was reduced. Bile acid levels decreased as taurocholic acid and cholic acid levels decreased. In addition, SLC27A5 is only highly expressed in the liver [49]. Bile acids block long-chain fatty acid uptake in a SLC27A5-dependent way and diminish lipid deposition in the liver to maintain lipid homeostasis [55]. PFE suppressed SLC27A5 expression and decreased the bile acids' ability to digest and absorb fatty acids. At the same time, ABCC3, which is located in the liver's basolateral membrane and translocates bile acids from hepatocytes to the hepatic portal vein [56], was upregulated in the PFE group. This increased the levels of bile acids that were transported to the blood. In long-term toxicity experiments, we also found elevated levels of DBIL, TBIL, and IBIL in mice serum, which is in line with the findings of multiomics studies.

In summary, this study clarified that impaired lipid oxidative catabolism due to mitochondrial damage is the main mechanism of PF hepatotoxicity, which suggested that mitochondria-targeted interventions may be a novel research strategy for hepatotoxicity of bone marrow lipids in future studies. In subsequent studies, drugs that protect mitochondria can be found to reduce or eliminate PF hepatotoxicity. This is one of the highlights of this study. Secondly, this study confirmed that the hepatotoxicity of PF was significant when it was extracted from 70% ethanol. This result suggested that it is necessary to avoid the 70% ethanol extraction process to ensure the safety of drug administration in the drug discovery and evaluation containing PF. It is the second highlight of this study. Finally, in conjunction with our previous study, hepatotoxicity was mild after four weeks of PF water extract administration in mice, but significant after four weeks of PFE administration, and these data helped us to find the safe dose range for PF and clarify the “dose-time-toxicity” relationship of it, which may help guide the safe and rational use of PF in clinical practice. It is the third highlight of this study.

## 5. Conclusion

In conclusion, we confirmed the hepatotoxicity of PF using in vivo experiments and explored the mechanism of hepatotoxicity using multiomics analysis. This study revealed that PFE induced hepatotoxicity by damaging mitochondria, reducing fatty acid *β*-oxidation levels, and inhibiting fatty acids ingested by bile acids. Changes in recognized metabolic biomarkers and proteins may aid in elucidating the mechanism underlying PF hepatotoxicity. Our results provided a reference for the clinical diagnosis of PF hepatotoxicity and suggested that it is necessary to avoid the 70% ethanol extraction process in drug discovery.

## Data Availability

Data are available from the author upon reasonable requests.

## Supplementary Materials

Supplementary Materials Figure S1: expressions of 92 metabolites in PFE group. Table S1: chemical composition identification of PFE. Table S2: table of 2-level metabolites information. Table S3: table of differential proteins information.
