# Supplementary material for: Psoraleae Fructus Ethanol Extract Induced Hepatotoxicity via Impaired Lipid Metabolism Caused by Disruption of Fatty Acid β-Oxidation
Source: Oxid Med Cell Longev. 2023 Jan 7;2023:4202861. doi: 10.1155/2023/4202861 (PMC9840557; doi:10.1155/2023/4202861)
Supplement: Supplementary Materials — Figure S1: expressions of 92 metabolites in PFE group. Table S1: chemical composition identification of PFE. Table S2: table of 2-level metabolites information. Table S3: table of differential proteins information. [file 4202861.f1.zip › R1-Table S2.docx]

**Table S2 Table of 2-level metabolites information**

| **Compounds** | **Formula** | **Precursor(g/mol)** | **Mass(g/mol)** | **score** | **RT(min)** | **MS_level** | **CAS_ID** | **HMDB_ID** | **cpd_ID** | **Con1** | **Con2** | **Con3** | **Con4** | **Con5** | **Con6** | **Con7** | **Con8** | **PFE1** | **PFE2** | **PFE3** | **PFE4** | **PFE5** | **PFE6** | **PFE7** | **PFE8** | **VIP** | **p_value** | **Fold_Change** | **Log2FC** | **Type** |
| --- | --- | --- | --- | --- | --- | --- | --- | --- | --- | --- | --- | --- | --- | --- | --- | --- | --- | --- | --- | --- | --- | --- | --- | --- | --- | --- | --- | --- | --- | --- |
| Cholic acid | C_24_H_40_O_5_ | 427.3247 | 408.2876 | 0.9722 | 6.7010 | 2-MetDNA | 81-25-4 | HMDB0000505 | C00695 | 1.0 | 1.0 | 1.0 | 1.0 | 18385.16 | 1.0 | 1.0 | 1.0 | 871551.88 | 870544.81 | 425481.93 | 1.0 | 3836.49 | 54710.91 | 1897200.5 | 455957.06 | 1.2170 | 0.0362 | 0.0852 | -3.5537 | down |
| Cinnamic acid | C_9_H_8_O_2_ | 132.0526 | 148.0524 | 0.9677 | 2.2552 | 2-MetDNA | 140-10-3 | HMDB0000930 | C00423 | 983124.15 | 1300223.61 | 1201367.02 | 1599106.94 | 1689770.42 | 1169754.26 | 1226488.35 | 893227.57 | 24523.22 | 360687.72 | 966993.8 | 484325.27 | 721118.08 | 261803.57 | 178191.83 | 335049.77 | 1.9209 | 0.0000 | 3.8797 | 1.9559 | up |
| Leukotriene B4 | C_20_H_32_O_4_ | 338.2405 | 336.2301 | 0.9606 | 7.0456 | 2-MetDNA | 71160-24-2 | HMDB0001085 | C02165 | 83982.29 | 140706.94 | 93277.12 | 124703.02 | 179223.36 | 87768.74 | 123518.85 | 77012.53 | 354400.73 | 402348.04 | 689978.0 | 445282.15 | 288506.47 | 548332.65 | 288984.26 | 292540.52 | 1.4943 | 0.0021 | 0.4012 | -1.3177 | down |
| D-Arginine | C_6_H_14_N_4_O_2_ | 175.1188 | 174.1117 | 0.9419 | 0.8942 | 2-MetDNA | 157-06-2 | HMDB0003416 | C00792 | 127580158.57 | 112113598.44 | 68026314.1 | 157633475.96 | 159465404.48 | 110377029.21 | 51861047.45 | 85214741.2 | 49732905.88 | 55895992.52 | 58538939.07 | 27418614.82 | 72068071.02 | 25590921.43 | 80559941.91 | 50508009.8 | 1.7789 | 0.0004 | 2.2088 | 1.1432 | up |
| Linoleic Acid(C18:2N6C) | C_18_H_32_O_2_ | 304.2347 | 280.2402 | 0.9413 | 14.0187 | 2-MetDNA | 60-33-3 | HMDB0000673 | C01595 | 231929.02 | 24781.23 | 47007.7 | 56388.69 | 1901960.08 | 1398631.64 | 1472121.97 | 1269580.21 | 2551224.21 | 3057172.62 | 2332132.49 | 1053291.36 | 1557297.01 | 3509657.06 | 2589545.04 | 2046568.92 | 1.6582 | 0.0007 | 0.3038 | -1.7189 | down |
| D-Ribose | C_5_H_10_O_5_ | 172.0309 | 150.0528 | 0.9322 | 1.169 | 2-MetDNA | 50-69-1 | HMDB00283 | C00121 | 172291.11 | 142105.97 | 400672.85 | 110157.66 | 64254.99 | 432732.46 | 537794.14 | 290623.59 | 975287.24 | 909743.49 | 698624.35 | 624372.43 | 695638.55 | 343426.24 | 1141096.43 | 801993.56 | 1.6451 | 0.0003 | 0.3482 | -1.5221 | down |
| O-Acetylserine | C_5_H_9_NO_4_ | 189.0195 | 147.0532 | 0.9309 | 1.2403 | 2-MetDNA | 5147-00-2 | HMDB0003011 | C00979 | 106234.04 | 27738.68 | 45873.35 | 85988.97 | 218271.14 | 37002.82 | 46833.36 | 19416.68 | 18196.18 | 43309.31 | 1.0 | 7142.41 | 27550.75 | 1.0 | 47827.47 | 24741.73 | 1.4869 | 0.0135 | 4.0749 | 2.0268 | up |
| Leukotriene F4 | C_28_H_44_N_2_O_8_S | 635.2345 | 568.2818 | 0.9115 | 10.1586 | 2-MetDNA | - | HMDB0006465 | C06462 | 1.0 | 1.0 | 1.0 | 1.0 | 1.0 | 1.0 | 1.0 | 1.0 | 99455.52 | 82309.13 | 118625.85 | 39728.22 | 77751.07 | 67596.39 | 31228.41 | 28272.59 | 1.9254 | 0.0001 | 0.0000 | -16.1054 | down |
| L-Gulonic acid | C_6_H_12_O_7_ | 431.0793 | 196.0583 | 0.903 | 8.1131 | 2-MetDNA | 526-97-6 | HMDB0003290 | C00800 | 1.0 | 1.0 | 1.0 | 1.0 | 1.0 | 1.0 | 1.0 | 1.0 | 73990.74 | 80147.33 | 74682.12 | 42696.06 | 32765.94 | 42897.86 | 98743.05 | 31891.27 | 1.8863 | 0.0003 | 0.0000 | -15.6630 | down |
| 2,6-Dihydroxypurine | C_5_H_4_N_4_O_2_ | 190.9822 | 152.033 | 0.8859 | 1.3995 | 2-MetDNA | 69-89-6 | HMDB0000292 | C00385 | 1152916.99 | 1184001.65 | 1108254.79 | 1803498.12 | 3481542.5 | 1025642.15 | 1415395.33 | 675982.94 | 926844.36 | 685281.36 | 1067538.27 | 645958.12 | 867024.14 | 569750.19 | 615571.95 | 566147.41 | 1.4505 | 0.0146 | 2.3642 | 1.2413 | up |
| N-Acetyl-D-tryptophan | C_13_H_14_N_2_O_3_ | 247.1076 | 246.1004 | 0.8843 | 1.8586 | 2-Search-DB | 138-79-8 | -- | C03137 | 10333096.52 | 10649439.0 | 6662011.52 | 16520613.02 | 16213548.37 | 7645810.06 | 6753101.72 | 8559840.52 | 26660298.68 | 28606078.61 | 42886300.0 | 14410364.02 | 23711333.69 | 30893261.54 | 29082404.63 | 18875837.58 | 1.5620 | 0.0013 | 0.4495 | -1.1536 | down |
| Cyclopeptine | C_17_H_16_N_2_O_2_ | 583.2326 | 280.1212 | 0.8825 | 8.9211 | 2-MetDNA | 50886-63-0 | -- | C20579 | 1.0 | 1.0 | 1.0 | 1.0 | 1.0 | 1.0 | 1.0 | 1.0 | 181272.56 | 209565.4 | 63246.95 | 1.0 | 97803.24 | 189780.16 | 349737.83 | 149889.2 | 1.6965 | 0.0013 | 0.0000 | -17.1177 | down |
| 2,3-Dihydroxypropyl octadecanoate | C_21_H_42_O_4_ | 341.3046 | 358.3083 | 0.8623 | 14.6752 | 2-Search-DB | 123-94-4 | -- | D01947 | 1.0 | 87410.45 | 1.0 | 6423.36 | 5365.17 | 4799.82 | 761421.86 | 1082630.76 | 1763815.95 | 1437823.36 | 2109493.06 | 3431956.77 | 2265688.34 | 2788467.1 | 1489312.25 | 1786895.52 | 1.9296 | 0.0000 | 0.0877 | -3.5109 | down |
| n-Oleoylethanolamine | C_20_H_39_NO_2_ | 326.3049 | 325.2981 | 0.8614 | 14.2363 | 2-Search-DB | 111-58-0 | HMDB0002088 | C20792 | 68837.12 | 5822133.62 | 83734.27 | 34432.88 | 1544438.52 | 2586761.09 | 4276996.72 | 3428592.41 | 5522367.16 | 5039204.91 | 9375887.7 | 5228082.11 | 3936127.24 | 5993950.72 | 4831334.14 | 3546004.68 | 1.5856 | 0.0024 | 0.3790 | -1.3996 | down |
| 3-Hydroxypyruvic acid | C_3_H_4_O_4_ | 148.977 | 104.01 | 0.8566 | 2.1194 | 2-MetDNA | 1113-60-6 | HMDB0001352 | C00168 | 1586369.38 | 1136178.62 | 1507605.65 | 1201452.1 | 4566150.19 | 1875763.12 | 1702101.82 | 1649305.13 | 2082298.71 | 6469836.13 | 5765259.56 | 6543315.96 | 14098567.65 | 2845739.33 | 6220746.11 | 1639922.69 | 1.2069 | 0.0164 | 0.3477 | -1.5240 | down |
| Indole-3-lactic acid | C_11_H_11_NO_3_ | 204.0667 | 205.0739 | 0.8411 | 1.9739 | 2-Search-DB | 1821-52-9 | HMDB0000671 | C02043 | 17771662.66 | 16577524.74 | 6796193.04 | 26536012.78 | 36902887.74 | 7305002.95 | 14706496.56 | 9632195.84 | 63871495.18 | 60151579.94 | 96498363.83 | 31617287.47 | 53932151.87 | 58620578.71 | 48678734.71 | 38777415.1 | 1.4811 | 0.0017 | 0.4120 | -1.2794 | down |
| 3-(3-hydroxyphenyl)prop-2-enoic acid | C_9_H_8_O_3_ | 163.0402 | 164.0473 | 0.8371 | 1.6843 | 2-Search-DB | 588-30-7 | HMDB0001713 | C12621 | 16339214.28 | 9260098.79 | 5323661.4 | 10447220.14 | 66401561.1 | 11426678.2 | 6362887.17 | 5086882.19 | 52978340.7 | 90008509.68 | 176196392.11 | 32550320.66 | 101038599.21 | 112491049.53 | 90176138.28 | 60003598.16 | 1.4779 | 0.0027 | 0.2669 | -1.9054 | down |
| Ectoine | C_6_H_10_N_2_O_2_ | 143.0814 | 142.0742 | 0.8345 | 0.8758 | 2-Search-DB | 96702-03-3 | -- | C06231 | 141369919.83 | 108924192.02 | 131981458.59 | 81298160.67 | 200560631.05 | 123006260.92 | 150685193.43 | 65760639.48 | 5402004.15 | 8941754.56 | 1098848.94 | 21075780.5 | 4883066.45 | 1677418.21 | 15795363.54 | 7488023.61 | 1.9003 | 0.0001 | 15.1425 | 3.9205 | up |
| 13-cis-Retinol | C_20_H_30_O | 269.2258 | 286.2297 | 0.8239 | 15.3806 | 2-Search-DB | 2052-63-3 | HMDB0006221 | C19962 | 46478.04 | 183050.22 | 81133.59 | 148311.71 | 162071.32 | 130104.26 | 5628767.65 | 3790014.31 | 11606832.63 | 11209715.25 | 12620961.56 | 6614143.83 | 11056165.94 | 9102215.41 | 9103951.14 | 8407486.3 | 2.0049 | 0.0000 | 0.1300 | -2.9431 | down |
| N-Hydroxy-L-isoleucine | C_6_H_13_NO_3_ | 163.113 | 147.0895 | 0.8234 | 8.5124 | 2-MetDNA | - | -- | C20310 | 497765.51 | 547020.63 | 512114.31 | 790038.5 | 967095.17 | 458916.07 | 579857.87 | 369159.82 | 303364.83 | 212122.47 | 55715.33 | 148947.54 | 277280.36 | 1136839.21 | 152251.28 | 180339.09 | 1.3588 | 0.0107 | 2.2761 | 1.1866 | up |
| Trigonelline | C_7_H_7_NO_2_ | 138.0548 | 137.0477 | 0.8214 | 0.8975 | 2-Search-Local | 535-83-1 | HMDB0000875 | C01004 | 21543912.34 | 15165755.91 | 24586369.76 | 14094903.44 | 30656012.06 | 20938847.61 | 21638766.35 | 10670637.54 | 7837868.82 | 6711449.12 | 9456972.93 | 7710030.6 | 5770242.9 | 5825052.35 | 5978267.37 | 8934283.61 | 1.7873 | 0.0005 | 2.4839 | 1.3126 | up |
| Aminolevulinic acid | C_5_H_9_NO_3_ | 178.0334 | 131.0582 | 0.8208 | 1.4289 | 2-MetDNA | 106-60-5 | HMDB0001149 | C00430 | 745299.63 | 510775.84 | 624032.72 | 1007224.36 | 1116085.85 | 489425.41 | 730291.73 | 59808.68 | 272818.62 | 170116.16 | 441617.55 | 4648.77 | 9237.96 | 11450.71 | 173199.68 | 91485.12 | 1.8268 | 0.0003 | 5.4299 | 2.4409 | up |
| PC(16:0/16:0) | C_40_H_80_NO_8_P | 756.5525 | 733.5622 | 0.8203 | 12.2527 | 2-Search-DB | 63-89-8 | HMDB0000564 | D03585 | 1.0 | 7390368.82 | 3565359.83 | 7940505.0 | 11479887.7 | 15367004.31 | 9762990.0 | 17517879.6 | 10946384.16 | 52201816.44 | 33174809.44 | 25190565.5 | 11092524.59 | 7749934.61 | 1785523.03 | 7493888.41 | 1.0692 | 0.0373 | 0.3526 | -1.5040 | down |
| 9Z,11E,13E-Octadecatrienoic acid | C_18_H_30_O_2_ | 279.2315 | 278.2246 | 0.8161 | 14.2203 | 2-Search-DB | 506-23-0 | -- | C08315 | 217712.31 | 16910608.74 | 620188.87 | 552710.78 | 24366778.64 | 11251384.55 | 16013366.69 | 11752430.17 | 20888334.55 | 30738595.77 | 23353761.89 | 11138337.86 | 20280519.48 | 24913694.77 | 23309869.94 | 16452863.77 | 1.5236 | 0.0021 | 0.3899 | -1.3588 | down |
| (9E)-9-Octadecenamide | C_18_H_35_NO | 282.2786 | 281.2719 | 0.8142 | 15.3498 | 2-Search-DB | 4303-70-2 | HMDB0002117 | C19670 | 152628.66 | 158644.9 | 133599.76 | 115991.48 | 136471.37 | 169004.25 | 1454554.51 | 1188281.9 | 4152588.24 | 4682245.5 | 3059338.62 | 1590882.56 | 1686860.33 | 1651111.74 | 1669010.29 | 2914268.61 | 1.8279 | 0.0002 | 0.1508 | -2.7289 | down |
| Hippuric acid | C_9_H_9_NO_3_ | 180.0655 | 179.0582 | 0.8135 | 1.4111 | 2-Search-DB | 140480-84-8 | HMDB0000714 | C01586 | 13689163.03 | 13249325.3 | 10817332.58 | 18104600.38 | 29402861.96 | 10606079.37 | 12278632.98 | 9675064.52 | 1020189.96 | 2921067.13 | 6380207.16 | 4292102.44 | 6342252.11 | 4266953.68 | 2295770.46 | 2816668.85 | 1.8675 | 0.0004 | 4.4411 | 2.1509 | up |
| 1,3-Thiazolidine-4-carboxylic acid | C_4_H_7_NO_2_S | 134.0271 | 133.0197 | 0.8108 | 1.4273 | 2-Search-DB | 444-27-9 | -- | D08601 | 10655756.49 | 8251720.51 | 7233469.63 | 12475804.88 | 13300916.58 | 5836690.9 | 9100677.12 | 970955.96 | 3780042.59 | 2562148.23 | 5481006.51 | 424484.74 | 441757.91 | 571077.58 | 2436692.74 | 1690022.56 | 1.8449 | 0.0002 | 4.6869 | 2.2286 | up |
| Kinurenine | C_10_H_12_N_2_O_3_ | 209.0918 | 208.0848 | 0.8048 | 1.1731 | 2-Search-Local | 343-65-7 | HMDB0000684 | C00328 | 167749.81 | 298223.7 | 219234.89 | 155696.62 | 33102.3 | 227050.96 | 485817.34 | 327830.84 | 824156.09 | 948077.33 | 1343403.9 | 144035.59 | 447997.69 | 38834.21 | 1182520.34 | 520542.03 | 1.2133 | 0.0285 | 0.4348 | -1.2015 | down |
| Tricin | C_17_H_14_O_7_ | 329.0672 | 330.074 | 0.8005 | 4.4597 | 2-Search-DB | 520-32-1 | HMDB0124861 | C10193 | 920466.94 | 701761.49 | 448238.8 | 1105141.9 | 2457575.84 | 552699.21 | 571072.91 | 426929.43 | 5296023.69 | 12787439.34 | 8083771.66 | 699608.52 | 7285644.09 | 2777450.65 | 9041544.69 | 5246172.35 | 1.3602 | 0.0038 | 0.2075 | -2.2685 | down |
| 2-Hydroxyquinoline | C_9_H_7_NO | 144.0456 | 145.0528 | 0.7922 | 2.5864 | 2-Search-DB | 104534-80-7 | -- | C06338 | 2637070.75 | 2368174.78 | 2189597.43 | 2579647.06 | 5745696.27 | 2168385.66 | 2369558.72 | 1950245.86 | 7203164.92 | 5848601.42 | 4630256.08 | 4531256.65 | 6351773.95 | 12606097.49 | 5466794.61 | 5499698.8 | 1.2348 | 0.0105 | 0.4846 | -1.0452 | down |
| Leupeptin | C_20_H_38_N_6_O_4_ | 427.3025 | 426.2955 | 0.7904 | 14.1011 | 2-Search-DB | 24365-47-7 | -- | C01591 | 5025.75 | 20918.5 | 5018.8 | 1.0 | 1097807.39 | 1126141.03 | 1069552.86 | 1046355.51 | 870055.44 | 1039110.99 | 1154280.63 | 1000341.99 | 919223.87 | 1278131.51 | 1169619.11 | 1154349.6 | 1.4021 | 0.0101 | 0.4313 | -1.2133 | down |
| 13(S)-HODE | C_18_H_32_O_3_ | 295.2196 | 296.2351 | 0.7877 | 11.6578 | 2-Search-DB | 29623-28-7 | HMDB0061708 | C14762 | 20738.3 | 1168592.99 | 1548232.33 | 37662.39 | 1170396.68 | 1120840.41 | 24287.2 | 1390727.04 | 125804.07 | 42111.02 | 143161.37 | 50545.79 | 47972.72 | 78628.36 | 63775.21 | 45938.61 | 1.0743 | 0.0181 | 10.1191 | 3.3390 | up |
| 9,10-DiHOME | C_18_H_34_O_4_ | 337.2343 | 314.2457 | 0.7858 | 8.5124 | 2-Search-DB | 263399-34-4 | HMDB0004704 | C14828 | 7177994.09 | 5269916.64 | 3998873.05 | 6797365.39 | 23880059.04 | 5377633.89 | 5016187.68 | 2522664.2 | 3901018.35 | 3858957.15 | 3462816.27 | 2305095.25 | 2839812.03 | 2619783.32 | 3598832.09 | 2657410.41 | 1.3607 | 0.0324 | 2.6853 | 1.4251 | up |
| Ricinoleic acid | C_18_H_34_O_3_ | 297.2438 | 298.2508 | 0.7757 | 14.5443 | 2-Search-DB | 141-22-0 | HMDB0034297 | C08365 | 16045793.77 | 11237129.55 | 12269509.89 | 15207560.07 | 32288184.79 | 8488616.62 | 13895036.62 | 5875510.83 | 4169940.97 | 4497660.31 | 4356234.83 | 3259915.97 | 4699010.94 | 4925648.6 | 4035999.55 | 3213522.37 | 1.7334 | 0.0026 | 3.8463 | 1.9435 | up |
| Karacoline | C_22_H_35_NO_4_ | 378.2608 | 377.2566 | 0.775 | 15.7065 | 2-Search-DB | 39089-30-0 | -- | C08693 | 265037.48 | 284030.14 | 227142.4 | 1.0 | 1.0 | 1.0 | 82730.29 | 7925.86 | 488402.26 | 1178703.06 | 414098.36 | 236695.88 | 353407.91 | 393037.66 | 476668.13 | 462129.72 | 1.2646 | 0.0107 | 0.3838 | -1.3817 | down |
| Parthenolide | C_15_H_20_O_3_ | 249.1484 | 248.1412 | 0.7711 | 4.9236 | 2-Search-DB | 20554-84-1 | -- | C07609 | 3037590.68 | 1858294.77 | 1097661.83 | 4244059.76 | 6695918.67 | 1577663.62 | 962609.97 | 968215.96 | 1631090.39 | 1633407.18 | 886806.2 | 629992.61 | 2219091.19 | 1138952.56 | 2159727.22 | 1387218.41 | 1.3317 | 0.0371 | 2.1626 | 1.1128 | up |
| o-Cresol | C_7_H_8_O | 107.0503 | 108.0575 | 0.7704 | 1.7214 | 2-Search-DB | 95-48-7 | HMDB0002055 | C01542 | 573370.68 | 563977.31 | 470661.05 | 912764.05 | 1248930.01 | 454043.58 | 574645.09 | 305446.91 | 1944821.09 | 1646647.07 | 2353983.58 | 906447.41 | 2410183.09 | 2186634.84 | 1512750.11 | 1406795.99 | 1.3103 | 0.0068 | 0.4952 | -1.0138 | down |
| Esculetin | C_9_H_6_O_4_ | 177.0194 | 178.0266 | 0.7688 | 4.5695 | 2-Search-DB | 305-01-1 | HMDB0030819 | C09263 | 4435533.84 | 6367436.74 | 4570428.93 | 6306906.19 | 14745253.54 | 3724363.74 | 9117542.61 | 2522116.55 | 43444916.03 | 32581411.76 | 24328167.83 | 15105708.0 | 15040522.61 | 30735696.57 | 37572908.07 | 24221888.27 | 1.7223 | 0.0004 | 0.2510 | -1.9944 | down |
| Ursocholic acid | C_24_H_40_O_5_ | 407.2807 | 408.2876 | 0.7666 | 6.6271 | 2-Search-Local | 2955-27-3 | HMDB0000917 | C17644 | 17207.61 | 13751.71 | 3483.74 | 16133.35 | 103927.79 | 14346.56 | 6954.48 | 7886.47 | 3655835.47 | 3093470.44 | 1603945.22 | 18641.16 | 69639.7 | 246557.21 | 6426403.98 | 1531734.18 | 1.2756 | 0.0277 | 0.0741 | -3.7545 | down |
| Pyruvic acid | C_3_H_4_O_3_ | 172.977 | 88.016 | 0.7654 | 1.7867 | 2-MetDNA | 127-17-3 | HMDB0000243 | C00022 | 781046.75 | 481902.03 | 691133.71 | 728050.2 | 2066044.29 | 513332.78 | 887175.74 | 412573.34 | 1355978.4 | 2613411.14 | 4420390.45 | 2736716.62 | 2357624.74 | 3134737.21 | 1701697.78 | 1273354.34 | 1.4238 | 0.0033 | 0.3884 | -1.3642 | down |
| 12S-Hydroxy-5Z,8Z,10E,14Z-eicosatetraenoic acid | C_20_H_32_O_3_ | 303.2315 | 320.2351 | 0.7644 | 11.1331 | 2-Search-DB | 54397-83-0 | -- | C14777 | 1927319.76 | 2221112.03 | 2618526.52 | 3413060.39 | 2958296.23 | 2258317.58 | 2263947.46 | 1109784.47 | 5972607.1 | 7699655.16 | 5350100.99 | 3933853.28 | 4601929.53 | 9890226.69 | 7010859.65 | 3574923.83 | 1.3502 | 0.0105 | 0.4867 | -1.0388 | down |
| Levocetirizine | C_21_H_25_ClN_2_O_3_ | 387.1488 | 388.1554 | 0.7614 | 4.3012 | 2-Search-DB | 130018-77-8 | HMDB0240226 | D07402 | 13270.34 | 3730.61 | 1.0 | 35603.84 | 105287.12 | 1.0 | 1.0 | 1.0 | 4839589.44 | 5087344.57 | 13937949.23 | 423807.98 | 3775032.65 | 2944603.76 | 4642183.06 | 3177090.94 | 1.4949 | 0.0085 | 0.0053 | -7.5464 | down |
| 2-Amino-3,4-dimethylimidazo[4,5-f]quinoline | C_12_H_12_N_4_ | 213.1134 | 212.1062 | 0.7533 | 1.7654 | 2-Insilico | 77094-11-2 | HMDB0029707 | C19254 | 1800488.49 | 1410563.35 | 1078089.66 | 3673807.83 | 568432.2 | 155851.01 | 3943743.98 | 3506123.56 | 1007606.14 | 527263.75 | 47554.91 | 52812.34 | 24429.23 | 54004.52 | 120867.38 | 255653.27 | 1.0092 | 0.0331 | 9.4016 | 3.2329 | up |
| 4-Vinylphenol | C_8_H_8_O | 121.065 | 120.0575 | 0.753 | 5.8228 | 2-Search-DB | 2628-17-3 | HMDB0004072 | C05627 | 128736.94 | 131980.71 | 153356.57 | 87622.27 | 227697.86 | 182185.16 | 125770.02 | 147057.41 | 6071928.0 | 4060423.61 | 4179653.19 | 2048534.95 | 4867638.07 | 4133365.26 | 2748722.46 | 3879017.75 | 1.9831 | 0.0000 | 0.0400 | -4.6427 | down |
| 4-Hydroxybenzenesulfonic acid | C_6_H_6_O_4_S | 172.9915 | 173.9987 | 0.7515 | 1.3995 | 2-Search-DB | 98-67-9 | -- | C12849 | 42140020.52 | 49930118.16 | 53297055.45 | 69770577.14 | 93804798.5 | 69410570.13 | 56058924.21 | 20631387.62 | 245517559.25 | 190822045.8 | 222171095.58 | 35700489.32 | 71675064.93 | 325278592.12 | 228987816.69 | 105946154.31 | 1.4778 | 0.0052 | 0.3819 | -1.3887 | down |
| 15-Oxo-ETE | C_20_H_30_O_3_ | 341.2058 | 318.2195 | 0.7447 | 13.2275 | 2-Search-DB | 81416-72-0 | HMDB0010210 | C04577 | 3090316.68 | 2608046.31 | 3409404.02 | 3139849.71 | 7281007.17 | 1690714.97 | 2703318.62 | 900256.67 | 114832.29 | 287634.52 | 19914.29 | 163041.42 | 507370.29 | 103820.03 | 176783.98 | 321282.72 | 1.8125 | 0.0014 | 17.6087 | 4.1382 | up |
| Uracil | C_4_H_4_N_2_O_2_ | 111.0201 | 112.0273 | 0.7426 | 0.9862 | 2-Search-DB | 66-22-8 | HMDB0000300 | C00106 | 4847259.47 | 5313586.24 | 4611666.36 | 6840398.65 | 6363767.21 | 4637520.05 | 4819221.77 | 4176370.65 | 31084356.57 | 25855545.24 | 15870829.35 | 21809782.22 | 13960191.21 | 30719808.06 | 26308693.3 | 5787886.04 | 1.6803 | 0.0005 | 0.3202 | -1.6431 | down |
| Stachydrine | C_7_H_13_NO_2_ | 144.1018 | 143.0946 | 0.7389 | 0.892 | 2-Search-Local | 471-87-4 | HMDB0004827 | C10172 | 9338824.01 | 6542612.37 | 8232522.11 | 6480276.74 | 16142352.81 | 7469948.79 | 9366501.71 | 4049012.56 | 1018339.75 | 1354582.94 | 967254.62 | 1535386.61 | 1143370.61 | 1413041.8 | 1599599.83 | 1030302.44 | 1.8038 | 0.0006 | 6.4406 | 2.6872 | up |
| Taurocholate;Taurocholic acid;Cholyltaurine | C_26_H_45_NO_7_S | 533.3255 | 515.2917 | 0.7384 | 4.0749 | 2-Search-Local | 81-24-3 | HMDB0000036 | C05122 | 1286566.94 | 1193969.26 | 1020542.38 | 1165892.54 | 3381426.07 | 902714.94 | 1200368.06 | 795582.01 | 3147017.3 | 3851508.0 | 4952716.66 | 2178182.07 | 4898456.84 | 5541989.6 | 5745576.91 | 3475402.96 | 1.5924 | 0.0006 | 0.3775 | -1.4053 | down |
| 2-Hydroxy-7-methoxy-5-methyl-1-naphthoate | C_13_H_12_O_4_ | 231.0663 | 232.0736 | 0.7332 | 5.5654 | 2-Insilico | - | -- | C20841 | 1.0 | 1.0 | 1.0 | 3333.98 | 1.0 | 1.0 | 1.0 | 1.0 | 396102.53 | 358595.13 | 397407.35 | 205444.58 | 275822.55 | 425668.27 | 372452.08 | 168318.06 | 2.0234 | 0.0000 | 0.0012 | -9.7495 | down |
| Desogestrel | C_22_H_30_O | 311.2364 | 310.2297 | 0.7266 | 14.9242 | 2-Search-DB | 54024-22-5 | HMDB0014449 | C07629 | 1.0 | 1.0 | 5036.11 | 18508.02 | 1.0 | 5682.99 | 2562118.32 | 2448844.68 | 4501187.47 | 5873642.82 | 5878606.85 | 3831769.19 | 3997281.4 | 5288446.14 | 4176829.82 | 4008808.55 | 2.0524 | 0.0000 | 0.1169 | -3.0965 | down |
| Osalmid | C_13_H_11_NO_3_ | 252.0606 | 229.0739 | 0.7258 | 1.7511 | 2-Search-DB | 526-18-1 | HMDB0003156 | D01579 | 723558.19 | 717243.58 | 451373.61 | 1242724.69 | 940749.48 | 442543.55 | 413939.28 | 628892.55 | 1711373.72 | 1698943.33 | 2904683.96 | 1413722.29 | 1661302.92 | 1708708.02 | 1820736.92 | 1385286.76 | 1.5292 | 0.0012 | 0.4517 | -1.1465 | down |
| Methacholine | C_8_H_17_NO_2_ | 160.1331 | 159.1259 | 0.7197 | 0.8603 | 2-Search-DB | 62-51-1 | HMDB0015654 | C07471 | 323045012.85 | 421209898.73 | 446546698.94 | 296716438.76 | 624722698.69 | 536613624.03 | 465326259.77 | 395768449.17 | 266271163.02 | 217328845.59 | 168745676.06 | 248345592.95 | 251927564.15 | 204502827.07 | 194916743.94 | 208005499.75 | 1.8908 | 0.0000 | 2.1060 | 1.0745 | up |
| Cilastatin | C_16_H_26_N_2_O_5_S | 359.166 | 358.1562 | 0.7156 | 15.3445 | 2-Search-DB | 82009-34-5 | HMDB0015535 | C01675 | 1.0 | 1.0 | 1.0 | 1.0 | 1.0 | 1.0 | 69973.52 | 7244.02 | 525608.9 | 709658.37 | 400193.22 | 285921.29 | 283434.93 | 285621.04 | 329800.98 | 343955.22 | 2.0015 | 0.0000 | 0.0202 | -5.6268 | down |
| Arnottin II | C_20_H_14_O_7_ | 365.0680 | 366.074 | 0.7155 | 4.1945 | 2-Insilico | - | -- | C12258 | 90820.15 | 54584.19 | 66643.87 | 64105.0 | 313658.62 | 57472.87 | 110650.66 | 4199.37 | 4725723.79 | 1927590.42 | 28424442.41 | 2482411.63 | 8214920.99 | 2322900.93 | 4528891.25 | 4680027.59 | 1.1236 | 0.0496 | 0.0178 | -5.8152 | down |
| 2'-Deoxyinosine | C_10_H_12_N_4_O_4_ | 273.0579 | 252.0859 | 0.7125 | 2.2202 | 2-MetDNA | 890-38-0 | HMDB0000071 | C05512 | 292992.2 | 421902.02 | 388869.94 | 569991.84 | 542866.41 | 402965.01 | 417557.2 | 315646.87 | 1.0 | 65027.66 | 79414.59 | 115668.23 | 212925.51 | 52531.14 | 38115.79 | 82672.25 | 2.0516 | 0.0000 | 6.6913 | 2.7423 | up |
| 2-Oxoglutaric acid | C_5_H_6_O_5_ | 293.0542 | 146.0215 | 0.7120 | 1.1471 | 2-MetDNA | 328-50-7 | HMDB0000208 | C00026 | 1.0 | 1.0 | 1.0 | 1.0 | 1.0 | 1.0 | 1.0 | 1.0 | 165355.59 | 168841.14 | 470596.59 | 186161.47 | 389220.17 | 142650.43 | 125897.01 | 119965.23 | 1.7399 | 0.0003 | 0.0272 | -5.2018 | down |
| Kikkanol A | C_15_H_26_O_3_ | 253.1812 | 254.1882 | 0.7097 | 9.8286 | 2-Insilico | - | -- | C17603 | 13096652.05 | 11458150.6 | 12866514.93 | 15726249.89 | 45568358.57 | 14233360.98 | 14622218.14 | 5388760.45 | 11940687.71 | 6163658.87 | 3071116.04 | 1416269.08 | 2370316.3 | 1445409.58 | 8388861.04 | 4145823.16 | 1.5707 | 0.0063 | 3.9494 | 1.9816 | up |
| Caffeyl alcohol | C_9_H_10_O_3_ | 131.0492 | 166.063 | 0.7042 | 2.2563 | 2-Search-DB | 3598-26-3 | HMDB0141765 | C12206 | 10684796.57 | 14003904.11 | 12576126.66 | 17477961.63 | 17913135.24 | 12763526.89 | 13321639.82 | 9566259.15 | 662106.42 | 3863492.17 | 10761179.93 | 5449207.45 | 7667010.94 | 2658055.44 | 2690709.03 | 3338674.99 | 1.9230 | 0.0000 | 3.6937 | 1.8851 | up |
| Oxaloglycolate | C_4_H_4_O_6_ | 186.9562 | 148.0008 | 0.7032 | 1.876 | 2-MetDNA | - | -- | C03459 | 1967361.83 | 1642065.6 | 2120809.62 | 1710139.41 | 5496268.54 | 1708298.34 | 4323647.43 | 1771607.6 | 7995010.43 | 5674565.71 | 9979756.69 | 8088242.34 | 6780139.72 | 16253135.19 | 8680266.96 | 4168769.11 | 1.5383 | 0.0023 | 0.3522 | -1.5057 | down |
| 3,6,8-Trihydroxy-2-naphthoate | C_11_H_8_O_5_ | 219.0277 | 220.0372 | 0.703 | 1.7194 | 2-Insilico | - | -- | C21320 | 135307.17 | 126911.5 | 122979.65 | 223410.78 | 329539.58 | 81003.68 | 171790.49 | 72783.36 | 568000.12 | 368057.68 | 538066.82 | 179250.7 | 396478.32 | 549650.71 | 352623.88 | 334404.92 | 1.3463 | 0.0059 | 0.4696 | -1.0905 | down |
| Dichlorophen | C_13_H_10_Cl_2_O_2_ | 266.9949 | 268.0058 | 0.6929 | 1.262 | 2-Search-DB | 97-23-4 | -- | C14292 | 480945.18 | 822106.35 | 519067.93 | 683689.1 | 1606796.71 | 1031854.93 | 453401.83 | 714314.58 | 7605242.94 | 5371730.92 | 6933171.44 | 3065645.5 | 4089755.64 | 7192594.95 | 7989400.4 | 4143657.31 | 1.8525 | 0.0003 | 0.1588 | -2.6545 | down |
| Napelline | C_22_H_33_NO_3_ | 360.2503 | 359.246 | 0.6847 | 15.7072 | 2-Search-DB | 5008-52-6 | -- | C08700 | 114746.95 | 3062.96 | 133349.89 | 75178.41 | 1.0 | 1.0 | 13089.8 | 1.0 | 315275.28 | 647274.03 | 272716.03 | 165295.26 | 154736.65 | 167285.92 | 184427.45 | 305470.57 | 1.6458 | 0.0008 | 0.1599 | -2.6450 | down |
| 1-(4-Butoxyphenyl)-3-(1-piperidinyl)-1-propanone | C_18_H_27_NO_2_ | 290.2088 | 289.2042 | 0.6784 | 7.6934 | 2-Search-DB | 586-60-7 | HMDB0014783 | C07881 | 157229.79 | 217448.53 | 656717.88 | 344905.77 | 829710.3 | 605684.0 | 274449.47 | 371922.84 | 3773348.01 | 1630031.21 | 1737476.02 | 1682488.55 | 1708350.69 | 492762.03 | 5581553.95 | 2483676.03 | 1.1099 | 0.0276 | 0.3198 | -1.6449 | down |
| Decahydronaphthalene-2-carboxylic acid | C_11_H_18_O_2_ | 200.1642 | 182.1307 | 0.66 | 15.249 | 2-Search-DB | 13032-41-2 | -- | C14113 | 1115192.15 | 709222.83 | 2295900.41 | 1421737.34 | 6998645.58 | 2838275.92 | 1.0 | 1.0 | 1.0 | 4437.44 | 1.0 | 1.0 | 1.0 | 1.0 | 1.0 | 22671.19 | 1.4109 | 0.0255 | 617.8506 | 9.2711 | up |
| 2,3-Dihydroxybenzoic acid | C_7_H_6_O_4_ | 153.0195 | 154.0266 | 0.6508 | 1.6944 | 2-Search-Local | 303-38-8 | HMDB0000397 | C00196 | 10901292.11 | 8104271.58 | 6705023.83 | 19186392.8 | 23129624.12 | 7214235.4 | 7770816.13 | 3553905.19 | 2938681.22 | 3041369.27 | 4251710.23 | 4886601.71 | 3687420.02 | 3892761.26 | 2746833.47 | 2266492.51 | 1.6109 | 0.0095 | 3.3856 | 1.7594 | up |
| cis-5,6-Dihydroxy-4-isopropylcyclohexa-1,3-dienecarboxylic acid | C_10_H_14_O_4_ | 197.0821 | 198.0892 | 0.6494 | 1.8872 | 2-Insilico | - | -- | C06579 | 622103.7 | 1257899.92 | 574634.04 | 1071308.21 | 811728.06 | 629156.92 | 608531.86 | 1813259.58 | 4359044.58 | 2869976.03 | 5637562.42 | 1841995.54 | 1669507.0 | 2657785.73 | 1590159.19 | 1801325.75 | 1.2745 | 0.0219 | 0.4774 | -1.0668 | down |
| L-Sorbosone;1-Dehydro-L-sorbose | C_6_H_10_O_6_ | 198.0465 | 178.0477 | 0.6422 | 1.2577 | 2-MetDNA | - | -- | C19679 | 123444.49 | 132551.93 | 129153.83 | 159396.7 | 165798.14 | 159696.83 | 129642.42 | 71401.75 | 519849.49 | 484087.02 | 560648.17 | 337953.05 | 453570.08 | 190163.72 | 619075.4 | 260819.49 | 1.6147 | 0.0015 | 0.4002 | -1.3214 | down |
| N-Acetyl-L-phenylalanine | C_11_H_13_NO_3_ | 208.0968 | 207.0895 | 0.6396 | 1.7532 | 2-Search-Local | 2018-61-3 | HMDB0000512 | C03519 | 5342129.67 | 5390492.18 | 3299452.89 | 9396320.96 | 7299848.5 | 3514883.3 | 3446280.86 | 4832351.07 | 11832523.53 | 12862811.36 | 21863684.35 | 9845114.36 | 12532620.82 | 12839671.23 | 13106661.23 | 9526598.75 | 1.4708 | 0.0023 | 0.4715 | -1.0846 | down |
| Tinuvin PED | C_13_H_11_N_3_O | 267.1221 | 225.0902 | 0.6335 | 15.5046 | 2-Search-DB | 2440-22-4 | -- | D03913 | 4489.14 | 1.0 | 4720.15 | 1.0 | 1.0 | 4127.24 | 653171.87 | 674997.28 | 1517023.66 | 1277186.87 | 1267604.77 | 1014296.96 | 880451.14 | 1188670.5 | 995001.19 | 1526063.51 | 2.0523 | 0.0000 | 0.1145 | -3.1260 | down |
| Erucic acid | C_22_H_42_O_2_ | 356.3515 | 338.3185 | 0.625 | 15.3113 | 2-Search-DB | 112-86-7 | HMDB0002068 | C08316 | 7091065.55 | 5203079.19 | 7377464.21 | 1545956.47 | 3585820.54 | 5088545.02 | 240573.81 | 336547.7 | 288442.32 | 386773.75 | 392285.34 | 323518.37 | 353103.48 | 326294.12 | 544681.77 | 633125.78 | 1.5274 | 0.0019 | 10.6810 | 3.4170 | up |
| Dicumarol | C_19_H_12_O_6_ | 335.0601 | 336.0634 | 0.6187 | 5.5229 | 2-Search-DB | 66-76-2 | HMDB0014411 | C00796 | 5039803.11 | 4738223.52 | 5722089.42 | 6002229.68 | 13395940.55 | 3724955.58 | 7019826.1 | 2622770.48 | 1326496.87 | 758273.4 | 395750.68 | 1108688.54 | 715612.83 | 1659436.74 | 871075.05 | 596117.66 | 1.8295 | 0.0008 | 7.4255 | 2.8925 | up |
| Vernoflexuoside | C_21_H_28_O_8_ | 407.1717 | 408.1784 | 0.6145 | 2.7175 | 2-Insilico | 57576-33-7 | -- | C09579 | 160998.02 | 181447.75 | 268713.58 | 70997.45 | 718011.2 | 150927.28 | 372361.17 | 64513.85 | 2803735.02 | 4675604.3 | 8709222.54 | 1664980.51 | 2083936.49 | 1769266.78 | 7874411.48 | 2167359.51 | 1.6087 | 0.0031 | 0.0705 | -3.8264 | down |
| N-(13-Methyltetradecyl)acetamide | C_17_H_35_NO | 270.2786 | 269.2719 | 0.6105 | 15.6577 | 2-Insilico | 64317-66-4 | HMDB0040940 | C17515 | 243771.89 | 253411.86 | 245701.8 | 94064.66 | 176507.99 | 754777.13 | 613947.04 | 493228.56 | 846115.5 | 843601.53 | 789202.64 | 637567.15 | 587299.66 | 729149.38 | 553159.86 | 715620.17 | 1.8388 | 0.0003 | 0.4692 | -1.0918 | down |
| Callystatin A | C_29_H_44_O_4_ | 455.3174 | 456.324 | 0.6089 | 10.7041 | 2-Insilico | 189883-16-7 | -- | C16891 | 1082079.69 | 898081.26 | 935965.81 | 677279.4 | 3735207.97 | 1060001.61 | 1086018.21 | 514416.81 | 1.0 | 42208.48 | 80378.14 | 5740.1 | 146639.84 | 7044.68 | 25279.05 | 55554.1 | 1.6300 | 0.0044 | 32.6966 | 5.0311 | up |
| Lasonolide A | C_41_H_60_O_9_ | 679.4170 | 696.4237 | 0.6085 | 15.5019 | 2-Insilico | - | -- | C19903 | 1.0 | 1.0 | 1.0 | 1.0 | 1.0 | 1.0 | 9165819.44 | 8336195.72 | 22902029.25 | 31340669.59 | 16620973.18 | 13102981.62 | 14439355.98 | 17008153.92 | 18435736.21 | 32551414.38 | 1.9336 | 0.0000 | 0.0879 | -3.5084 | down |
| Carbendazim | C_9_H_9_N_3_O_2_ | 214.0562 | 191.0695 | 0.6046 | 0.7198 | 2-Search-DB | 10605-21-7 | HMDB0031769 | C10897 | 2520083.55 | 3055249.14 | 2519130.94 | 2680099.55 | 2536869.25 | 4081088.67 | 2246775.94 | 2703702.76 | 6055577.39 | 7261662.55 | 6994875.76 | 7602344.19 | 5915992.75 | 6413463.52 | 8733275.1 | 5933990.89 | 1.8610 | 0.0000 | 0.4884 | -1.0339 | down |
| 2,2-Dichloro-1,1-ethanediol | C_2_H_4_Cl_2_O_2_ | 171.9929 | 129.9588 | 0.591 | 1.8962 | 2-Insilico | 16086-14-9 | HMDB0062193 | C14860 | 24637190.9 | 21440072.28 | 25914014.48 | 21196575.21 | 60913324.73 | 22161778.35 | 34277112.47 | 22463214.1 | 37848577.28 | 91521048.2 | 75903636.04 | 105299310.83 | 77313221.03 | 184167671.42 | 110410146.63 | 55142809.6 | 1.3496 | 0.0055 | 0.4023 | -1.3136 | down |
| 17-beta-Estradiol 3-sulfate-17-(beta-D-glucuronide) | C_24_H_32_O_11_S | 527.16 | 528.1665 | 0.5837 | 8.1943 | 2-Insilico | - | HMDB0010358 | C11289 | 1.0 | 3849.27 | 1.0 | 11680.65 | 32638.36 | 1.0 | 1.0 | 1.0 | 3986320.25 | 5063099.25 | 3928053.02 | 2001036.6 | 1969682.62 | 5240542.33 | 13423405.39 | 3757917.92 | 1.6084 | 0.0029 | 0.0011 | -9.8533 | down |
| Chrysoobtusin | C_19_H_18_O_7_ | 357.1021 | 358.1053 | 0.5836 | 6.8851 | 2-Insilico | 70588-06-6 | HMDB0034218 | C17669 | 50651.2 | 43137.97 | 42965.53 | 95116.06 | 191215.11 | 10971.42 | 51881.31 | 13546.0 | 7379525.92 | 10701069.05 | 29009727.71 | 4604438.19 | 17611890.89 | 7111901.22 | 14803726.3 | 8335324.43 | 1.5957 | 0.0034 | 0.0059 | -7.3960 | down |
| Prostaglandin A2 | C_20_H_30_O_4_ | 299.1952 | 334.2144 | 0.5811 | 14.9918 | 2-Search-DB | 13345-50-1 | HMDB0002752 | C05953 | 1.0 | 1.0 | 1.0 | 1.0 | 1.0 | 1.0 | 678215.21 | 578369.81 | 1096035.64 | 799542.86 | 697122.44 | 355069.89 | 434832.44 | 433110.38 | 308810.83 | 341390.32 | 1.6135 | 0.0022 | 0.2298 | -2.1218 | down |
| (+)-Aschantin | C_22_H_24_O_7_ | 399.1488 | 400.4270 | 0.5764 | 3.6138 | 2-Insilico | - | - | C17845 | 56590.92 | 54645.15 | 47865.38 | 66492.09 | 162360.64 | 36019.75 | 51497.33 | 33824.48 | 12565397.56 | 6602993.88 | 3820925.82 | 2792062.48 | 2970125.64 | 6613004.95 | 6624745.62 | 4570018.13 | 1.8168 | 0.0004 | 0.0127 | -6.3006 | down |
| 2,4,6-Triaminotoluene | C_7_H_11_N_3_ | 138.1024 | 137.0953 | 0.5624 | 15.2402 | 2-Insilico | 88-02-8 | -- | C16400 | 5407340.83 | 4669470.63 | 5290065.64 | 2072920.96 | 3486773.22 | 1717983.54 | 4054155.88 | 4219643.23 | 4313403.75 | 9644897.11 | 19850425.45 | 7115147.52 | 6782716.35 | 5918905.39 | 5060666.48 | 4348000.67 | 1.3003 | 0.0172 | 0.4740 | -1.0771 | down |
| Acetylaniline | C_8_H_9_NO | 134.0613 | 135.0684 | 0.5586 | 1.3724 | 2-Search-DB | 103-84-4 | HMDB0001250 | C07565 | 2251933.53 | 1937325.89 | 1667692.76 | 2729851.78 | 4789884.92 | 1640036.69 | 1997435.34 | 1521137.78 | 137978.47 | 480079.19 | 1266710.71 | 735734.27 | 924097.54 | 694459.32 | 358401.4 | 454137.1 | 1.8029 | 0.0006 | 4.2065 | 2.0726 | up |
| D-Psicose | C_6_H_12_O_6_ | 179.0545 | 180.0634 | 0.5521 | 1.3704 | 2-Search-DB | 551-68-8 | -- | C06468 | 9145711.33 | 7918509.97 | 6903697.19 | 14177680.51 | 17293273.12 | 6683702.08 | 7954025.85 | 5796541.67 | 5490234.72 | 4307765.92 | 4638247.83 | 3001053.21 | 74107.61 | 2924049.24 | 4278028.84 | 1501379.26 | 1.7323 | 0.0010 | 3.1859 | 1.6717 | up |
| 9-Fluoro-16alpha-hydroxyandrost-4-ene-3,11,17-trione | C_19_H_23_FO_4_ | 376.1926 | 334.158 | 0.5506 | 15.7052 | 2-Insilico | - | -- | C15105 | 1.0 | 1.0 | 1.0 | 151160.65 | 1.0 | 1.0 | 28890.52 | 16825.88 | 221613.8 | 428639.12 | 219447.97 | 185942.43 | 153933.6 | 169225.52 | 141707.63 | 206873.26 | 1.5250 | 0.0004 | 0.2426 | -2.0434 | down |
| 3-[(4As,12aR)-2-methyl-1,3,4,5,12,12a-hexahydropyrido[3,4-b]acridin-4a-yl]phenol | C_23_H_24_N_2_O | 343.1766 | 344.1889 | 0.5292 | 5.2015 | 2-Search-DB | - | -- | C20168 | 1582316.96 | 1160862.7 | 1486024.7 | 1937687.05 | 4095985.85 | 1019213.67 | 1789760.51 | 328396.05 | 1022871.86 | 430663.97 | 342611.33 | 79322.65 | 106041.83 | 42560.86 | 207237.49 | 287955.09 | 1.6856 | 0.0032 | 5.6077 | 2.4874 | up |
| Glyoxylic acid | C_2_H_2_O_3_ | 158.9613 | 74.0004 | 0.525 | 1.508 | 2-MetDNA | 298-12-4 | HMDB0000119 | C00048 | 6626054.43 | 18385404.16 | 36801251.24 | 118391210.85 | 32161120.88 | 24187632.1 | 26605884.47 | 37974344.07 | 78082301.27 | 70319014.25 | 273321067.31 | 85083079.31 | 150476535.95 | 61435572.39 | 89455280.69 | 44778855.39 | 1.2376 | 0.0105 | 0.3436 | -1.5413 | down |
| Urapidil | C_20_H_29_N_5_O_3_ | 386.2273 | 387.227 | 0.5237 | 7.8253 | 2-Search-DB | 34661-75-1 | -- | D01333 | 7146522.98 | 5593332.83 | 6774009.2 | 7485153.24 | 22002054.58 | 5761339.88 | 8284703.23 | 1723059.87 | 2654950.62 | 904482.34 | 594898.99 | 312280.38 | 300873.89 | 157047.03 | 522722.31 | 674778.31 | 1.7020 | 0.0032 | 11.4197 | 3.5135 | up |
| Oleandolide | C_20_H_34_O_7_ | 385.2239 | 386.2305 | 0.5133 | 7.8253 | 2-Insilico | 68540-16-9 | -- | C11990 | 32661108.7 | 25377291.52 | 30528941.65 | 33876894.86 | 99143765.81 | 26219729.07 | 37831254.85 | 7755473.86 | 12008236.94 | 4381853.46 | 2823264.33 | 1500030.09 | 1419126.75 | 766463.82 | 2419507.01 | 3300937.04 | 1.7064 | 0.0030 | 11.1299 | 3.4764 | up |
| Thymine | C_5_H_6_N_2_O_2_ | 125.0356 | 126.0429 | 0.5076 | 2.2506 | 2-Search-DB | 65-71-4 | HMDB0000262 | C00178 | 52074.63 | 72438.97 | 11007.38 | 11494.83 | 1.0 | 21347.25 | 36866.1 | 37104.01 | 693458.31 | 388882.82 | 656936.65 | 109536.03 | 75996.04 | 78617.24 | 24095.2 | 35640.25 | 1.3647 | 0.0107 | 0.0958 | -3.3842 | down |
| Etofenprox | C_25_H_28_O_3_ | 415.1743 | 376.2038 | 0.5059 | 15.5085 | 2-Search-DB | 80844-07-1 | -- | C18410 | 1.0 | 1.0 | 1.0 | 1.0 | 1.0 | 1.0 | 281964.53 | 215404.67 | 569926.36 | 476729.12 | 477686.4 | 448424.31 | 373916.79 | 443877.35 | 426068.56 | 625865.62 | 2.0556 | 0.0000 | 0.1106 | -3.1763 | down |
