# Supplementary material for: Psoraleae Fructus Ethanol Extract Induced Hepatotoxicity via Impaired Lipid Metabolism Caused by Disruption of Fatty Acid β-Oxidation
Source: Oxid Med Cell Longev. 2023 Jan 7;2023:4202861. doi: 10.1155/2023/4202861 (PMC9840557; doi:10.1155/2023/4202861)
Supplement: Supplementary Materials — Figure S1: expressions of 92 metabolites in PFE group. Table S1: chemical composition identification of PFE. Table S2: table of 2-level metabolites information. Table S3: table of differential proteins information. [file 4202861.f1.zip › R1-Table S3.docx]

**Table S3 Table of differential proteins information**

| **MW [kDa]** | **Coverage [%]** | **Peptides** | **Unique peptides** | **PSMs** | **Con1** | **Con2** | **Con3** | **PFE1** | **PFE2** | **PFE3** | **Subcellular localization** | **KEGG KO No.** | **KEGG Gene** | **KEGG pathway** | **Pfam ID** | **Domain description** | **KOG category** | **KOG NO.** | **KOG description** |
| --- | --- | --- | --- | --- | --- | --- | --- | --- | --- | --- | --- | --- | --- | --- | --- | --- | --- | --- | --- |
| 61.761 | 32.9 | 15 | 15 | 33 | 0.754 | 0.882 | 0.742 | 1.592 | 1.351 | 1.243 | nucleus | K01067 | E3.1.2.1, ACH1; acetyl-CoA hydrolase [EC:3.1.2.1] | map00620 Pyruvate metabolism; map01100 Metabolic pathways | PF03061; PF01852 | Thioesterase superfamily; START domain | I | KOG2763 | Acyl-CoA thioesterase |
| 69.56 | 7.4 | 3 | 3 | 3 | 1.539 | 1.537 | 1.562 | 0.863 | 0.552 | 0.457 | plasma membrane | K03846 | ALG9; alpha-1,2-mannosyltransferase [EC:2.4.1.259 2.4.1.261] | map00510 N-Glycan biosynthesis; map00513 Various types of N-glycan biosynthesis; map01100 Metabolic pathways | | | G | KOG2515 | Mannosyltransferase |
| 126.18 | 4 | 4 | 4 | 4 | 0.736 | 0.636 | 0.759 | 1.242 | 1.325 | 1.481 | nucleus | K08854 | BMP2K, BIKE; BMP2 inducible kinase [EC:2.7.11.1] | map05202 Transcriptional misregulation in cancer | PF00069 | Protein kinase domain | T | KOG1989 | ARK protein kinase family |
| 61.936 | 12.8 | 7 | 5 | 9 | 0.808 | 0.878 | 0.81 | 1.1 | 1.281 | 1.11 | cytoplasm |  |  |  | PF01979 | Amidohydrolase family | F | KOG2584 | Dihydroorotase and related enzymes |
| 55.873 | 4.4 | 2 | 2 | 2 | 0.998 | 0.727 | 0.481 | 1.719 | 1.226 | 1.646 | nucleus | K16513 | NOS1AP, CAPON; carboxyl-terminal PDZ ligand of neuronal nitric oxide synthase protein | map04713 Circadian entrainment | PF00640 | Phosphotyrosine interaction domain (PTB/PID) | T | KOG4815 | Muscular protein implicated in muscular dystrophy phenotype |
| 93.85 | 2.5 | 2 | 2 | 2 | 0.724 | 0.766 | 0.78 | 1.162 | 1.118 | 1.046 | cytoplasm | K19759 | DNAAF5; dynein assembly factor 5, axonemal | | | |  |  |  |
| 29.884 | 46.6 | 12 | 12 | 40 | 0.827 | 0.823 | 0.741 | 1.495 | 1.257 | 1.265 | mitochondria | K11147 | DHRS4; dehydrogenase/reductase SDR family member 4 [EC:1.1.-.-] | map00830 Retinol metabolism; map01100 Metabolic pathways; map04146 Peroxisome | PF13561 | Enoyl-(Acyl carrier protein) reductase | Q | KOG0725 | Reductases with broad range of substrate specificities |
| 23.307 | 4.4 | 1 | 1 | 3 | 1.219 | 1.684 | 1.049 | 0.836 | 0.509 | 0.614 | plasma membrane | |  |  |  |  | I | KOG4826 | C-8,7 sterol isomerase |
| 36.384 | 27.2 | 9 | 9 | 11 | 0.802 | 0.916 | 0.882 | 1.042 | 1.35 | 1.068 | cytoplasm | K17089 | ANXA3; annexin A3 | |  |  | U | KOG0819 | Annexin |
| 39.807 | 10.4 | 4 | 3 | 5 | 0.764 | 0.664 | 0.751 | 1.387 | 1.055 | 1.253 | nucleus | K09512 | DNAJB6; DnaJ homolog subfamily B member 6 | | PF00226 | DnaJ domain | O | KOG0714 | Molecular chaperone (DnaJ superfamily) |
| 75.363 | 2.2 | 1 | 1 | 1 | 0.801 | 0.981 | 0.887 | 1.174 | 1.235 | 1.074 | cytoplasm | K23336 | ARMC8; armadillo repeat-containing protein 8 | | | | S | KOG1293 | Proteins containing armadillo/beta-catenin-like repeat |
| 49.656 | 22.1 | 9 | 3 | 20 | 0.587 | 0.545 | 0.533 | 2.218 | 1.393 | 1.189 | mitochondria | K01068 | ACOT1_2_4; acyl-coenzyme A thioesterase 1/2/4 [EC:3.1.2.2] | map00062 Fatty acid elongation; map01040 Biosynthesis of unsaturated fatty acids; map01100 Metabolic pathways | PF08840 | BAAT / Acyl-CoA thioester hydrolase C terminal | | | |
| 46.584 | 64.8 | 27 | 27 | 153 | 1.167 | 1.267 | 1.082 | 0.781 | 0.829 | 0.905 | cytoplasm | K01940 | argG, ASS1; argininosuccinate synthase [EC:6.3.4.5] | map00220 Arginine biosynthesis; map00250 Alanine, aspartate and glutamate metabolism; map01100 Metabolic pathways;  map01230 Biosynthesis of amino acids; map05418 Fluid shear stress and atherosclerosis | | | E | KOG1706 | Argininosuccinate synthase |
| 146.9 | 35.7 | 42 | 39 | 153 | 1.17 | 1.35 | 1.189 | 0.599 | 0.786 | 0.726 | cytoplasm | K00157 | AOX; aldehyde oxidase [EC:1.2.3.1] | map00280 Valine, leucine and isoleucine degradation; map00350 Tyrosine metabolism; map00380 Tryptophan metabolism; map00750 Vitamin B6 metabolism; map00760 Nicotinate and nicotinamide metabolism; map00830 Retinol metabolism; map00982 Drug metabolism - cytochrome P450; map01100 Metabolic pathways; map04630 JAK-STAT signaling pathway | PF00111; PF01799; PF03450; PF01315 | 2Fe-2S iron-sulfur cluster binding domain; [2Fe-2S] binding domain; CO dehydrogenase flavoprotein C-terminal domain; Aldehyde oxidase and xanthine dehydrogenase, a/b hammerhead domain | F | KOG0430 | Xanthine dehydrogenase |
| 87.051 | 0.9 | 1 | 1 | 1 | 0.741 | 0.893 | 0.618 | 1.176 | 0.972 | 1.57 | cytoplasm | K05729 | ARHGEF6, PIXA; Rho guanine nucleotide exchange factor 6 | map04810 Regulation of actin cytoskeleton; map05212 Pancreatic cancer | PF00307; PF00621; PF00169 | Calponin homology (CH) domain; RhoGEF domain; PH domain | Z | KOG2070 | Guanine nucleotide exchange factor |
| 10.344 | 26.1 | 2 | 2 | 12 | 1.087 | 1.128 | 1.172 | 0.764 | 0.815 | 0.896 | cytoplasm | K02130 | ATPeF0F, ATP5J2; F-type H+-transporting ATPase subunit f | map00190 Oxidative phosphorylation; map01100 Metabolic pathways; map04714 Thermogenesis | | | C | KOG4092 | Mitochondrial F1F0-ATP synthase, subunit f |
| 11.987 | 25.9 | 3 | 3 | 4 | 1.543 | 1.59 | 1.404 | 0.752 | 0.736 | 0.754 | cytoplasm,nucleus | K18175 | CCDC56, COA3; cytochrome c oxidase assembly factor 3, animal type | map04714 Thermogenesis | | | S | KOG4782 | Predicted membrane protein |
| 7.3325 | 28.6 | 2 | 2 | 7 | 1.615 | 1.676 | 1.508 | 0.7 | 0.502 | 0.638 | mitochondria | K02272 | COX7C; cytochrome c oxidase subunit 7c | map00190 Oxidative phosphorylation; map01100 Metabolic pathways;  map04260 Cardiac muscle contraction; map04714 Thermogenesis; map04932 Non-alcoholic fatty liver disease (NAFLD);  map05010 Alzheimer disease; map05012 Parkinson disease; map05016 Huntington disease | | | C | KOG4527 | Cytochrome c oxidase, subunit VIIc/COX8 |
| 15.531 | 14.3 | 2 | 2 | 2 | 0.591 | 0.607 | 0.358 | 1.504 | 0.81 | 1.714 | extracellular | K13899 | CST3; cystatin-C | map04970 Salivary secretion | PF00031 | Cystatin domain | |  |  |
| 11.605 | 55.2 | 6 | 6 | 24 | 1.528 | 1.522 | 1.472 | 0.749 | 0.829 | 0.755 | mitochondria | K08738 | CYC; cytochrome c | map01524 Platinum drug resistance; map04115 p53 signaling pathway; map04210 Apoptosis; map04214 Apoptosis - fly; map04215 Apoptosis - multiple species; map04932 Non-alcoholic fatty liver disease (NAFLD); map05010 Alzheimer disease; map05012 Parkinson disease; map05014 Amyotrophic lateral sclerosis (ALS); map05016 Huntington disease; map05130 Pathogenic Escherichia coli infection; map05131 Shigellosis; map05134 Legionellosis; map05145 Toxoplasmosis; map05152 Tuberculosis; map05160 Hepatitis C; map05161 Hepatitis B; map05162 Measles; map05163 Human cytomegalovirus infection; map05164 Influenza A; map05167 Kaposi sarcoma-associated herpesvirus infection; map05168 Herpes simplex virus 1 infection; map05169 Epstein-Barr virus infection; map05170 Human immunodeficiency virus 1 infection; map05200 Pathways in cancer; map05210 Colorectal cancer; map05222 Small cell lung cancer; map05416 Viral myocarditis | PF00034 | Cytochrome c | C | KOG3453 | Cytochrome c |
| 60.938 | 26.9 | 15 | 15 | 27 | 1.792 | 1.452 | 1.551 | 0.59 | 0.644 | 0.577 | plasma membrane | K07427 | CYP4V2; docosahexaenoic acid omega-hydroxylase [EC:1.14.14.79] | | PF00067 | Cytochrome P450 | Q | KOG0157 | Cytochrome P450 CYP4/CYP19/CYP26 subfamilies |
| 46.07 | 23.6 | 8 | 3 | 14 | 0.79 | 0.97 | 0.752 | 1.428 | 1.281 | 1.344 | cytoplasm | K00659 | BAAT; bile acid-CoA:amino acid N-acyltransferase [EC:2.3.1.65 3.1.2.2] | map00120 Primary bile acid biosynthesis; map00430 Taurine and hypotaurine metabolism; map01040 Biosynthesis of unsaturated fatty acids; map01100 Metabolic pathways; map04146 Peroxisome; map04976 Bile secretion | PF08840 | BAAT / Acyl-CoA thioester hydrolase C terminal | | | |
| 38.676 | 57.2 | 19 | 19 | 29 | 0.822 | 0.77 | 0.908 | 1.092 | 1.175 | 1.262 | cytoplasm | K17092 | ANXA2; annexin A2 | |  |  | U | KOG0819 | Annexin |
| 6.3814 | 43.1 | 2 | 2 | 7 | 1.407 | 1.542 | 1.314 | 0.668 | 0.734 | 0.692 | cytoplasm | K18194 | USMG5, DAPIT; up-regulated during skeletal muscle growth protein 5 | | | | | |  |
| 12.228 | 38.2 | 4 | 4 | 5 | 1.383 | 1.43 | 1.33 | 0.86 | 0.877 | 0.922 | mitochondria | K22075 | BOLA3; BolA-like protein 3 | | |  | T | KOG3348 | BolA (bacterial stress-induced morphogen)-related protein |
| 79.761 | 5.2 | 3 | 1 | 4 | 1.133 | 1.247 | 1.11 | 0.816 | 0.882 | 0.912 | nucleus | K11721 | BRD3; bromodomain-containing protein 3 | | PF00439 | Bromodomain | K | KOG1474 | Transcription initiation factor TFIID, subunit BDF1 and related bromodomain proteins |
| 37.505 | 20.2 | 6 | 6 | 11 | 0.933 | 1.206 | 1.193 | 0.746 | 0.776 | 0.714 | plasma membrane | K01672 | CA; carbonic anhydrase [EC:4.2.1.1] | map00910 Nitrogen metabolism; map01100 Metabolic pathways | PF00194 | Eukaryotic-type carbonic anhydrase | P | KOG0382 | Carbonic anhydrase |
| 36.654 | 2 | 1 | 1 | 1 | 0.63 | 0.594 | 0.598 | 1.281 | 0.883 | 1.165 | extracellular |  |  |  |  |  |  |  |  |
| 81.858 | 9.5 | 5 | 5 | 5 | 0.852 | 0.873 | 0.913 | 1.208 | 1.306 | 0.971 | plasma membrane | K05675 | ABCD1, ALD; ATP-binding cassette, subfamily D (ALD), member 1 | map02010 ABC transporters; map04146 Peroxisome | PF00005 | ABC transporter | I | KOG0064 | Peroxisomal long-chain acyl-CoA transporter, ABC superfamily |
| 147.94 | 0.6 | 1 | 1 | 1 | 0.543 | 0.375 | 0.351 | 1.495 | 0.859 | 1.819 | plasma membrane | K16727 | ARHGEF10; Rho guanine nucleotide exchange factor 10 | | PF00621 | RhoGEF domain | T | KOG3522 | Predicted guanine nucleotide exchange factor |
| 7.3384 | 64.7 | 3 | 3 | 11 | 0.802 | 0.812 | 0.877 | 1.113 | 1.191 | 1.07 | extracellular | K07213 | ATOX1, ATX1, copZ, golB; copper chaperone | map04978 Mineral absorption | PF00403 | Heavy-metal-associated domain | C | KOG1603 | Copper chaperone |
| 42.761 | 8.9 | 3 | 2 | 3 | 1.18 | 1.214 | 1.212 | 0.88 | 0.783 | 0.893 | nucleus | K02211 | CDK9; cyclin-dependent kinase 9 [EC:2.7.11.22 2.7.11.23] | map05202 Transcriptional misregulation in cancer | PF00069 | Protein kinase domain | D | KOG0669 | Cyclin T-dependent kinase CDK9 |
| 45.974 | 29.8 | 11 | 2 | 47 | 0.793 | 0.986 | 0.808 | 1.324 | 1.013 | 1.276 | plasma membrane | K03984 | SERPINA1, AAT; alpha-1-antitrypsin | map04610 Complement and coagulation cascades | PF00079 | Serpin (serine protease inhibitor) | V | KOG2392 | Serpin |
| 35.752 | 53.3 | 15 | 15 | 38 | 0.811 | 0.872 | 0.76 | 1.276 | 1.433 | 1.073 | cytoplasm | K16646 | ANXA5; annexin A5 | |  |  | U | KOG0819 | Annexin |
| 47.597 | 43.5 | 14 | 14 | 25 | 1.346 | 1.288 | 1.494 | 0.733 | 0.734 | 0.948 | mitochondria | K00825 | AADAT, KAT2; kynurenine/2-aminoadipate aminotransferase [EC:2.6.1.7 2.6.1.39] | map00300 Lysine biosynthesis; map00310 Lysine degradation; map00380 Tryptophan metabolism; map01100 Metabolic pathways; map01210 2-Oxocarboxylic acid metabolism | PF00155 | Aminotransferase class I and II | E | KOG0634 | Aromatic amino acid aminotransferase and related proteins |
| 37.516 | 4.6 | 2 | 2 | 3 | 0.48 | 0.344 | 0.279 | 1.67 | 0.78 | 1.197 | cytoplasm | K15442 | TAD3, ADAT3; tRNA-specific adenosine deaminase 3 | | | | A | KOG2771 | Subunit of tRNA-specific adenosine-34 deaminase |
| 28.793 | 13.8 | 3 | 3 | 5 | 1.014 | 1.122 | 1.156 | 0.818 | 0.865 | 0.686 | plasma membrane | K09864 | AQP1; aquaporin-1 | map04924 Renin secretion; map04964 Proximal tubule bicarbonate reclamation; map04976 Bile secretion | | | G | KOG0223 | Aquaporin (major intrinsic protein family) |
| 11.424 | 19.4 | 2 | 2 | 5 | 1.22 | 1.268 | 1.43 | 0.847 | 0.923 | 0.789 | cytoplasm | K02140 | ATPeFG, ATP5L, ATP20; F-type H+-transporting ATPase subunit g | map00190 Oxidative phosphorylation; map01100 Metabolic pathways; map04714 Thermogenesis | | | C | KOG4103 | Mitochondrial F1F0-ATP synthase, subunit g/ATP20 |
| 38.86 | 11.1 | 4 | 4 | 4 | 0.849 | 0.844 | 0.864 | 1.544 | 1.367 | 1.131 | nucleus | K13698 | ABHD4; abhydrolase domain-containing protein 4 | | PF00561 | alpha/beta hydrolase fold | S | KOG4409 | Predicted hydrolase/acyltransferase (alpha/beta hydrolase superfamily) |
| 35.286 | 50.6 | 10 | 10 | 24 | 1.162 | 1.166 | 1.141 | 0.812 | 0.922 | 0.851 | extracellular | K18458 | ACY3; N-acyl-aromatic-L-amino acid amidohydrolase [EC:3.5.1.114] | | PF04952 | Succinylglutamate desuccinylase / Aspartoacylase family | | | |
| 167.32 | 4.1 | 5 | 5 | 6 | 0.919 | 0.695 | 0.675 | 1.185 | 1.031 | 0.965 | extracellular | K06237 | COL4A; collagen, type IV, alpha | map04151 PI3K-Akt signaling pathway; map04510 Focal adhesion; map04512 ECM-receptor interaction; map04926 Relaxin signaling pathway; map04933 AGE-RAGE signaling pathway in diabetic complications; map04974 Protein digestion and absorption; map05146 Amoebiasis; map05165 Human papillomavirus infection; map05200 Pathways in cancer; map05222 Small cell lung cancer | PF01413 | C-terminal tandem repeated domain in type 4 procollagen | W | KOG3544 | Collagens (type IV and type XIII), and related proteins |
| 27.013 | 15.8 | 3 | 3 | 5 | 0.831 | 0.817 | 0.871 | 0.996 | 1.444 | 1.098 | cytoplasm | K05021 | CLIC1; chloride intracellular channel protein 1 | | PF13409; PF13410 | Glutathione S-transferase, N-terminal domain; Glutathione S-transferase, C-terminal domain | P | KOG1422 | Intracellular Cl- channel CLIC, contains GST domain |
| 47.874 | 37 | 14 | 14 | 28 | 1.232 | 1.09 | 1.184 | 0.83 | 0.849 | 0.928 | mitochondria | K09478 | ACADSB; short/branched chain acyl-CoA dehydrogenase [EC:1.3.99.12] | map00071 Fatty acid degradation; map00280 Valine, leucine and isoleucine degradation; map01100 Metabolic pathways; map01212 Fatty acid metabolism | PF02771; PF02770; PF00441 | Acyl-CoA dehydrogenase, N-terminal domain; Acyl-CoA dehydrogenase, middle domain; Acyl-CoA dehydrogenase, C-terminal domain | I | KOG0139 | Short-chain acyl-CoA dehydrogenase |
| 28.211 | 43.1 | 12 | 8 | 42 | 0.876 | 0.843 | 0.845 | 1.15 | 1.226 | 1.095 | cytoplasm | K16198 | YWHAG_H; 14-3-3 protein gamma/eta | map04110 Cell cycle; map04114 Oocyte meiosis; map04151 PI3K-Akt signaling pathway; map04390 Hippo signaling pathway; map05160 Hepatitis C; map05203 Viral carcinogenesis | PF00244 | 14-3-3 protein | O | KOG0841 | Multifunctional chaperone (14-3-3 family) |
| 45.02 | 79.9 | 32 | 28 | 229 | 1.229 | 1.402 | 1.271 | 0.725 | 0.727 | 0.858 | cytoplasm | K00544 | BHMT; betaine-homocysteine S-methyltransferase [EC:2.1.1.5] | map00260 Glycine, serine and threonine metabolism; map00270 Cysteine and methionine metabolism; map01100 Metabolic pathways | | | E | KOG1579 | Homocysteine S-methyltransferase |
| 56.743 | 21 | 10 | 6 | 18 | 0.322 | 0.328 | 0.415 | 2.732 | 2.498 | 1.26 | endoplasmic reticulum | K07412 | CYP2B; cytochrome P450 family 2 subfamily B [EC:1.14.14.1] | map00140 Steroid hormone biosynthesis; map00590 Arachidonic acid metabolism; map00830 Retinol metabolism; map01100 Metabolic pathways; map05204 Chemical carcinogenesis | PF00067 | Cytochrome P450 | Q | KOG0156 | Cytochrome P450 CYP2 subfamily |
| 54.467 | 59.5 | 31 | 17 | 243 | 0.795 | 0.742 | 0.7 | 1.471 | 1.369 | 1.288 | cytoplasm | K07249 | ALDH1A; retinal dehydrogenase [EC:1.2.1.36] | map00830 Retinol metabolism; map01100 Metabolic pathways | | | C | KOG2450 | Aldehyde dehydrogenase |
| 49.925 | 28.5 | 12 | 12 | 31 | 0.827 | 0.877 | 0.786 | 1.328 | 1.208 | 1.174 | cytoplasm,nucleus | K17095 | ANXA7_11; annexin A7/11 | | |  | U | KOG0819 | Annexin |
| 59.646 | 9.7 | 5 | 5 | 6 | 1.142 | 1.468 | 1.35 | 0.822 | 0.803 | 0.976 | extracellular | K01135 | ARSB; arylsulfatase B [EC:3.1.6.12] | map00531 Glycosaminoglycan degradation; map01100 Metabolic pathways; map04142 Lysosome | | | P | KOG3867 | Sulfatase |
| 46.135 | 31.3 | 11 | 4 | 28 | 0.725 | 0.389 | 0.51 | 2.276 | 1.268 | 1.182 | cytoplasm | K01068 | ACOT1_2_4; acyl-coenzyme A thioesterase 1/2/4 [EC:3.1.2.2] | map00062 Fatty acid elongation; map01040 Biosynthesis of unsaturated fatty acids; map01100 Metabolic pathways | PF08840 | BAAT / Acyl-CoA thioester hydrolase C terminal | | | |
| 35.44 | 35.8 | 8 | 8 | 26 | 1.032 | 1.128 | 1.154 | 0.905 | 0.82 | 0.816 | mitochondria | K00020 | mmsB, HIBADH; 3-hydroxyisobutyrate dehydrogenase [EC:1.1.1.31] | map00280 Valine, leucine and isoleucine degradation; map01100 Metabolic pathways | PF03446 | NAD binding domain of 6-phosphogluconate dehydrogenase | I | KOG0409 | Predicted dehydrogenase |
| 8.2355 | 32.4 | 2 | 2 | 6 | 1.424 | 1.526 | 1.373 | 0.641 | 0.674 | 0.647 | cytoplasm | K02129 | ATPeF0E, ATP5I; F-type H+-transporting ATPase subunit e | map00190 Oxidative phosphorylation; map01100 Metabolic pathways; map04714 Thermogenesis | | | C | KOG4326 | Mitochondrial F1F0-ATP synthase, subunit e |
| 76.862 | 36.1 | 22 | 22 | 40 | 0.75 | 0.925 | 0.887 | 1.365 | 1.163 | 1.25 | cytoplasm | K10214 | ACOX2; 3alpha,7alpha,12alpha-trihydroxy-5beta-cholestanoyl-CoA 24-hydroxylase [EC:1.17.99.3] | map00120 Primary bile acid biosynthesis; map01100 Metabolic pathways; map03320 PPAR signaling pathway; map04146 Peroxisome | PF02770 | Acyl-CoA dehydrogenase, middle domain | I | KOG0136 | Acyl-CoA oxidase |
| 12.552 | 17.9 | 2 | 2 | 2 | 1.801 | 1.265 | 1.658 | 0.506 | 0.646 | 0.643 | extracellular | K18171 | CMC1; COX assembly mitochondrial protein 1 | | | | S | KOG4624 | Uncharacterized conserved protein |
| 35.977 | 11.4 | 3 | 3 | 5 | 1.329 | 1.056 | 1.4 | 0.893 | 0.744 | 0.756 | extracellular | K23344 | DDRGK1; DDRGK domain-containing protein 1 | | | | S | KOG3054 | Uncharacterized conserved protein |
| 30.641 | 70.4 | 18 | 17 | 37 | 0.74 | 0.64 | 0.576 | 1.471 | 1.497 | 1.301 | mitochondria | K00079 | CBR1; carbonyl reductase 1 [EC:1.1.1.184 1.1.1.189 1.1.1.197] | map00590 Arachidonic acid metabolism; map00790 Folate biosynthesis; map00980 Metabolism of xenobiotics by cytochrome P450; map01100 Metabolic pathways; map05204 Chemical carcinogenesis | PF00106 | short chain dehydrogenase | Q | KOG1208 | Dehydrogenases with different specificities (related to short-chain alcohol dehydrogenases) |
| 7.7662 | 34.3 | 2 | 2 | 5 | 1.26 | 1.158 | 1.054 | 0.63 | 0.864 | 0.792 | extracellular | K02125 | ATPeF08, MTATP8, ATP8; F-type H+-transporting ATPase subunit 8 | map00190 Oxidative phosphorylation; map01100 Metabolic pathways; map04714 Thermogenesis; map05010 Alzheimer disease;  map05012 Parkinson disease; map05016 Huntington disease | | | | | |
| 43.095 | 2 | 1 | 1 | 2 | 0.63 | 0.981 | 0.763 | 1.335 | 1.048 | 1.215 | nucleus |  |  |  | PF00786 | P21-Rho-binding domain | | |  |
| 74.648 | 52 | 27 | 27 | 131 | 0.815 | 0.986 | 0.825 | 1.392 | 1.118 | 1.276 | cytoplasm | K00232 | E1.3.3.6, ACOX1, ACOX3; acyl-CoA oxidase [EC:1.3.3.6] | map00071 Fatty acid degradation; map00410 beta-Alanine metabolism; map00592 alpha-Linolenic acid metabolism; map00640 Propanoate metabolism; map01040 Biosynthesis of unsaturated fatty acids; map01100 Metabolic pathways; map01200 Carbon metabolism; map01212 Fatty acid metabolism; map03320 PPAR signaling pathway; map04024 cAMP signaling pathway; map04146 Peroxisome | PF02770 | Acyl-CoA dehydrogenase, middle domain | I | KOG0136 | Acyl-CoA oxidase |
| 53.247 | 38.3 | 16 | 16 | 34 | 0.871 | 0.887 | 0.76 | 1.146 | 1.281 | 1.212 | cytoplasm | K00033 | PGD, gnd, gntZ; 6-phosphogluconate dehydrogenase [EC:1.1.1.44 1.1.1.343] | map00030 Pentose phosphate pathway; map00480 Glutathione metabolism; map01100 Metabolic pathways; map01200 Carbon metabolism | PF03446; PF00393 | NAD binding domain of 6-phosphogluconate dehydrogenase; 6-phosphogluconate dehydrogenase, C-terminal domain | G | KOG2653 | 6-phosphogluconate dehydrogenase |
| 237.79 | 0.6 | 1 | 1 | 1 | 0.851 | 0.866 | 0.758 | 0.984 | 1.213 | 1.037 | plasma membrane | K11653 | ARID1; AT-rich interactive domain-containing protein 1 | map04714 Thermogenesis; map05225 Hepatocellular carcinoma | PF01388 | ARID/BRIGHT DNA binding domain | K | KOG2510 | SWI-SNF chromatin-remodeling complex protein |
| 49.094 | 8.1 | 3 | 3 | 4 | 0.752 | 0.747 | 0.656 | 1.07 | 1.076 | 1.278 | nucleus | K09558 | BAG4; BCL2-associated athanogene 4 | map04668 TNF signaling pathway | | | T | KOG4361 | BCL2-associated athanogene-like proteins and related BAG family chaperone regulators |
| 25.013 | 23.9 | 6 | 4 | 17 | 1.263 | 1.202 | 1.106 | 0.893 | 0.983 | 0.768 | plasma membrane | K20838 | NAT8; N-acetyltransferase 8 [EC:2.3.1.80 2.3.1.-] | map00480 Glutathione metabolism; map01100 Metabolic pathways | | | S | KOG3139 | N-acetyltransferase |
| 55.715 | 41.4 | 21 | 12 | 98 | 0.696 | 0.838 | 0.755 | 1.512 | 1.596 | 0.993 | endoplasmic reticulum | K07413 | CYP2C; cytochrome P450 family 2 subfamily C [EC:1.14.14.1] | map00140 Steroid hormone biosynthesis; map00590 Arachidonic acid metabolism; map00591 Linoleic acid metabolism; map00830 Retinol metabolism; map01100 Metabolic pathways; map04726 Serotonergic synapse; map04750 Inflammatory mediator regulation of TRP channels; map05204 Chemical carcinogenesis | PF00067 | Cytochrome P450 | Q | KOG0156 | Cytochrome P450 CYP2 subfamily |
| 35.827 | 24.7 | 7 | 7 | 8 | 0.64 | 0.744 | 0.477 | 1.869 | 1.491 | 1.63 | cytoplasm | K11992 | ACOT8, PTE; acyl-CoA thioesterase 8 [EC:3.1.2.27] | map00120 Primary bile acid biosynthesis; map01100 Metabolic pathways; map04146 Peroxisome | PF13622 | Thioesterase-like superfamily | I | KOG3016 | Acyl-CoA thioesterase |
| 19.152 | 11 | 3 | 3 | 3 | 0.691 | 0.667 | 0.405 | 1.406 | 1.16 | 0.87 | plasma membrane | K06731 | BST2, CD317; bone marrow stromal cell antigen 2 | map05168 Herpes simplex virus 1 infection; map05170 Human immunodeficiency virus 1 infection | | | | | |
| 29.366 | 80.4 | 18 | 18 | 249 | 1.207 | 1.314 | 1.124 | 0.772 | 0.734 | 0.689 | cytoplasm | K01672 | CA; carbonic anhydrase [EC:4.2.1.1] | map00910 Nitrogen metabolism; map01100 Metabolic pathways | PF00194 | Eukaryotic-type carbonic anhydrase | P | KOG0382 | Carbonic anhydrase |
| 36.79 | 11.1 | 3 | 3 | 5 | 1.16 | 1.248 | 1.112 | 0.874 | 1.042 | 0.788 | extracellular | K00729 | ALG5; dolichyl-phosphate beta-glucosyltransferase [EC:2.4.1.117] | map00510 N-Glycan biosynthesis; map01100 Metabolic pathways | | | M | KOG2977 | Glycosyltransferase |
| 44.669 | 14.7 | 7 | 7 | 12 | 0.773 | 0.797 | 0.884 | 1.1 | 1.506 | 1.271 | extracellular | K12348 | ASAH1; acid ceramidase [EC:3.5.1.23] | map00600 Sphingolipid metabolism; map01100 Metabolic pathways; map04071 Sphingolipid signaling pathway; map04142 Lysosome | PF02275 | Linear amide C-N hydrolases, choloylglycine hydrolase family | | | |
| 186.41 | 1.2 | 1 | 1 | 5 | 1.307 | 1.149 | 1.272 | 0.746 | 0.744 | 0.677 | plasma membrane | K11265 | ADCY10; adenylate cyclase 10 [EC:4.6.1.1] | map00230 Purine metabolism; map01100 Metabolic pathways; map04024 cAMP signaling pathway; map04371 Apelin signaling pathway; map04713 Circadian entrainment; map04714 Thermogenesis; map04935 Growth hormone synthesis, secretion and action | PF00211 | Adenylate and Guanylate cyclase catalytic domain | | | |
| 25.422 | 14.9 | 2 | 2 | 3 | 1.086 | 0.99 | 0.987 | 0.88 | 0.771 | 0.694 | extracellular | K06722 | CD302; CD302 antigen | | PF00059 | Lectin C-type domain | T | KOG4297 | C-type lectin |
| 13.274 | 9.3 | 1 | 1 | 1 | 0.81 | 0.894 | 0.874 | 1.313 | 1.369 | 0.976 | cytoplasm | K08504 | BET1; blocked early in transport 1 | map04130 SNARE interactions in vesicular transport | | | U | KOG3385 | V-SNARE |
| 55.948 | 1.8 | 1 | 1 | 2 | 0.496 | 0.404 | 0.547 | 1.149 | 0.927 | 1.161 | nucleus | K07767 | KATNA1; katanin p60 ATPase-containing subunit A1 [EC:5.6.1.1] | | PF00004; PF09336 | ATPase family associated with various cellular activities (AAA); Vps4 C terminal oligomerisation domain | O | KOG0738 | AAA+-type ATPase |
| 62.644 | 1.6 | 1 | 1 | 1 | 0.982 | 1.177 | 1.268 | 0.741 | 0.768 | 0.903 | plasma membrane | K08228 | SLC43A1; MFS transporter, LAT3 family, solute carrier family 43, member 1 | | | | | | |
| 34.155 | 38.9 | 11 | 11 | 18 | 1.127 | 1.122 | 1.151 | 0.811 | 0.86 | 0.74 | cytoplasm | K15104 | SLC25A11, OGC; solute carrier family 25 (mitochondrial oxoglutarate transporter), member 11 | | | | C | KOG0759 | Mitochondrial oxoglutarate/malate carrier proteins |
| 21.71 | 24.9 | 5 | 5 | 10 | 1.29 | 1.232 | 1.274 | 0.763 | 0.791 | 0.762 | mitochondria | K03961 | NDUFB5; NADH dehydrogenase (ubiquinone) 1 beta subcomplex subunit 5 | map00190 Oxidative phosphorylation; map01100 Metabolic pathways; map04714 Thermogenesis; map04723 Retrograde endocannabinoid signaling;  map04932 Non-alcoholic fatty liver disease (NAFLD); map05010 Alzheimer disease; map05012 Parkinson disease; map05016 Huntington disease | | | C | KOG4632 | NADH:ubiquinone oxidoreductase, NDUFB5/SGDH subunit |
| 45.345 | 9.7 | 3 | 2 | 8 | 0.704 | 0.766 | 0.657 | 1.359 | 1.473 | 1.234 | cytoplasm,nucleus | K11279 | NAP1L1, NRP; nucleosome assembly protein 1-like 1 | | | | BD | KOG1507 | Nucleosome assembly protein NAP-1 |
| 284.08 | 0.6 | 2 | 2 | 2 | 0.631 | 0.556 | 0.555 | 1.201 | 1.14 | 1.115 | nucleus | K15588 | NSD1; [histone H3]-lysine36 N-dimethyltransferase NSD1 [EC:2.1.1.357] | map00310 Lysine degradation; map01100 Metabolic pathways | PF00855 | PWWP domain | K | KOG1081 | Transcription factor NSD1 and related SET domain proteins |
| 79.957 | 1.4 | 1 | 1 | 1 | 1.077 | 1.69 | 1.288 | 0.759 | 0.428 | 0.689 | mitochondria | |  |  | PF13923 | Zinc finger, C3HC4 type (RING finger) | | | |
| 44.953 | 31.7 | 10 | 10 | 30 | 0.843 | 0.845 | 0.777 | 1.076 | 1.386 | 1.13 | extracellular | K01379 | CTSD; cathepsin D [EC:3.4.23.5] | map04071 Sphingolipid signaling pathway; map04140 Autophagy - animal; map04142 Lysosome; map04210 Apoptosis;  map04915 Estrogen signaling pathway; map05152 Tuberculosis | | | O | KOG1339 | Aspartyl protease |
| 32.928 | 28 | 5 | 5 | 10 | 0.781 | 0.735 | 0.87 | 1.409 | 1.366 | 1.249 | cytoplasm | K00510 | HMOX1; heme oxygenase 1 [EC:1.14.14.18] | map00860 Porphyrin and chlorophyll metabolism; map01100 Metabolic pathways; map04066 HIF-1 signaling pathway; map04216 Ferroptosis; map04978 Mineral absorption; map05200 Pathways in cancer; map05206 MicroRNAs in cancer; map05225 Hepatocellular carcinoma; map05418 Fluid shear stress and atherosclerosis | PF01126 | Heme oxygenase | P | KOG4480 | Heme oxygenase |
| 11.934 | 43.9 | 4 | 4 | 17 | 0.786 | 0.777 | 0.719 | 1.474 | 1.339 | 1.22 | extracellular | K06554 | IGLL1, IGLL, CD179b; immunoglobulin lambda-like polypeptide 1 | map05340 Primary immunodeficiency | PF07654 | Immunoglobulin C1-set domain | | |  |
| 43.929 | 6 | 2 | 2 | 2 | 0.823 | 0.862 | 0.701 | 1.626 | 1.419 | 1.131 | plasma membrane | K06856 | IGH; immunoglobulin heavy chain | map04020 Calcium signaling pathway; map04064 NF-kappa B signaling pathway; map04072 Phospholipase D signaling pathway; map04145 Phagosome; map04151 PI3K-Akt signaling pathway; map04640 Hematopoietic cell lineage; map04650 Natural killer cell mediated cytotoxicity; map04662 B cell receptor signaling pathway; map04664 Fc epsilon RI signaling pathway; map04666 Fc gamma R-mediated phagocytosis; map04672 Intestinal immune network for IgA production; map05130 Pathogenic Escherichia coli infection; map05140 Leishmaniasis; map05143 African trypanosomiasis; map05146 Amoebiasis; map05150 Staphylococcus aureus infection; map05152 Tuberculosis; map05169 Epstein-Barr virus infection; map05202 Transcriptional misregulation in cancer; map05310 Asthma; map05320 Autoimmune thyroid disease; map05322 Systemic lupus erythematosus; map05323 Rheumatoid arthritis; map05330 Allograft rejection; map05340 Primary immunodeficiency; map05414 Dilated cardiomyopathy (DCM); map05416 Viral myocarditis | PF07654 | Immunoglobulin C1-set domain | | |  |
| 71.991 | 1.7 | 1 | 1 | 1 | 2.151 | 1.661 | 2.027 | 0.507 | 0.748 | 0.37 | plasma membrane | K07523 | LRRC4C, NGL1; netrin-G1 ligand | map04360 Axon guidance; map04514 Cell adhesion molecules (CAMs) | PF07679 | Immunoglobulin I-set domain | T | KOG0619 | FOG: Leucine rich repeat |
| 21.536 | 28.4 | 7 | 7 | 8 | 0.979 | 0.802 | 0.798 | 1.238 | 1.224 | 1.045 | cytoplasm |  |  |  |  |  |  |  |  |
| 21.139 | 20.8 | 4 | 4 | 6 | 1.382 | 1.172 | 1.28 | 0.774 | 0.821 | 0.938 | nucleus |  |  |  |  |  | S | KOG4706 | Uncharacterized conserved protein |
| 52.697 | 17.2 | 7 | 7 | 11 | 0.648 | 0.68 | 0.622 | 1.438 | 1.406 | 1.083 | plasma membrane | K06259 | CD36; CD36 antigen | map03320 PPAR signaling pathway; map04145 Phagosome; map04152 AMPK signaling pathway; map04512 ECM-receptor interaction;  map04640 Hematopoietic cell lineage; map04920 Adipocytokine signaling pathway; map04931 Insulin resistance;  map04975 Fat digestion and absorption; map04979 Cholesterol metabolism; map05144 Malaria | | | T | KOG3776 | Plasma membrane glycoprotein CD36 and related membrane receptors |
| 201.82 | 4.5 | 7 | 7 | 7 | 0.742 | 0.8 | 0.884 | 1.013 | 1.052 | 1.26 | extracellular | K06241 | LAMA4; laminin, alpha 4 | map04151 PI3K-Akt signaling pathway; map04510 Focal adhesion; map04512 ECM-receptor interaction; map05143 African trypanosomiasis; map05145 Toxoplasmosis; map05146 Amoebiasis; map05165 Human papillomavirus infection; map05200 Pathways in cancer; map05222 Small cell lung cancer | PF00053; PF02210 | Laminin EGF-like (Domains III and V); Laminin G domain | W | KOG1836 | Extracellular matrix glycoprotein Laminin subunits alpha and gamma |
| 114.65 | 18.5 | 16 | 16 | 20 | 1.182 | 1.152 | 1.284 | 0.896 | 0.844 | 0.796 | nucleus | K12311 | MAN2B1, LAMAN; lysosomal alpha-mannosidase [EC:3.2.1.24] | map00511 Other glycan degradation; map04142 Lysosome | PF01074; PF09261; PF07748 | Glycosyl hydrolases family 38 N-terminal domain; Alpha mannosidase, middle domain; Glycosyl hydrolases family 38 C-terminal domain | G | KOG1959 | Glycosyl hydrolase, family 38 - alpha-mannosidase |
| 54.317 | 4.2 | 2 | 2 | 2 | 0.752 | 0.608 | 0.602 | 1.356 | 2.098 | 1.062 | cytoplasm | K08849 | MLKL; mixed lineage kinase domain-like [EC:2.7.11.1] | map04217 Necroptosis; map04668 TNF signaling pathway | PF07714 | Protein tyrosine kinase | T | KOG0192 | Tyrosine kinase specific for activated (GTP-bound) p21cdc42Hs |
| 53.394 | 7.2 | 3 | 1 | 4 | 1.058 | 1.284 | 1.333 | 0.932 | 0.741 | 0.792 | nucleus | K09171 | NFIX; nuclear factor I/X | | PF03165 | MH1 domain | K | KOG3663 | Nuclear factor I |
| 79.076 | 8.7 | 5 | 5 | 7 | 0.846 | 0.941 | 0.773 | 1.31 | 1.255 | 1.303 | endoplasmic reticulum | K01897 | ACSL, fadD; long-chain acyl-CoA synthetase [EC:6.2.1.3] | map00061 Fatty acid biosynthesis; map00071 Fatty acid degradation; map01100 Metabolic pathways; map01212 Fatty acid metabolism;  map03320 PPAR signaling pathway; map04146 Peroxisome; map04216 Ferroptosis; map04714 Thermogenesis;  map04920 Adipocytokine signaling pathway | | | I | KOG1180 | Acyl-CoA synthetase |
| 46.975 | 19.5 | 8 | 8 | 9 | 1.119 | 1.254 | 1.231 | 0.836 | 0.87 | 0.766 | cytoplasm | K09881 | AGK; acylglycerol kinase [EC:2.7.1.94] | map00561 Glycerolipid metabolism; map01100 Metabolic pathways | | | IT | KOG4435 | Predicted lipid kinase |
| 39.355 | 64.3 | 23 | 17 | 49 | 0.802 | 0.833 | 0.835 | 1.194 | 1.319 | 1.269 | cytoplasm | K01623 | ALDO; fructose-bisphosphate aldolase, class I [EC:4.1.2.13] | map00010 Glycolysis / Gluconeogenesis; map00030 Pentose phosphate pathway; map00051 Fructose and mannose metabolism; map01100 Metabolic pathways; map01200 Carbon metabolism; map01230 Biosynthesis of amino acids; map04066 HIF-1 signaling pathway | PF00274 | Fructose-bisphosphate aldolase class-I | G | KOG1557 | Fructose-biphosphate aldolase |
| 16.98 | 37.3 | 5 | 5 | 6 | 0.86 | 0.756 | 0.724 | 1.04 | 1.055 | 1.154 | nucleus | K05754 | ARPC5; actin related protein 2/3 complex, subunit 5 | map04144 Endocytosis; map04666 Fc gamma R-mediated phagocytosis; map04810 Regulation of actin cytoskeleton; map05100 Bacterial invasion of epithelial cells; map05130 Pathogenic Escherichia coli infection; map05131 Shigellosis; map05132 Salmonella infection | PF04699 | ARP2/3 complex 16 kDa subunit (p16-Arc) | Z | KOG3380 | Actin-related protein Arp2/3 complex, subunit ARPC5 |
| 191.19 | 0.8 | 1 | 1 | 1 | 0.71 | 0.656 | 0.768 | 1.032 | 1.304 | 0.894 | nucleus |  |  |  |  |  | K | KOG4377 | Zn-finger protein |
| 82.367 | 10.6 | 7 | 7 | 7 | 0.851 | 0.802 | 0.844 | 1.177 | 1.242 | 0.949 | cytoplasm |  |  |  |  |  | U | KOG4677 | Golgi integral membrane protein |
| 15.792 | 16.8 | 3 | 3 | 3 | 1.215 | 1.256 | 1.238 | 0.923 | 0.604 | 0.771 | extracellular |  |  |  | PF10256 | Golgin subfamily A member 7/ERF4 family | S | KOG4069 | Uncharacterized conserved protein |
| 53.076 | 13.6 | 6 | 6 | 7 | 1.272 | 1.253 | 1.125 | 1.003 | 0.833 | 0.904 | mitochondria | |  |  | PF00076 | RNA recognition motif. (a.k.a. RRM, RBD, or RNP domain) | A | KOG4211 | Splicing factor hnRNP-F and related RNA-binding proteins |
| 48.974 | 2 | 1 | 1 | 1 | 1.069 | 1.175 | 1.229 | 0.922 | 0.828 | 0.761 | plasma membrane | |  |  |  |  | S | KOG4290 | Predicted membrane protein |
| 57.568 | 24.6 | 10 | 9 | 15 | 0.868 | 0.867 | 0.857 | 1.305 | 1.22 | 1.168 | cytoplasm | K01641 | E2.3.3.10; hydroxymethylglutaryl-CoA synthase [EC:2.3.3.10] | map00072 Synthesis and degradation of ketone bodies; map00280 Valine, leucine and isoleucine degradation; map00650 Butanoate metabolism; map00900 Terpenoid backbone biosynthesis; map01100 Metabolic pathways; map03320 PPAR signaling pathway | PF01154; PF08540 | Hydroxymethylglutaryl-coenzyme A synthase N terminal; Hydroxymethylglutaryl-coenzyme A synthase C terminal | I | KOG1393 | Hydroxymethylglutaryl-CoA synthase |
| 20.082 | 20.8 | 4 | 4 | 6 | 1.322 | 1.357 | 1.361 | 0.905 | 0.882 | 0.771 | mitochondria | K18161 | NDUFAF4; NADH dehydrogenase [ubiquinone] 1 alpha subcomplex assembly factor 4 | map04714 Thermogenesis | | | S | KOG4481 | Uncharacterized conserved protein |
| 21.024 | 64.2 | 10 | 10 | 22 | 1.318 | 1.302 | 1.256 | 0.753 | 0.812 | 0.812 | cytoplasm | K03966 | NDUFB10; NADH dehydrogenase (ubiquinone) 1 beta subcomplex subunit 10 | map00190 Oxidative phosphorylation; map01100 Metabolic pathways; map04714 Thermogenesis; map04723 Retrograde endocannabinoid signaling;  map04932 Non-alcoholic fatty liver disease (NAFLD); map05010 Alzheimer disease; map05012 Parkinson disease; map05016 Huntington disease | | | C | KOG4009 | NADH-ubiquinone oxidoreductase, subunit NDUFB10/PDSW |
| 16.555 | 7.9 | 1 | 1 | 1 | 1.214 | 1.336 | 1.403 | 0.809 | 0.801 | 0.694 | plasma membrane | |  |  |  |  |  |  |  |
| 55.948 | 35 | 17 | 17 | 65 | 1.456 | 1.054 | 1.146 | 0.73 | 0.846 | 0.655 | endoplasmic reticulum | K07416 | CYP2F; cytochrome P450 family 2 subfamily F [EC:1.14.14.1] | map00980 Metabolism of xenobiotics by cytochrome P450 | PF00067 | Cytochrome P450 | Q | KOG0156 | Cytochrome P450 CYP2 subfamily |
| 38.752 | 46.7 | 17 | 17 | 44 | 0.602 | 0.601 | 0.546 | 1.364 | 1.3 | 0.849 | extracellular | K16142 | HP; haptoglobin | | PF00089 | Trypsin | E | KOG3627 | Trypsin |
| 26.87 | 36.4 | 9 | 9 | 14 | 0.527 | 0.597 | 0.499 | 1.994 | 1.875 | 1.427 | cytoplasm | K17290 | HTATIP2; oxidoreductase [EC:1.1.1.-] | | PF13460 | NADH(P)-binding | T | KOG4039 | Serine/threonine kinase TIP30/CC3 |
| 39.401 | 7.4 | 3 | 3 | 3 | 1.503 | 1.564 | 1.523 | 1.026 | 0.394 | 0.656 | extracellular | K22688 | INHBC; inhibin beta C chain | map04060 Cytokine-cytokine receptor interaction; map04350 TGF-beta signaling pathway; map04550 Signaling pathways regulating pluripotency of stem cells | PF00019 | Transforming growth factor beta like domain | T | KOG3900 | Transforming growth factor beta, bone morphogenetic protein and related proteins |
| 18.582 | 9.8 | 1 | 1 | 2 | 0.782 | 0.798 | 0.859 | 1.123 | 1.152 | 1.047 | cytoplasm | K17619 | MDP1; magnesium-dependent phosphatase 1 [EC:3.1.3.48 3.1.3.-] | | | | S | KOG4549 | Magnesium-dependent phosphatase |
| 41.565 | 9.5 | 5 | 5 | 8 | 1.266 | 1.376 | 1.271 | 0.862 | 0.64 | 0.759 | plasma membrane | K17885 | MTCH; mitochondrial carrier | | |  | C | KOG2745 | Mitochondrial carrier protein |
| 66.235 | 1.8 | 1 | 1 | 1 | 0.777 | 0.727 | 0.667 | 1.201 | 1.143 | 1.143 | plasma membrane | K08183 | SLC16A6; MFS transporter, MCT family, solute carrier family 16 (monocarboxylic acid transporters), member 6 | | | | G | KOG2504 | Monocarboxylate transporter |
| 22.228 | 52.3 | 10 | 10 | 19 | 0.878 | 0.939 | 0.811 | 1.265 | 1.234 | 1.182 | extracellular | K05361 | GPX4; phospholipid-hydroperoxide glutathione peroxidase [EC:1.11.1.12] | map00480 Glutathione metabolism; map01100 Metabolic pathways; map04216 Ferroptosis | | | O | KOG1651 | Glutathione peroxidase |
| 11.871 | 43.9 | 4 | 4 | 10 | 0.74 | 0.688 | 0.889 | 1.064 | 1.115 | 1.219 | cytoplasm | K03676 | grxC, GLRX, GLRX2; glutaredoxin 3 | | PF00462 | Glutaredoxin | O | KOG1752 | Glutaredoxin and related proteins |
| 38.018 | 18.1 | 7 | 7 | 9 | 0.86 | 0.796 | 0.914 | 1.068 | 1.341 | 1.118 | cytoplasm | K00364 | E1.7.1.7, guaC; GMP reductase [EC:1.7.1.7] | map00230 Purine metabolism; map01100 Metabolic pathways | PF00478 | IMP dehydrogenase / GMP reductase domain | F | KOG2550 | IMP dehydrogenase/GMP reductase |
| 46.402 | 35.9 | 19 | 7 | 48 | 0.748 | 0.924 | 0.905 | 1.142 | 1.168 | 1.119 | nucleus | K03257 | EIF4A; translation initiation factor 4A | map03013 RNA transport | PF00270 | DEAD/DEAH box helicase | J | KOG0327 | Translation initiation factor 4F, helicase subunit (eIF-4A) and related helicases |
| 54.531 | 43 | 18 | 18 | 45 | 1.238 | 1.296 | 1.244 | 0.682 | 0.814 | 0.761 | endoplasmic reticulum | K00486 | KMO; kynurenine 3-monooxygenase [EC:1.14.13.9] | map00380 Tryptophan metabolism; map01100 Metabolic pathways | | | C | KOG2614 | Kynurenine 3-monooxygenase and related flavoprotein monooxygenases |
| 58.408 | 52.5 | 24 | 24 | 72 | 1.138 | 1.162 | 1.148 | 0.886 | 0.832 | 0.913 | mitochondria | K01966 | PCCB, pccB; propionyl-CoA carboxylase beta chain [EC:6.4.1.3 2.1.3.15] | map00280 Valine, leucine and isoleucine degradation; map00630 Glyoxylate and dicarboxylate metabolism; map00640 Propanoate metabolism;  map01100 Metabolic pathways; map01200 Carbon metabolism | | | EI | KOG0540 | 3-Methylcrotonyl-CoA carboxylase, non-biotin containing subunit/Acetyl-CoA carboxylase carboxyl transferase, subunit beta |
| 111.25 | 42.5 | 37 | 37 | 74 | 1.314 | 1.179 | 1.034 | 0.801 | 0.824 | 0.976 | cytoplasm | K00207 | DPYD; dihydropyrimidine dehydrogenase (NADP+) [EC:1.3.1.2] | map00240 Pyrimidine metabolism; map00410 beta-Alanine metabolism; map00770 Pantothenate and CoA biosynthesis;  map00983 Drug metabolism - other enzymes; map01100 Metabolic pathways | PF07992; PF01180 | Pyridine nucleotide-disulphide oxidoreductase; Dihydroorotate dehydrogenase | F | KOG1799 | Dihydropyrimidine dehydrogenase |
| 42.145 | 11.8 | 4 | 4 | 4 | 1.198 | 1.298 | 1.218 | 0.947 | 0.787 | 0.822 | cytoplasm | K10105 | LIPT1; lipoyltransferase 1 | map00785 Lipoic acid metabolism; map01100 Metabolic pathways | | | H | KOG3159 | Lipoate-protein ligase A |
| 169.12 | 11.4 | 15 | 15 | 21 | 0.846 | 0.732 | 0.799 | 1.287 | 1.245 | 0.934 | plasma membrane | K05667 | ABCC3; ATP-binding cassette, subfamily C (CFTR/MRP), member 3 | map01523 Antifolate resistance; map02010 ABC transporters; map04976 Bile secretion | PF00005 | ABC transporter | Q | KOG0054 | Multidrug resistance-associated protein/mitoxantrone resistance protein, ABC superfamily |
| 21.876 | 38.2 | 5 | 5 | 8 | 1.196 | 1.101 | 1.2 | 0.834 | 0.889 | 0.826 | mitochondria | K03964 | NDUFB8; NADH dehydrogenase (ubiquinone) 1 beta subcomplex subunit 8 | map00190 Oxidative phosphorylation; map01100 Metabolic pathways; map04714 Thermogenesis; map04723 Retrograde endocannabinoid signaling;  map04932 Non-alcoholic fatty liver disease (NAFLD); map05010 Alzheimer disease; map05012 Parkinson disease; map05016 Huntington disease | | | C | KOG4040 | NADH:ubiquinone oxidoreductase, NDUFB8/ASHI subunit |
| 41.989 | 11.6 | 4 | 4 | 4 | 0.924 | 0.8 | 0.769 | 1.103 | 1.015 | 1.185 | nucleus | K14305 | NUP43; nuclear pore complex protein Nup43 | map03013 RNA transport | | | Y | KOG4714 | Nucleoporin |
| 12.159 | 8.5 | 1 | 1 | 1 | 2.453 | 2.621 | 2.121 | 0.423 | 0.334 | 0.421 | mitochondria | K22255 | ATPIF1; ATPase inhibitor, mitochondrial | | | |  |  |  |
| 160.75 | 0.9 | 1 | 1 | 1 | 0.67 | 0.672 | 0.738 | 1.213 | 1.394 | 0.89 | nucleus | K16467 | CNTLN; centlein | |  |  |  |  |  |
| 64.958 | 4.8 | 3 | 3 | 4 | 0.856 | 0.87 | 0.787 | 1.156 | 0.971 | 1.156 | nucleus | K14405 | FIP1L1, FIP1; pre-mRNA 3'-end-processing factor FIP1 | map03015 mRNA surveillance pathway | | | A | KOG1049 | Polyadenylation factor I complex, subunit FIP1 |
| 27.014 | 19.5 | 5 | 5 | 7 | 0.768 | 0.966 | 0.922 | 1.245 | 1.127 | 1.32 | cytoplasm | K01495 | GCH1, folE; GTP cyclohydrolase IA [EC:3.5.4.16] | map00790 Folate biosynthesis; map01100 Metabolic pathways | PF01227 | GTP cyclohydrolase I | H | KOG2698 | GTP cyclohydrolase I |
| 35.704 | 24.7 | 5 | 5 | 11 | 0.877 | 0.99 | 0.758 | 1.286 | 1.08 | 1.343 | extracellular | K06856 | IGH; immunoglobulin heavy chain | map04020 Calcium signaling pathway; map04064 NF-kappa B signaling pathway; map04072 Phospholipase D signaling pathway; map04145 Phagosome; map04151 PI3K-Akt signaling pathway; map04640 Hematopoietic cell lineage; map04650 Natural killer cell mediated cytotoxicity; map04662 B cell receptor signaling pathway; map04664 Fc epsilon RI signaling pathway; map04666 Fc gamma R-mediated phagocytosis; map04672 Intestinal immune network for IgA production; map05130 Pathogenic Escherichia coli infection; map05140 Leishmaniasis; map05143 African trypanosomiasis; map05146 Amoebiasis; map05150 Staphylococcus aureus infection; map05152 Tuberculosis; map05169 Epstein-Barr virus infection; map05202 Transcriptional misregulation in cancer; map05310 Asthma; map05320 Autoimmune thyroid disease; map05322 Systemic lupus erythematosus; map05323 Rheumatoid arthritis; map05330 Allograft rejection; map05340 Primary immunodeficiency; map05414 Dilated cardiomyopathy (DCM); map05416 Viral myocarditis | PF07654 | Immunoglobulin C1-set domain | | |  |
| 20.648 | 66.1 | 14 | 1 | 112 | 2.022 | 1.464 | 1.553 | 0.158 | 0.206 | 0.574 | extracellular |  |  |  | PF00061 | Lipocalin / cytosolic fatty-acid binding protein family | | | |
| 51.51 | 28.8 | 13 | 13 | 25 | 0.827 | 0.852 | 0.745 | 1.412 | 1.191 | 1.292 | nucleus | K03426 | E3.6.1.22, NUDT12, nudC; NAD+ diphosphatase [EC:3.6.1.22] | map00760 Nicotinate and nicotinamide metabolism; map01100 Metabolic pathways; map04146 Peroxisome | PF09296; PF09297; PF00293 | NADH pyrophosphatase-like rudimentary NUDIX domain; NADH pyrophosphatase zinc ribbon domain; NUDIX domain | L | KOG3084 | NADH pyrophosphatase I of the Nudix family of hydrolases |
| 88.25 | 29.6 | 22 | 22 | 36 | 1.143 | 1.176 | 1.089 | 0.877 | 0.963 | 0.752 | plasma membrane | K08765 | CPT1A; carnitine O-palmitoyltransferase 1, liver isoform [EC:2.3.1.21] | map00071 Fatty acid degradation; map01212 Fatty acid metabolism; map03320 PPAR signaling pathway; map04152 AMPK signaling pathway;  map04714 Thermogenesis; map04920 Adipocytokine signaling pathway; map04922 Glucagon signaling pathway; map04931 Insulin resistance | | | I | KOG3716 | Carnitine O-acyltransferase CPTI |
| 52.683 | 11.2 | 5 | 5 | 6 | 1.312 | 1.438 | 1.057 | 0.655 | 0.878 | 0.879 | nucleus | K07292 | NR2A1, HNF4A; hepatocyte nuclear factor 4-alpha | map04152 AMPK signaling pathway; map04950 Maturity onset diabetes of the young | PF00105; PF00104 | Zinc finger, C4 type (two domains); Ligand-binding domain of nuclear hormone receptor | K | KOG4215 | Hepatocyte nuclear factor 4 and similar steroid hormone receptors |
| 186.53 | 5.1 | 6 | 5 | 7 | 0.374 | 0.378 | 0.447 | 1.439 | 1.312 | 1.352 | nucleus | K10395 | KIF4_21_27; kinesin family member 4/21/27 | | PF00225 | Kinesin motor domain | Z | KOG0244 | Kinesin-like protein |
| 77.902 | 2.1 | 2 | 2 | 3 | 0.669 | 0.73 | 0.742 | 1.157 | 1.051 | 0.965 | nucleus | K15589 | ARNT2; aryl hydrocarbon receptor nuclear translocator 2 | map05200 Pathways in cancer; map05202 Transcriptional misregulation in cancer; map05211 Renal cell carcinoma | PF00010; PF00989 | Helix-loop-helix DNA-binding domain; PAS fold | K | KOG3561 | Aryl-hydrocarbon receptor nuclear translocator |
| 20.153 | 34.8 | 6 | 6 | 6 | 0.854 | 0.943 | 0.706 | 1.238 | 1.073 | 1.453 | cytoplasm | K07232 | CHAC, chaC; glutathione-specific gamma-glutamylcyclotransferase [EC:4.3.2.7] | map00480 Glutathione metabolism; map01100 Metabolic pathways | | | P | KOG3182 | Predicted cation transporter |
| 52.145 | 4.2 | 2 | 2 | 2 | 1.468 | 1.275 | 1.574 | 0.862 | 0.411 | 0.712 | nucleus | K09048 | CREB3; cyclic AMP-responsive element-binding protein 3 | map04022 cGMP-PKG signaling pathway; map04024 cAMP signaling pathway; map04151 PI3K-Akt signaling pathway;  map04152 AMPK signaling pathway; map04211 Longevity regulating pathway; map04261 Adrenergic signaling in cardiomyocytes;  map04668 TNF signaling pathway; map04714 Thermogenesis; map04725 Cholinergic synapse; map04728 Dopaminergic synapse;  map04911 Insulin secretion; map04915 Estrogen signaling pathway; map04916 Melanogenesis; map04918 Thyroid hormone synthesis;  map04922 Glucagon signaling pathway; map04925 Aldosterone synthesis and secretion; map04926 Relaxin signaling pathway;  map04927 Cortisol synthesis and secretion; map04928 Parathyroid hormone synthesis, secretion and action; map04931 Insulin resistance;  map04934 Cushing syndrome; map04935 Growth hormone synthesis, secretion and action; map04962 Vasopressin-regulated water reabsorption;  map05016 Huntington disease; map05030 Cocaine addiction; map05031 Amphetamine addiction; map05034 Alcoholism; map05161 Hepatitis B;  map05163 Human cytomegalovirus infection; map05165 Human papillomavirus infection; map05166 Human T-cell leukemia virus 1 infection;  map05203 Viral carcinogenesis; map05215 Prostate cancer | | | K | KOG0709 | CREB/ATF family transcription factor |
| 31.458 | 15.4 | 4 | 4 | 7 | 0.771 | 0.734 | 0.705 | 1.237 | 1.082 | 1.323 | mitochondria | K07071 | K07071; uncharacterized protein | | |  | F | KOG3019 | Predicted nucleoside-diphosphate sugar epimerase |
| 33.387 | 35.6 | 8 | 8 | 22 | 0.734 | 0.756 | 0.791 | 1.503 | 1.378 | 1.116 | cytoplasm | K01054 | MGLL; acylglycerol lipase [EC:3.1.1.23] | map00561 Glycerolipid metabolism; map01100 Metabolic pathways; map04714 Thermogenesis; map04723 Retrograde endocannabinoid signaling;  map04923 Regulation of lipolysis in adipocytes | | | I | KOG1455 | Lysophospholipase |
| 39.764 | 47.2 | 17 | 17 | 113 | 1.164 | 1.263 | 1.203 | 0.785 | 0.808 | 0.877 | mitochondria | K00611 | OTC, argF, argI; ornithine carbamoyltransferase [EC:2.1.3.3] | map00220 Arginine biosynthesis; map01100 Metabolic pathways; map01230 Biosynthesis of amino acids | PF02729; PF00185 | Aspartate/ornithine carbamoyltransferase, carbamoyl-P binding domain; Aspartate/ornithine carbamoyltransferase, Asp/Orn binding domain | E | KOG1504 | Ornithine carbamoyltransferase OTC/ARG3 |
| 22.579 | 6.5 | 1 | 1 | 3 | 1.446 | 1.585 | 1.484 | 0.699 | 0.764 | 0.637 | plasma membrane | K00551 | PEMT; phosphatidylethanolamine/phosphatidyl-N-methylethanolamine N-methyltransferase [EC:2.1.1.17 2.1.1.71] | map00564 Glycerophospholipid metabolism; map01100 Metabolic pathways | | | I | KOG4142 | Phospholipid methyltransferase |
| 27.372 | 65 | 15 | 15 | 44 | 0.845 | 0.876 | 0.902 | 1.08 | 1.253 | 1.162 | cytoplasm | K02730 | PSMA6; 20S proteasome subunit alpha 1 [EC:3.4.25.1] | map03050 Proteasome | PF10584; PF00227 | Proteasome subunit A N-terminal signature; Proteasome subunit | O | KOG0182 | 20S proteasome, regulatory subunit alpha type PSMA6/SCL1 |
| 70.865 | 5.3 | 3 | 3 | 3 | 1.17 | 1.142 | 1.208 | 0.87 | 0.766 | 0.796 | plasma membrane | K14640 | SLC20A, PIT; solute carrier family 20 (sodium-dependent phosphate transporter) | | | | P | KOG2493 | Na+/Pi symporter |
| 37.328 | 15.2 | 6 | 6 | 11 | 1.163 | 1.11 | 1.124 | 0.89 | 0.922 | 0.771 | plasma membrane | K23503 | SFXN5; sideroflexin-5 | |  |  | S | KOG3767 | Sideroflexin |
| 31.814 | 44.7 | 12 | 12 | 40 | 1.288 | 1.141 | 1.122 | 0.87 | 0.855 | 0.976 | mitochondria | K00235 | SDHB, SDH2; succinate dehydrogenase (ubiquinone) iron-sulfur subunit [EC:1.3.5.1] | map00020 Citrate cycle (TCA cycle); map00190 Oxidative phosphorylation; map01100 Metabolic pathways; map01200 Carbon metabolism; map04714 Thermogenesis; map04932 Non-alcoholic fatty liver disease (NAFLD); map05010 Alzheimer disease; map05012 Parkinson disease; map05016 Huntington disease | PF13085; PF13534 | 2Fe-2S iron-sulfur cluster binding domain; 4Fe-4S dicluster domain | C | KOG3049 | Succinate dehydrogenase, Fe-S protein subunit |
| 9.2864 | 26.5 | 2 | 2 | 3 | 2.02 | 1.741 | 1.963 | 0.56 | 0.537 | 0.642 | extracellular | K17780 | TIM8; mitochondrial import inner membrane translocase subunit TIM8 | | PF02953 | Tim10/DDP family zinc finger | U | KOG3489 | Mitochondrial import inner membrane translocase, subunit TIM8 |
| 88.324 | 5.7 | 4 | 4 | 7 | 0.879 | 0.737 | 0.789 | 1.023 | 1.031 | 1.144 | nucleus | K09566 | PPIG; peptidyl-prolyl isomerase G (cyclophilin G) [EC:5.2.1.8] | | PF00160 | Cyclophilin type peptidyl-prolyl cis-trans isomerase/CLD | O | KOG0546 | HSP90 co-chaperone CPR7/Cyclophilin |
| 27.855 | 55.2 | 14 | 14 | 29 | 0.84 | 0.811 | 0.786 | 1.146 | 1.281 | 1.179 | cytoplasm | K02731 | PSMA7; 20S proteasome subunit alpha 4 [EC:3.4.25.1] | map03050 Proteasome | PF10584; PF00227 | Proteasome subunit A N-terminal signature; Proteasome subunit | O | KOG0183 | 20S proteasome, regulatory subunit alpha type PSMA7/PRE6 |
| 76.202 | 25.4 | 19 | 19 | 47 | 1.208 | 1.121 | 1.07 | 0.845 | 0.877 | 0.662 | endoplasmic reticulum | K08748 | SLC27A5, FATP5; solute carrier family 27 (fatty acid transporter), member 5 [EC:6.2.1.7] | map00120 Primary bile acid biosynthesis; map01100 Metabolic pathways; map03320 PPAR signaling pathway; map04931 Insulin resistance; map04976 Bile secretion | PF13193 | Domain of unknown function (DUF4009) | I | KOG1179 | Very long-chain acyl-CoA synthetase/fatty acid transporter |
| 52.62 | 8.5 | 5 | 3 | 7 | 1.276 | 1.133 | 1.157 | 0.872 | 0.674 | 0.687 | extracellular | K14684 | SLC25A23S; solute carrier family 25 (mitochondrial phosphate transporter), member 23/24/25/41 | | PF13833; PF13499 | EF-hand domain pair; EF-hand domain pair | C | KOG0036 | Predicted mitochondrial carrier protein |
| 33.189 | 41.7 | 10 | 10 | 17 | 0.869 | 0.875 | 0.847 | 1.134 | 1.146 | 1.155 | cytoplasm | K15296 | NAPA, SNAPA, SEC17; alpha-soluble NSF attachment protein | map04721 Synaptic vesicle cycle | | | U | KOG1586 | Protein required for fusion of vesicles in vesicular transport, alpha-SNAP |
| 26.022 | 30.4 | 6 | 4 | 15 | 0.739 | 0.732 | 0.815 | 1.12 | 1.118 | 0.994 | extracellular | K20346 | TMED4_9_11; p24 family protein alpha | | PF01105 | emp24/gp25L/p24 family/GOLD | U | KOG1690 | emp24/gp25L/p24 family of membrane trafficking proteins |
| 35.216 | 10.6 | 3 | 3 | 5 | 0.747 | 0.886 | 0.731 | 1.168 | 0.985 | 1.085 | plasma membrane | K01080 | PLPP1_2_3; phosphatidate phosphatase [EC:3.1.3.4] | map00561 Glycerolipid metabolism; map00564 Glycerophospholipid metabolism; map00565 Ether lipid metabolism;  map00600 Sphingolipid metabolism; map01100 Metabolic pathways; map04072 Phospholipase D signaling pathway;  map04666 Fc gamma R-mediated phagocytosis; map04975 Fat digestion and absorption; map05231 Choline metabolism in cancer | | | I | KOG3030 | Lipid phosphate phosphatase and related enzymes of the PAP2 family |
| 41.811 | 1.8 | 1 | 1 | 2 | 1.397 | 1.427 | 1.462 | 0.981 | 0.756 | 0.519 | extracellular |  |  |  | PF00354 | Pentaxin family | |  |  |
| 74.396 | 25.2 | 17 | 15 | 37 | 1.117 | 1 | 1.172 | 0.757 | 0.704 | 0.59 | plasma membrane | K03460 | SLCO1A; solute carrier organic anion transporter family, member 1A | map04976 Bile secretion | PF07648 | Kazal-type serine protease inhibitor domain | P | KOG3626 | Organic anion transporter |
| 33.213 | 7 | 2 | 1 | 6 | 1.286 | 1.303 | 1.509 | 0.659 | 0.716 | 0.78 | cytoplasm | K11822 | SULT2A1; bile-salt sulfotransferase [EC:2.8.2.14] | map00980 Metabolism of xenobiotics by cytochrome P450; map04976 Bile secretion; map05204 Chemical carcinogenesis | PF00685 | Sulfotransferase domain | S | KOG1584 | Sulfotransferase |
| 212.73 | 1.5 | 2 | 2 | 2 | 1.173 | 0.946 | 1.149 | 0.895 | 0.727 | 0.803 | nucleus | K20915 | TP53BP1, P202; tumor suppressor p53-binding protein 1 | map04621 NOD-like receptor signaling pathway | PF09038 | Tumour suppressor p53-binding protein-1 Tudor | K | KOG3548 | DNA damage checkpoint protein RHP9/CRB2/53BP1 |
| 56.979 | 48 | 26 | 26 | 86 | 0.896 | 0.856 | 0.911 | 1.084 | 1.13 | 1.41 | cytoplasm | K00963 | UGP2, galU, galF; UTP--glucose-1-phosphate uridylyltransferase [EC:2.7.7.9] | map00040 Pentose and glucuronate interconversions; map00052 Galactose metabolism; map00500 Starch and sucrose metabolism;  map00520 Amino sugar and nucleotide sugar metabolism; map01100 Metabolic pathways | | | G | KOG2638 | UDP-glucose pyrophosphorylase |
| 19.029 | 10.1 | 2 | 2 | 2 | 1.73 | 1.56 | 1.323 | 0.686 | 0.548 | 0.769 | nucleus | K07766 | E3.6.1.52; diphosphoinositol-polyphosphate diphosphatase [EC:3.6.1.52] | | PF00293 | NUDIX domain | F | KOG2839 | Diadenosine and diphosphoinositol polyphosphate phosphohydrolase |
| 26.738 | 28.3 | 7 | 7 | 10 | 0.8 | 0.85 | 0.828 | 1.12 | 1.505 | 1.158 | cytoplasm | K13288 | orn, REX2, REXO2; oligoribonuclease [EC:3.1.-.-] | map03008 Ribosome biogenesis in eukaryotes | | | L | KOG3242 | Oligoribonuclease (3'->5' exoribonuclease) |
| 70.683 | 3.4 | 2 | 2 | 3 | 0.548 | 0.537 | 0.566 | 0.907 | 1.217 | 1.12 | nucleus | K12484 | RAB11FIP1_2_5; Rab11 family-interacting protein 1/2/5 | map04144 Endocytosis | PF00168 | C2 domain |  |  |  |
| 22.382 | 49 | 8 | 5 | 27 | 0.854 | 0.851 | 0.805 | 1.264 | 1.194 | 1.18 | cytoplasm | K07953 | SAR1; GTP-binding protein SAR1 [EC:3.6.5.-] | map04141 Protein processing in endoplasmic reticulum; map05134 Legionellosis | PF00025 | ADP-ribosylation factor family | U | KOG0077 | Vesicle coat complex COPII, GTPase subunit SAR1 |
| 20.046 | 17.1 | 3 | 3 | 3 | 0.887 | 0.838 | 0.788 | 1.134 | 1.158 | 1.144 | nucleus |  |  |  |  |  |  |  |  |
| 66.357 | 16.2 | 8 | 8 | 9 | 0.819 | 0.908 | 0.857 | 1.182 | 1.32 | 1.05 | cytoplasm,nucleus | K15300 | STXBP2, MUNC18-2; syntaxin-binding protein 2 | | | | U | KOG1300 | Vesicle trafficking protein Sec1 |
| 10.344 | 38.2 | 3 | 3 | 5 | 1.447 | 1.385 | 1.375 | 0.818 | 0.856 | 0.814 | extracellular | K17777 | TIM9; mitochondrial import inner membrane translocase subunit TIM9 | | PF02953 | Tim10/DDP family zinc finger | U | KOG3479 | Mitochondrial import inner membrane translocase, subunit TIM9 |
| 54.495 | 20.1 | 7 | 6 | 8 | 0.702 | 0.734 | 0.619 | 1.444 | 1.171 | 0.921 | cytoplasm | K23001 | TOR1AIP; torsin-1A-interacting protein | | | |  |  |  |
| 27.911 | 33.2 | 7 | 7 | 14 | 1.14 | 1.215 | 1.132 | 0.885 | 0.871 | 0.722 | mitochondria | K17796 | TIM21; mitochondrial import inner membrane translocase subunit TIM21 | | PF08294 | TIM21 | U | KOG4836 | Uncharacterized conserved protein |
| 29.18 | 41.3 | 9 | 9 | 14 | 0.798 | 0.897 | 0.716 | 1.227 | 1.29 | 1.137 | cytoplasm | K00069 | HPGD; 15-hydroxyprostaglandin dehydrogenase (NAD) [EC:1.1.1.141] | map05202 Transcriptional misregulation in cancer | PF00106 | short chain dehydrogenase | Q | KOG4169 | 15-hydroxyprostaglandin dehydrogenase and related dehydrogenases |
| 47.655 | 28 | 10 | 4 | 19 | 0.846 | 0.863 | 0.927 | 1.208 | 1.092 | 1.274 | cytoplasm,nucleus | K10752 | RBBP4, HAT2, CAF1, MIS16; histone-binding protein RBBP4 | map04218 Cellular senescence | | | B | KOG0264 | Nucleosome remodeling factor, subunit CAF1/NURF55/MSI1 |
| 12.554 | 40 | 5 | 5 | 13 | 1.163 | 1.082 | 1.069 | 0.887 | 0.73 | 0.84 | cytoplasm | K02917 | RP-L35Ae, RPL35A; large subunit ribosomal protein L35Ae | map03010 Ribosome | PF01247 | Ribosomal protein L35Ae | J | KOG0887 | 60S ribosomal protein L35A/L37 |
| 43.995 | 51.2 | 17 | 8 | 92 | 0.539 | 0.681 | 0.546 | 1.993 | 1.196 | 1.848 | mitochondria | K07513 | ACAA1; acetyl-CoA acyltransferase 1 [EC:2.3.1.16] | map00071 Fatty acid degradation; map00280 Valine, leucine and isoleucine degradation; map00592 alpha-Linolenic acid metabolism; map01040 Biosynthesis of unsaturated fatty acids; map01100 Metabolic pathways; map01212 Fatty acid metabolism; map03320 PPAR signaling pathway; map04146 Peroxisome | PF00108; PF02803 | Thiolase, N-terminal domain; Thiolase, C-terminal domain | I | KOG1389 | 3-oxoacyl CoA thiolase |
| 33.665 | 12.5 | 4 | 3 | 11 | 1.319 | 1.767 | 1.849 | 0.82 | 0.611 | 0.692 | nucleus | K12897 | TRA2; transformer-2 protein | map03040 Spliceosome | PF00076 | RNA recognition motif. (a.k.a. RRM, RBD, or RNP domain) | A | KOG0118 | FOG: RRM domain |
| 61.012 | 18.1 | 7 | 7 | 7 | 1.401 | 1.604 | 1.313 | 0.901 | 0.756 | 0.814 | nucleus |  |  |  |  |  |  |  |  |
| 10.531 | 10.9 | 1 | 1 | 1 | 1.459 | 1.424 | 1.627 | 0.784 | 0.622 | 0.923 | mitochondria | K02914 | RP-L34, MRPL34, rpmH; large subunit ribosomal protein L34 | map03010 Ribosome | |  | J | KOG4612 | Mitochondrial ribosomal protein L34 |
| 31.689 | 17.7 | 5 | 5 | 6 | 1.07 | 1.455 | 1.428 | 0.817 | 0.854 | 0.834 | extracellular | K15123 | SLC25A45_47; solute carrier family 25, member 45/47 | | | | C | KOG0758 | Mitochondrial carnitine-acylcarnitine carrier protein |
| 49.939 | 32.1 | 10 | 10 | 12 | 1.13 | 1.214 | 1.286 | 0.836 | 0.914 | 0.966 | nucleus | K17409 | MRPS30; small subunit ribosomal protein S30 | | | | J | KOG4461 | Mitochondrial 28S ribosomal protein S30 |
| 76.358 | 1.2 | 1 | 1 | 1 | 1.47 | 1.836 | 1.844 | 0.737 | 0.686 | 0.723 | nucleus | K18499 | SKIL; Ski-like protein | map04550 Signaling pathways regulating pluripotency of stem cells | PF08782 | c-SKI Smad4 binding domain | | |  |
| 42.705 | 11.6 | 4 | 4 | 5 | 0.809 | 0.698 | 0.652 | 1.366 | 1.008 | 1.078 | extracellular |  |  |  |  |  |  |  |  |
| 122.89 | 3.5 | 4 | 2 | 4 | 1.262 | 1.515 | 1.343 | 0.73 | 0.583 | 0.706 | cytoplasm | K11649 | SMARCC; SWI/SNF related-matrix-associated actin-dependent regulator of chromatin subfamily C | map04714 Thermogenesis; map05225 Hepatocellular carcinoma | PF04433; PF00249 | SWIRM domain; Myb-like DNA-binding domain | B | KOG1279 | Chromatin remodeling factor subunit and related transcription factors |
| 9.9583 | 44.8 | 4 | 4 | 13 | 1.301 | 1.118 | 1.023 | 0.725 | 0.778 | 0.639 | mitochondria | K09481 | SEC61B, SBH2; protein transport protein SEC61 subunit beta | map03060 Protein export; map04141 Protein processing in endoplasmic reticulum; map04145 Phagosome; map05110 Vibrio cholerae infection | | | U | KOG3457 | Sec61 protein translocation complex, beta subunit |
| 35.897 | 5 | 1 | 1 | 1 | 0.862 | 0.858 | 0.935 | 1.062 | 1.301 | 1.127 | extracellular | K22999 | TOR2A; torsin-2A | |  |  | O | KOG2170 | ATPase of the AAA+ superfamily |
| 67.544 | 14.8 | 9 | 9 | 11 | 1.153 | 1.108 | 1.182 | 0.822 | 1.002 | 0.824 | plasma membrane | K17087 | TM9SF3; transmembrane 9 superfamily member 3 | | | | U | KOG1278 | Endosomal membrane proteins, EMP70 |
| 167.64 | 1.2 | 2 | 2 | 3 | 1.579 | 1.434 | 1.628 | 0.645 | 0.666 | 0.574 | nucleus | K16777 | NEURL4; neuralized-like protein 4 | | PF07177 | Neuralized | T | KOG4625 | Notch signaling protein Neuralized, Nuez domain |
| 24.192 | 12.3 | 2 | 2 | 2 | 0.763 | 0.818 | 0.79 | 1.034 | 1.027 | 1.033 | cytoplasm |  |  |  | PF06201 | PITH domain | O | KOG1730 | Thioredoxin-like protein |
| 9.7681 | 54.9 | 6 | 6 | 25 | 1.36 | 1.371 | 1.474 | 0.703 | 0.708 | 0.754 | mitochondria | K00418 | QCR8, UQCRQ; ubiquinol-cytochrome c reductase subunit 8 | map00190 Oxidative phosphorylation; map01100 Metabolic pathways; map04260 Cardiac muscle contraction; map04714 Thermogenesis;  map04932 Non-alcoholic fatty liver disease (NAFLD); map05010 Alzheimer disease; map05012 Parkinson disease;  map05016 Huntington disease | | | C | KOG4116 | Ubiquinol cytochrome c reductase, subunit QCR8 |
| 34.119 | 8 | 3 | 3 | 7 | 1.51 | 1.505 | 1.062 | 0.854 | 0.499 | 0.88 | plasma membrane | K00852 | rbsK, RBKS; ribokinase [EC:2.7.1.15] | map00030 Pentose phosphate pathway; map01100 Metabolic pathways | | | G | KOG2855 | Ribokinase |
| 24.305 | 37.4 | 8 | 8 | 11 | 1.572 | 1.403 | 1.296 | 0.825 | 0.44 | 0.755 | cytoplasm | K02873 | RP-L13e, RPL13; large subunit ribosomal protein L13e | map03010 Ribosome | |  | J | KOG3295 | 60S Ribosomal protein L13 |
| 68.284 | 8 | 3 | 3 | 5 | 1.146 | 1.271 | 1.232 | 0.861 | 0.832 | 0.778 | plasma membrane | K05046 | SLC6A13, GAT2; solute carrier family 6 (neurotransmitter transporter, GABA) member 13 | map04721 Synaptic vesicle cycle; map04727 GABAergic synapse | | | P | KOG3660 | Sodium-neurotransmitter symporter |
| 24.785 | 27.6 | 6 | 6 | 11 | 0.697 | 0.766 | 0.613 | 1.943 | 1.692 | 1.442 | cytoplasm |  |  |  | PF01852 | START domain | I | KOG2761 | START domain-containing proteins involved in steroidogenesis/phosphatidylcholine transfer |
| 29.659 | 3.4 | 1 | 1 | 1 | 0.703 | 0.862 | 0.702 | 1.25 | 1.009 | 1.31 | nucleus |  |  |  | PF13902; PF01424 | R3H-associated N-terminal domain; R3H domain | S | KOG1478 | 3-keto sterol reductase |
| 50.421 | 19.2 | 7 | 7 | 7 | 0.951 | 0.742 | 0.705 | 1.14 | 1.234 | 1.18 | nucleus | K14775 | UTP30, RSL1D1; ribosome biogenesis protein UTP30 | | PF00687 | Ribosomal protein L1p/L10e family | J | KOG1685 | Uncharacterized conserved protein |
| 28.152 | 30.7 | 7 | 7 | 12 | 1.181 | 1.322 | 1.258 | 0.88 | 0.894 | 0.895 | cytoplasm | K15433 | CGI99, CLE7, RLLM1; RLL motif containing protein 1 | | | | K | KOG4380 | Carnitine deficiency associated protein |
| 36.289 | 4.4 | 1 | 1 | 1 | 1.847 | 1.126 | 0.976 | 0.676 | 0.567 | 0.79 | plasma membrane | K08474 | TAS2R; taste receptor type 2 | map04742 Taste transduction | | |  |  |  |
| 59.623 | 50.5 | 26 | 26 | 68 | 0.865 | 0.83 | 0.83 | 1.156 | 1.219 | 1.227 | cytoplasm | K09497 | CCT5; T-complex protein 1 subunit epsilon | | | | O | KOG0357 | Chaperonin complex component, TCP-1 epsilon subunit (CCT5) |
| 43.561 | 15.4 | 6 | 6 | 9 | 0.819 | 0.987 | 0.855 | 1.29 | 1.354 | 1.124 | nucleus | K01062 | PLA2G7, PAFAH; platelet-activating factor acetylhydrolase [EC:3.1.1.47] | map00565 Ether lipid metabolism; map01100 Metabolic pathways | PF03403 | Platelet-activating factor acetylhydrolase, isoform II | I | KOG3847 | Phospholipase A2 (platelet-activating factor acetylhydrolase in humans) |
| 22.223 | 5.6 | 1 | 1 | 1 | 0.835 | 0.839 | 0.88 | 1.172 | 1.194 | 1.158 | cytoskeleton | |  |  |  |  | S | KOG4093 | Uncharacterized conserved protein |
| 21.708 | 46.3 | 7 | 7 | 15 | 1.323 | 1.376 | 1.288 | 0.838 | 0.881 | 0.965 | mitochondria | K02935 | RP-L7, MRPL12, rplL; large subunit ribosomal protein L7/L12 | map03010 Ribosome | PF00542 | Ribosomal protein L7/L12 C-terminal domain | J | KOG1715 | Mitochondrial/chloroplast ribosomal protein L12 |
| 6.6767 | 19.6 | 1 | 1 | 3 | 1.476 | 1.424 | 1.288 | 1.012 | 0.808 | 0.734 | extracellular | K02980 | RP-S29e, RPS29; small subunit ribosomal protein S29e | map03010 Ribosome | |  | J | KOG3506 | 40S ribosomal protein S29 |
| 58.507 | 6.4 | 4 | 4 | 4 | 1.266 | 1.205 | 1.148 | 0.973 | 0.879 | 0.803 | extracellular |  |  |  |  |  |  |  |  |
| 105.54 | 2 | 2 | 2 | 2 | 1.242 | 1.331 | 1.249 | 0.822 | 0.718 | 0.852 | mitochondria | K01109 | INPP4; inositol polyphosphate-4-phosphatase [EC:3.1.3.66] | map00562 Inositol phosphate metabolism; map01100 Metabolic pathways; map04070 Phosphatidylinositol signaling system | | | T | KOG4428 | Inositol-polyphosphate 4-phosphatase |
| 128.33 | 2.1 | 3 | 3 | 3 | 0.56 | 0.533 | 0.353 | 1.393 | 1.251 | 1.031 | peroxisome | K05718 | ITGAL, CD11a; integrin alpha L | map04015 Rap1 signaling pathway; map04514 Cell adhesion molecules (CAMs); map04650 Natural killer cell mediated cytotoxicity; map04670 Leukocyte transendothelial migration; map04810 Regulation of actin cytoskeleton; map05144 Malaria; map05150 Staphylococcus aureus infection; map05166 Human T-cell leukemia virus 1 infection; map05169 Epstein-Barr virus infection; map05323 Rheumatoid arthritis; map05416 Viral myocarditis | PF00092 | von Willebrand factor type A domain | W | KOG3637 | Vitronectin receptor, alpha subunit |
| 20.638 | 68.9 | 13 | 3 | 94 | 1.686 | 2.611 | 2.759 | 0.198 | 0.262 | 0.57 | extracellular |  |  |  | PF00061 | Lipocalin / cytosolic fatty-acid binding protein family | | | |
| 9.3267 | 56.1 | 6 | 6 | 26 | 1.176 | 1.16 | 1.226 | 0.805 | 0.837 | 0.845 | extracellular | K03948 | NDUFA4; NADH dehydrogenase (ubiquinone) 1 alpha subcomplex subunit 4 | map00190 Oxidative phosphorylation; map01100 Metabolic pathways; map04714 Thermogenesis; map04723 Retrograde endocannabinoid signaling;  map04932 Non-alcoholic fatty liver disease (NAFLD); map05010 Alzheimer disease; map05012 Parkinson disease; map05016 Huntington disease | | | | | |
| 42.554 | 5.1 | 2 | 2 | 2 | 1.388 | 1.227 | 1.153 | 0.936 | 0.949 | 0.924 | nucleus | K15182 | RDBP, NELFE; negative elongation factor E | | PF00076 | RNA recognition motif. (a.k.a. RRM, RBD, or RNP domain) | | | |
| 114.99 | 14.7 | 14 | 14 | 15 | 0.916 | 0.798 | 0.824 | 1.161 | 1.087 | 1.101 | nucleus | K06270 | PPP1R12A, MYPT1; protein phosphatase 1 regulatory subunit 12A | map04022 cGMP-PKG signaling pathway; map04024 cAMP signaling pathway; map04270 Vascular smooth muscle contraction;  map04510 Focal adhesion; map04611 Platelet activation; map04810 Regulation of actin cytoskeleton; map04921 Oxytocin signaling pathway;  map05205 Proteoglycans in cancer | | | OT | KOG0505 | Myosin phosphatase, regulatory subunit |
| 39.966 | 8.3 | 3 | 1 | 8 | 1.306 | 0.974 | 1.675 | 0.825 | 0.715 | 0.853 | cytoplasm | K04631 | GNAT1_2; guanine nucleotide-binding protein G(t) subunit alpha 1/2 | map04744 Phototransduction | PF00503 | G-protein alpha subunit | T | KOG0082 | G-protein alpha subunit (small G protein superfamily) |
| 64.753 | 6.3 | 3 | 3 | 3 | 1.188 | 1.275 | 1.212 | 0.765 | 0.723 | 0.854 | mitochondria | K00643 | E2.3.1.37, ALAS; 5-aminolevulinate synthase [EC:2.3.1.37] | map00260 Glycine, serine and threonine metabolism; map00860 Porphyrin and chlorophyll metabolism; map01100 Metabolic pathways | PF09029; PF00155 | 5-aminolevulinate synthase presequence; Aminotransferase class I and II | H | KOG1360 | 5-aminolevulinate synthase |
| 40.092 | 11.3 | 4 | 4 | 4 | 0.75 | 0.654 | 0.7 | 1.026 | 1.094 | 1.134 | mitochondria | K11251 | H2A; histone H2A | map04217 Necroptosis; map05034 Alcoholism; map05322 Systemic lupus erythematosus | PF00125; PF01661 | Core histone H2A/H2B/H3/H4; Macro domain | B | KOG2633 | Hismacro and SEC14 domain-containing proteins |
| 29.12 | 3.9 | 1 | 1 | 1 | 0.456 | 0.413 | 0.408 | 0.831 | 0.69 | 0.85 | endoplasmic reticulum | K06752 | MHC2; MHC class II antigen | map04145 Phagosome; map04514 Cell adhesion molecules (CAMs); map04612 Antigen processing and presentation; map04640 Hematopoietic cell lineage; map04658 Th1 and Th2 cell differentiation; map04659 Th17 cell differentiation; map04672 Intestinal immune network for IgA production; map04940 Type I diabetes mellitus; map05140 Leishmaniasis; map05145 Toxoplasmosis; map05150 Staphylococcus aureus infection; map05152 Tuberculosis; map05164 Influenza A; map05166 Human T-cell leukemia virus 1 infection; map05168 Herpes simplex virus 1 infection; map05169 Epstein-Barr virus infection; map05310 Asthma; map05320 Autoimmune thyroid disease; map05321 Inflammatory bowel disease (IBD); map05322 Systemic lupus erythematosus; map05323 Rheumatoid arthritis; map05330 Allograft rejection; map05332 Graft-versus-host disease; map05416 Viral myocarditis | PF00993; PF07654 | Class II histocompatibility antigen, alpha domain; Immunoglobulin C1-set domain | | | |
| 71.253 | 1.4 | 1 | 1 | 1 | 0.837 | 0.841 | 0.926 | 1.268 | 1.182 | 1.105 | extracellular | K01217 | IDUA; L-iduronidase [EC:3.2.1.76] | map00531 Glycosaminoglycan degradation; map01100 Metabolic pathways; map04142 Lysosome | | | | | |
| 46.852 | 16.7 | 8 | 8 | 10 | 0.891 | 0.826 | 0.696 | 1.197 | 0.988 | 1.129 | cytoplasm | K05411 | IRF3; interferon regulatory factor 3 | map04620 Toll-like receptor signaling pathway; map04621 NOD-like receptor signaling pathway; map04622 RIG-I-like receptor signaling pathway; map04623 Cytosolic DNA-sensing pathway; map05131 Shigellosis; map05133 Pertussis; map05135 Yersinia infection; map05160 Hepatitis C; map05161 Hepatitis B; map05162 Measles; map05163 Human cytomegalovirus infection; map05164 Influenza A; map05165 Human papillomavirus infection; map05167 Kaposi sarcoma-associated herpesvirus infection; map05168 Herpes simplex virus 1 infection; map05169 Epstein-Barr virus infection; map05170 Human immunodeficiency virus 1 infection; map05203 Viral carcinogenesis | PF00605 | Interferon regulatory factor transcription factor | | | |
| 48.937 | 12.3 | 5 | 5 | 5 | 0.634 | 0.575 | 0.644 | 1.095 | 1.151 | 1.028 | nucleus | K08958 | CSNK1G; casein kinase 1, gamma [EC:2.7.11.1] | map04340 Hedgehog signaling pathway; map04341 Hedgehog signaling pathway - fly | PF00069 | Protein kinase domain | T | KOG1165 | Casein kinase (serine/threonine/tyrosine protein kinase) |
| 12.295 | 18.3 | 2 | 2 | 3 | 0.779 | 0.825 | 0.864 | 1.188 | 1.293 | 1.067 | cytoplasm | K07401 | K07401; selenoprotein W-related protein | | | |  |  |  |
| 13.996 | 60.5 | 8 | 8 | 10 | 0.843 | 0.68 | 0.857 | 1.14 | 1.008 | 1.183 | cytoplasm | K01112 | PHPT1; phosphohistidine phosphatase [EC:3.9.1.3] | | | | |  |  |
| 14.254 | 14.8 | 2 | 2 | 2 | 1.387 | 1.265 | 1.237 | 0.825 | 0.899 | 0.849 | cytoplasm | K09548 | PFDN1; prefoldin subunit 1 | | |  | O | KOG3501 | Molecular chaperone Prefoldin, subunit 1 |
| 17.234 | 24 | 3 | 3 | 4 | 2.318 | 1.341 | 1.995 | 0.778 | 0.403 | 0.517 | mitochondria | K01522 | FHIT; bis(5'-adenosyl)-triphosphatase [EC:3.6.1.29] | map00230 Purine metabolism; map01100 Metabolic pathways; map05222 Small cell lung cancer; map05223 Non-small cell lung cancer | PF01230 | HIT domain | K | KOG3379 | Diadenosine polyphosphate hydrolase and related proteins of the histidine triad (HIT) family |
| 42.295 | 28.8 | 11 | 11 | 20 | 0.939 | 0.807 | 0.867 | 1.074 | 1.308 | 1.292 | nucleus | K00849 | galK; galactokinase [EC:2.7.1.6] | map00052 Galactose metabolism; map00520 Amino sugar and nucleotide sugar metabolism; map01100 Metabolic pathways | PF10509 | Galactokinase galactose-binding signature | G | KOG0631 | Galactokinase |
| 56.451 | 57.6 | 22 | 22 | 60 | 1.257 | 1.312 | 1.274 | 0.831 | 0.736 | 0.95 | mitochondria | K13524 | ABAT; 4-aminobutyrate aminotransferase / (S)-3-amino-2-methylpropionate transaminase [EC:2.6.1.19 2.6.1.22] | map00250 Alanine, aspartate and glutamate metabolism; map00280 Valine, leucine and isoleucine degradation; map00410 beta-Alanine metabolism; map00640 Propanoate metabolism; map00650 Butanoate metabolism; map01100 Metabolic pathways; map04727 GABAergic synapse | PF00202 | Aminotransferase class-III | E | KOG1405 | 4-aminobutyrate aminotransferase |
| 38.822 | 21.3 | 6 | 6 | 10 | 0.84 | 0.808 | 0.907 | 1.08 | 1.207 | 1.136 | cytoplasm | K01551 | arsA, ASNA1, GET3; arsenite/tail-anchored protein-transporting ATPase [EC:7.3.2.7 7.3.-.-] | | PF02374 | Anion-transporting ATPase | P | KOG2825 | Putative arsenite-translocating ATPase |
| 36.946 | 15.3 | 4 | 3 | 4 | 0.825 | 0.777 | 0.892 | 1.104 | 1.31 | 1.062 | cytoplasm | K10436 | MAPRE; microtubule-associated protein, RP/EB family | | PF00307 | Calponin homology (CH) domain | Z | KOG3000 | Microtubule-binding protein involved in cell cycle control |
| 77.999 | 11 | 7 | 7 | 8 | 1.024 | 1.516 | 1.047 | 0.859 | 0.695 | 0.75 | plasma membrane | K05655 | ABCB8; ATP-binding cassette, subfamily B (MDR/TAP), member 8 | map02010 ABC transporters | PF00005 | ABC transporter | U | KOG0058 | Peptide exporter, ABC superfamily |
| 24.683 | 18.8 | 5 | 5 | 11 | 1.24 | 1.244 | 1.38 | 0.753 | 0.783 | 0.768 | mitochondria | K03940 | NDUFS7; NADH dehydrogenase (ubiquinone) Fe-S protein 7 [EC:7.1.1.2 1.6.99.3] | map00190 Oxidative phosphorylation; map01100 Metabolic pathways; map04714 Thermogenesis; map04723 Retrograde endocannabinoid signaling;  map04932 Non-alcoholic fatty liver disease (NAFLD); map05010 Alzheimer disease; map05012 Parkinson disease; map05016 Huntington disease | | | C | KOG1687 | NADH-ubiquinone oxidoreductase, NUFS7/PSST/20 kDa subunit |
| 55.792 | 1.4 | 1 | 1 | 1 | 0.651 | 0.713 | 0.863 | 1.133 | 0.956 | 1.23 | nucleus | K14840 | NOP53, GLTSCR2; nucleolar protein 53 | | | | S | KOG2823 | Cellular protein (glioma tumor suppressor candidate region gene 2) |
| 60.577 | 11.3 | 7 | 7 | 10 | 1.276 | 1.331 | 1.207 | 0.7 | 0.702 | 0.662 | cytoplasm | K07422 | CYP2U1; cytochrome P450 family 2 subfamily U polypeptide 1 | map00590 Arachidonic acid metabolism; map01100 Metabolic pathways | PF00067 | Cytochrome P450 | Q | KOG0156 | Cytochrome P450 CYP2 subfamily |
| 21.854 | 3.6 | 1 | 1 | 2 | 0.47 | 0.397 | 0.423 | 1.67 | 1.815 | 0.987 | cytoplasm |  |  |  |  |  |  |  |  |
| 47.24 | 4.3 | 2 | 2 | 2 | 1.138 | 1.221 | 1.166 | 0.854 | 0.937 | 0.893 | extracellular | K21027 | TRMU, SLM3; tRNA-5-taurinomethyluridine 2-sulfurtransferase [EC:2.8.1.14] | | | | J | KOG2805 | tRNA (5-methylaminomethyl-2-thiouridylate)-methyltransferase |
| 12.648 | 58.5 | 7 | 7 | 15 | 1.226 | 1.196 | 1.284 | 0.857 | 0.802 | 0.927 | mitochondria | K03938 | NDUFS5; NADH dehydrogenase (ubiquinone) Fe-S protein 5 | map00190 Oxidative phosphorylation; map01100 Metabolic pathways; map04714 Thermogenesis; map04723 Retrograde endocannabinoid signaling;  map04932 Non-alcoholic fatty liver disease (NAFLD); map05010 Alzheimer disease; map05012 Parkinson disease; map05016 Huntington disease | | | C | KOG4110 | NADH:ubiquinone oxidoreductase, NDUFS5/15kDa |
| 33.794 | 11.6 | 4 | 4 | 5 | 1.433 | 1.578 | 1.229 | 0.804 | 0.82 | 0.786 | cytoplasm | K01922 | PPCS, COAB; phosphopantothenate---cysteine ligase (ATP) [EC:6.3.2.51] | map00770 Pantothenate and CoA biosynthesis; map01100 Metabolic pathways | | | H | KOG2728 | Uncharacterized conserved protein with similarity to phosphopantothenoylcysteine synthetase/decarboxylase |
| 15.674 | 53.6 | 6 | 6 | 14 | 1.326 | 1.106 | 1.151 | 0.867 | 0.849 | 0.933 | extracellular |  |  |  |  |  | S | KOG3377 | Uncharacterized conserved protein |
| 43.232 | 23.2 | 8 | 8 | 14 | 0.726 | 0.819 | 0.783 | 1.019 | 1.262 | 1.402 | cytoplasm | K00965 | galT, GALT; UDPglucose--hexose-1-phosphate uridylyltransferase [EC:2.7.7.12] | map00052 Galactose metabolism; map00520 Amino sugar and nucleotide sugar metabolism; map01100 Metabolic pathways; map04917 Prolactin signaling pathway | PF01087; PF02744 | Galactose-1-phosphate uridyl transferase, N-terminal domain; Galactose-1-phosphate uridyl transferase, C-terminal domain | C | KOG2958 | Galactose-1-phosphate uridylyltransferase |
| 51.828 | 35.7 | 15 | 13 | 23 | 0.824 | 0.857 | 0.943 | 1.132 | 1.108 | 1.18 | cytoplasm | K17265 | G3BP1; Ras GTPase-activating protein-binding protein 1 [EC:3.6.4.12 3.6.4.13] | | PF02136; PF00076 | Nuclear transport factor 2 (NTF2) domain; RNA recognition motif. (a.k.a. RRM, RBD, or RNP domain) | A | KOG0116 | RasGAP SH3 binding protein rasputin, contains NTF2 and RRM domains |
| 43.008 | 7.1 | 2 | 2 | 2 | 0.73 | 0.566 | 0.923 | 1.264 | 1.34 | 1.326 | cytoplasm,nucleus | K18266 | NDRG1; protein NDRG1 | | PF03096 | Ndr family | S | KOG2931 | Differentiation-related gene 1 protein (NDR1 protein), related proteins |
| 19.331 | 28.7 | 5 | 5 | 5 | 0.838 | 0.774 | 0.742 | 1.486 | 1.682 | 1.334 | extracellular | K13916 | CAMP, LL37; cathelicidin antimicrobial peptide | map04621 NOD-like receptor signaling pathway; map04970 Salivary secretion; map05150 Staphylococcus aureus infection; map05152 Tuberculosis | PF00666 | Cathelicidin |  |  |  |
| 16.331 | 60.6 | 6 | 6 | 18 | 1.183 | 1.19 | 1.198 | 0.843 | 0.929 | 0.814 | cytoplasm | K03963 | NDUFB7; NADH dehydrogenase (ubiquinone) 1 beta subcomplex subunit 7 | map00190 Oxidative phosphorylation; map01100 Metabolic pathways; map04714 Thermogenesis; map04723 Retrograde endocannabinoid signaling;  map04932 Non-alcoholic fatty liver disease (NAFLD); map05010 Alzheimer disease; map05012 Parkinson disease; map05016 Huntington disease | | | C | KOG3468 | NADH:ubiquinone oxidoreductase, NDUFB7/B18 subunit |
| 41.963 | 18 | 7 | 7 | 9 | 1.166 | 1.222 | 1.356 | 0.983 | 0.726 | 0.798 | cytoplasm | K09650 | PARL, PSARL, PCP1; rhomboid-like protein [EC:3.4.21.105] | | | | T | KOG2980 | Integral membrane protease of the rhomboid family involved in different forms of regulated intramembrane proteolysis |
| 23.349 | 19.4 | 4 | 2 | 4 | 1.096 | 1.128 | 1.235 | 0.939 | 0.77 | 0.751 | cytoplasm | K07835 | RALB; Ras-related protein Ral-B | map04014 Ras signaling pathway; map04015 Rap1 signaling pathway; map04072 Phospholipase D signaling pathway; map05200 Pathways in cancer; map05210 Colorectal cancer; map05212 Pancreatic cancer | PF00071 | Ras family | U | KOG0395 | Ras-related GTPase |
| 97.254 | 45.7 | 34 | 34 | 104 | 1.19 | 1.276 | 1.276 | 0.755 | 0.756 | 0.81 | mitochondria | K00315 | DMGDH; dimethylglycine dehydrogenase [EC:1.5.8.4] | map00260 Glycine, serine and threonine metabolism; map01100 Metabolic pathways | PF01266; PF01571; PF08669 | FAD dependent oxidoreductase; Aminomethyltransferase folate-binding domain; Glycine cleavage T-protein C-terminal barrel domain | E | KOG2844 | Dimethylglycine dehydrogenase precursor |
| 15.525 | 12.2 | 2 | 2 | 3 | 0.498 | 0.424 | 0.53 | 1.386 | 1.068 | 1.19 | extracellular | K17782 | MIA40, CHCHD4; mitochondrial intermembrane space import and assembly protein 40 | | PF06747 | CHCH domain | S | KOG4149 | Uncharacterized conserved protein |
| 22.604 | 26.8 | 5 | 5 | 9 | 1.225 | 1.212 | 1.138 | 0.834 | 0.894 | 0.874 | mitochondria | |  |  |  |  | S | KOG4798 | Uncharacterized conserved protein |
| 160.47 | 0.8 | 1 | 1 | 3 | 0.545 | 0.46 | 0.595 | 1.063 | 0.922 | 2.043 | nucleus | K13240 | NOS1; nitric-oxide synthase, brain [EC:1.14.13.39] | map00220 Arginine biosynthesis; map00330 Arginine and proline metabolism; map01100 Metabolic pathways; map04020 Calcium signaling pathway; map04145 Phagosome; map04371 Apelin signaling pathway; map04713 Circadian entrainment; map04730 Long-term depression; map04926 Relaxin signaling pathway; map04970 Salivary secretion; map05010 Alzheimer disease; map05014 Amyotrophic lateral sclerosis (ALS) | PF00595; PF02898; PF00258; PF00667; PF00175 | PDZ domain (Also known as DHR or GLGF); Nitric oxide synthase, oxygenase domain; Flavodoxin; FAD binding domain; Oxidoreductase NAD-binding domain | C | KOG1158 | NADP/FAD dependent oxidoreductase |
| 49.485 | 4.5 | 2 | 2 | 3 | 0.907 | 0.861 | 0.731 | 1.056 | 1.149 | 1.108 | cytoplasm | K14295 | NUP50, NPAP60; nuclear pore complex protein Nup50 | map03013 RNA transport | PF08911 | NUP50 (Nucleoporin 50 kDa) | U | KOG2724 | Nuclear pore complex component NPAP60L/NUP50 |
| 105.62 | 4.8 | 5 | 5 | 5 | 0.802 | 0.857 | 0.766 | 1.122 | 1.051 | 1.074 | mitochondria | K18283 | PDE2A; cGMP-dependent 3',5'-cyclic phosphodiesterase [EC:3.1.4.17] | map00230 Purine metabolism; map01100 Metabolic pathways; map04022 cGMP-PKG signaling pathway; map04740 Olfactory transduction; map04925 Aldosterone synthesis and secretion; map05032 Morphine addiction | PF13185; PF01590; PF00233 | GAF domain; GAF domain; 3'5'-cyclic nucleotide phosphodiesterase | T | KOG3689 | Cyclic nucleotide phosphodiesterase |
| 34.786 | 25.5 | 7 | 3 | 8 | 2.087 | 2.48 | 1.941 | 0.413 | 0.432 | 0.46 | cytoplasm,nucleus | K00948 | PRPS, prsA; ribose-phosphate pyrophosphokinase [EC:2.7.6.1] | map00030 Pentose phosphate pathway; map00230 Purine metabolism; map01100 Metabolic pathways; map01200 Carbon metabolism; map01230 Biosynthesis of amino acids | PF13793 | N-terminal domain of ribose phosphate pyrophosphokinase | F | KOG1448 | Ribose-phosphate pyrophosphokinase |
| 33.104 | 10.4 | 3 | 3 | 3 | 0.908 | 0.868 | 0.875 | 1.1 | 1.192 | 1.179 | nucleus | K11875 | PSMG1, DSCR2, PAC1; proteasome assembly chaperone 1 | | | | |  |  |
| 35.892 | 25.1 | 7 | 7 | 8 | 0.698 | 0.732 | 0.716 | 0.974 | 1.634 | 1.022 | nucleus | K21918 | KCTD8_12_16; BTB/POZ domain-containing protein KCTD8/12/16 | | PF02214 | K+ channel tetramerisation domain | S | KOG2723 | Uncharacterized conserved protein, contains BTB/POZ domain |
| 57.844 | 41.4 | 20 | 19 | 37 | 0.784 | 0.751 | 0.83 | 1.037 | 1.612 | 1.068 | cytoplasm | K00873 | PK, pyk; pyruvate kinase [EC:2.7.1.40] | map00010 Glycolysis / Gluconeogenesis; map00230 Purine metabolism; map00620 Pyruvate metabolism; map01100 Metabolic pathways; map01200 Carbon metabolism; map01230 Biosynthesis of amino acids; map04922 Glucagon signaling pathway; map04930 Type II diabetes mellitus; map05165 Human papillomavirus infection; map05203 Viral carcinogenesis; map05230 Central carbon metabolism in cancer | PF02887 | Pyruvate kinase, alpha/beta domain | F | KOG2323 | Pyruvate kinase |
| 99.098 | 44.2 | 39 | 39 | 120 | 0.858 | 0.878 | 0.837 | 1.276 | 1.245 | 1.103 | endoplasmic reticulum | K14463 | MTTP, MTP; microsomal triglyceride transfer protein large subunit | map04975 Fat digestion and absorption | | | IU | KOG4337 | Microsomal triglyceride transfer protein |
| 141.43 | 18.9 | 24 | 24 | 28 | 0.824 | 0.945 | 0.78 | 1.398 | 1.211 | 1.232 | nucleus | K13338 | PEX1; peroxin-1 | map04146 Peroxisome | PF09263; PF09262; PF00004 | Peroxisome biogenesis factor 1, N-terminal ; Peroxisome biogenesis factor 1, N-terminal ; ATPase family associated with various cellular activities (AAA) | O | KOG0735 | AAA+-type ATPase |
| 42.202 | 6.6 | 2 | 2 | 2 | 1.194 | 1.168 | 1.196 | 0.845 | 1.026 | 0.841 | mitochondria | K19007 | AGPAT5; lysophosphatidiate acyltransferase [EC:2.3.1.51] | map00561 Glycerolipid metabolism; map00564 Glycerophospholipid metabolism; map01100 Metabolic pathways;  map04072 Phospholipase D signaling pathway | | | I | KOG1505 | Lysophosphatidic acid acyltransferase LPAAT and related acyltransferases |
| 22.965 | 36.1 | 7 | 7 | 13 | 0.867 | 0.855 | 0.806 | 1.101 | 1.204 | 1.092 | cytoplasm | K02735 | PSMB3; 20S proteasome subunit beta 3 [EC:3.4.25.1] | map03050 Proteasome | PF00227 | Proteasome subunit | O | KOG0180 | 20S proteasome, regulatory subunit beta type PSMB3/PUP3 |
| 105.72 | 4.1 | 3 | 3 | 3 | 1.606 | 1.573 | 1.838 | 0.732 | 0.691 | 0.68 | nucleus | K13190 | RBM15; RNA-binding protein 15 | | PF00076; PF07744 | RNA recognition motif. (a.k.a. RRM, RBD, or RNP domain); SPOC domain | A | KOG0112 | Large RNA-binding protein (RRM superfamily) |
| 12.454 | 15.6 | 3 | 3 | 9 | 1.335 | 1.277 | 1.341 | 0.817 | 0.77 | 0.722 | cytoplasm | K22138 | MPC1; mitochondrial pyruvate carrier 1 | | | | C | KOG1590 | Uncharacterized conserved protein |
| 20.594 | 8.5 | 2 | 2 | 5 | 0.686 | 0.719 | 0.516 | 1.207 | 1.025 | 1.183 | cytoplasm | K05738 | MYL1; myosin light chain 1 | | |  | Z | KOG0030 | Myosin essential light chain, EF-Hand protein superfamily |
| 8.9723 | 24.7 | 2 | 2 | 3 | 0.776 | 0.741 | 0.92 | 0.992 | 1.123 | 1.15 | cytoplasm | K12158 | NEDD8; ubiquitin-like protein Nedd8 | | PF00240 | Ubiquitin family | DO | KOG0005 | Ubiquitin-like protein |
| 32.066 | 22.4 | 6 | 6 | 9 | 0.915 | 0.738 | 0.843 | 1.282 | 1.272 | 1.202 | cytoplasm | K06911 | K06911; uncharacterized protein | | PF05726 | Pirin C-terminal cupin domain | | |  |
| 34.376 | 19.4 | 6 | 6 | 9 | 1.064 | 1.225 | 1.239 | 0.912 | 0.76 | 0.953 | cytoplasm |  |  |  |  |  |  |  |  |
| 32.823 | 52.5 | 13 | 13 | 26 | 1.206 | 1.373 | 1.388 | 0.796 | 0.878 | 0.76 | cytoplasm | K15101 | SLC25A2_15, ORNT; solute carrier family 25 (mitochondrial ornithine transporter) member 2/15 | | | | C | KOG0763 | Mitochondrial ornithine transporter |
| 45.234 | 40.3 | 16 | 16 | 33 | 0.908 | 0.896 | 0.818 | 1.171 | 1.071 | 1.211 | cytoplasm | K00967 | PCYT2; ethanolamine-phosphate cytidylyltransferase [EC:2.7.7.14] | map00440 Phosphonate and phosphinate metabolism; map00564 Glycerophospholipid metabolism; map01100 Metabolic pathways | PF01467 | Cytidylyltransferase | I | KOG2803 | Choline phosphate cytidylyltransferase/Predicted CDP-ethanolamine synthase |
| 84.921 | 5.8 | 4 | 4 | 4 | 0.884 | 0.852 | 0.81 | 1.168 | 1.11 | 1.152 | endoplasmic reticulum | K13646 | PLOD3; lysyl hydroxylase/galactosyltransferase/glucosyltransferase [EC:1.14.11.4 2.4.1.50 2.4.1.66] | map00310 Lysine degradation; map00514 Other types of O-glycan biosynthesis; map01100 Metabolic pathways | PF03171 | 2OG-Fe(II) oxygenase superfamily | O | KOG1971 | Lysyl hydroxylase |
| 20.732 | 39.2 | 7 | 7 | 15 | 1.239 | 1.094 | 1.348 | 0.879 | 0.874 | 0.869 | cytoplasm | K02882 | RP-L18Ae, RPL18A; large subunit ribosomal protein L18Ae | map03010 Ribosome | PF01775 | Ribosomal L18ae/LX protein domain | J | KOG0829 | 60S ribosomal protein L18A |
| 14.552 | 8.1 | 1 | 1 | 1 | 2.047 | 1.667 | 1.97 | 0.591 | 0.486 | 0.647 | cytoplasm | K02918 | RP-L35e, RPL35; large subunit ribosomal protein L35e | map03010 Ribosome | |  | J | KOG3436 | 60S ribosomal protein L35 |
| 28.78 | 35.6 | 10 | 10 | 15 | 0.827 | 0.819 | 0.811 | 1.116 | 1.189 | 1.18 | mitochondria | K14574 | SDO1, SBDS; ribosome maturation protein SDO1 | map03008 Ribosome biogenesis in eukaryotes | PF09377 | SBDS protein C-terminal domain | J | KOG2917 | Predicted exosome subunit |
| 24.408 | 10.6 | 2 | 2 | 3 | 1.545 | 1.338 | 0.967 | 0.664 | 0.824 | 0.774 | cytoplasm | K07879 | RAB4A, RAB4; Ras-related protein Rab-4A | map04144 Endocytosis | PF00071 | Ras family | U | KOG0086 | GTPase Rab4, small G protein superfamily |
| 25.826 | 16.5 | 3 | 3 | 5 | 1.242 | 1.27 | 1.292 | 0.901 | 0.75 | 0.888 | mitochondria | K17412 | MRPS34; small subunit ribosomal protein S34 | | | |  |  |  |
| 88.812 | 1.6 | 1 | 1 | 1 | 0.366 | 0.393 | 0.363 | 1.078 | 1.187 | 1.16 | extracellular | K06840 | SEMA3; semaphorin 3 | map04360 Axon guidance | | | T | KOG3611 | Semaphorins |
| 10.477 | 19.4 | 2 | 2 | 3 | 0.785 | 0.83 | 0.686 | 1.095 | 1.299 | 1.077 | mitochondria | K23740 |  |  |  |  | S | KOG4023 | Uncharacterized conserved protein |
| 17.319 | 21.1 | 3 | 3 | 7 | 0.844 | 0.809 | 0.866 | 1.07 | 1.164 | 1.054 | nucleus | K03111 | ssb; single-strand DNA-binding protein | map03030 DNA replication; map03430 Mismatch repair; map03440 Homologous recombination | PF00436 | Single-strand binding protein family | L | KOG1653 | Single-stranded DNA-binding protein |
| 33.974 | 45 | 10 | 9 | 26 | 0.859 | 0.875 | 0.767 | 1.076 | 1.228 | 1.11 | cytoplasm | K01014 | SULT1A; aryl sulfotransferase [EC:2.8.2.1] | map05204 Chemical carcinogenesis | PF00685 | Sulfotransferase domain | S | KOG1584 | Sulfotransferase |
| 29.47 | 47.9 | 11 | 11 | 25 | 0.895 | 0.899 | 0.825 | 1.142 | 1.192 | 1.156 | mitochondria | K02728 | PSMA4; 20S proteasome subunit alpha 3 [EC:3.4.25.1] | map03050 Proteasome | PF10584; PF00227 | Proteasome subunit A N-terminal signature; Proteasome subunit | O | KOG0178 | 20S proteasome, regulatory subunit alpha type PSMA4/PRE9 |
| 21.423 | 36.4 | 6 | 6 | 21 | 1.325 | 1.23 | 1.259 | 0.83 | 0.783 | 0.944 | cytoplasm | K02880 | RP-L17e, RPL17; large subunit ribosomal protein L17e | map03010 Ribosome | PF00237 | Ribosomal protein L22p/L17e | J | KOG3353 | 60S ribosomal protein L22 |
| 8.2038 | 47.1 | 4 | 4 | 9 | 1.195 | 1.183 | 1.154 | 0.881 | 0.855 | 0.911 | nucleus | K02923 | RP-L38e, RPL38; large subunit ribosomal protein L38e | map03010 Ribosome | |  | J | KOG3499 | 60S ribosomal protein L38 |
| 33.689 | 5.4 | 2 | 2 | 2 | 1.124 | 1.374 | 1.19 | 0.833 | 0.9 | 0.819 | cytoplasm |  |  |  |  |  | C | KOG1519 | Predicted mitochondrial carrier protein |
| 75.787 | 14.8 | 10 | 10 | 12 | 1.087 | 1.033 | 1.166 | 0.871 | 0.729 | 0.781 | plasma membrane | K14700 | SLC26A1, SAT1; solute carrier family 26 (sulfate anion transporter), member 1 | | PF01740 | STAS domain | P | KOG0236 | Sulfate/bicarbonate/oxalate exchanger SAT-1 and related transporters (SLC26 family) |
| 61.422 | 12.4 | 7 | 7 | 8 | 0.801 | 0.768 | 0.818 | 0.959 | 1.286 | 0.985 | extracellular | K12382 | PSAP, SGP1; saposin | map04142 Lysosome | PF05184 | Saposin-like type B, region 1 | GI | KOG1340 | Prosaposin |
| 52.496 | 16.1 | 8 | 6 | 11 | 1.144 | 1.232 | 1.182 | 0.788 | 0.728 | 0.844 | cytoplasm,nucleus | K14684 | SLC25A23S; solute carrier family 25 (mitochondrial phosphate transporter), member 23/24/25/41 | | PF13499 | EF-hand domain pair | C | KOG0036 | Predicted mitochondrial carrier protein |
| 118.07 | 2.5 | 2 | 2 | 2 | 1.18 | 1.423 | 1.494 | 0.68 | 0.782 | 0.887 | nucleus | K16913 | PELP1, MNAR; proline-, glutamic acid- and leucine-rich protein 1 | | PF08167; PF08166 | rRNA processing/ribosome biogenesis; NUC202 domain | | | |
| 50.239 | 20.5 | 9 | 9 | 12 | 0.784 | 0.77 | 0.783 | 1.388 | 1.254 | 1.371 | nucleus | K01514 | PRUNE, PPX1; exopolyphosphatase [EC:3.6.1.11] | map00230 Purine metabolism; map01100 Metabolic pathways | | | C | KOG4129 | Exopolyphosphatases and related proteins |
| 14.728 | 44.5 | 7 | 2 | 36 | 1.669 | 1.54 | 1.586 | 0.486 | 0.492 | 0.763 | extracellular | K02927 | RP-L40e, RPL40; large subunit ribosomal protein L40e | map03010 Ribosome | PF00240 | Ubiquitin family | J | KOG0003 | Ubiquitin/60s ribosomal protein L40 fusion |
| 13.742 | 36 | 6 | 6 | 18 | 1.182 | 1.184 | 1.262 | 0.864 | 0.826 | 0.916 | nucleus | K02975 | RP-S25e, RPS25; small subunit ribosomal protein S25e | map03010 Ribosome | |  | J | KOG1767 | 40S ribosomal protein S25 |
| 69.613 | 4.2 | 2 | 2 | 6 | 1.26 | 1.418 | 1.367 | 0.8 | 0.542 | 0.598 | plasma membrane | K05045 | SLC6A12, BGT1; solute carrier family 6 (neurotransmitter transporter, betaine/GABA) member 12 | map04721 Synaptic vesicle cycle; map04727 GABAergic synapse | | | P | KOG3660 | Sodium-neurotransmitter symporter |
| 564.81 | 0.2 | 1 | 1 | 1 | 0.62 | 0.544 | 0.57 | 1.108 | 1.14 | 1.077 | plasma membrane | K04962 | RYR2; ryanodine receptor 2 | map04020 Calcium signaling pathway; map04024 cAMP signaling pathway; map04260 Cardiac muscle contraction; map04261 Adrenergic signaling in cardiomyocytes; map04371 Apelin signaling pathway; map04713 Circadian entrainment; map04911 Insulin secretion; map04921 Oxytocin signaling pathway; map04972 Pancreatic secretion; map05410 Hypertrophic cardiomyopathy (HCM); map05412 Arrhythmogenic right ventricular cardiomyopathy (ARVC); map05414 Dilated cardiomyopathy (DCM) | PF08709; PF02815; PF13833 | Inositol 1,4,5-trisphosphate/ryanodine receptor; MIR domain; EF-hand domain pair | T | KOG2243 | Ca2+ release channel (ryanodine receptor) |
| 204.1 | 5.3 | 9 | 9 | 9 | 1.264 | 1.127 | 1.245 | 0.612 | 0.978 | 0.668 | plasma membrane | K14314 | NUP210, GP210; nuclear pore complex protein Nup210 | map03013 RNA transport | PF02368 | Bacterial Ig-like domain (group 2) | U | KOG1833 | Nuclear pore complex, gp210 component |
| 94.371 | 6.8 | 5 | 5 | 7 | 0.78 | 0.736 | 0.894 | 0.95 | 1.044 | 1.168 | nucleus | K17552 | PPP1R10; protein phosphatase 1 regulatory subunit 10 | | PF08711 | TFIIS helical bundle-like domain | | |  |
| 14.467 | 9.8 | 1 | 1 | 2 | 1.554 | 1.778 | 1.614 | 0.56 | 0.698 | 0.616 | nucleus | K02891 | RP-L22e, RPL22; large subunit ribosomal protein L22e | map03010 Ribosome | |  | J | KOG3434 | 60S ribosomal protein L22 |
| 15.733 | 44.5 | 8 | 8 | 13 | 1.199 | 1.072 | 1.223 | 0.876 | 0.773 | 0.956 | mitochondria | K02903 | RP-L28e, RPL28; large subunit ribosomal protein L28e | map03010 Ribosome | |  | J | KOG3412 | 60S ribosomal protein L28 |
| 45.775 | 1.7 | 1 | 1 | 1 | 0.958 | 0.758 | 0.812 | 1.389 | 1.188 | 1.095 | mitochondria | K23430 | SERPINI2; serpin I2 | | PF00079 | Serpin (serine protease inhibitor) | V | KOG2392 | Serpin |
| 82.988 | 45.1 | 29 | 29 | 49 | 1.102 | 1.129 | 1.201 | 0.861 | 0.888 | 0.858 | mitochondria | K17800 | LETM1, MDM38; LETM1 and EF-hand domain-containing protein 1, mitochondrial | | | | S | KOG1043 | Ca2+-binding transmembrane protein LETM1/MRS7 |
| 26.411 | 52.3 | 10 | 10 | 34 | 0.885 | 0.879 | 0.896 | 1.108 | 1.305 | 1.14 | cytoplasm | K02729 | PSMA5; 20S proteasome subunit alpha 5 [EC:3.4.25.1] | map03050 Proteasome | PF10584; PF00227 | Proteasome subunit A N-terminal signature; Proteasome subunit | O | KOG0176 | 20S proteasome, regulatory subunit alpha type PSMA5/PUP2 |
| 123.81 | 2.2 | 2 | 2 | 2 | 1.258 | 1.101 | 1.188 | 0.849 | 0.815 | 0.969 | nucleus | K12236 | NFX1; transcriptional repressor NF-X1 | map05165 Human papillomavirus infection | PF01424 | R3H domain | K | KOG1952 | Transcription factor NF-X1, contains NFX-type Zn2+-binding and R3H domains |
| 106.72 | 8.7 | 6 | 6 | 7 | 0.84 | 0.782 | 0.864 | 1.142 | 1.083 | 1.096 | cytoplasm | K14301 | NUP107, NUP84; nuclear pore complex protein Nup107 | map03013 RNA transport | | | UY | KOG1964 | Nuclear pore complex, rNup107 component (sc Nup84) |
| 35.774 | 37 | 9 | 9 | 15 | 0.816 | 0.936 | 0.811 | 1.111 | 1.301 | 1.084 | cytoplasm,nucleus | K23353 | PDLIM1_2_3_4; PDZ and LIM domain protein 1/2/3/4 | | PF00595; PF00412 | PDZ domain (Also known as DHR or GLGF); LIM domain | TZ | KOG1703 | Adaptor protein Enigma and related PDZ-LIM proteins |
| 36.807 | 19 | 4 | 4 | 5 | 1.806 | 2.006 | 1.7 | 0.62 | 0.659 | 0.607 | extracellular | K14379 | ACP5; tartrate-resistant acid phosphatase type 5 [EC:3.1.3.2] | map00740 Riboflavin metabolism; map01100 Metabolic pathways; map04142 Lysosome; map04380 Osteoclast differentiation; map05323 Rheumatoid arthritis | PF00149 | Calcineurin-like phosphoesterase | O | KOG2679 | Purple (tartrate-resistant) acid phosphatase |
| 14.544 | 12.2 | 2 | 2 | 2 | 1.151 | 1.163 | 1.145 | 0.833 | 0.891 | 0.926 | nucleus | K17553 | PPP1R11; protein phosphatase 1 regulatory subunit 11 | | | | S | KOG4102 | Uncharacterized conserved protein |
| 23.553 | 19.4 | 4 | 2 | 4 | 1.132 | 1.164 | 1.203 | 1.002 | 0.811 | 0.714 | cytoplasm | K07834 | RALA; Ras-related protein Ral-A | map04014 Ras signaling pathway; map04015 Rap1 signaling pathway; map04072 Phospholipase D signaling pathway; map05200 Pathways in cancer; map05210 Colorectal cancer; map05212 Pancreatic cancer | PF00071 | Ras family | U | KOG0395 | Ras-related GTPase |
| 7.4454 | 50 | 3 | 3 | 8 | 1.342 | 1.262 | 1.324 | 0.753 | 0.81 | 0.881 | mitochondria | K00419 | QCR9, UCRC; ubiquinol-cytochrome c reductase subunit 9 | map00190 Oxidative phosphorylation; map01100 Metabolic pathways; map04260 Cardiac muscle contraction; map04714 Thermogenesis;  map04932 Non-alcoholic fatty liver disease (NAFLD); map05010 Alzheimer disease; map05012 Parkinson disease; map05016 Huntington disease | | | C | KOG3494 | Ubiquinol cytochrome c oxidoreductase, subunit QCR9 |
| 17.222 | 59.6 | 9 | 9 | 19 | 1.225 | 1.251 | 1.306 | 0.859 | 0.8 | 0.94 | cytoplasm | K02953 | RP-S13e, RPS13; small subunit ribosomal protein S13e | map03010 Ribosome | PF08069; PF00312 | Ribosomal S13/S15 N-terminal domain; Ribosomal protein S15 | J | KOG0400 | 40S ribosomal protein S13 |
| 31.617 | 11.3 | 4 | 4 | 6 | 1.437 | 1.653 | 1.678 | 0.746 | 0.74 | 0.696 | mitochondria | K07152 | SCO1; protein SCO1 | |  |  | C | KOG2792 | Putative cytochrome C oxidase assembly protein |
| 62.289 | 2.2 | 1 | 1 | 1 | 1.27 | 1.361 | 1.998 | 0.825 | 0.774 | 0.956 | plasma membrane | K08202 | SLC22A4_5, OCTN; MFS transporter, OCT family, solute carrier family 22 (organic cation transporter), member 4/5 | map05231 Choline metabolism in cancer | | | S | KOG0255 | Synaptic vesicle transporter SVOP and related transporters (major facilitator superfamily) |
| 95.012 | 18 | 12 | 12 | 14 | 0.866 | 0.855 | 0.85 | 1.181 | 1.28 | 1.113 | cytoplasm,nucleus | K15631 | ABA3; molybdenum cofactor sulfurtransferase [EC:2.8.1.9] | map00790 Folate biosynthesis | PF00266; PF03476; PF03473 | Aminotransferase class-V; MOSC N-terminal beta barrel domain; MOSC domain | H | KOG2142 | Molybdenum cofactor sulfurase |
| 32.355 | 35.1 | 8 | 8 | 10 | 0.768 | 0.716 | 0.879 | 1.184 | 1.114 | 1.068 | cytoplasm | K06210 | NMNAT; nicotinamide mononucleotide adenylyltransferase [EC:2.7.7.1 2.7.7.18] | map00760 Nicotinate and nicotinamide metabolism; map01100 Metabolic pathways | PF01467 | Cytidylyltransferase | H | KOG3199 | Nicotinamide mononucleotide adenylyl transferase |
| 84.998 | 13.7 | 8 | 8 | 11 | 1.066 | 1.336 | 1.228 | 0.529 | 0.943 | 0.566 | peroxisome | K13073 | PIGR; polymeric immunoglobulin receptor | map04672 Intestinal immune network for IgA production | PF07686 | Immunoglobulin V-set domain | | |  |
| 34.577 | 28.1 | 9 | 9 | 15 | 0.859 | 0.836 | 0.821 | 1.111 | 1.097 | 1.175 | cytoplasm | K03030 | PSMD14, RPN11, POH1; 26S proteasome regulatory subunit N11 | map03050 Proteasome; map05169 Epstein-Barr virus infection | | | O | KOG1555 | 26S proteasome regulatory complex, subunit RPN11 |
| 59.613 | 1.5 | 1 | 1 | 1 | 1.365 | 1.749 | 1.68 | 0.54 | 0.473 | 0.703 | plasma membrane | K08204 | SLC22A7, OAT2; MFS transporter, OCT family, solute carrier family 22 (organic anion transporter), member 7 | map04976 Bile secretion | |  | S | KOG0255 | Synaptic vesicle transporter SVOP and related transporters (major facilitator superfamily) |
| 117.33 | 1 | 1 | 1 | 1 | 0.343 | 0.49 | 0.302 | 1.907 | 0.772 | 1.517 | plasma membrane | |  |  | PF13589; PF07496 | Histidine kinase-, DNA gyrase B-, and HSP90-like ATPase; CW-type Zinc Finger | D | KOG1845 | MORC family ATPases |
| 60.024 | 4.2 | 1 | 1 | 1 | 0.984 | 1.206 | 1.319 | 0.674 | 0.818 | 0.859 | plasma membrane | K08231 | SLC16A2; MFS transporter, MCT family, solute carrier family 16 (monocarboxylic acid transporters), member 2 | map04919 Thyroid hormone signaling pathway | | | G | KOG2504 | Monocarboxylate transporter |
| 20.208 | 9.9 | 2 | 2 | 3 | 1.238 | 1.283 | 1.234 | 0.967 | 0.864 | 0.966 | mitochondria | K02948 | RP-S11, MRPS11, rpsK; small subunit ribosomal protein S11 | map03010 Ribosome | |  | J | KOG0408 | Mitochondrial/chloroplast ribosomal protein S11 |
| 10.294 | 49.4 | 2 | 2 | 3 | 0.58 | 0.768 | 0.958 | 1.388 | 1.588 | 1.351 | cytoplasm | K21127 | S100A8; protein S100-A8 | map04657 IL-17 signaling pathway | PF01023 | S-100/ICaBP type calcium binding domain | | | |
| 6.954 | 19.3 | 1 | 1 | 3 | 1.502 | 1.445 | 1.544 | 0.712 | 0.682 | 0.778 | cytoplasm | K03957 | NDUFB1; NADH dehydrogenase (ubiquinone) 1 beta subcomplex subunit 1 | map00190 Oxidative phosphorylation; map01100 Metabolic pathways; map04714 Thermogenesis; map04723 Retrograde endocannabinoid signaling;  map04932 Non-alcoholic fatty liver disease (NAFLD); map05010 Alzheimer disease; map05012 Parkinson disease; map05016 Huntington disease | | | | | |
| 17.982 | 26.5 | 5 | 5 | 8 | 0.662 | 0.654 | 0.606 | 1.185 | 1.03 | 1.185 | extracellular |  |  |  |  |  |  |  |  |
| 73.697 | 8.8 | 7 | 7 | 8 | 1.324 | 1.251 | 1.235 | 0.988 | 0.975 | 0.912 | nucleus |  |  |  | PF05022 | SRP40, C-terminal domain | Y | KOG2992 | Nucleolar GTPase/ATPase p130 |
| 34.727 | 18.3 | 5 | 5 | 6 | 1.324 | 1.425 | 1.336 | 0.754 | 0.927 | 0.883 | mitochondria | |  |  | PF00175 | Oxidoreductase NAD-binding domain | CH | KOG0534 | NADH-cytochrome b-5 reductase |
| 37.67 | 16.7 | 5 | 5 | 6 | 1.394 | 1.421 | 1.334 | 0.924 | 0.591 | 0.815 | cytoplasm | K14945 | QKI; protein quaking | | PF00013 | KH domain | K | KOG1588 | RNA-binding protein Sam68 and related KH domain proteins |
| 10.854 | 23.1 | 2 | 2 | 2 | 1.457 | 1.41 | 1.44 | 0.772 | 0.699 | 0.763 | cytoplasm | K22069 | LYRM4; LYR motif-containing protein 4 | | | | A | KOG3801 | Uncharacterized conserved protein BCN92 |
| 63.953 | 38.1 | 19 | 19 | 85 | 0.753 | 0.691 | 0.494 | 1.658 | 1.234 | 1.635 | nucleus | K00029 | E1.1.1.40, maeB; malate dehydrogenase (oxaloacetate-decarboxylating)(NADP+) [EC:1.1.1.40] | map00620 Pyruvate metabolism; map01100 Metabolic pathways; map01200 Carbon metabolism; map03320 PPAR signaling pathway | PF00390; PF03949 | Malic enzyme, N-terminal domain; Malic enzyme, NAD binding domain | C | KOG1257 | NADP+-dependent malic enzyme |
| 66.264 | 1.7 | 1 | 1 | 1 | 0.834 | 0.746 | 0.775 | 1.198 | 0.985 | 1.311 | plasma membrane | K03860 | PIGQ, GPI1; phosphatidylinositol N-acetylglucosaminyltransferase subunit Q | map00563 Glycosylphosphatidylinositol (GPI)-anchor biosynthesis; map01100 Metabolic pathways | | | MO | KOG1183 | N-acetylglucosaminyltransferase complex, subunit PIG-Q/GPI1, required for phosphatidylinositol biosynthesis |
| 6.5386 | 21.4 | 1 | 1 | 6 | 1.433 | 1.49 | 1.383 | 0.784 | 0.713 | 0.697 | mitochondria | K00420 | QCR10, UQCR; ubiquinol-cytochrome c reductase subunit 10 | map00190 Oxidative phosphorylation; map01100 Metabolic pathways; map04260 Cardiac muscle contraction; map04714 Thermogenesis; map04932 Non-alcoholic fatty liver disease (NAFLD); map05010 Alzheimer disease; map05012 Parkinson disease; map05016 Huntington disease | PF08997 | Ubiquinol-cytochrome C reductase complex, 6.4kD protein | | | |
| 16.604 | 13.7 | 2 | 2 | 2 | 0.675 | 0.79 | 0.582 | 0.996 | 1.341 | 1.128 | nucleus | K13186 | RBM3; RNA-binding protein 3 | | PF00076 | RNA recognition motif. (a.k.a. RRM, RBD, or RNP domain) | A | KOG0118 | FOG: RRM domain |
| 91.83 | 17 | 11 | 11 | 17 | 1.113 | 1.199 | 1.208 | 0.806 | 1.051 | 0.741 | plasma membrane | K01228 | MOGS; mannosyl-oligosaccharide glucosidase [EC:3.2.1.106] | map00510 N-Glycan biosynthesis; map01100 Metabolic pathways; map04141 Protein processing in endoplasmic reticulum | PF03200 | Mannosyl oligosaccharide glucosidase | G | KOG2161 | Glucosidase I |
| 15.081 | 36.4 | 3 | 3 | 9 | 1.252 | 1.398 | 1.449 | 0.768 | 0.8 | 0.81 | cytoplasm,nucleus | K03960 | NDUFB4; NADH dehydrogenase (ubiquinone) 1 beta subcomplex subunit 4 | map00190 Oxidative phosphorylation; map01100 Metabolic pathways; map04714 Thermogenesis; map04723 Retrograde endocannabinoid signaling;  map04932 Non-alcoholic fatty liver disease (NAFLD); map05010 Alzheimer disease; map05012 Parkinson disease; map05016 Huntington disease | | | | | |
| 39.413 | 3.9 | 1 | 1 | 2 | 1.14 | 1.122 | 1.236 | 0.952 | 0.668 | 0.697 | plasma membrane | K14341 | SLC10A1, NTCP; solute carrier family 10 (sodium/bile acid cotransporter), member 1 | map04976 Bile secretion; map05161 Hepatitis B | | | P | KOG2718 | Na+-bile acid cotransporter |
| 31.802 | 10.1 | 2 | 2 | 2 | 1.311 | 1.266 | 1.311 | 0.852 | 0.819 | 0.991 | extracellular |  |  |  | PF03803 | Scramblase | M | KOG0621 | Phospholipid scramblase |
| 14.454 | 17.3 | 2 | 2 | 2 | 1.779 | 1.879 | 1.828 | 0.655 | 0.599 | 0.635 | cytoplasm | K04798 | pfdB, PFDN6; prefoldin beta subunit | | |  | O | KOG3478 | Prefoldin subunit 6, KE2 family |
| 90.028 | 2.8 | 2 | 2 | 2 | 0.769 | 0.817 | 0.83 | 1.051 | 1.083 | 1.133 | plasma membrane | K17568 | ELFN2; protein phosphatase 1 regulatory subunit 29 | | | | |  |  |
| 17.695 | 37.8 | 8 | 8 | 18 | 1.306 | 1.282 | 1.225 | 0.873 | 0.795 | 0.905 | cytoplasm | K02893 | RP-L23Ae, RPL23A; large subunit ribosomal protein L23Ae | map03010 Ribosome | |  | J | KOG1751 | 60s ribosomal protein L23 |
| 38.194 | 36.1 | 13 | 12 | 43 | 1.169 | 0.959 | 1.209 | 0.85 | 0.895 | 0.781 | mitochondria | |  |  | PF03476; PF03473 | MOSC N-terminal beta barrel domain; MOSC domain | E | KOG2362 | Uncharacterized Fe-S protein |
| 17.551 | 23.2 | 4 | 4 | 47 | 1.324 | 1.308 | 1.262 | 0.786 | 0.874 | 0.734 | mitochondria | K00799 | GST, gst; glutathione S-transferase [EC:2.5.1.18] | map00480 Glutathione metabolism; map00980 Metabolism of xenobiotics by cytochrome P450; map00982 Drug metabolism - cytochrome P450;  map00983 Drug metabolism - other enzymes; map01100 Metabolic pathways; map01524 Platinum drug resistance;  map04212 Longevity regulating pathway - worm; map05200 Pathways in cancer; map05204 Chemical carcinogenesis;  map05225 Hepatocellular carcinoma; map05418 Fluid shear stress and atherosclerosis | | | | | |
| 46.646 | 42.4 | 17 | 17 | 38 | 0.509 | 0.446 | 0.775 | 2.314 | 1.451 | 1.308 | cytoplasm,nucleus | K17284 | PLIN2, ADRP; perilipin-2 | map03320 PPAR signaling pathway | | |  |  |  |
| 40.742 | 47.8 | 19 | 18 | 36 | 0.854 | 0.837 | 0.792 | 1.096 | 1.161 | 1.23 | cytoplasm | K05864 | PPID, CYPD; peptidyl-prolyl isomerase D [EC:5.2.1.8] | map04217 Necroptosis; map04218 Cellular senescence; map05131 Shigellosis | PF00160 | Cyclophilin type peptidyl-prolyl cis-trans isomerase/CLD | O | KOG0546 | HSP90 co-chaperone CPR7/Cyclophilin |
| 199.59 | 4.2 | 7 | 7 | 7 | 1.282 | 1.324 | 1.173 | 0.893 | 0.815 | 0.91 | nucleus | K10624 | RBBP6; E3 ubiquitin-protein ligase RBBP6 [EC:2.3.2.27] | | PF08783; PF13696; PF04564 | DWNN domain; Zinc knuckle; U-box domain | O | KOG0314 | Predicted E3 ubiquitin ligase |
| 117.43 | 23.3 | 20 | 20 | 26 | 0.93 | 0.871 | 0.882 | 1.251 | 1.225 | 1.14 | nucleus | K10431 | MAP4; microtubule-associated protein 4 | | | | Z | KOG2418 | Microtubule-associated protein TAU |
| 95.781 | 1.8 | 2 | 2 | 2 | 0.648 | 0.761 | 0.556 | 1.148 | 0.885 | 1.528 | nucleus | K17334 | NFATC4, NFAT3; nuclear factor of activated T-cells, cytoplasmic 4 | map04022 cGMP-PKG signaling pathway; map04218 Cellular senescence; map04310 Wnt signaling pathway; map04360 Axon guidance; map04625 C-type lectin receptor signaling pathway; map04921 Oxytocin signaling pathway; map05161 Hepatitis B; map05163 Human cytomegalovirus infection; map05166 Human T-cell leukemia virus 1 infection; map05167 Kaposi sarcoma-associated herpesvirus infection; map05170 Human immunodeficiency virus 1 infection | PF00554 | Rel homology domain (RHD) | | |  |
| 38.677 | 15.2 | 5 | 5 | 8 | 0.899 | 0.973 | 0.924 | 1.285 | 1.431 | 1.058 | cytoplasm | K13335 | PEX16; peroxin-16 | map04146 Peroxisome | |  | U | KOG4546 | Peroxisomal biogenesis protein (peroxin 16) |
| 73.976 | 3.2 | 2 | 2 | 4 | 0.766 | 0.864 | 0.653 | 1.626 | 1.353 | 1.493 | extracellular | K23460 | CHM, CHML; Rab proteins geranylgeranyltransferase component A | | | | O | KOG4405 | GDP dissociation inhibitor |
| 65.514 | 7.2 | 4 | 3 | 4 | 0.766 | 0.712 | 0.714 | 1.167 | 1.246 | 1.293 | peroxisome | K01937 | pyrG, CTPS; CTP synthase [EC:6.3.4.2] | map00240 Pyrimidine metabolism; map01100 Metabolic pathways | PF06418; PF00117 | CTP synthase N-terminus; Glutamine amidotransferase class-I | F | KOG2387 | CTP synthase (UTP-ammonia lyase) |
| 23.466 | 22.4 | 5 | 5 | 7 | 1.45 | 1.129 | 1.403 | 0.744 | 0.739 | 0.862 | cytoplasm | K02885 | RP-L19e, RPL19; large subunit ribosomal protein L19e | map03010 Ribosome | |  | J | KOG1696 | 60s ribosomal protein L19 |
| 34.468 | 15.9 | 4 | 4 | 5 | 0.723 | 0.88 | 0.961 | 1.231 | 1.119 | 1.245 | cytoplasm | K15523 | FN3KRP; protein-ribulosamine 3-kinase [EC:2.7.1.172] | | | | G | KOG3021 | Predicted kinase |
| 104.58 | 2.1 | 2 | 2 | 2 | 1.246 | 1.127 | 1.177 | 0.811 | 0.958 | 0.696 | plasma membrane | K05294 | PGAP1; GPI inositol-deacylase [EC:3.-.-.-] | map00563 Glycosylphosphatidylinositol (GPI)-anchor biosynthesis; map01100 Metabolic pathways | | | U | KOG3724 | Negative regulator of COPII vesicle formation |
| 11.952 | 12.7 | 1 | 1 | 1 | 1.218 | 1.211 | 1.235 | 0.863 | 0.803 | 0.911 | mitochondria | |  |  |  |  |  |  |  |
| 147.13 | 0.6 | 1 | 1 | 1 | 0.768 | 0.697 | 0.703 | 1.049 | 0.899 | 0.906 | plasma membrane | K14461 | NPC1L1; Niemann-Pick C1-like protein 1 | map04975 Fat digestion and absorption | | | I | KOG1933 | Cholesterol transport protein (Niemann-Pick C disease protein) |
| 23.984 | 20.2 | 4 | 4 | 6 | 0.674 | 0.706 | 0.813 | 1.136 | 1.35 | 1.055 | nucleus | K13987 | NUDT5; ADP-sugar pyrophosphatase / 8-oxo-dGDP phosphatase / ADP-D-ribose pyrophosphorylase [EC:3.6.1.13 3.6.1.58 2.7.7.96] | map00230 Purine metabolism; map01100 Metabolic pathways | PF00293 | NUDIX domain | L | KOG3041 | Nucleoside diphosphate-sugar hydrolase of the MutT (NUDIX) family |
| 203.8 | 3.8 | 7 | 7 | 8 | 0.887 | 0.887 | 0.843 | 1.174 | 1.267 | 0.986 | cytoplasm | K15261 | PARP10_14_15; poly [ADP-ribose] polymerase 10/14/15 [EC:2.4.2.30] | | PF01661 | Macro domain | BK | KOG2633 | Hismacro and SEC14 domain-containing proteins |
| 55.238 | 19.6 | 8 | 8 | 11 | 0.82 | 0.725 | 0.852 | 1.21 | 0.968 | 1.066 | cytoplasm,nucleus | K10599 | PRPF19, PRP19; pre-mRNA-processing factor 19 [EC:2.3.2.27] | map03040 Spliceosome; map04120 Ubiquitin mediated proteolysis | PF04564; PF08606 | U-box domain; Prp19/Pso4-like | S | KOG0289 | mRNA splicing factor |
| 25.957 | 39.3 | 9 | 7 | 22 | 0.932 | 0.787 | 0.891 | 1.153 | 1.413 | 1.218 | extracellular | K03991 | MBL; mannose-binding lectin | map04145 Phagosome; map04610 Complement and coagulation cascades; map05150 Staphylococcus aureus infection | PF00059 | Lectin C-type domain | W | KOG4297 | C-type lectin |
| 33.499 | 26.4 | 7 | 7 | 14 | 1.194 | 1.26 | 1.22 | 0.82 | 0.953 | 0.759 | extracellular | K17885 | MTCH; mitochondrial carrier | | |  | C | KOG2745 | Mitochondrial carrier protein |
| 34.772 | 3.8 | 1 | 1 | 1 | 1.636 | 1.547 | 1.21 | 0.733 | 0.876 | 0.707 | peroxisome | K07750 | MESO1, ERG25; methylsterol monooxygenase [EC:1.14.18.9] | map00100 Steroid biosynthesis; map01100 Metabolic pathways | | | I | KOG0873 | C-4 sterol methyl oxidase |
| 18.314 | 21.7 | 3 | 3 | 3 | 1.168 | 1.145 | 1.058 | 0.853 | 0.908 | 0.726 | cytoplasm,nucleus | K17981 | MTFP1, MTP18; mitochondrial fission process protein 1 | | | | S | KOG3945 | Uncharacterized conserved protein |
| 15.515 | 35.9 | 5 | 5 | 11 | 1.176 | 1.192 | 1.217 | 0.826 | 0.892 | 0.778 | cytoplasm | K03962 | NDUFB6; NADH dehydrogenase (ubiquinone) 1 beta subcomplex subunit 6 | map00190 Oxidative phosphorylation; map04714 Thermogenesis; map04723 Retrograde endocannabinoid signaling; map04932 Non-alcoholic fatty liver  disease (NAFLD); map05010 Alzheimer disease; map05012 Parkinson disease; map05016 Huntington disease | | | C | KOG4633 | NADH:ubiquinone oxidoreductase, NDUFB6/B17 subunit |
| 39.218 | 35.5 | 11 | 11 | 31 | 0.956 | 0.812 | 0.733 | 1.17 | 1.174 | 1.052 | cytoplasm | K07048 | PTER, php; phosphotriesterase-related protein | | PF02126 | Phosphotriesterase family | | |  |
| 23.464 | 31.5 | 7 | 7 | 17 | 1.227 | 1.196 | 1.234 | 0.848 | 0.677 | 0.845 | cytoplasm | K02872 | RP-L13Ae, RPL13A; large subunit ribosomal protein L13Ae | map03010 Ribosome | |  | J | KOG3204 | 60S ribosomal protein L13a |
| 15.798 | 37.5 | 5 | 5 | 14 | 1.403 | 1.192 | 1.342 | 0.736 | 0.616 | 0.849 | mitochondria | K02901 | RP-L27e, RPL27; large subunit ribosomal protein L27e | map03010 Ribosome | |  | J | KOG3418 | 60S ribosomal protein L27 |
| 14.866 | 31.9 | 4 | 4 | 8 | 0.751 | 0.619 | 0.588 | 1.277 | 1.257 | 1.331 | extracellular | K06830 | LGALS1; galectin-1 | | PF00337 | Galactoside-binding lectin | W | KOG3587 | Galectin, galactose-binding lectin |
| 13.219 | 13 | 1 | 1 | 3 | 1.606 | 1.478 | 1.613 | 0.544 | 0.737 | 0.692 | extracellular | K03880 | ND3; NADH-ubiquinone oxidoreductase chain 3 [EC:7.1.1.2] | map00190 Oxidative phosphorylation; map01100 Metabolic pathways; map04714 Thermogenesis; map04723 Retrograde endocannabinoid signaling;  map05012 Parkinson disease | | | C | KOG4662 | NADH dehydrogenase subunit 3 and related proteins |
| 104.55 | 9.1 | 6 | 6 | 7 | 0.805 | 0.973 | 0.81 | 1.44 | 1.226 | 1.22 | extracellular | K13339 | PEX6, PXAAA1; peroxin-6 | map04146 Peroxisome | PF00004 | ATPase family associated with various cellular activities (AAA) | O | KOG0736 | Peroxisome assembly factor 2 containing the AAA+-type ATPase domain |
| 13.527 | 37.8 | 7 | 7 | 20 | 1.28 | 1.276 | 1.33 | 0.867 | 0.825 | 0.86 | nucleus | K00417 | QCR7, UQCRB; ubiquinol-cytochrome c reductase subunit 7 | map00190 Oxidative phosphorylation; map01100 Metabolic pathways; map04260 Cardiac muscle contraction; map04714 Thermogenesis;  map04932 Non-alcoholic fatty liver disease (NAFLD); map05010 Alzheimer disease; map05012 Parkinson disease; map05016 Huntington disease | | | C | KOG3440 | Ubiquinol cytochrome c reductase, subunit QCR7 |
| 11.845 | 11.8 | 1 | 1 | 2 | 1.35 | 1.101 | 1.328 | 0.903 | 1.037 | 0.858 | cytoskeleton | K12622 | LSM3; U6 snRNA-associated Sm-like protein LSm3 | map03018 RNA degradation; map03040 Spliceosome | PF01423 | LSM domain | A | KOG3460 | Small nuclear ribonucleoprotein (snRNP) LSM3 |
| 79.996 | 3.3 | 2 | 2 | 2 | 1.097 | 1.434 | 1.144 | 0.803 | 0.993 | 0.92 | plasma membrane | |  |  |  |  |  |  |  |
| 75.516 | 4.4 | 3 | 3 | 3 | 1.318 | 1.18 | 1.154 | 0.926 | 0.766 | 0.906 | extracellular | K03993 | MASP2; mannan-binding lectin serine protease 2 [EC:3.4.21.104] | map04610 Complement and coagulation cascades; map05150 Staphylococcus aureus infection | PF00431; PF07645; PF00084; PF00089 | CUB domain; Calcium-binding EGF domain; Sushi domain (SCR repeat); Trypsin | E | KOG3627 | Trypsin |
| 25.493 | 16.7 | 3 | 3 | 3 | 0.9 | 0.736 | 0.87 | 1.483 | 1.047 | 1.131 | cytoplasm |  |  |  |  |  | J | KOG3942 | MIF4G domain-containing protein |
| 8.5669 | 10.5 | 1 | 1 | 3 | 1.392 | 1.442 | 1.38 | 0.636 | 0.568 | 0.761 | mitochondria | K17784 | MINOS1, MOS1; mitochondrial inner membrane organizing system protein 1 | | PF04418 | Domain of unknown function (DUF543) | S | KOG4604 | Uncharacterized conserved protein |
| 95.923 | 51.7 | 35 | 35 | 63 | 0.742 | 0.66 | 0.652 | 1.31 | 1.47 | 1.172 | cytoplasm | K17266 | MVP; major vault protein | | PF01505; PF11978 | Major Vault Protein repeat; Shoulder domain | | | |
| 10.435 | 74.2 | 6 | 6 | 9 | 1.404 | 1.47 | 1.369 | 0.621 | 0.706 | 0.847 | extracellular | K00416 | QCR6, UQCRH; ubiquinol-cytochrome c reductase subunit 6 | map00190 Oxidative phosphorylation; map01100 Metabolic pathways; map04260 Cardiac muscle contraction; map04714 Thermogenesis;  map04932 Non-alcoholic fatty liver disease (NAFLD); map05010 Alzheimer disease; map05012 Parkinson disease; map05016 Huntington disease | | | C | KOG4763 | Ubiquinol-cytochrome c reductase hinge protein |
| 87.365 | 40.1 | 26 | 26 | 47 | 0.844 | 0.927 | 0.746 | 1.456 | 1.192 | 1.205 | nucleus | K11730 | ACAD11; acyl-CoA dehydrogenase family member 11 | | PF02771; PF02770; PF00441 | Acyl-CoA dehydrogenase, N-terminal domain; Acyl-CoA dehydrogenase, middle domain; Acyl-CoA dehydrogenase, C-terminal domain | I | KOG1469 | Predicted acyl-CoA dehydrogenase |
| 51.691 | 18.3 | 8 | 2 | 8 | 0.817 | 0.971 | 0.838 | 1.078 | 1.333 | 1.239 | cytoplasm | K04354 | PPP2R2; serine/threonine-protein phosphatase 2A regulatory subunit B | map03015 mRNA surveillance pathway; map04071 Sphingolipid signaling pathway; map04151 PI3K-Akt signaling pathway;  map04152 AMPK signaling pathway; map04261 Adrenergic signaling in cardiomyocytes; map04390 Hippo signaling pathway;  map04391 Hippo signaling pathway - fly; map04530 Tight junction; map04728 Dopaminergic synapse; map05142 Chagas disease  (American trypanosomiasis); map05160 Hepatitis C; map05165 Human papillomavirus infection | | | T | KOG1354 | Serine/threonine protein phosphatase 2A, regulatory subunit |
| 41.892 | 39.1 | 12 | 7 | 41 | 1.689 | 1.442 | 1.597 | 0.567 | 0.543 | 0.499 | cytoplasm | K00070 | HSD3B; 3beta-hydroxy-Delta5-steroid dehydrogenase / steroid Delta-isomerase [EC:1.1.1.145 5.3.3.1] | map00140 Steroid hormone biosynthesis; map01100 Metabolic pathways; map04913 Ovarian steroidogenesis; map04925 Aldosterone synthesis  and secretion; map04927 Cortisol synthesis and secretion; map04934 Cushing syndrome | | | EI | KOG1430 | C-3 sterol dehydrogenase/3-beta-hydroxysteroid dehydrogenase and related dehydrogenases |
| 35.194 | 35.2 | 10 | 10 | 23 | 1.06 | 0.976 | 1.132 | 0.834 | 0.861 | 0.738 | plasma membrane | K01540 | ATP1B, CD298; sodium/potassium-transporting ATPase subunit beta | map04022 cGMP-PKG signaling pathway; map04024 cAMP signaling pathway; map04260 Cardiac muscle contraction;  map04261 Adrenergic signaling in cardiomyocytes; map04911 Insulin secretion; map04918 Thyroid hormone synthesis;  map04919 Thyroid hormone signaling pathway; map04925 Aldosterone synthesis and secretion; map04960 Aldosterone-regulated sodium reabsorption;  map04961 Endocrine and other factor-regulated calcium reabsorption; map04964 Proximal tubule bicarbonate reclamation; map04970 Salivary secretion;  map04971 Gastric acid secretion; map04972 Pancreatic secretion; map04973 Carbohydrate digestion and absorption;  map04974 Protein digestion and absorption; map04976 Bile secretion; map04978 Mineral absorption | | | P | KOG3927 | Na+/K+ ATPase, beta subunit |
| 121.04 | 6.8 | 6 | 6 | 7 | 0.872 | 0.89 | 0.822 | 1.166 | 1.215 | 1.177 | nucleus | K23390 | BAG6; large proline-rich protein BAG6 | | PF00240; PF12057 | Ubiquitin family; Domain of unknown function (DUF3538) | O | KOG4248 | Ubiquitin-like protein, regulator of apoptosis |
| 24.936 | 21 | 4 | 4 | 6 | 1.242 | 1.362 | 1.297 | 0.86 | 0.918 | 0.843 | nucleus | K12194 | CHMP4, SNF7, VPS32; charged multivesicular body protein 4 | map04144 Endocytosis; map04217 Necroptosis | | | U | KOG1656 | Protein involved in glucose derepression and pre-vacuolar endosome protein sorting |
| 73.963 | 2.5 | 2 | 2 | 2 | 1.384 | 1.328 | 1.262 | 0.744 | 0.557 | 0.652 | mitochondria | K01425 | glsA, GLS; glutaminase [EC:3.5.1.2] | map00220 Arginine biosynthesis; map00250 Alanine, aspartate and glutamate metabolism; map00471 D-Glutamine and D-glutamate metabolism; map01100 Metabolic pathways; map04724 Glutamatergic synapse; map04727 GABAergic synapse; map04964 Proximal tubule bicarbonate reclamation; map05206 MicroRNAs in cancer; map05230 Central carbon metabolism in cancer | PF04960 | Glutaminase | E | KOG0506 | Glutaminase (contains ankyrin repeat) |
| 42.158 | 24.8 | 8 | 4 | 10 | 1.079 | 1.099 | 1.248 | 0.943 | 0.881 | 0.731 | cytoskeleton | K04634 | GNAQ; guanine nucleotide-binding protein G(q) subunit alpha | map04015 Rap1 signaling pathway; map04020 Calcium signaling pathway; map04022 cGMP-PKG signaling pathway; map04071 Sphingolipid signaling pathway; map04261 Adrenergic signaling in cardiomyocytes; map04270 Vascular smooth muscle contraction; map04361 Axon regeneration; map04371 Apelin signaling pathway; map04540 Gap junction; map04611 Platelet activation; map04713 Circadian entrainment; map04720 Long-term potentiation; map04723 Retrograde endocannabinoid signaling; map04724 Glutamatergic synapse; map04725 Cholinergic synapse; map04726 Serotonergic synapse; map04728 Dopaminergic synapse; map04730 Long-term depression; map04745 Phototransduction - fly; map04750 Inflammatory mediator regulation of TRP channels; map04911 Insulin secretion; map04912 GnRH signaling pathway; map04915 Estrogen signaling pathway; map04916 Melanogenesis; map04918 Thyroid hormone synthesis; map04921 Oxytocin signaling pathway; map04922 Glucagon signaling pathway; map04924 Renin secretion; map04925 Aldosterone synthesis and secretion; map04927 Cortisol synthesis and secretion; map04928 Parathyroid hormone synthesis, secretion and action; map04929 GnRH secretion; map04934 Cushing syndrome; map04935 Growth hormone synthesis, secretion and action; map04961 Endocrine and other factor-regulated calcium reabsorption; map04970 Salivary secretion; map04971 Gastric acid secretion; map04972 Pancreatic secretion; map05010 Alzheimer disease; map05016 Huntington disease; map05142 Chagas disease (American trypanosomiasis); map05143 African trypanosomiasis; map05146 Amoebiasis; map05163 Human cytomegalovirus infection; map05170 Human immunodeficiency virus 1 infection; map05200 Pathways in cancer | PF00503 | G-protein alpha subunit | T | KOG0085 | G protein subunit Galphaq/Galphay, small G protein superfamily |
| 71.017 | 5.9 | 3 | 3 | 3 | 0.501 | 0.659 | 0.77 | 1.393 | 1.659 | 1.436 | mitochondria | K00643 | E2.3.1.37, ALAS; 5-aminolevulinate synthase [EC:2.3.1.37] | map00260 Glycine, serine and threonine metabolism; map00860 Porphyrin and chlorophyll metabolism; map01100 Metabolic pathways | PF09029; PF00155 | 5-aminolevulinate synthase presequence; Aminotransferase class I and II | H | KOG1360 | 5-aminolevulinate synthase |
| 39.632 | 30 | 12 | 12 | 44 | 1.126 | 1.109 | 1.096 | 0.784 | 0.936 | 0.77 | nucleus | K15102 | SLC25A3, PHC, PIC; solute carrier family 25 (mitochondrial phosphate transporter), member 3 | | | | C | KOG0767 | Mitochondrial phosphate carrier protein |
| 42.613 | 24.7 | 8 | 4 | 10 | 0.779 | 0.845 | 0.907 | 1.14 | 1.198 | 1.355 | cytoskeleton | K16575 | ACTR1, ARP1; centractin | | |  | Z | KOG0676 | Actin and related proteins |
| 28.37 | 4 | 1 | 1 | 1 | 0.845 | 0.796 | 0.876 | 1.149 | 1.02 | 1.138 | cytoplasm | K12587 | MTR3, EXOSC6; exosome complex component MTR3 | map03018 RNA degradation | PF01138 | 3' exoribonuclease family, domain 1 | J | KOG1068 | Exosomal 3'-5' exoribonuclease complex, subunit Rrp41 and related exoribonucleases |
| 25.701 | 68.3 | 18 | 10 | 137 | 0.442 | 0.314 | 0.386 | 1.916 | 1.593 | 1.7 | cytoplasm | K00799 | GST, gst; glutathione S-transferase [EC:2.5.1.18] | map00480 Glutathione metabolism; map00980 Metabolism of xenobiotics by cytochrome P450; map00982 Drug metabolism - cytochrome P450; map00983 Drug metabolism - other enzymes; map01100 Metabolic pathways; map01524 Platinum drug resistance; map04212 Longevity regulating pathway - worm; map05200 Pathways in cancer; map05204 Chemical carcinogenesis; map05225 Hepatocellular carcinoma; map05418 Fluid shear stress and atherosclerosis | PF02798; PF00043 | Glutathione S-transferase, N-terminal domain; Glutathione S-transferase, C-terminal domain | O | KOG1695 | Glutathione S-transferase |
| 13.509 | 31.2 | 4 | 2 | 7 | 1.037 | 1.08 | 1.232 | 0.756 | 0.877 | 0.814 | nucleus | K11251 | H2A; histone H2A | map04217 Necroptosis; map05034 Alcoholism; map05322 Systemic lupus erythematosus | PF00125 | Core histone H2A/H2B/H3/H4 | B | KOG1757 | Histone 2A |
| 54.352 | 5.2 | 3 | 3 | 3 | 1.219 | 1.199 | 1.303 | 0.851 | 0.598 | 0.869 | mitochondria | K22827 | MICU1; calcium uptake protein 1, mitochondrial | | PF13202; PF13833 | EF hand; EF-hand domain pair | C | KOG2643 | Ca2+ binding protein, contains EF-hand motifs |
| 20.93 | 56.4 | 10 | 6 | 44 | 0.718 | 0.778 | 1.01 | 1.157 | 1.441 | 1.782 | extracellular |  |  |  | PF00061 | Lipocalin / cytosolic fatty-acid binding protein family | | | |
| 18.382 | 4.7 | 1 | 1 | 5 | 1.42 | 1.244 | 1.158 | 0.678 | 0.901 | 0.633 | plasma membrane | K00236 | SDHC, SDH3; succinate dehydrogenase (ubiquinone) cytochrome b560 subunit | map00020 Citrate cycle (TCA cycle); map00190 Oxidative phosphorylation; map01100 Metabolic pathways; map01200 Carbon metabolism;  map04714 Thermogenesis; map04932 Non-alcoholic fatty liver disease (NAFLD); map05010 Alzheimer disease; map05012 Parkinson disease;  map05016 Huntington disease | | | C | KOG0449 | Succinate dehydrogenase, cytochrome b subunit |
| 74.466 | 42.8 | 29 | 24 | 71 | 1.254 | 1.192 | 1.288 | 0.823 | 0.82 | 0.746 | cytoplasm | K15105 | SLC25A12_13, AGC; solute carrier family 25 (mitochondrial aspartate/glutamate transporter), member 12/13 | | PF13833 | EF-hand domain pair | C | KOG0751 | Mitochondrial aspartate/glutamate carrier protein Aralar/Citrin (contains EF-hand Ca2+-binding domains) |
| 26.268 | 42.2 | 9 | 8 | 21 | 0.891 | 0.716 | 0.793 | 1.081 | 1.081 | 1.16 | nucleus | K16641 | HDGF; hepatoma-derived growth factor | | PF00855 | PWWP domain | K | KOG1904 | Transcription coactivator |
| 28.263 | 6.5 | 1 | 1 | 1 | 0.793 | 0.656 | 0.673 | 1.068 | 1.188 | 1.224 | cytoplasm | K03259 | EIF4E; translation initiation factor 4E | map01521 EGFR tyrosine kinase inhibitor resistance; map03013 RNA transport; map04066 HIF-1 signaling pathway; map04150 mTOR signaling pathway; map04151 PI3K-Akt signaling pathway; map04211 Longevity regulating pathway; map04910 Insulin signaling pathway | PF01652 | Eukaryotic initiation factor 4E | J | KOG1669 | Predicted mRNA cap-binding protein related to eIF-4E |
| 67.289 | 2.3 | 1 | 1 | 1 | 0.424 | 0.409 | 0.423 | 1.137 | 1.373 | 1.256 | nucleus |  |  |  |  |  |  |  |  |
| 25.345 | 9.2 | 2 | 2 | 3 | 1.232 | 1.4 | 1.101 | 0.861 | 0.838 | 0.958 | nucleus |  |  |  |  |  | S | KOG3223 | Uncharacterized conserved protein |
| 43.208 | 9.1 | 4 | 4 | 4 | 0.608 | 0.634 | 0.579 | 1.244 | 1.332 | 0.931 | plasma membrane | K20367 | ERGIC3, ERV46; endoplasmic reticulum-Golgi intermediate compartment protein 3 | | PF13850 | Endoplasmic Reticulum-Golgi Intermediate Compartment (ERGIC) | U | KOG2667 | COPII vesicle protein |
| 50.478 | 48 | 21 | 21 | 62 | 1.208 | 1.321 | 1.231 | 0.782 | 0.862 | 0.763 | cytoplasm | K00103 | GULO; L-gulonolactone oxidase [EC:1.1.3.8] | map00053 Ascorbate and aldarate metabolism; map01100 Metabolic pathways | PF01565 | FAD binding domain | V | KOG4730 | D-arabinono-1, 4-lactone oxidase |
| 69.641 | 22.9 | 17 | 2 | 57 | 0.98 | 0.77 | 0.881 | 1.459 | 1.287 | 1.222 | cytoplasm | K03283 | HSPA1s; heat shock 70kDa protein 1/2/6/8 | map03040 Spliceosome; map04010 MAPK signaling pathway; map04141 Protein processing in endoplasmic reticulum; map04144 Endocytosis;  map04213 Longevity regulating pathway - multiple species; map04612 Antigen processing and presentation; map04915 Estrogen signaling pathway;  map05134 Legionellosis; map05145 Toxoplasmosis; map05162 Measles | | | O | KOG0101 | Molecular chaperones HSP70/HSC70, HSP70 superfamily |
| 27.515 | 16.3 | 4 | 4 | 5 | 0.925 | 0.943 | 0.812 | 1.314 | 1.256 | 1.213 | cytoplasm | K06831 | LGALS3; galectin-3 | | PF00337 | Galactoside-binding lectin | W | KOG3587 | Galectin, galactose-binding lectin |
| 164.86 | 15.9 | 20 | 20 | 30 | 1.054 | 1.122 | 1.142 | 0.818 | 0.868 | 0.69 | plasma membrane | K05669 | ABCC6; ATP-binding cassette, subfamily C (CFTR/MRP), member 6 | map02010 ABC transporters | PF00005 | ABC transporter | Q | KOG0054 | Multidrug resistance-associated protein/mitoxantrone resistance protein, ABC superfamily |
| 31.715 | 26.5 | 7 | 7 | 18 | 1.137 | 1.212 | 1.051 | 0.845 | 0.874 | 0.774 | extracellular | K13577 | SLC25A10, DIC; solute carrier family 25 (mitochondrial dicarboxylate transporter), member 10 | map04964 Proximal tubule bicarbonate reclamation | | | C | KOG0759 | Mitochondrial oxoglutarate/malate carrier proteins |
| 85.025 | 14.7 | 10 | 10 | 11 | 0.593 | 0.814 | 0.749 | 1.21 | 1.42 | 1.041 | plasma membrane | K06464 | ITGB2, CD18; integrin beta 2 | map04015 Rap1 signaling pathway; map04145 Phagosome; map04390 Hippo signaling pathway; map04514 Cell adhesion molecules (CAMs); map04610 Complement and coagulation cascades; map04650 Natural killer cell mediated cytotoxicity; map04670 Leukocyte transendothelial migration; map04810 Regulation of actin cytoskeleton; map05133 Pertussis; map05134 Legionellosis; map05140 Leishmaniasis; map05144 Malaria; map05146 Amoebiasis; map05150 Staphylococcus aureus infection; map05152 Tuberculosis; map05166 Human T-cell leukemia virus 1 infection; map05323 Rheumatoid arthritis; map05416 Viral myocarditis | PF00362; PF07965; PF08725 | Integrin, beta chain; Integrin beta tail domain; Integrin beta cytoplasmic domain | T | KOG1226 | Integrin beta subunit (N-terminal portion of extracellular region) |
| 136.95 | 6.6 | 8 | 8 | 9 | 0.87 | 0.766 | 0.683 | 1.068 | 1.039 | 1.458 | nucleus |  |  |  |  |  |  |  |  |
| 62.844 | 5.6 | 4 | 1 | 3 | 0.76 | 0.583 | 0.517 | 0.939 | 1.092 | 1.334 | nucleus | K07605 | KRT2; type II keratin, basic | | |  |  |  |  |
| 20.373 | 31.8 | 7 | 7 | 11 | 1.641 | 1.773 | 1.444 | 0.695 | 0.908 | 0.704 | extracellular | K22556 | MANF, ARMET; mesencephalic astrocyte-derived neurotrophic factor | | | | S | KOG4154 | Arginine-rich protein |
| 13.813 | 38.3 | 6 | 6 | 31 | 1.259 | 1.263 | 1.177 | 0.863 | 0.747 | 0.924 | mitochondria | K02265 | COX5B; cytochrome c oxidase subunit 5b | map00190 Oxidative phosphorylation; map01100 Metabolic pathways; map04260 Cardiac muscle contraction; map04714 Thermogenesis; map04932 Non-alcoholic fatty liver disease (NAFLD); map05010 Alzheimer disease; map05012 Parkinson disease; map05016 Huntington disease | PF01215 | Cytochrome c oxidase subunit Vb | C | KOG3352 | Cytochrome c oxidase, subunit Vb/COX4 |
| 53.918 | 16.6 | 7 | 7 | 10 | 1.404 | 1.384 | 1.17 | 0.7 | 1.079 | 0.678 | plasma membrane | K00213 | DHCR7; 7-dehydrocholesterol reductase [EC:1.3.1.21] | map00100 Steroid biosynthesis; map01100 Metabolic pathways | | | IT | KOG1435 | Sterol reductase/lamin B receptor |
| 31.205 | 13.3 | 4 | 4 | 5 | 1.296 | 1.303 | 1.61 | 0.698 | 0.938 | 0.608 | cytoplasm,nucleus | |  |  | PF00753 | Metallo-beta-lactamase superfamily | S | KOG0813 | Glyoxylase |
| 38.299 | 32.7 | 11 | 11 | 45 | 1.12 | 1.184 | 1.216 | 0.838 | 0.856 | 0.871 | mitochondria | K00019 | E1.1.1.30, bdh; 3-hydroxybutyrate dehydrogenase [EC:1.1.1.30] | map00072 Synthesis and degradation of ketone bodies; map00650 Butanoate metabolism; map01100 Metabolic pathways | PF00106 | short chain dehydrogenase | Q | KOG1610 | Corticosteroid 11-beta-dehydrogenase and related short chain-type dehydrogenases |
| 65.74 | 8.8 | 6 | 6 | 7 | 1.222 | 1.259 | 1.148 | 0.872 | 0.918 | 0.872 | cytoplasm | K04723 | IL1RAP; interleukin 1 receptor accessory protein | map04010 MAPK signaling pathway; map04060 Cytokine-cytokine receptor interaction; map04659 Th17 cell differentiation; map04750 Inflammatory mediator regulation of TRP channels | PF13895; PF13927 | Immunoglobulin domain; Immunoglobulin domain | | | |
| 109.34 | 1 | 1 | 1 | 1 | 1.914 | 2.547 | 1.54 | 0.563 | 0.361 | 0.433 | endoplasmic reticulum | K05091 | KIT, SCFR, CD117; proto-oncogene tyrosine-protein kinase Kit [EC:2.7.10.1] | map04010 MAPK signaling pathway; map04014 Ras signaling pathway; map04015 Rap1 signaling pathway; map04072 Phospholipase D signaling pathway; map04151 PI3K-Akt signaling pathway; map04640 Hematopoietic cell lineage; map04916 Melanogenesis; map05200 Pathways in cancer; map05221 Acute myeloid leukemia; map05224 Breast cancer; map05230 Central carbon metabolism in cancer | PF00047; PF07714 | Immunoglobulin domain; Protein tyrosine kinase | T | KOG0200 | Fibroblast/platelet-derived growth factor receptor and related receptor tyrosine kinases |
| 20.555 | 16.6 | 3 | 3 | 8 | 0.804 | 0.782 | 0.94 | 1.108 | 1.096 | 1.096 | cytoplasm | K07575 | K07575; PUA domain protein | | |  | K | KOG2523 | Predicted RNA-binding protein with PUA domain |
| 16.369 | 38.5 | 6 | 6 | 6 | 0.89 | 0.848 | 0.728 | 1.188 | 1.047 | 1.069 | mitochondria | K03627 | MBF1; putative transcription factor | | PF01381 | Helix-turn-helix | K | KOG3398 | Transcription factor MBF1 |
| 46.977 | 17.9 | 5 | 1 | 9 | 0.703 | 0.413 | 0.726 | 1.627 | 1.953 | 0.96 | nucleus | K20914 | IFI16; gamma-interferon-inducible protein 16 | map04621 NOD-like receptor signaling pathway | PF02758 | PAAD/DAPIN/Pyrin domain | | |  |
| 49.135 | 3.8 | 2 | 2 | 3 | 0.694 | 0.657 | 0.504 | 1.008 | 0.954 | 1.087 | cytoplasm | K21031 | ISPD; D-ribitol-5-phosphate cytidylyltransferase [EC:2.7.7.40] | map00040 Pentose and glucuronate interconversions; map00515 Mannose type O-glycan biosynthesis; map01100 Metabolic pathways | | | | | |
| 176.35 | 1.6 | 2 | 2 | 2 | 1.209 | 1.209 | 0.927 | 0.851 | 0.815 | 0.89 | extracellular | K11448 | KDM6B, JMJD3; lysine-specific demethylase 6B [EC:1.14.11.68] | | PF02373 | JmjC domain, hydroxylase | C | KOG1246 | DNA-binding protein jumonji/RBP2/SMCY, contains JmjC domain |
| 80.169 | 6 | 4 | 4 | 4 | 0.159 | 0.269 | 0.197 | 1.793 | 2.916 | 1.367 | nucleus | K10394 | KIF3A; kinesin family member 3A | map04340 Hedgehog signaling pathway | PF00225 | Kinesin motor domain | Z | KOG4280 | Kinesin-like protein |
| 18.749 | 80.7 | 13 | 13 | 51 | 1.206 | 1.233 | 1.289 | 0.808 | 0.835 | 0.724 | cytoplasm | K02138 | ATPeF0D, ATP5H, ATP7; F-type H+-transporting ATPase subunit d | map00190 Oxidative phosphorylation; map01100 Metabolic pathways; map04714 Thermogenesis; map05010 Alzheimer disease;  map05012 Parkinson disease; map05016 Huntington disease | | | C | KOG3366 | Mitochondrial F1F0-ATP synthase, subunit d/ATP7 |
| 56.987 | 24 | 13 | 1 | 49 | 1.218 | 1.234 | 1.228 | 0.815 | 0.884 | 0.587 | endoplasmic reticulum | K07414 | CYP2D; cytochrome P450 family 2 subfamily D [EC:1.14.14.1] | map00140 Steroid hormone biosynthesis; map04726 Serotonergic synapse | PF00067 | Cytochrome P450 | Q | KOG0156 | Cytochrome P450 CYP2 subfamily |
| 38.224 | 20.5 | 6 | 6 | 10 | 0.91 | 0.775 | 0.727 | 1.121 | 1.38 | 1.213 | cytoplasm | K01784 | galE, GALE; UDP-glucose 4-epimerase [EC:5.1.3.2] | map00052 Galactose metabolism; map00520 Amino sugar and nucleotide sugar metabolism; map01100 Metabolic pathways | | | M | KOG1371 | UDP-glucose 4-epimerase/UDP-sulfoquinovose synthase |
| 84.787 | 49.4 | 36 | 26 | 136 | 0.905 | 0.888 | 0.87 | 1.237 | 1.159 | 1.188 | cytoplasm | K04079 | HSP90A, htpG; molecular chaperone HtpG | map04141 Protein processing in endoplasmic reticulum; map04151 PI3K-Akt signaling pathway; map04217 Necroptosis; map04612 Antigen processing and presentation; map04621 NOD-like receptor signaling pathway; map04657 IL-17 signaling pathway; map04659 Th17 cell differentiation; map04914 Progesterone-mediated oocyte maturation; map04915 Estrogen signaling pathway; map05200 Pathways in cancer; map05215 Prostate cancer; map05418 Fluid shear stress and atherosclerosis | PF02518 | Histidine kinase-, DNA gyrase B-, and HSP90-like ATPase | O | KOG0019 | Molecular chaperone (HSP90 family) |
| 28.061 | 2.7 | 1 | 1 | 1 | 0.35 | 0.254 | 0.28 | 1.187 | 1.2 | 1.76 | extracellular | K05074 | IL15RA, CD215; interleukin 15 receptor alpha | map04060 Cytokine-cytokine receptor interaction; map04630 JAK-STAT signaling pathway; map04672 Intestinal immune network for IgA production; map05166 Human T-cell leukemia virus 1 infection; map05200 Pathways in cancer | PF00084 | Sushi domain (SCR repeat) | | |  |
| 8.4689 | 46.1 | 4 | 4 | 12 | 1.507 | 1.557 | 1.462 | 0.714 | 0.566 | 0.692 | mitochondria | K02268 | COX6C; cytochrome c oxidase subunit 6c | map00190 Oxidative phosphorylation; map01100 Metabolic pathways; map04260 Cardiac muscle contraction; map04714 Thermogenesis;  map04932 Non-alcoholic fatty liver disease (NAFLD); map05010 Alzheimer disease; map05012 Parkinson disease; map05016 Huntington disease | | | | | |
| 84.186 | 6 | 3 | 3 | 3 | 1.245 | 1.289 | 1.36 | 0.802 | 0.86 | 0.73 | plasma membrane | |  |  |  |  | S | KOG4587 | Predicted membrane protein |
| 31.218 | 3.2 | 1 | 1 | 1 | 0.859 | 0.791 | 0.947 | 1.043 | 1.253 | 1.256 | cytoplasm | K00586 | DPH5; diphthine methyl ester synthase [EC:2.1.1.314] | | PF00590 | Tetrapyrrole (Corrin/Porphyrin) Methylases | J | KOG3123 | Diphthine synthase |
| 47.13 | 35.5 | 15 | 15 | 31 | 1.145 | 1.182 | 1.17 | 0.815 | 0.815 | 1.017 | mitochondria | K01772 | hemH, FECH; protoporphyrin/coproporphyrin ferrochelatase [EC:4.99.1.1 4.99.1.9] | map00860 Porphyrin and chlorophyll metabolism; map01100 Metabolic pathways | PF00762 | Ferrochelatase | H | KOG1321 | Protoheme ferro-lyase (ferrochelatase) |
| 26.585 | 5.2 | 1 | 1 | 4 | 1.31 | 1.183 | 1.451 | 0.831 | 0.744 | 0.935 | mitochondria | |  |  |  |  |  |  |  |
| 59.71 | 5.2 | 3 | 3 | 3 | 0.799 | 0.674 | 0.771 | 1.257 | 1.001 | 1.275 | nucleus | K12605 | CNOT2, NOT2; CCR4-NOT transcription complex subunit 2 | map03018 RNA degradation | | | K | KOG2151 | Predicted transcriptional regulator |
| 15.676 | 37.2 | 5 | 5 | 9 | 1.362 | 1.106 | 1.132 | 0.874 | 0.838 | 0.799 | extracellular | K23588 | MLLT6_10, AF17_10; protein AF-17/10 | | PF09360 | Iron-binding zinc finger CDGSH type | S | KOG4605 | Uncharacterized conserved protein containing CDGSH-type Zn-finger |
| 56.421 | 28.3 | 14 | 13 | 24 | 1.413 | 1.14 | 1.038 | 0.673 | 0.704 | 0.769 | endoplasmic reticulum | K07413 | CYP2C; cytochrome P450 family 2 subfamily C [EC:1.14.14.1] | map00140 Steroid hormone biosynthesis; map00590 Arachidonic acid metabolism; map00591 Linoleic acid metabolism; map00830 Retinol metabolism; map01100 Metabolic pathways; map04726 Serotonergic synapse; map04750 Inflammatory mediator regulation of TRP channels; map05204 Chemical carcinogenesis | PF00067 | Cytochrome P450 | Q | KOG0156 | Cytochrome P450 CYP2 subfamily |
| 57.854 | 44.2 | 24 | 24 | 73 | 0.979 | 1.439 | 1.323 | 0.723 | 0.864 | 0.49 | endoplasmic reticulum | K07424 | CYP3A; cytochrome P450 family 3 subfamily A [EC:1.14.14.1] | map00140 Steroid hormone biosynthesis; map00591 Linoleic acid metabolism; map00830 Retinol metabolism; map01100 Metabolic pathways; map05204 Chemical carcinogenesis | PF00067 | Cytochrome P450 | Q | KOG0158 | Cytochrome P450 CYP3/CYP5/CYP6/CYP9 subfamilies |
| 15.241 | 47.8 | 5 | 5 | 36 | 0.863 | 0.807 | 0.957 | 1.123 | 1.3 | 1.061 | cytoplasm | K23490 | CYB5; cytochrome b5 | | PF00173 | Cytochrome b5-like Heme/Steroid binding domain | C | KOG0537 | Cytochrome b5 |
| 31.599 | 8.1 | 2 | 2 | 3 | 1.105 | 1.028 | 1.007 | 0.799 | 0.832 | 0.735 | mitochondria | |  |  |  |  | S | KOG4082 | Uncharacterized conserved protein |
| 11.118 | 7.8 | 1 | 1 | 2 | 0.635 | 0.622 | 0.468 | 1.23 | 0.716 | 1.243 | extracellular |  |  |  |  |  |  |  |  |
| 22.417 | 17 | 3 | 3 | 11 | 1.3 | 1.213 | 1.108 | 0.786 | 0.853 | 0.876 | cytoplasm,nucleus | |  |  |  |  | Q | KOG4044 | Mitochondrial associated endoribonuclease MAR1 (isochorismatase superfamily) |
| 197.09 | 1 | 2 | 2 | 3 | 0.49 | 0.571 | 0.569 | 1.336 | 1.186 | 1.216 | extracellular | K05636 | LAMB1; laminin, beta 1 | map04151 PI3K-Akt signaling pathway; map04510 Focal adhesion; map04512 ECM-receptor interaction; map05145 Toxoplasmosis; map05146 Amoebiasis; map05165 Human papillomavirus infection; map05200 Pathways in cancer; map05222 Small cell lung cancer | PF00053 | Laminin EGF-like (Domains III and V) | W | KOG0994 | Extracellular matrix glycoprotein Laminin subunit beta |
| 16.794 | 4.7 | 1 | 1 | 2 | 0.784 | 0.724 | 0.786 | 1.122 | 1.809 | 1.371 | extracellular | K13915 | LYZ; lysozyme C [EC:3.2.1.17] | map04970 Salivary secretion | PF00062 | C-type lysozyme/alpha-lactalbumin family | | | |
| 68.561 | 3.4 | 2 | 2 | 2 | 0.796 | 0.779 | 0.904 | 1.006 | 1.063 | 1.179 | extracellular | K03355 | APC8, CDC23; anaphase-promoting complex subunit 8 | map04110 Cell cycle; map04114 Oocyte meiosis; map04120 Ubiquitin mediated proteolysis; map04914 Progesterone-mediated oocyte maturation;  map05166 Human T-cell leukemia virus 1 infection | | | DO | KOG1155 | Anaphase-promoting complex (APC), Cdc23 subunit |
| 46.52 | 12.7 | 6 | 6 | 7 | 1.849 | 1.761 | 0.974 | 0.825 | 0.785 | 0.54 | plasma membrane | K00222 | TM7SF2, ERG24; Delta14-sterol reductase [EC:1.3.1.70] | map00100 Steroid biosynthesis; map01100 Metabolic pathways | | | IT | KOG1435 | Sterol reductase/lamin B receptor |
| 15.806 | 22.9 | 3 | 3 | 6 | 1.263 | 1.318 | 1.095 | 0.847 | 1.021 | 0.826 | plasma membrane | |  |  |  |  | S | KOG3455 | Predicted membrane protein |
| 23.407 | 37.3 | 8 | 8 | 23 | 0.854 | 0.892 | 0.86 | 1.007 | 1.312 | 1.084 | cytoplasm | K12462 | ARHGDI, RHOGDI; Rho GDP-dissociation inhibitor | map04722 Neurotrophin signaling pathway; map04962 Vasopressin-regulated water reabsorption | PF02115 | RHO protein GDP dissociation inhibitor | T | KOG3205 | Rho GDP-dissociation inhibitor |
| 25.542 | 41.9 | 10 | 1 | 49 | 0.519 | 0.346 | 0.21 | 2.223 | 0.849 | 2.044 | cytoplasm | K00799 | GST, gst; glutathione S-transferase [EC:2.5.1.18] | map00480 Glutathione metabolism; map00980 Metabolism of xenobiotics by cytochrome P450; map00982 Drug metabolism - cytochrome P450; map00983 Drug metabolism - other enzymes; map01100 Metabolic pathways; map01524 Platinum drug resistance; map04212 Longevity regulating pathway - worm; map05200 Pathways in cancer; map05204 Chemical carcinogenesis; map05225 Hepatocellular carcinoma; map05418 Fluid shear stress and atherosclerosis | PF02798; PF00043 | Glutathione S-transferase, N-terminal domain; Glutathione S-transferase, C-terminal domain | O | KOG1695 | Glutathione S-transferase |
| 96.02 | 15.4 | 12 | 10 | 14 | 0.863 | 0.768 | 0.805 | 1.096 | 1.107 | 1.055 | nucleus | K13090 | ILF3; interleukin enhancer-binding factor 3 | | PF07528; PF00035 | DZF domain; Double-stranded RNA binding motif | K | KOG3792 | Transcription factor NFAT, subunit NF90 |
| 39.035 | 2.9 | 1 | 1 | 2 | 0.582 | 0.507 | 0.494 | 1.868 | 1.252 | 1.46 | nucleus | K09370 | ISL1; insulin gene enhancer protein ISL-1 | map04550 Signaling pathways regulating pluripotency of stem cells | PF00412; PF00046 | LIM domain; Homeobox domain | K | KOG0490 | Transcription factor, contains HOX domain |
| 16.433 | 6 | 1 | 1 | 1 | 0.719 | 0.727 | 0.7 | 1.432 | 1.284 | 1.121 | extracellular |  |  |  | PF07686 | Immunoglobulin V-set domain | | |  |
| 131.96 | 11 | 11 | 11 | 12 | 1.073 | 1.207 | 1.221 | 0.806 | 0.93 | 0.709 | plasma membrane | |  |  | PF00041 | Fibronectin type III domain | | |  |
| 7.3184 | 10.3 | 1 | 1 | 1 | 1.303 | 1.282 | 1.468 | 0.76 | 0.785 | 0.61 | extracellular | K04542 | GNG5; guanine nucleotide-binding protein G(I)/G(S)/G(O) subunit gamma-5 | map04014 Ras signaling pathway; map04062 Chemokine signaling pathway; map04151 PI3K-Akt signaling pathway; map04371 Apelin signaling pathway; map04713 Circadian entrainment; map04723 Retrograde endocannabinoid signaling; map04724 Glutamatergic synapse; map04725 Cholinergic synapse; map04726 Serotonergic synapse; map04727 GABAergic synapse; map04728 Dopaminergic synapse; map04926 Relaxin signaling pathway; map05032 Morphine addiction; map05034 Alcoholism; map05163 Human cytomegalovirus infection; map05167 Kaposi sarcoma-associated herpesvirus infection; map05170 Human immunodeficiency virus 1 infection; map05200 Pathways in cancer | PF00631 | GGL domain | T | KOG4119 | G protein gamma subunit |
| 86.052 | 5.6 | 3 | 3 | 3 | 1.244 | 1.435 | 1.193 | 0.734 | 0.79 | 0.758 | nucleus | K05771 | NR3C1, GR; glucocorticoid receptor | map04080 Neuroactive ligand-receptor interaction | PF00105; PF00104 | Zinc finger, C4 type (two domains); Ligand-binding domain of nuclear hormone receptor | K | KOG3575 | FOG: Hormone receptors |
| 24.915 | 8 | 2 | 2 | 3 | 1.382 | 1.437 | 1.561 | 0.873 | 0.639 | 0.51 | plasma membrane | |  |  |  |  | T | KOG4363 | Putative growth response protein |
| 14.954 | 21.9 | 2 | 1 | 2 | 1.432 | 1.219 | 1.258 | 0.815 | 0.804 | 0.852 | plasma membrane | K06566 | IFITM; interferon induced transmembrane protein | | | |  |  |  |
| 25.219 | 10.7 | 3 | 3 | 4 | 1.892 | 2.33 | 1.802 | 0.66 | 0.389 | 0.638 | cytoplasm | K12193 | VPS24, CHMP3; charged multivesicular body protein 3 | map04144 Endocytosis; map04217 Necroptosis | | | U | KOG3229 | Vacuolar sorting protein VPS24 |
| 58.279 | 9.3 | 4 | 4 | 4 | 0.889 | 0.832 | 0.789 | 1.301 | 1.035 | 0.984 | cytoplasm | K16195 | EIF2AK2; eukaryotic translation initiation factor 2-alpha kinase 2 [EC:2.7.11.1] | map04141 Protein processing in endoplasmic reticulum; map04217 Necroptosis; map05160 Hepatitis C; map05162 Measles; map05164 Influenza A; map05165 Human papillomavirus infection; map05167 Kaposi sarcoma-associated herpesvirus infection; map05168 Herpes simplex virus 1 infection; map05169 Epstein-Barr virus infection; map05203 Viral carcinogenesis | PF00035; PF00069 | Double-stranded RNA binding motif; Protein kinase domain | J | KOG1033 | eIF-2alpha kinase PEK/EIF2AK3 |
| 40.634 | 8.3 | 3 | 3 | 3 | 1.673 | 1.762 | 1.648 | 0.934 | 0.889 | 0.624 | mitochondria | K22745 | AIFM2; apoptosis-inducing factor 2 | map04115 p53 signaling pathway | PF07992 | Pyridine nucleotide-disulphide oxidoreductase | S | KOG1336 | Monodehydroascorbate/ferredoxin reductase |
| 66.365 | 36.2 | 18 | 18 | 38 | 1.206 | 1.281 | 1.276 | 0.756 | 0.608 | 0.726 | mitochondria | K01425 | glsA, GLS; glutaminase [EC:3.5.1.2] | map00220 Arginine biosynthesis; map00250 Alanine, aspartate and glutamate metabolism; map00471 D-Glutamine and D-glutamate metabolism; map01100 Metabolic pathways; map04724 Glutamatergic synapse; map04727 GABAergic synapse; map04964 Proximal tubule bicarbonate reclamation; map05206 MicroRNAs in cancer; map05230 Central carbon metabolism in cancer | PF04960 | Glutaminase | E | KOG0506 | Glutaminase (contains ankyrin repeat) |
| 25.716 | 70.2 | 17 | 9 | 109 | 0.792 | 0.688 | 0.843 | 1.355 | 1.181 | 1.268 | cytoplasm | K00799 | GST, gst; glutathione S-transferase [EC:2.5.1.18] | map00480 Glutathione metabolism; map00980 Metabolism of xenobiotics by cytochrome P450; map00982 Drug metabolism - cytochrome P450; map00983 Drug metabolism - other enzymes; map01100 Metabolic pathways; map01524 Platinum drug resistance; map04212 Longevity regulating pathway - worm; map05200 Pathways in cancer; map05204 Chemical carcinogenesis; map05225 Hepatocellular carcinoma; map05418 Fluid shear stress and atherosclerosis | PF02798; PF00043 | Glutathione S-transferase, N-terminal domain; Glutathione S-transferase, C-terminal domain | O | KOG1695 | Glutathione S-transferase |
| 46.763 | 6.9 | 3 | 3 | 4 | 1.255 | 1.469 | 1.448 | 0.981 | 0.674 | 0.904 | cytoplasm |  |  |  |  |  |  |  |  |
| 30.953 | 30.7 | 6 | 5 | 7 | 0.572 | 0.494 | 0.52 | 1.362 | 1.668 | 1.765 | mitochondria | K00084 | CBR3; carbonyl reductase 3 [EC:1.1.1.184] | map00590 Arachidonic acid metabolism; map00980 Metabolism of xenobiotics by cytochrome P450; map01100 Metabolic pathways | PF00106; PF13561 | short chain dehydrogenase; Enoyl-(Acyl carrier protein) reductase | Q | KOG1208 | Dehydrogenases with different specificities (related to short-chain alcohol dehydrogenases) |
| 25.608 | 45.3 | 11 | 1 | 53 | 0.196 | 0.078 | 0.071 | 3.057 | 1.008 | 2.276 | cytoplasm | K00799 | GST, gst; glutathione S-transferase [EC:2.5.1.18] | map00480 Glutathione metabolism; map00980 Metabolism of xenobiotics by cytochrome P450; map00982 Drug metabolism - cytochrome P450; map00983 Drug metabolism - other enzymes; map01100 Metabolic pathways; map01524 Platinum drug resistance; map04212 Longevity regulating pathway - worm; map05200 Pathways in cancer; map05204 Chemical carcinogenesis; map05225 Hepatocellular carcinoma; map05418 Fluid shear stress and atherosclerosis | PF02798; PF00043 | Glutathione S-transferase, N-terminal domain; Glutathione S-transferase, C-terminal domain | O | KOG1695 | Glutathione S-transferase |
| 9.0113 | 8.8 | 1 | 1 | 2 | 0.788 | 0.92 | 0.84 | 1.16 | 1.076 | 1.134 | mitochondria | K02271 | COX7B; cytochrome c oxidase subunit 7b | map00190 Oxidative phosphorylation; map01100 Metabolic pathways; map04260 Cardiac muscle contraction; map04714 Thermogenesis;  map04932 Non-alcoholic fatty liver disease (NAFLD); map05010 Alzheimer disease; map05012 Parkinson disease; map05016 Huntington disease | | | | | |
| 28.463 | 11.2 | 3 | 3 | 4 | 0.863 | 0.854 | 0.816 | 1.082 | 1.258 | 1.112 | cytoplasm | K08583 | CAPNS1, CAPN4; calpain, small subunit 1 | map05131 Shigellosis | PF13833 | EF-hand domain pair | T | KOG0037 | Ca2+-binding protein, EF-Hand protein superfamily |
| 55.763 | 40.9 | 19 | 17 | 49 | 1.252 | 1.112 | 1.17 | 0.76 | 1.036 | 0.785 | endoplasmic reticulum | K07413 | CYP2C; cytochrome P450 family 2 subfamily C [EC:1.14.14.1] | map00140 Steroid hormone biosynthesis; map00590 Arachidonic acid metabolism; map00591 Linoleic acid metabolism; map00830 Retinol metabolism; map01100 Metabolic pathways; map04726 Serotonergic synapse; map04750 Inflammatory mediator regulation of TRP channels; map05204 Chemical carcinogenesis | PF00067 | Cytochrome P450 | Q | KOG0156 | Cytochrome P450 CYP2 subfamily |
| 53.662 | 31.2 | 13 | 13 | 28 | 0.923 | 0.768 | 0.845 | 1.243 | 1.214 | 1.2 | mitochondria | K00383 | GSR, gor; glutathione reductase (NADPH) [EC:1.8.1.7] | map00480 Glutathione metabolism; map01100 Metabolic pathways; map04918 Thyroid hormone synthesis | PF07992; PF02852 | Pyridine nucleotide-disulphide oxidoreductase; Pyridine nucleotide-disulphide oxidoreductase, dimerisation domain | C | KOG0405 | Pyridine nucleotide-disulphide oxidoreductase |
| 40.243 | 12.6 | 4 | 4 | 4 | 1.415 | 1.367 | 1.271 | 0.849 | 0.988 | 0.869 | nucleus | K15189 | HEXIM1_2; protein HEXIM1/2 | | |  |  |  |  |
| 211.46 | 2.2 | 4 | 4 | 4 | 0.912 | 0.867 | 0.757 | 1.294 | 0.978 | 1.222 | plasma membrane | K20163 | DENND4; DENN domain-containing protein 4 | | PF03456; PF03455 | uDENN domain; dDENN domain | T | KOG2127 | Calmodulin-binding protein CRAG, contains DENN domain |
| 26.476 | 28.1 | 6 | 6 | 8 | 0.842 | 0.799 | 0.806 | 1.21 | 1.19 | 1.032 | mitochondria | |  |  | PF01121 | Dephospho-CoA kinase | H | KOG3220 | Similar to bacterial dephospho-CoA kinase |
| 40.472 | 2 | 1 | 1 | 2 | 1.148 | 1.446 | 1.194 | 0.693 | 0.946 | 0.73 | plasma membrane | K01084 | G6PC; glucose-6-phosphatase [EC:3.1.3.9] | map00010 Glycolysis / Gluconeogenesis; map00052 Galactose metabolism; map00500 Starch and sucrose metabolism; map01100 Metabolic pathways;  map04068 FoxO signaling pathway; map04151 PI3K-Akt signaling pathway; map04152 AMPK signaling pathway; map04910 Insulin signaling pathway;  map04920 Adipocytokine signaling pathway; map04922 Glucagon signaling pathway; map04931 Insulin resistance;  map04973 Carbohydrate digestion and absorption | | | | | |
| 54.208 | 31.8 | 14 | 14 | 28 | 0.796 | 0.852 | 0.847 | 1.448 | 1.007 | 1.236 | cytoplasm |  |  |  | PF00106 | short chain dehydrogenase | Q | KOG4170 | 2-enoyl-CoA hydratase/3-hydroxyacyl-CoA dehydrogenase/Peroxisomal 3-ketoacyl-CoA-thiolase, sterol-binding domain and related enzymes |
| 38.862 | 16.2 | 5 | 5 | 7 | 0.624 | 0.846 | 0.637 | 1.37 | 1.516 | 1.093 | extracellular |  |  |  | PF00530 | Scavenger receptor cysteine-rich domain | | | |
| 25.485 | 4.9 | 1 | 1 | 1 | 1.213 | 1.117 | 1.309 | 0.715 | 0.815 | 0.9 | extracellular |  |  |  |  |  |  |  |  |
| 313.82 | 0.5 | 1 | 1 | 1 | 3.687 | 1.014 | 2.658 | 0.234 | 0.24 | 0.332 | extracellular | K23342 | FBN2_3; fibrillin 2/3 | | PF07645; PF12661; PF12662 | Calcium-binding EGF domain; Human growth factor-like EGF; Complement Clr-like EGF-like | T | KOG1217 | Fibrillins and related proteins containing Ca2+-binding EGF-like domains |
| 62.679 | 40 | 20 | 17 | 51 | 0.866 | 0.944 | 0.745 | 1.108 | 1.238 | 1.04 | endoplasmic reticulum | K01044 | CES1; carboxylesterase 1 [EC:3.1.1.1] | map00983 Drug metabolism - other enzymes | PF00135 | Carboxylesterase family | I | KOG1516 | Carboxylesterase and related proteins |
| 37.402 | 10.2 | 3 | 3 | 4 | 0.775 | 0.717 | 0.909 | 1.144 | 1.299 | 1.134 | nucleus | K00750 | GYG1, GYG2; glycogenin [EC:2.4.1.186] | map00500 Starch and sucrose metabolism; map01100 Metabolic pathways | | | G | KOG1950 | Glycosyl transferase, family 8 - glycogenin |
| 86.041 | 1.1 | 1 | 1 | 2 | 1.096 | 1.145 | 1.182 | 0.87 | 0.823 | 0.696 | extracellular | K06589 | ITGB6; integrin beta 6 | map04151 PI3K-Akt signaling pathway; map04510 Focal adhesion; map04512 ECM-receptor interaction; map04810 Regulation of actin cytoskeleton; map05165 Human papillomavirus infection; map05410 Hypertrophic cardiomyopathy (HCM); map05412 Arrhythmogenic right ventricular cardiomyopathy (ARVC); map05414 Dilated cardiomyopathy (DCM) | PF00362; PF07965; PF08725 | Integrin, beta chain; Integrin beta tail domain; Integrin beta cytoplasmic domain | TW | KOG1226 | Integrin beta subunit (N-terminal portion of extracellular region) |
| 254.47 | 0.4 | 1 | 1 | 1 | 0.844 | 0.776 | 0.654 | 1.075 | 1.21 | 1.21 | extracellular |  |  |  | PF10347 | RNA pol II promoter Fmp27 protein domain | T | KOG1910 | Uncharacterized conserved protein |
| 97.213 | 7 | 5 | 1 | 5 | 1.361 | 1.15 | 1.227 | 0.76 | 0.848 | 0.765 | cytoplasm | K11593 | ELF2C, AGO; eukaryotic translation initiation factor 2C | map04361 Axon regeneration | PF08699; PF02170 | Domain of unknown function (DUF1785); PAZ domain | J | KOG1041 | Translation initiation factor 2C (eIF-2C) and related proteins |
| 34.46 | 18.9 | 4 | 4 | 5 | 0.802 | 0.838 | 0.894 | 1.115 | 1.203 | 1.271 | cytoplasm | K13981 | AKR1E2, AKR1CL2; 1,5-anhydro-D-fructose reductase [EC:1.1.1.263] | | PF00248 | Aldo/keto reductase family | C | KOG1577 | Aldo/keto reductase family proteins |
| 76.187 | 2.9 | 2 | 2 | 3 | 0.902 | 0.819 | 0.708 | 1.073 | 1.041 | 1.167 | plasma membrane | K19327 | ANO10, TMEM16K; anoctamin-10 | | |  | D | KOG2513 | Protein required for meiotic chromosome segregation |
| 59.795 | 66.8 | 32 | 32 | 263 | 0.958 | 0.976 | 0.842 | 1.305 | 1.094 | 1.279 | cytoplasm | K03781 | katE, CAT, catB, srpA; catalase [EC:1.11.1.6] | map00380 Tryptophan metabolism; map00630 Glyoxylate and dicarboxylate metabolism; map01100 Metabolic pathways; map01200 Carbon metabolism; map04068 FoxO signaling pathway; map04146 Peroxisome; map04211 Longevity regulating pathway; map04212 Longevity regulating pathway - worm; map04213 Longevity regulating pathway - multiple species; map05014 Amyotrophic lateral sclerosis (ALS) | PF00199 | Catalase | C | KOG0047 | Catalase |
| 31.595 | 17 | 5 | 5 | 6 | 1.302 | 1.39 | 1.378 | 0.76 | 0.894 | 0.813 | cytoplasm | K04396 | CASP6; caspase 6 [EC:3.4.22.59] | map04210 Apoptosis | PF00656 | Caspase domain | D | KOG3573 | Caspase, apoptotic cysteine protease |
| 58.006 | 2 | 2 | 2 | 2 | 0.821 | 0.811 | 0.93 | 1.221 | 1.498 | 1.05 | cytoplasm | K19469 | FTO; mRNA N6-methyladenine demethylase [EC:1.14.11.53] | | PF12933; PF12934 | FTO catalytic domain; FTO C-terminal domain | | | |
| 20.802 | 60.7 | 9 | 9 | 40 | 1.392 | 1.436 | 1.287 | 0.847 | 0.786 | 0.841 | mitochondria | K13625 | FTL; ferritin light chain | map04216 Ferroptosis; map04217 Necroptosis; map04978 Mineral absorption | PF00210 | Ferritin-like domain | P | KOG2332 | Ferritin |
| 42.712 | 10.6 | 3 | 3 | 5 | 1.15 | 1.171 | 0.985 | 0.826 | 0.816 | 0.706 | extracellular | K23411 | FETUB; fetuin-B | | PF00031 | Cystatin domain | |  |  |
| 37.331 | 31.2 | 9 | 3 | 22 | 0.922 | 0.75 | 0.722 | 1.059 | 1.094 | 1.212 | cytoplasm | K04537 | GNB2; guanine nucleotide-binding protein G(I)/G(S)/G(T) subunit beta-2 | map04014 Ras signaling pathway; map04062 Chemokine signaling pathway; map04151 PI3K-Akt signaling pathway; map04371 Apelin signaling pathway;  map04713 Circadian entrainment; map04723 Retrograde endocannabinoid signaling; map04724 Glutamatergic synapse; map04725 Cholinergic synapse;  map04726 Serotonergic synapse; map04727 GABAergic synapse; map04728 Dopaminergic synapse; map04926 Relaxin signaling pathway;  map05032 Morphine addiction; map05034 Alcoholism; map05163 Human cytomegalovirus infection; map05167 Kaposi sarcoma-associated herpesvirus infection;  map05170 Human immunodeficiency virus 1 infection; map05200 Pathways in cancer | | | S | KOG0286 | G-protein beta subunit |
| 23.014 | 40.7 | 7 | 7 | 10 | 0.554 | 0.7 | 0.778 | 2.354 | 1.818 | 1.093 | mitochondria | K04455 | HSPB1; heat shock protein beta-1 | map04010 MAPK signaling pathway; map04370 VEGF signaling pathway; map05146 Amoebiasis | | | O | KOG3591 | Alpha crystallins |
| 9.2908 | 27.7 | 2 | 2 | 18 | 1.496 | 1.486 | 1.443 | 0.732 | 0.679 | 0.632 | mitochondria | K02270 | COX7A; cytochrome c oxidase subunit 7a | map00190 Oxidative phosphorylation; map01100 Metabolic pathways; map04260 Cardiac muscle contraction; map04714 Thermogenesis;  map04932 Non-alcoholic fatty liver disease (NAFLD); map05010 Alzheimer disease; map05012 Parkinson disease; map05016 Huntington disease | | | | | |
| 153.97 | 6 | 7 | 7 | 8 | 1.092 | 1.592 | 1.221 | 0.886 | 0.812 | 0.672 | nucleus | K18995 | DHX29; ATP-dependent RNA helicase DHX29 [EC:3.6.4.13] | | PF00270; PF04408; PF07717 | DEAD/DEAH box helicase; Helicase associated domain (HA2); Oligonucleotide/oligosaccharide-binding (OB)-fold | A | KOG0920 | ATP-dependent RNA helicase A |
| 22.924 | 14.5 | 3 | 3 | 6 | 1.211 | 1.16 | 1.07 | 0.87 | 0.866 | 0.91 | mitochondria | K19054 | FXN; frataxin [EC:1.16.3.1] | map00860 Porphyrin and chlorophyll metabolism | PF01491 | Frataxin-like domain | P | KOG3413 | Mitochondrial matrix protein frataxin, involved in Fe/S protein biosynthesis |
| 13.918 | 15.4 | 3 | 2 | 4 | 0.59 | 0.614 | 0.652 | 1.08 | 1.208 | 1.359 | nucleus | K08341 | GABARAP, ATG8, LC3; GABA(A) receptor-associated protein | map04068 FoxO signaling pathway; map04136 Autophagy - other; map04137 Mitophagy - animal; map04140 Autophagy - animal; map04212 Longevity regulating pathway - worm; map04371 Apelin signaling pathway; map04621 NOD-like receptor signaling pathway; map04727 GABAergic synapse; map05131 Shigellosis; map05167 Kaposi sarcoma-associated herpesvirus infection | PF02991 | Autophagy protein Atg8 ubiquitin like | Z | KOG1654 | Microtubule-associated anchor protein involved in autophagy and membrane trafficking |
| 78.765 | 1.7 | 1 | 1 | 1 | 0.95 | 0.801 | 0.832 | 1.096 | 1.236 | 1.141 | nucleus | K12487 | GIT2; G protein-coupled receptor kinase interactor 2 | map04144 Endocytosis; map05135 Yersinia infection | PF01412; PF12205 | Putative GTPase activating protein for Arf; G protein-coupled receptor kinase-interacting protein 1 C term | T | KOG0818 | GTPase-activating proteins of the GIT family |
| 41.412 | 5.5 | 2 | 2 | 3 | 2.342 | 2.241 | 1.162 | 0.636 | 0.374 | 0.539 | cytoplasm | K15010 | HOMER; homer | map04068 FoxO signaling pathway; map04724 Glutamatergic synapse | PF00568 | WH1 domain | |  |  |
| 33.975 | 6.5 | 2 | 2 | 2 | 1.168 | 1.214 | 1.285 | 0.96 | 0.65 | 0.924 | mitochondria | K12474 | LDLRAP1, ARH; low density lipoprotein receptor adapter protein 1 | map04144 Endocytosis; map04979 Cholesterol metabolism | PF00640 | Phosphotyrosine interaction domain (PTB/PID) | T | KOG3536 | Adaptor protein CED-6, contains PTB domain |
| 39.24 | 4 | 1 | 1 | 1 | 0.737 | 0.436 | 0.52 | 1.185 | 1.866 | 1.57 | cytoplasm,nucleus | K10368 | CAPG; capping protein (actin filament), gelsolin-like | | PF00626 | Gelsolin repeat | Z | KOG0443 | Actin regulatory proteins (gelsolin/villin family) |
| 61.787 | 43.7 | 23 | 19 | 106 | 0.894 | 0.793 | 0.845 | 1.306 | 1.214 | 0.955 | extracellular | K01044 | CES1; carboxylesterase 1 [EC:3.1.1.1] | map00983 Drug metabolism - other enzymes | PF00135 | Carboxylesterase family | I | KOG1516 | Carboxylesterase and related proteins |
| 12.097 | 57.4 | 6 | 6 | 24 | 1.45 | 1.354 | 1.34 | 0.742 | 0.821 | 0.674 | extracellular |  |  |  | PF10660; PF09360 | Iron-containing outer mitochondrial membrane protein N-terminus ; Iron-binding zinc finger CDGSH type | S | KOG3461 | CDGSH-type Zn-finger containing protein |
| 54.017 | 7.1 | 3 | 3 | 3 | 0.842 | 0.82 | 0.655 | 1.068 | 1.051 | 1.102 | extracellular | K01314 | F10; coagulation factor X [EC:3.4.21.6] | map04610 Complement and coagulation cascades | PF00594; PF00008; PF00089 | Vitamin K-dependent carboxylation/gamma-carboxyglutamic (GLA) domain; EGF-like domain; Trypsin | | | |
| 55.302 | 8 | 4 | 3 | 5 | 1.222 | 1.383 | 1.131 | 1.046 | 0.655 | 0.704 | cytoplasm | K06067 | HDAC1_2; histone deacetylase 1/2 [EC:3.5.1.98] | map04110 Cell cycle; map04213 Longevity regulating pathway - multiple species; map04330 Notch signaling pathway; map04919 Thyroid hormone signaling pathway; map05016 Huntington disease; map05031 Amphetamine addiction; map05034 Alcoholism; map05165 Human papillomavirus infection; map05169 Epstein-Barr virus infection; map05200 Pathways in cancer; map05202 Transcriptional misregulation in cancer; map05203 Viral carcinogenesis; map05206 MicroRNAs in cancer; map05220 Chronic myeloid leukemia | PF00850 | Histone deacetylase domain | B | KOG1342 | Histone deacetylase complex, catalytic component RPD3 |
| 29.622 | 4.6 | 1 | 1 | 2 | 0.728 | 0.633 | 0.662 | 1.252 | 1.178 | 1.171 | cytoplasm | K18648 | ANP32E; acidic leucine-rich nuclear phosphoprotein 32 family member E | | | | D | KOG2739 | Leucine-rich acidic nuclear protein |
| 58.47 | 33.1 | 17 | 17 | 34 | 1.425 | 1.517 | 1.852 | 0.442 | 0.439 | 0.667 | endoplasmic reticulum | K07430 | CYP7B; 25/26-hydroxycholesterol 7alpha-hydroxylase [EC:1.14.14.29] | map00120 Primary bile acid biosynthesis; map00140 Steroid hormone biosynthesis | PF00067 | Cytochrome P450 | Q | KOG0684 | Cytochrome P450 |
| 35.327 | 37.5 | 10 | 10 | 36 | 1.286 | 1.294 | 1.259 | 0.711 | 0.86 | 0.757 | mitochondria | K00413 | CYC1, CYT1, petC; ubiquinol-cytochrome c reductase cytochrome c1 subunit | map00190 Oxidative phosphorylation; map01100 Metabolic pathways; map04260 Cardiac muscle contraction; map04714 Thermogenesis;  map04932 Non-alcoholic fatty liver disease (NAFLD); map05010 Alzheimer disease; map05012 Parkinson disease; map05016 Huntington disease | | | C | KOG3052 | Cytochrome c1 |
| 55.764 | 40.4 | 19 | 3 | 48 | 1.444 | 1.846 | 1.538 | 0.804 | 0.913 | 0.552 | endoplasmic reticulum | K07413 | CYP2C; cytochrome P450 family 2 subfamily C [EC:1.14.14.1] | map00140 Steroid hormone biosynthesis; map00590 Arachidonic acid metabolism; map00591 Linoleic acid metabolism; map00830 Retinol metabolism; map01100 Metabolic pathways; map04726 Serotonergic synapse; map04750 Inflammatory mediator regulation of TRP channels; map05204 Chemical carcinogenesis | PF00067 | Cytochrome P450 | Q | KOG0156 | Cytochrome P450 CYP2 subfamily |
| 10.35 | 57.3 | 4 | 2 | 14 | 1.155 | 1.113 | 1.143 | 0.842 | 0.903 | 0.875 | cytoplasm | K10418 | DYNLL; dynein light chain LC8-type | map04962 Vasopressin-regulated water reabsorption | PF01221 | Dynein light chain type 1 | Z | KOG3430 | Dynein light chain type 1 |
| 69.612 | 2.7 | 2 | 2 | 2 | 1.634 | 1.746 | 1.325 | 0.595 | 0.51 | 0.76 | plasma membrane | |  |  |  |  |  |  |  |
| 63.66 | 43.7 | 20 | 20 | 73 | 0.814 | 0.841 | 0.728 | 1.559 | 1.297 | 1.364 | cytoplasm | K12261 | HACL1; 2-hydroxyacyl-CoA lyase 1 [EC:4.1.-.-] | map04146 Peroxisome | PF02776; PF00205; PF02775 | Thiamine pyrophosphate enzyme, N-terminal TPP binding domain; Thiamine pyrophosphate enzyme, central domain; Thiamine pyrophosphate enzyme, C-terminal TPP binding domain | EH | KOG1185 | Thiamine pyrophosphate-requiring enzyme |
| 55.074 | 10 | 5 | 4 | 6 | 0.62 | 0.536 | 0.543 | 1.288 | 0.828 | 1.146 | cytoplasm | K06067 | HDAC1_2; histone deacetylase 1/2 [EC:3.5.1.98] | map04110 Cell cycle; map04213 Longevity regulating pathway - multiple species; map04330 Notch signaling pathway; map04919 Thyroid hormone signaling pathway; map05016 Huntington disease; map05031 Amphetamine addiction; map05034 Alcoholism; map05165 Human papillomavirus infection; map05169 Epstein-Barr virus infection; map05200 Pathways in cancer; map05202 Transcriptional misregulation in cancer; map05203 Viral carcinogenesis; map05206 MicroRNAs in cancer; map05220 Chronic myeloid leukemia | PF00850 | Histone deacetylase domain | B | KOG1342 | Histone deacetylase complex, catalytic component RPD3 |
| 24.893 | 34.4 | 8 | 7 | 12 | 0.788 | 0.724 | 0.806 | 1.161 | 1.072 | 0.956 | nucleus | K10802 | HMGB1; high mobility group protein B1 | map03410 Base excision repair; map04140 Autophagy - animal; map04217 Necroptosis | PF09011; PF00505 | Domain of unknown function (DUF1898); HMG (high mobility group) box | K | KOG0381 | HMG box-containing protein |
| 24.162 | 21 | 4 | 3 | 6 | 0.787 | 0.721 | 0.756 | 1.395 | 0.933 | 0.996 | nucleus | K11295 | HMGB2; high mobility group protein B2 | | PF09011; PF00505 | Domain of unknown function (DUF1898); HMG (high mobility group) box | K | KOG0381 | HMG box-containing protein |
| 112.76 | 4.7 | 4 | 4 | 4 | 1.316 | 1.224 | 1.229 | 0.968 | 0.738 | 1.053 | cytoplasm | K01099 | INPP5B_F; inositol polyphosphate 5-phosphatase INPP5B/F [EC:3.1.3.36] | map00562 Inositol phosphate metabolism; map01100 Metabolic pathways; map04070 Phosphatidylinositol signaling system | PF03372; PF00620 | Endonuclease/Exonuclease/phosphatase family; RhoGAP domain | T | KOG4270 | GTPase-activator protein |
| 11.344 | 12.6 | 1 | 1 | 1 | 2.107 | 1.913 | 2.006 | 0.654 | 0.521 | 0.542 | plasma membrane | |  |  |  |  |  |  |  |
| 78.301 | 62 | 40 | 40 | 113 | 0.58 | 0.662 | 0.56 | 2.254 | 1.291 | 1.63 | cytoplasm | K07514 | EHHADH; enoyl-CoA hydratase / 3-hydroxyacyl-CoA dehydrogenase / 3,2-trans-enoyl-CoA isomerase [EC:4.2.1.17 1.1.1.35 5.3.3.8] | map00071 Fatty acid degradation; map00280 Valine, leucine and isoleucine degradation; map00310 Lysine degradation; map00380 Tryptophan metabolism; map00410 beta-Alanine metabolism; map00640 Propanoate metabolism; map00650 Butanoate metabolism; map01100 Metabolic pathways; map01200 Carbon metabolism; map01212 Fatty acid metabolism; map03320 PPAR signaling pathway; map04146 Peroxisome | PF00378; PF02737; PF00725 | Enoyl-CoA hydratase/isomerase family; 3-hydroxyacyl-CoA dehydrogenase, NAD binding domain; 3-hydroxyacyl-CoA dehydrogenase, C-terminal domain | I | KOG1683 | Hydroxyacyl-CoA dehydrogenase/enoyl-CoA hydratase |
| 20.344 | 10.5 | 2 | 2 | 3 | 1.326 | 1.256 | 1.162 | 0.894 | 0.528 | 0.623 | extracellular |  |  |  |  |  | S | KOG4526 | Predicted membrane protein |
| 32.675 | 43.7 | 11 | 11 | 52 | 1.254 | 1.283 | 1.1 | 0.778 | 0.837 | 0.974 | cytoplasm,nucleus | K00552 | GNMT; glycine N-methyltransferase [EC:2.1.1.20] | map00260 Glycine, serine and threonine metabolism; map01100 Metabolic pathways | PF13847 | Methyltransferase domain | | |  |
| 120.64 | 1.9 | 2 | 2 | 2 | 0.698 | 0.772 | 0.767 | 1.095 | 1.282 | 1.048 | plasma membrane | K12321 | GUCY2D_E; guanylate cyclase 2D/E [EC:4.6.1.2] | map00230 Purine metabolism; map01100 Metabolic pathways; map04740 Olfactory transduction; map04744 Phototransduction | PF07714; PF07701; PF00211 | Protein tyrosine kinase; Heme NO binding associated; Adenylate and Guanylate cyclase catalytic domain | T | KOG1023 | Natriuretic peptide receptor, guanylate cyclase |
| 25.97 | 66.1 | 19 | 12 | 239 | 0.641 | 0.561 | 0.699 | 1.506 | 1.479 | 1.508 | cytoplasm | K00799 | GST, gst; glutathione S-transferase [EC:2.5.1.18] | map00480 Glutathione metabolism; map00980 Metabolism of xenobiotics by cytochrome P450; map00982 Drug metabolism - cytochrome P450; map00983 Drug metabolism - other enzymes; map01100 Metabolic pathways; map01524 Platinum drug resistance; map04212 Longevity regulating pathway - worm; map05200 Pathways in cancer; map05204 Chemical carcinogenesis; map05225 Hepatocellular carcinoma; map05418 Fluid shear stress and atherosclerosis | PF02798 | Glutathione S-transferase, N-terminal domain | O | KOG1695 | Glutathione S-transferase |
| 55.826 | 35.5 | 18 | 9 | 39 | 0.643 | 0.77 | 0.625 | 1.326 | 1.466 | 0.819 | endoplasmic reticulum | K07413 | CYP2C; cytochrome P450 family 2 subfamily C [EC:1.14.14.1] | map00140 Steroid hormone biosynthesis; map00590 Arachidonic acid metabolism; map00591 Linoleic acid metabolism; map00830 Retinol metabolism; map01100 Metabolic pathways; map04726 Serotonergic synapse; map04750 Inflammatory mediator regulation of TRP channels; map05204 Chemical carcinogenesis | PF00067 | Cytochrome P450 | Q | KOG0156 | Cytochrome P450 CYP2 subfamily |
| 11.922 | 39.8 | 3 | 3 | 9 | 0.911 | 0.833 | 0.882 | 1.187 | 1.244 | 1.163 | cytoplasm | K09568 | FKBP1; FK506-binding protein 1 [EC:5.2.1.8] | | PF00254 | FKBP-type peptidyl-prolyl cis-trans isomerase | O | KOG0544 | FKBP-type peptidyl-prolyl cis-trans isomerase |
| 15.878 | 57.8 | 9 | 2 | 201 | 1.636 | 1.895 | 1.859 | 0.663 | 0.706 | 0.675 | cytoplasm | K13823 | HBB; hemoglobin subunit beta | map05143 African trypanosomiasis; map05144 Malaria | PF00042 | Globin | C | KOG3378 | Globins and related hemoproteins |
| 36.389 | 23.9 | 5 | 4 | 13 | 0.767 | 0.591 | 0.791 | 0.974 | 0.973 | 1.139 | cytoplasm | K06856 | IGH; immunoglobulin heavy chain | map04020 Calcium signaling pathway; map04064 NF-kappa B signaling pathway; map04072 Phospholipase D signaling pathway; map04145 Phagosome; map04151 PI3K-Akt signaling pathway; map04640 Hematopoietic cell lineage; map04650 Natural killer cell mediated cytotoxicity; map04662 B cell receptor signaling pathway; map04664 Fc epsilon RI signaling pathway; map04666 Fc gamma R-mediated phagocytosis; map04672 Intestinal immune network for IgA production; map05130 Pathogenic Escherichia coli infection; map05140 Leishmaniasis; map05143 African trypanosomiasis; map05146 Amoebiasis; map05150 Staphylococcus aureus infection; map05152 Tuberculosis; map05169 Epstein-Barr virus infection; map05202 Transcriptional misregulation in cancer; map05310 Asthma; map05320 Autoimmune thyroid disease; map05322 Systemic lupus erythematosus; map05323 Rheumatoid arthritis; map05330 Allograft rejection; map05340 Primary immunodeficiency; map05414 Dilated cardiomyopathy (DCM); map05416 Viral myocarditis | PF07654 | Immunoglobulin C1-set domain | | |  |
| 71.194 | 1.3 | 1 | 1 | 1 | 0.465 | 0.587 | 0.393 | 1.137 | 1 | 1.369 | nucleus | K09275 | TFCP2; transcription factor CP2 and related proteins | | | | K | KOG4091 | Transcription factor |
| 37.778 | 23.4 | 7 | 7 | 21 | 0.954 | 0.864 | 0.826 | 1.164 | 1.164 | 1.16 | cytoplasm |  |  |  | PF00085; PF00462 | Thioredoxin; Glutaredoxin | O | KOG0911 | Glutaredoxin-related protein |
| 17 | 12.5 | 2 | 2 | 3 | 1.319 | 1.244 | 1.223 | 0.815 | 0.913 | 0.929 | extracellular | K12873 | BUD31, G10; bud site selection protein 31 | map03040 Spliceosome | PF01125 | G10 protein | K | KOG3404 | G10 protein/predicted nuclear transcription regulator |
| 80.085 | 3.9 | 3 | 3 | 3 | 0.498 | 0.417 | 0.418 | 1.787 | 0.797 | 1.608 | mitochondria | K03240 | EIF2B5; translation initiation factor eIF-2B subunit epsilon | map03013 RNA transport; map05168 Herpes simplex virus 1 infection | | | J | KOG1461 | Translation initiation factor 2B, epsilon subunit (eIF-2Bepsilon/GCD6) |
| 19.747 | 4.7 | 1 | 1 | 2 | 0.481 | 0.316 | 0.372 | 1.186 | 0.9 | 1.099 | plasma membrane | |  |  |  |  |  |  |  |
| 22.876 | 31.3 | 6 | 6 | 7 | 0.742 | 1.006 | 0.756 | 1.026 | 1.144 | 1.268 | extracellular | K17783 | ERV1, GFER, ALR; mitochondrial FAD-linked sulfhydryl oxidase [EC:1.8.3.2] | | | | O | KOG3355 | Mitochondrial sulfhydryl oxidase involved in the biogenesis of cytosolic Fe/S proteins |
| 85.643 | 2.2 | 1 | 1 | 5 | 1.146 | 1.171 | 1.306 | 0.718 | 0.851 | 1.05 | extracellular,plasma membrane | K06809 | CDH15, CDH3, CDH14; cadherin 15, M-cadherin | map04514 Cell adhesion molecules (CAMs) | PF00028 | Cadherin domain | S | KOG3594 | FOG: Cadherin repeats |
| 24.18 | 3.2 | 1 | 1 | 2 | 1.249 | 1.245 | 1.329 | 0.79 | 0.86 | 0.946 | nucleus | K14165 | K14165; atypical dual specificity phosphatase [EC:3.1.3.16 3.1.3.48] | | PF00782 | Dual specificity phosphatase, catalytic domain | V | KOG1716 | Dual specificity phosphatase |
| 25.147 | 21 | 4 | 4 | 8 | 0.691 | 0.75 | 0.772 | 1.158 | 1.147 | 1.296 | cytoplasm | K09570 | FKBP3; FK506-binding protein 3 [EC:5.2.1.8] | | PF00254 | FKBP-type peptidyl-prolyl cis-trans isomerase | O | KOG0544 | FKBP-type peptidyl-prolyl cis-trans isomerase |
| 10.071 | 70.9 | 7 | 7 | 21 | 1.338 | 1.473 | 1.349 | 0.779 | 0.75 | 0.852 | extracellular | K02267 | COX6B; cytochrome c oxidase subunit 6b | map00190 Oxidative phosphorylation; map01100 Metabolic pathways; map04260 Cardiac muscle contraction; map04714 Thermogenesis; map04932 Non-alcoholic fatty liver disease (NAFLD); map05010 Alzheimer disease; map05012 Parkinson disease; map05016 Huntington disease | PF02297 | Cytochrome oxidase c subunit VIb | C | KOG3057 | Cytochrome c oxidase, subunit VIb/COX12 |
| 62.469 | 27.6 | 12 | 10 | 29 | 0.738 | 0.794 | 1.012 | 1.213 | 1.368 | 1.116 | extracellular | K03927 | CES2; carboxylesterase 2 [EC:3.1.1.1 3.1.1.84 3.1.1.56] | map00983 Drug metabolism - other enzymes | PF00135 | Carboxylesterase family | I | KOG1516 | Carboxylesterase and related proteins |
| 60.18 | 23.2 | 9 | 9 | 11 | 0.771 | 0.767 | 0.886 | 1.068 | 1.155 | 1.134 | nucleus |  |  |  | PF00887; PF13897 | Acyl CoA binding protein; Golgi-dynamics membrane-trafficking | U | KOG3878 | Protein involved in maintenance of Golgi structure and ER-Golgi transport |
| 64.268 | 5.7 | 4 | 3 | 4 | 1.218 | 1.166 | 1.396 | 0.843 | 0.883 | 0.842 | cytoplasm | K01896 | ACSM; medium-chain acyl-CoA synthetase [EC:6.2.1.2] | map00650 Butanoate metabolism; map01100 Metabolic pathways | PF13193 | Domain of unknown function (DUF4009) | I | KOG1175 | Acyl-CoA synthetase |
| 26.776 | 7.1 | 1 | 1 | 1 | 0.759 | 0.632 | 0.774 | 1.125 | 1.532 | 1.249 | cytoplasm | K10768 | ALKBH6; alkylated DNA repair protein alkB homolog 6 [EC:1.14.11.-] | | PF13532 | 2OG-Fe(II) oxygenase superfamily | S | KOG3200 | Uncharacterized conserved protein |
| 59.744 | 18.8 | 9 | 9 | 9 | 1.12 | 1.133 | 1.089 | 0.887 | 0.76 | 0.877 | nucleus |  |  |  |  |  |  |  |  |
| 16.782 | 13.1 | 1 | 1 | 1 | 0.698 | 0.555 | 0.608 | 1.114 | 1.437 | 1.196 | extracellular | K06520 | CD99, MIC2; CD99 antigen | map04514 Cell adhesion molecules (CAMs); map04670 Leukocyte transendothelial migration | | | | | |
| 24.499 | 5.6 | 1 | 1 | 1 | 0.859 | 0.845 | 0.905 | 1.035 | 1.275 | 1.257 | cytoplasm | K22855 | EEF1AKMT1, EFM5; EEF1A lysine methyltransferase 1 [EC:2.1.1.-] | | | | J | KOG3350 | Uncharacterized conserved protein |
| 47.101 | 26.2 | 9 | 9 | 22 | 0.613 | 0.672 | 0.619 | 1.608 | 1.574 | 1.411 | extracellular | K01511 | ENTPD5_6; ectonucleoside triphosphate diphosphohydrolase 5/6 [EC:3.6.1.6] | map00230 Purine metabolism; map00240 Pyrimidine metabolism; map01100 Metabolic pathways | | | F | KOG1385 | Nucleoside phosphatase |
| 80.484 | 15.3 | 9 | 9 | 11 | 0.877 | 0.781 | 0.675 | 1.166 | 0.993 | 1.138 | nucleus |  |  |  |  |  |  |  |  |
| 42.435 | 27 | 9 | 9 | 12 | 0.804 | 0.749 | 0.831 | 0.975 | 1.163 | 1.119 | cytoplasm | K11434 | PRMT1; type I protein arginine methyltransferase [EC:2.1.1.319] | map04068 FoxO signaling pathway; map04922 Glucagon signaling pathway | PF13649 | Methyltransferase domain | KOT | KOG1499 | Protein arginine N-methyltransferase PRMT1 and related enzymes |
| 80.363 | 38.6 | 24 | 24 | 50 | 0.93 | 0.86 | 0.841 | 1.148 | 1.233 | 1.177 | cytoplasm | K00700 | GBE1, glgB; 1,4-alpha-glucan branching enzyme [EC:2.4.1.18] | map00500 Starch and sucrose metabolism; map01100 Metabolic pathways | PF02922; PF00128; PF02806 | Carbohydrate-binding module 48 (Isoamylase N-terminal domain); Alpha amylase, catalytic domain; Alpha amylase, C-terminal all-beta domain | G | KOG0470 | 1,4-alpha-glucan branching enzyme/starch branching enzyme II |
| 77.187 | 10.2 | 6 | 6 | 8 | 1.256 | 1.084 | 1.234 | 0.788 | 0.888 | 0.792 | plasma membrane | K05657 | ABCB10; ATP-binding cassette, subfamily B (MDR/TAP), member 10 | map02010 ABC transporters | PF00005 | ABC transporter | Q | KOG0058 | Peptide exporter, ABC superfamily |
| 46.595 | 17.6 | 6 | 1 | 11 | 1.152 | 1.527 | 1.556 | 1.02 | 0.853 | 0.679 | cytoskeleton | K00659 | BAAT; bile acid-CoA:amino acid N-acyltransferase [EC:2.3.1.65 3.1.2.2] | map00120 Primary bile acid biosynthesis; map00430 Taurine and hypotaurine metabolism; map01040 Biosynthesis of unsaturated fatty acids; map01100 Metabolic pathways; map04146 Peroxisome; map04976 Bile secretion | PF08840 | BAAT / Acyl-CoA thioester hydrolase C terminal | | | |
| 170.65 | 1.1 | 2 | 2 | 2 | 0.521 | 0.638 | 0.613 | 1.295 | 0.924 | 1.198 | nucleus | K11658 | BAZ1B, WSTF; bromodomain adjacent to zinc finger domain protein 1B | | PF10537; PF00628; PF00439 | ATP-utilising chromatin assembly and remodelling N-terminal; PHD-finger; Bromodomain | B | KOG1245 | Chromatin remodeling complex WSTF-ISWI, large subunit (contains heterochromatin localization, PHD and BROMO domains) |
| 115.79 | 1.6 | 1 | 1 | 1 | 0.932 | 0.92 | 0.854 | 1.11 | 1.347 | 1.192 | cytoplasm | K05871 | PTK2B, FAK2; focal adhesion kinase 2 [EC:2.7.10.2] | map04020 Calcium signaling pathway; map04062 Chemokine signaling pathway; map04072 Phospholipase D signaling pathway; map04650 Natural killer cell mediated cytotoxicity; map04670 Leukocyte transendothelial migration; map04912 GnRH signaling pathway; map05135 Yersinia infection; map05161 Hepatitis B; map05163 Human cytomegalovirus infection; map05170 Human immunodeficiency virus 1 infection | PF00373; PF07714; PF03623 | FERM central domain; Protein tyrosine kinase; Focal adhesion targeting region | T | KOG4257 | Focal adhesion tyrosine kinase FAK, contains FERM domain |
| 5.8378 | 44.2 | 3 | 3 | 7 | 1.332 | 1.287 | 1.12 | 0.81 | 0.81 | 0.854 | mitochondria | K02135 | ATPeF1E, ATP5E, ATP15; F-type H+-transporting ATPase subunit epsilon | map00190 Oxidative phosphorylation; map01100 Metabolic pathways; map04714 Thermogenesis; map05010 Alzheimer disease;  map05012 Parkinson disease; map05016 Huntington disease | | | C | KOG3495 | Mitochondrial F1F0-ATP synthase, subunit epsilon/ATP15 |
| 18.236 | 54.2 | 8 | 8 | 9 | 0.73 | 0.871 | 0.861 | 1.088 | 1.106 | 1.118 | mitochondria | K07560 | dtd, DTD; D-aminoacyl-tRNA deacylase [EC:3.1.1.96] | | | | J | KOG3323 | D-Tyr-tRNA (Tyr) deacylase |
| 17.725 | 31.6 | 4 | 4 | 6 | 1.462 | 1.454 | 1.391 | 0.728 | 0.748 | 0.762 | mitochondria | |  |  |  |  |  |  |  |
| 70.839 | 18.7 | 12 | 12 | 20 | 0.858 | 0.787 | 0.813 | 1.546 | 1.311 | 1.256 | mitochondria | K00624 | E2.3.1.7; carnitine O-acetyltransferase [EC:2.3.1.7] | map04146 Peroxisome | |  | I | KOG3717 | Carnitine O-acyltransferase CRAT |
| 63.352 | 16.8 | 8 | 4 | 40 | 1.076 | 1.269 | 1.078 | 0.812 | 0.909 | 0.851 | extracellular | K15743 | CES3_5; carboxylesterase 3/5 [EC:3.1.1.1] | | PF00135 | Carboxylesterase family | I | KOG1516 | Carboxylesterase and related proteins |
| 34.702 | 10.8 | 3 | 3 | 3 | 0.941 | 0.753 | 0.849 | 1.062 | 1.231 | 1.058 | nucleus | K14823 | EBP2, EBNA1BP2; rRNA-processing protein EBP2 | | | | A | KOG3080 | Nucleolar protein-like/EBNA1-binding protein |
| 141.97 | 1.2 | 1 | 1 | 1 | 0.353 | 0.624 | 0.479 | 0.85 | 0.802 | 1.04 | extracellular | K19719 | COL2A; collagen, type II, alpha | map04151 PI3K-Akt signaling pathway; map04510 Focal adhesion; map04512 ECM-receptor interaction; map04974 Protein digestion and absorption;  map05165 Human papillomavirus infection | | | W | KOG3544 | Collagens (type IV and type XIII), and related proteins |
| 56.781 | 32.2 | 15 | 1 | 45 | 0.234 | 0.188 | 0.216 | 2.489 | 3.431 | 0.479 | endoplasmic reticulum | K07411 | CYP2A; cytochrome P450 family 2 subfamily A [EC:1.14.14.1] | map00830 Retinol metabolism; map01100 Metabolic pathways | PF00067 | Cytochrome P450 | Q | KOG0156 | Cytochrome P450 CYP2 subfamily |
| 56.74 | 40.5 | 19 | 5 | 56 | 0.622 | 0.593 | 0.72 | 1.746 | 1.894 | 0.956 | endoplasmic reticulum | K07411 | CYP2A; cytochrome P450 family 2 subfamily A [EC:1.14.14.1] | map00830 Retinol metabolism; map01100 Metabolic pathways | PF00067 | Cytochrome P450 | Q | KOG0156 | Cytochrome P450 CYP2 subfamily |
| 247.23 | 2.4 | 5 | 5 | 6 | 1.142 | 1.105 | 1.173 | 0.924 | 0.536 | 0.76 | endoplasmic reticulum | K03902 | F5; coagulation factor V (labile factor) | map04610 Complement and coagulation cascades | PF07732; PF00754 | Multicopper oxidase; F5/8 type C domain | | | |
| 24.454 | 9 | 2 | 2 | 3 | 0.361 | 0.288 | 0.507 | 1.269 | 1.285 | 1.527 | extracellular | K10949 | KDELR; ER lumen protein retaining receptor | map05110 Vibrio cholerae infection | | | U | KOG3106 | ER lumen protein retaining receptor |
| 7.9972 | 56.9 | 3 | 3 | 6 | 1.288 | 1.308 | 1.319 | 0.809 | 0.718 | 0.674 | nucleus | K04347 | GNG12; guanine nucleotide-binding protein G(I)/G(S)/G(O) subunit gamma-12 | map04010 MAPK signaling pathway; map04014 Ras signaling pathway; map04062 Chemokine signaling pathway; map04151 PI3K-Akt signaling pathway; map04371 Apelin signaling pathway; map04713 Circadian entrainment; map04723 Retrograde endocannabinoid signaling; map04724 Glutamatergic synapse; map04725 Cholinergic synapse; map04726 Serotonergic synapse; map04727 GABAergic synapse; map04728 Dopaminergic synapse; map04810 Regulation of actin cytoskeleton; map04926 Relaxin signaling pathway; map05032 Morphine addiction; map05034 Alcoholism; map05163 Human cytomegalovirus infection; map05167 Kaposi sarcoma-associated herpesvirus infection; map05170 Human immunodeficiency virus 1 infection; map05200 Pathways in cancer | PF00631 | GGL domain | T | KOG4119 | G protein gamma subunit |
| 129.24 | 30.1 | 30 | 30 | 58 | 1.08 | 1.121 | 1.128 | 0.802 | 0.851 | 0.708 | plasma membrane | K01530 | E7.6.2.1; phospholipid-translocating ATPase [EC:7.6.2.1] | | | | P | KOG0206 | P-type ATPase |
| 164.62 | 63.9 | 87 | 84 | 908 | 1.144 | 1.076 | 1.137 | 0.807 | 0.832 | 0.883 | mitochondria | K01948 | CPS1; carbamoyl-phosphate synthase (ammonia) [EC:6.3.4.16] | map00220 Arginine biosynthesis; map00250 Alanine, aspartate and glutamate metabolism; map00910 Nitrogen metabolism; map01100 Metabolic pathways; map01200 Carbon metabolism; map01230 Biosynthesis of amino acids | PF00988; PF00117; PF02786; PF02787; PF02142 | Carbamoyl-phosphate synthase small chain, CPSase domain; Glutamine amidotransferase class-I; Carbamoyl-phosphate synthase L chain, ATP binding domain; Carbamoyl-phosphate synthetase large chain, oligomerisation domain; MGS-like domain | F | KOG0370 | Multifunctional pyrimidine synthesis protein CAD (includes carbamoyl-phophate synthetase, aspartate transcarbamylase, and glutamine amidotransferase) |
| 11.528 | 14.9 | 2 | 2 | 2 | 1.315 | 1.275 | 1.233 | 0.808 | 0.866 | 0.933 | cytoplasm | K11368 | ENY2, DC6, SUS1; enhancer of yellow 2 transcription factor | | | | K | KOG4479 | Transcription factor e(y)2 |
| 76.328 | 13.2 | 8 | 8 | 10 | 1.204 | 1.336 | 1.06 | 0.842 | 0.896 | 0.684 | extracellular |  |  |  |  |  |  |  |  |
| 12.352 | 40.5 | 3 | 3 | 10 | 1.309 | 1.289 | 1.281 | 0.749 | 0.837 | 0.938 | mitochondria | K02266 | COX6A; cytochrome c oxidase subunit 6a | map00190 Oxidative phosphorylation; map01100 Metabolic pathways; map04260 Cardiac muscle contraction; map04714 Thermogenesis;  map04932 Non-alcoholic fatty liver disease (NAFLD); map05010 Alzheimer disease; map05012 Parkinson disease; map05016 Huntington disease | | | C | KOG3469 | Cytochrome c oxidase, subunit VIa/COX13 |
| 79.481 | 46.4 | 32 | 32 | 121 | 0.833 | 0.948 | 0.741 | 1.596 | 1.26 | 1.294 | cytoplasm | K12405 | HSD17B4; (3R)-3-hydroxyacyl-CoA dehydrogenase / 3a,7a,12a-trihydroxy-5b-cholest-24-enoyl-CoA hydratase / enoyl-CoA hydratase 2 [EC:1.1.1.- 4.2.1.107 4.2.1.119] | map00120 Primary bile acid biosynthesis; map01040 Biosynthesis of unsaturated fatty acids; map01100 Metabolic pathways; map01212 Fatty acid metabolism; map04146 Peroxisome | PF00106; PF13452; PF01575 | short chain dehydrogenase; Metal-binding domain of MaoC dehydratase; MaoC like domain | | | |
| 37.988 | 2.2 | 1 | 1 | 1 | 0.84 | 0.969 | 0.748 | 1.045 | 1.155 | 1.153 | cytoplasm |  |  |  |  |  |  |  |  |
| 93.728 | 1.6 | 1 | 1 | 2 | 1.459 | 1.358 | 1.104 | 0.611 | 0.584 | 0.668 | nucleus |  |  |  |  |  |  |  |  |
| 13.17 | 83.9 | 8 | 8 | 16 | 0.887 | 0.825 | 0.834 | 1.045 | 1.136 | 1.158 | cytoplasm | K03873 | ELOB, TCEB2; elongin-B | map04066 HIF-1 signaling pathway; map04120 Ubiquitin mediated proteolysis; map05170 Human immunodeficiency virus 1 infection; map05200 Pathways in cancer; map05211 Renal cell carcinoma | PF00240 | Ubiquitin family | K | KOG4495 | RNA polymerase II transcription elongation factor Elongin/SIII, subunit elongin B |
| 11.309 | 9.8 | 1 | 1 | 4 | 1.474 | 1.844 | 1.6 | 0.668 | 0.725 | 0.722 | extracellular | K08758 | APOA2; apolipoprotein A-II | map03320 PPAR signaling pathway; map04979 Cholesterol metabolism | | | | | |
| 12.496 | 41.7 | 4 | 4 | 10 | 1.81 | 1.808 | 1.944 | 0.6 | 0.611 | 0.68 | mitochondria | K02131 | ATPeF0F6, ATP5J; F-type H+-transporting ATPase subunit 6 | map00190 Oxidative phosphorylation; map01100 Metabolic pathways; map04714 Thermogenesis; map05010 Alzheimer disease; map05012 Parkinson disease;  map05016 Huntington disease | | | C | KOG4634 | Mitochondrial F1F0-ATP synthase, subunit Cf6 (coupling factor 6) |
| 30.977 | 21.3 | 5 | 5 | 6 | 1.124 | 1.152 | 1.204 | 0.786 | 0.853 | 0.762 | cytoplasm,nucleus | K01764 | HCCS; cytochrome c heme-lyase [EC:4.4.1.17] | map00860 Porphyrin and chlorophyll metabolism | | | CO | KOG3996 | Holocytochrome c synthase/heme-lyase |
| 50.56 | 11.2 | 5 | 5 | 5 | 0.77 | 0.69 | 0.576 | 1.329 | 1.474 | 1.162 | mitochondria | K17361 | ACOT9; acyl-coenzyme A thioesterase 9 [EC:3.1.2.-] | | | | I | KOG2763 | Acyl-CoA thioesterase |
| 33.31 | 11.8 | 3 | 3 | 4 | 1.636 | 1.248 | 1.181 | 0.925 | 0.75 | 0.691 | extracellular |  |  |  |  |  |  |  |  |
| 55.496 | 32.3 | 13 | 13 | 23 | 1.462 | 1.269 | 0.957 | 0.807 | 0.859 | 0.833 | cytoplasm | K14286 | AGXT2L1, ETNPPL; ethanolamine-phosphate phospho-lyase [EC:4.2.3.2] | map00564 Glycerophospholipid metabolism; map01100 Metabolic pathways | PF00202 | Aminotransferase class-III | E | KOG1403 | Predicted alanine-glyoxylate aminotransferase |
| 46.719 | 24.2 | 9 | 9 | 12 | 0.928 | 1.554 | 1.329 | 0.724 | 0.786 | 0.511 | cytoplasm | K15485 | BCL2L13, BCL-RAMBO; Bcl-2-like protein 13 | map04137 Mitophagy - animal; map05134 Legionellosis | | | | |  |
| 43.267 | 43.5 | 14 | 14 | 39 | 0.779 | 0.831 | 0.804 | 1.52 | 1.2 | 1.224 | mitochondria | K13239 | ECI2, PECI; Delta3-Delta2-enoyl-CoA isomerase [EC:5.3.3.8] | map00071 Fatty acid degradation; map04146 Peroxisome | PF00887; PF00378 | Acyl CoA binding protein; Enoyl-CoA hydratase/isomerase family | I | KOG0817 | Acyl-CoA-binding protein |
| 26.51 | 32.3 | 7 | 7 | 12 | 0.935 | 0.805 | 0.821 | 1.221 | 1.181 | 1.164 | cytoplasm,nucleus | K04507 | CACYBP, SIP; calcyclin binding protein | map04310 Wnt signaling pathway | PF09032; PF04969; PF05002 | Siah interacting protein, N terminal ; CS domain; SGS domain | T | KOG3260 | Calcyclin-binding protein CacyBP |
| 82.545 | 7.8 | 3 | 3 | 3 | 0.738 | 0.702 | 0.728 | 1.181 | 0.939 | 1.234 | nucleus | K14824 | ERB1, BOP1; ribosome biogenesis protein ERB1 | | PF08145 | BOP1NT (NUC169) domain | J | KOG0650 | WD40 repeat nucleolar protein Bop1, involved in ribosome biogenesis |
| 22.197 | 63.6 | 9 | 9 | 34 | 0.757 | 0.747 | 0.865 | 1.207 | 1.361 | 1.219 | mitochondria | K05901 | BLVRB; biliverdin reductase / flavin reductase [EC:1.3.1.24 1.5.1.30] | map00740 Riboflavin metabolism; map00860 Porphyrin and chlorophyll metabolism; map01100 Metabolic pathways | PF13460 | NADH(P)-binding | |  |  |
| 25.414 | 27.1 | 6 | 6 | 13 | 1.456 | 1.478 | 1.378 | 0.814 | 0.782 | 0.808 | cytoplasm | K11539 | CBR4; carbonyl reductase 4 / 3-oxoacyl-[acyl-carrier protein] reductase beta subunit [EC:1.1.1.-] | map00061 Fatty acid biosynthesis; map01100 Metabolic pathways; map01212 Fatty acid metabolism | PF13561 | Enoyl-(Acyl carrier protein) reductase | Q | KOG1200 | Mitochondrial/plastidial beta-ketoacyl-ACP reductase |
| 57.783 | 34.9 | 17 | 15 | 45 | 1.404 | 1.289 | 1.236 | 0.703 | 0.735 | 0.626 | endoplasmic reticulum | K07418 | CYP2J; cytochrome P450 family 2 subfamily J [EC:1.14.14.1 1.14.14.73 1.14.14.74 1.14.14.75] | map00590 Arachidonic acid metabolism; map00591 Linoleic acid metabolism; map01100 Metabolic pathways; map04726 Serotonergic synapse; map04750 Inflammatory mediator regulation of TRP channels; map04913 Ovarian steroidogenesis | PF00067 | Cytochrome P450 | Q | KOG0156 | Cytochrome P450 CYP2 subfamily |
| 61.94 | 33 | 12 | 10 | 32 | 1.191 | 1.164 | 1.178 | 0.879 | 0.869 | 0.886 | endoplasmic reticulum | K03927 | CES2; carboxylesterase 2 [EC:3.1.1.1 3.1.1.84 3.1.1.56] | map00983 Drug metabolism - other enzymes | PF00135 | Carboxylesterase family | I | KOG1516 | Carboxylesterase and related proteins |
| 22.96 | 7.8 | 2 | 2 | 2 | 1.141 | 1.168 | 1.272 | 0.943 | 0.879 | 0.898 | extracellular | K02373 | FADD; FAS-associated death domain protein | map01524 Platinum drug resistance; map04210 Apoptosis; map04215 Apoptosis - multiple species; map04217 Necroptosis; map04620 Toll-like receptor signaling pathway; map04621 NOD-like receptor signaling pathway; map04622 RIG-I-like receptor signaling pathway; map04624 Toll and Imd signaling pathway; map04657 IL-17 signaling pathway; map04668 TNF signaling pathway; map05010 Alzheimer disease; map05130 Pathogenic Escherichia coli infection; map05142 Chagas disease (American trypanosomiasis); map05152 Tuberculosis; map05160 Hepatitis C; map05161 Hepatitis B; map05162 Measles; map05163 Human cytomegalovirus infection; map05164 Influenza A; map05165 Human papillomavirus infection; map05167 Kaposi sarcoma-associated herpesvirus infection; map05168 Herpes simplex virus 1 infection; map05169 Epstein-Barr virus infection; map05170 Human immunodeficiency virus 1 infection; map05200 Pathways in cancer | PF01335; PF00531 | Death effector domain; Death domain | | | |
| 35.866 | 42.1 | 14 | 14 | 36 | 1.432 | 1.566 | 1.413 | 0.785 | 0.697 | 0.778 | extracellular | K04524 | APOE; apolipoprotein E | map04979 Cholesterol metabolism; map05010 Alzheimer disease | PF01442 | Apolipoprotein A1/A4/E domain | | |  |
| 61.445 | 8.5 | 5 | 5 | 5 | 0.783 | 0.853 | 0.72 | 1.377 | 1.288 | 1.239 | nucleus |  |  |  |  |  |  |  |  |
| 25.36 | 11.6 | 2 | 2 | 3 | 0.796 | 0.795 | 0.729 | 1.159 | 1.222 | 1.131 | extracellular | K16143 | CRP; C-reactive protein | | PF00354 | Pentaxin family | |  |  |
| 29.922 | 5.4 | 1 | 1 | 3 | 0.618 | 0.713 | 0.874 | 1.109 | 1.161 | 1.021 | plasma membrane | K02262 | COX3; cytochrome c oxidase subunit 3 | map00190 Oxidative phosphorylation; map01100 Metabolic pathways; map04260 Cardiac muscle contraction; map04714 Thermogenesis;  map04932 Non-alcoholic fatty liver disease (NAFLD); map05010 Alzheimer disease; map05012 Parkinson disease; map05016 Huntington disease | | | C | KOG4664 | Cytochrome oxidase subunit III and related proteins |
| 58.121 | 21.1 | 15 | 14 | 22 | 1.362 | 1.116 | 1.054 | 0.895 | 0.75 | 0.586 | endoplasmic reticulum | K07424 | CYP3A; cytochrome P450 family 3 subfamily A [EC:1.14.14.1] | map00140 Steroid hormone biosynthesis; map00591 Linoleic acid metabolism; map00830 Retinol metabolism; map01100 Metabolic pathways; map05204 Chemical carcinogenesis | PF00067 | Cytochrome P450 | Q | KOG0158 | Cytochrome P450 CYP3/CYP5/CYP6/CYP9 subfamilies |
| 18.795 | 18.2 | 2 | 2 | 2 | 1.334 | 1.328 | 1.251 | 0.892 | 0.643 | 0.952 | extracellular | K16904 | DCTPP1; dCTP diphosphatase [EC:3.6.1.12] | map00240 Pyrimidine metabolism; map01100 Metabolic pathways | PF12643 | MazG-like family | |  |  |
| 78.756 | 7.9 | 3 | 3 | 3 | 0.81 | 0.762 | 0.985 | 1.125 | 1.027 | 1.26 | cytoplasm |  |  |  | PF08241; PF01564 | Methyltransferase domain; Spermine/spermidine synthase | E | KOG2352 | Predicted spermine/spermidine synthase |
| 71.683 | 3.4 | 2 | 2 | 2 | 0.876 | 1.032 | 0.866 | 1.414 | 1.13 | 1.249 | cytoplasm | K00803 | AGPS, agpS; alkyldihydroxyacetonephosphate synthase [EC:2.5.1.26] | map00565 Ether lipid metabolism; map01100 Metabolic pathways; map04146 Peroxisome | PF01565; PF02913 | FAD binding domain ; FAD linked oxidases, C-terminal domain | C | KOG1233 | Alkyl-dihydroxyacetonephosphate synthase |
| 12.473 | 66.1 | 7 | 7 | 10 | 0.855 | 0.836 | 0.912 | 1.064 | 1.194 | 1.243 | extracellular | K03872 | ELOC, TCEB1; elongin-C | map04066 HIF-1 signaling pathway; map04120 Ubiquitin mediated proteolysis; map05170 Human immunodeficiency virus 1 infection; map05200 Pathways in cancer; map05211 Renal cell carcinoma | PF03931 | Skp1 family, tetramerisation domain | K | KOG3473 | RNA polymerase II transcription elongation factor Elongin/SIII, subunit elongin C |
| 16.665 | 31.2 | 4 | 4 | 7 | 1.45 | 1.289 | 1.435 | 0.766 | 0.842 | 0.812 | cytoplasm |  |  |  |  |  | D | KOG4613 | Predicted component of DNA replication checkpoint response mechanism (S-M checkpoint) |
| 31.3 | 29.1 | 8 | 8 | 22 | 0.932 | 0.91 | 0.746 | 1.55 | 1.382 | 1.195 | cytoplasm | K13237 | DECR2; peroxisomal 2,4-dienoyl-CoA reductase [EC:1.3.1.34] | map04146 Peroxisome | PF13561 | Enoyl-(Acyl carrier protein) reductase | Q | KOG0725 | Reductases with broad range of substrate specificities |
| 63.869 | 10.7 | 6 | 6 | 12 | 1.478 | 1.305 | 1.254 | 0.67 | 0.746 | 0.673 | plasma membrane | K09521 | DNAJC1; DnaJ homolog subfamily C member 1 | map04141 Protein processing in endoplasmic reticulum | PF00226; PF00249 | DnaJ domain; Myb-like DNA-binding domain | O | KOG0724 | Zuotin and related molecular chaperones (DnaJ superfamily), contains DNA-binding domains |
| 22.101 | 18.7 | 3 | 3 | 4 | 1.161 | 1.015 | 1.161 | 0.924 | 0.816 | 0.795 | cytoplasm | K09525 | DNAJC5; DnaJ homolog subfamily C member 5 | map04141 Protein processing in endoplasmic reticulum | PF00226 | DnaJ domain | O | KOG0716 | Molecular chaperone (DnaJ superfamily) |
| 56.789 | 14.3 | 4 | 4 | 4 | 1.335 | 1.166 | 1.179 | 0.964 | 0.891 | 0.639 | plasma membrane | K11155 | DGAT1; diacylglycerol O-acyltransferase 1 [EC:2.3.1.20 2.3.1.75 2.3.1.76] | map00561 Glycerolipid metabolism; map00830 Retinol metabolism; map01100 Metabolic pathways; map04975 Fat digestion and absorption | | | I | KOG0380 | Sterol O-acyltransferase/Diacylglycerol O-acyltransferase |
| 78.798 | 1.5 | 1 | 1 | 1 | 0.369 | 0.267 | 0.413 | 2.325 | 1.546 | 0.883 | nucleus | K14433 | DQX1; ATP-dependent RNA helicase DQX1 [EC:3.6.4.13] | | PF04408; PF07717 | Helicase associated domain (HA2); Oligonucleotide/oligosaccharide-binding (OB)-fold | A | KOG0925 | mRNA splicing factor ATP-dependent RNA helicase |
| 62.829 | 6 | 4 | 4 | 5 | 0.889 | 0.872 | 0.878 | 1.303 | 1.125 | 1.301 | nucleus | K15027 | EIF2D; translation initiation factor 2D | | PF01253 | Translation initiation factor SUI1 | J | KOG2522 | Filamentous baseplate protein Ligatin, contains PUA domain |
| 15.126 | 48.5 | 7 | 7 | 19 | 1.393 | 1.238 | 1.166 | 0.921 | 0.808 | 0.821 | cytoplasm | K08751 | FABP2; fatty acid-binding protein 2, intestinal | map03320 PPAR signaling pathway; map04975 Fat digestion and absorption | PF00061 | Lipocalin / cytosolic fatty-acid binding protein family | I | KOG4015 | Fatty acid-binding protein FABP |
| 38.734 | 35 | 10 | 10 | 15 | 0.814 | 0.726 | 0.925 | 1.153 | 1.34 | 1.241 | cytoplasm | K17091 | ANXA1; annexin A1 | |  |  | U | KOG0819 | Annexin |
| 28.948 | 31.6 | 13 | 13 | 53 | 1.206 | 1.16 | 1.197 | 0.818 | 0.826 | 0.674 | mitochondria | K02127 | ATPeF0B, ATP5F1, ATP4; F-type H+-transporting ATPase subunit b | map00190 Oxidative phosphorylation; map01100 Metabolic pathways; map04714 Thermogenesis; map05010 Alzheimer disease;  map05012 Parkinson disease; map05016 Huntington disease | | | C | KOG3976 | Mitochondrial F1F0-ATP synthase, subunit b/ATP4 |
| 160.82 | 6.7 | 8 | 8 | 9 | 1.34 | 1.326 | 1.035 | 0.892 | 0.71 | 0.798 | nucleus | K14401 | CPSF1, CFT1; cleavage and polyadenylation specificity factor subunit 1 | map03015 mRNA surveillance pathway | PF10433 | Mono-functional DNA-alkylating methyl methanesulfonate N-term | A | KOG1896 | mRNA cleavage and polyadenylation factor II complex, subunit CFT1 (CPSF subunit) |
| 12.497 | 24.8 | 3 | 3 | 6 | 0.994 | 1.058 | 1.209 | 0.842 | 0.794 | 0.661 | plasma membrane | K12668 | OST2, DAD1; oligosaccharyltransferase complex subunit epsilon | map00510 N-Glycan biosynthesis; map00513 Various types of N-glycan biosynthesis; map01100 Metabolic pathways;  map04141 Protein processing in endoplasmic reticulum | | | DO | KOG1746 | Defender against cell death protein/oligosaccharyltransferase, epsilon subunit |
| 16.925 | 42.9 | 7 | 7 | 12 | 1.24 | 1.248 | 0.998 | 0.845 | 0.862 | 0.864 | extracellular |  |  |  |  |  |  |  |  |
| 47.624 | 23.7 | 9 | 9 | 13 | 1.187 | 1.24 | 1.386 | 0.863 | 0.759 | 0.841 | mitochondria | K14395 | ACP6; lysophosphatidic acid phosphatase type 6 [EC:3.1.3.2] | | | | I | KOG3720 | Lysosomal & prostatic acid phosphatases |
| 31.419 | 37.8 | 14 | 14 | 38 | 1.423 | 1.089 | 1.54 | 0.888 | 0.834 | 0.834 | cytoplasm | K02937 | RP-L7e, RPL7; large subunit ribosomal protein L7e | map03010 Ribosome | PF08079; PF00327 | Ribosomal L30 N-terminal domain; Ribosomal protein L30p/L7e | J | KOG3184 | 60S ribosomal protein L7 |
| 13.049 | 51.3 | 7 | 7 | 9 | 0.984 | 0.811 | 0.821 | 1.801 | 1.276 | 1.43 | cytoplasm | K21128 | S100A9; protein S100-A9 | map04657 IL-17 signaling pathway | PF01023 | S-100/ICaBP type calcium binding domain | | | |
| 34.737 | 7.8 | 3 | 3 | 4 | 0.89 | 0.882 | 0.757 | 1.166 | 1.262 | 1.177 | extracellular |  |  |  | PF03781 | Formylglycine-generating sulfatase enzyme | | | |
| 80.027 | 8.4 | 6 | 6 | 7 | 1.365 | 1.24 | 1.311 | 0.921 | 0.843 | 0.688 | peroxisome | K08955 | YME1; ATP-dependent metalloprotease [EC:3.4.24.-] | | PF00004; PF01434 | ATPase family associated with various cellular activities (AAA); Peptidase family M41 | O | KOG0734 | AAA+-type ATPase containing the peptidase M41 domain |
| 53.386 | 2.1 | 1 | 1 | 1 | 0.563 | 0.575 | 0.569 | 0.955 | 1.109 | 1.207 | nucleus | K10495 | ZBTB8; zinc finger and BTB domain-containing protein 8 | | PF00651; PF13909; PF00096 | BTB/POZ domain; C2H2-type zinc-finger domain; Zinc finger, C2H2 type | K | KOG1721 | FOG: Zn-finger |
| 31.992 | 11 | 3 | 3 | 3 | 1.157 | 1.103 | 1.321 | 0.85 | 0.747 | 0.764 | extracellular |  |  |  | PF01416 | tRNA pseudouridine synthase | J | KOG4393 | Predicted pseudouridylate synthase |
| 21.733 | 28.3 | 4 | 4 | 6 | 1.297 | 1.21 | 1.459 | 0.794 | 0.831 | 0.902 | extracellular | K02911 | RP-L32, MRPL32, rpmF; large subunit ribosomal protein L32 | map03010 Ribosome | |  | J | KOG4080 | Mitochondrial ribosomal protein L32 |
| 9.6871 | 12.5 | 2 | 2 | 2 | 1.18 | 1.148 | 1.142 | 0.966 | 0.638 | 0.785 | extracellular |  |  |  |  |  |  |  |  |
| 70.525 | 10.7 | 5 | 5 | 5 | 0.792 | 0.665 | 0.883 | 1.011 | 1.282 | 0.986 | cytoplasm | K23281 | VPS33B; vacuolar protein sorting-associated protein 33B | | | | U | KOG1302 | Vacuolar sorting protein VPS33/slp1 (Sec1 family) |
| 33.772 | 21 | 5 | 5 | 9 | 0.628 | 0.628 | 0.726 | 1.153 | 1.234 | 1.122 | cytoplasm | K12602 | WDR61, REC14, SKI8; WD repeat-containing protein 61 | map03018 RNA degradation | | | S | KOG4155 | FOG: WD40 repeat |
| 28.032 | 9.2 | 2 | 2 | 2 | 0.877 | 0.874 | 0.798 | 0.976 | 1.182 | 1.176 | cytoplasm | K22524 | WBP2; WW domain-binding protein 2 | | PF02893 | GRAM domain | K | KOG3294 | WW domain binding protein WBP-2, contains GRAM domain |
| 47.346 | 9.7 | 3 | 3 | 3 | 0.922 | 0.837 | 0.834 | 1.214 | 1.292 | 1.16 | nucleus | K14863 | WDR12, YTM1; ribosome biogenesis protein | | PF08154 | NLE (NUC135) domain | Z | KOG0313 | Microtubule binding protein YTM1 (contains WD40 repeats) |
| 43.843 | 4.7 | 2 | 2 | 2 | 0.853 | 0.809 | 0.755 | 0.928 | 1.164 | 1.169 | mitochondria | K17711 | PTCD2; pentatricopeptide repeat domain-containing protein 2 | | | | |  |  |
| 22.321 | 3.6 | 1 | 1 | 1 | 1.417 | 1.445 | 1.38 | 0.735 | 0.561 | 0.933 | mitochondria | K02963 | RP-S18, MRPS18, rpsR; small subunit ribosomal protein S18 | map03010 Ribosome | |  | J | KOG3162 | Mitochondrial/chloroplast ribosomal protein S18 |
| 36.614 | 12.3 | 4 | 4 | 4 | 0.942 | 0.774 | 0.8 | 1.004 | 1.104 | 1.208 | cytoplasm | K11413 | SIRT3, SIR2L3; NAD+-dependent protein deacetylase sirtuin 3 [EC:2.3.1.286] | map00760 Nicotinate and nicotinamide metabolism; map01100 Metabolic pathways; map05230 Central carbon metabolism in cancer | | | BK | KOG2682 | NAD-dependent histone deacetylases and class I sirtuins (SIR2 family) |
| 36.464 | 3.6 | 1 | 1 | 1 | 0.927 | 0.876 | 0.786 | 1.427 | 1.224 | 1.385 | plasma membrane | K19995 | SCAMP; secretory carrier-associated membrane protein | | | | U | KOG3088 | Secretory carrier membrane protein |
| 24.988 | 8 | 1 | 1 | 1 | 1.711 | 1.552 | 1.767 | 0.627 | 0.781 | 0.588 | plasma membrane | K18575 | TMEM86B; alkenylglycerophosphocholine hydrolase [EC:3.3.2.2] | map00565 Ether lipid metabolism | | | S | KOG4804 | Predicted membrane protein |
| 33.097 | 57.6 | 14 | 14 | 38 | 1.165 | 1.162 | 1.163 | 0.828 | 0.847 | 0.888 | mitochondria | K01011 | TST, MPST, sseA; thiosulfate/3-mercaptopyruvate sulfurtransferase [EC:2.8.1.1 2.8.1.2] | map00270 Cysteine and methionine metabolism; map00920 Sulfur metabolism; map01100 Metabolic pathways; map04122 Sulfur relay system | PF00581 | Rhodanese-like domain | V | KOG1529 | Mercaptopyruvate sulfurtransferase/thiosulfate sulfurtransferase |
| 85.893 | 1.3 | 1 | 1 | 1 | 2.023 | 1.703 | 1.81 | 0.57 | 0.513 | 0.681 | nucleus | K16812 | TPX2; targeting protein for Xklp2 | | PF09041 | Aurora-A binding | |  |  |
| 29.367 | 28.5 | 10 | 10 | 18 | 1.239 | 1.245 | 1.167 | 0.944 | 0.67 | 0.766 | mitochondria | K00411 | UQCRFS1, RIP1, petA; ubiquinol-cytochrome c reductase iron-sulfur subunit [EC:7.1.1.8] | map00190 Oxidative phosphorylation; map01100 Metabolic pathways; map04260 Cardiac muscle contraction; map04714 Thermogenesis; map04932 Non-alcoholic fatty liver disease (NAFLD); map05010 Alzheimer disease; map05012 Parkinson disease; map05016 Huntington disease | PF09165; PF00355 | Ubiquinol-cytochrome c reductase 8 kDa, N-terminal; Rieske [2Fe-2S] domain | C | KOG1671 | Ubiquinol cytochrome c reductase, subunit RIP1 |
| 109.73 | 6.1 | 5 | 5 | 7 | 0.778 | 0.788 | 0.943 | 1.056 | 1.077 | 1.238 | cytoplasm | K14288 | XPOT; exportin-T | map03013 RNA transport | | | JUY | KOG2021 | Nuclear mRNA export factor receptor LOS1/Exportin-t (importin beta superfamily) |
| 32.45 | 9.2 | 2 | 2 | 2 | 0.558 | 0.562 | 0.866 | 1.293 | 1.682 | 0.979 | cytoplasm | K01807 | rpiA; ribose 5-phosphate isomerase A [EC:5.3.1.6] | map00030 Pentose phosphate pathway; map01100 Metabolic pathways; map01200 Carbon metabolism; map01230 Biosynthesis of amino acids | | | G | KOG3075 | Ribose 5-phosphate isomerase |
| 34.732 | 17.6 | 7 | 7 | 8 | 0.785 | 0.906 | 0.787 | 1.377 | 1.074 | 1.218 | cytoplasm | K21198 | NAPG, SNAPG; gamma-soluble NSF attachment protein | | | | U | KOG1585 | Protein required for fusion of vesicles in vesicular transport, gamma-SNAP |
| 24.977 | 46.9 | 9 | 9 | 12 | 1.336 | 1.344 | 1.192 | 0.795 | 0.82 | 0.621 | plasma membrane | K12947 | SPCS2, SPC2; signal peptidase complex subunit 2 [EC:3.4.-.-] | map03060 Protein export | | | U | KOG4072 | Signal peptidase complex, subunit SPC25 |
| 136.97 | 3.5 | 3 | 3 | 3 | 1.108 | 1.189 | 0.921 | 0.889 | 0.74 | 0.758 | plasma membrane | K14289 | XPO5; exportin-5 | map03013 RNA transport | | | UY | KOG2020 | Nuclear transport receptor CRM1/MSN5 (importin beta superfamily) |
| 152.88 | 0.6 | 1 | 1 | 1 | 0.499 | 0.446 |  | 1.668 | 0.93 | 0.935 | cytoplasm |  |  |  | PF06467 | MYM-type Zinc finger with FCS sequence motif | | | |
| 204 | 1.2 | 2 | 2 | 2 | 0.88 | 0.738 | 0.701 | 1.467 | 1.126 | 1.324 | nucleus | K10386 | PPL; periplakin | |  |  | Z | KOG0516 | Dystonin, GAS (Growth-arrest-specific protein), and related proteins |
| 68.035 | 43.2 | 22 | 22 | 44 | 1.174 | 1.204 | 1.344 | 0.772 | 0.681 | 0.831 | mitochondria | K00318 | PRODH, fadM, putB; proline dehydrogenase [EC:1.5.5.2] | map00330 Arginine and proline metabolism; map01100 Metabolic pathways | | | E | KOG0186 | Proline oxidase |
| 21.298 | 32.8 | 5 | 1 | 6 | 1.053 | 1.059 | 1.227 | 0.841 | 0.873 | 0.743 | cytoplasm | K02833 | HRAS; GTPase HRas | map01521 EGFR tyrosine kinase inhibitor resistance; map01522 Endocrine resistance; map04010 MAPK signaling pathway; map04012 ErbB signaling pathway; map04014 Ras signaling pathway; map04015 Rap1 signaling pathway; map04062 Chemokine signaling pathway; map04068 FoxO signaling pathway; map04071 Sphingolipid signaling pathway; map04072 Phospholipase D signaling pathway; map04137 Mitophagy - animal; map04140 Autophagy - animal; map04144 Endocytosis; map04150 mTOR signaling pathway; map04151 PI3K-Akt signaling pathway; map04210 Apoptosis; map04211 Longevity regulating pathway; map04213 Longevity regulating pathway - multiple species; map04218 Cellular senescence; map04360 Axon guidance; map04370 VEGF signaling pathway; map04371 Apelin signaling pathway; map04510 Focal adhesion; map04540 Gap junction; map04550 Signaling pathways regulating pluripotency of stem cells; map04625 C-type lectin receptor signaling pathway; map04630 JAK-STAT signaling pathway; map04650 Natural killer cell mediated cytotoxicity; map04660 T cell receptor signaling pathway; map04662 B cell receptor signaling pathway; map04664 Fc epsilon RI signaling pathway; map04714 Thermogenesis; map04720 Long-term potentiation; map04722 Neurotrophin signaling pathway; map04725 Cholinergic synapse; map04726 Serotonergic synapse; map04730 Long-term depression; map04810 Regulation of actin cytoskeleton; map04910 Insulin signaling pathway; map04912 GnRH signaling pathway; map04915 Estrogen signaling pathway; map04916 Melanogenesis; map04917 Prolactin signaling pathway; map04919 Thyroid hormone signaling pathway; map04921 Oxytocin signaling pathway; map04926 Relaxin signaling pathway; map04929 GnRH secretion; map04933 AGE-RAGE signaling pathway in diabetic complications; map04935 Growth hormone synthesis, secretion and action; map05034 Alcoholism; map05160 Hepatitis C; map05161 Hepatitis B; map05163 Human cytomegalovirus infection; map05165 Human papillomavirus infection; map05166 Human T-cell leukemia virus 1 infection; map05167 Kaposi sarcoma-associated herpesvirus infection; map05170 Human immunodeficiency virus 1 infection; map05200 Pathways in cancer; map05203 Viral carcinogenesis; map05205 Proteoglycans in cancer; map05206 MicroRNAs in cancer; map05210 Colorectal cancer; map05211 Renal cell carcinoma; map05213 Endometrial cancer; map05214 Glioma; map05215 Prostate cancer; map05216 Thyroid cancer; map05218 Melanoma; map05219 Bladder cancer; map05220 Chronic myeloid leukemia; map05221 Acute myeloid leukemia; map05223 Non-small cell lung cancer; map05224 Breast cancer; map05225 Hepatocellular carcinoma; map05226 Gastric cancer; map05230 Central carbon metabolism in cancer; map05231 Choline metabolism in cancer; map05235 PD-L1 expression and PD-1 checkpoint pathway in cancer | PF00071 | Ras family | S | KOG0395 | Ras-related GTPase |
| 20.911 | 31.2 | 4 | 4 | 4 | 1.227 | 1.162 | 1.208 | 0.785 | 0.979 | 0.869 | cytoplasm | K07002 | K07002; uncharacterized protein | | PF06821 | Serine hydrolase | |  |  |
| 69.062 | 18.1 | 10 | 10 | 24 | 1.138 | 1.12 | 1.13 | 0.828 | 0.936 | 0.687 | extracellular | K12667 | SWP1, RPN2; oligosaccharyltransferase complex subunit delta (ribophorin II) | map00510 N-Glycan biosynthesis; map00513 Various types of N-glycan biosynthesis; map01100 Metabolic pathways;  map04141 Protein processing in endoplasmic reticulum | | | O | KOG2447 | Oligosaccharyltransferase, delta subunit (ribophorin II) |
| 55.325 | 17.5 | 10 | 10 | 17 | 0.823 | 0.815 | 0.792 | 1.158 | 1.05 | 1.173 | cytoplasm | K03341 | SEPSECS; O-phospho-L-seryl-tRNASec:L-selenocysteinyl-tRNA synthase [EC:2.9.1.2] | map00450 Selenocompound metabolism; map00970 Aminoacyl-tRNA biosynthesis; map01100 Metabolic pathways | PF05889 | Soluble liver antigen/liver pancreas antigen (SLA/LP autoantigen) | J | KOG3843 | Predicted serine hydroxymethyltransferase SLA/LP (autoimmune hepatitis marker in humans) |
| 90.641 | 3.4 | 3 | 3 | 4 | 0.735 | 0.653 | 0.827 | 1.003 | 0.887 | 1.213 | extracellular,plasma membrane | K23820 |  |  | PF03782; PF00084 | AMOP domain; Sushi domain (SCR repeat) | W | KOG4291 | Mucin/alpha-tectorin |
| 106.78 | 10 | 8 | 8 | 9 | 1.136 | 1.261 | 1.365 | 0.847 | 0.906 | 0.94 | mitochondria | K01872 | AARS, alaS; alanyl-tRNA synthetase [EC:6.1.1.7] | map00970 Aminoacyl-tRNA biosynthesis | PF07973 | Threonyl and Alanyl tRNA synthetase second additional domain | J | KOG0188 | Alanyl-tRNA synthetase |
| 80.597 | 19.6 | 17 | 16 | 30 | 1.127 | 1.121 | 1.141 | 0.78 | 0.905 | 0.59 | plasma membrane | K07151 | STT3; dolichyl-diphosphooligosaccharide---protein glycosyltransferase [EC:2.4.99.18] | map00510 N-Glycan biosynthesis; map00513 Various types of N-glycan biosynthesis; map01100 Metabolic pathways;  map04141 Protein processing in endoplasmic reticulum | | | O | KOG2292 | Oligosaccharyltransferase, STT3 subunit |
| 59.698 | 12 | 6 | 5 | 9 | 1.124 | 1.43 | 1.231 | 0.804 | 0.831 | 0.583 | plasma membrane | |  |  |  |  | CG | KOG1192 | UDP-glucuronosyl and UDP-glucosyl transferase |
| 56.471 | 18.8 | 6 | 6 | 7 | 0.882 | 0.874 | 0.734 | 1.28 | 1.256 | 1.122 | nucleus |  |  |  | PF00789 | UBX domain | S | KOG2507 | Ubiquitin regulatory protein UBXD2, contains UAS and UBX domains |
| 199.15 | 1.4 | 3 | 3 | 4 | 0.602 | 0.659 | 0.946 | 1.066 | 1.299 | 1.162 | nucleus | K10626 | UBR2; E3 ubiquitin-protein ligase UBR2 [EC:2.3.2.27] | | | | O | KOG1140 | N-end rule pathway, recognition component UBR1 |
| 11.48 | 16.5 | 1 | 1 | 1 | 1.292 | 1.138 | 1.403 | 0.887 | 0.991 | 0.909 | plasma membrane | K13505 | VAMP3; vesicle-associated membrane protein 3 | map04130 SNARE interactions in vesicular transport; map04145 Phagosome | | | U | KOG0860 | Synaptobrevin/VAMP-like protein |
| 43.096 | 18.7 | 6 | 6 | 9 | 0.904 | 0.786 | 0.85 | 1.193 | 1.105 | 1.235 | cytoplasm | K23167 | VAT1; synaptic vesicle membrane protein VAT-1 | | PF08240; PF13602 | Alcohol dehydrogenase GroES-like domain; Zinc-binding dehydrogenase | C | KOG1197 | Predicted quinone oxidoreductase |
| 31.068 | 9.4 | 2 | 2 | 2 | 0.759 | 0.86 | 0.874 | 1.364 | 1.06 | 1.19 | cytoplasm | K00876 | udk, UCK; uridine kinase [EC:2.7.1.48] | map00240 Pyrimidine metabolism; map00983 Drug metabolism - other enzymes; map01100 Metabolic pathways | PF00485 | Phosphoribulokinase / Uridine kinase family | TZ | KOG4203 | Armadillo/beta-Catenin/plakoglobin |
| 58.221 | 1.7 | 1 | 1 | 1 | 1.404 | 1.57 | 1.354 | 0.948 | 0.486 | 0.634 | plasma membrane | K11851 | USP30; ubiquitin carboxyl-terminal hydrolase 30 [EC:3.4.19.12] | map04137 Mitophagy - animal | | | O | KOG1867 | Ubiquitin-specific protease |
| 68.927 | 7.9 | 3 | 3 | 3 | 1.136 | 1.175 | 1.175 | 0.761 | 0.999 | 0.823 | plasma membrane | K17085 | TM9SF1; transmembrane 9 superfamily member 1 | | | | U | KOG1278 | Endosomal membrane proteins, EMP70 |
| 52.22 | 3.4 | 2 | 2 | 2 | 0.576 | 0.594 | 0.646 | 1.013 | 1.003 | 0.902 | cytoplasm |  |  |  | PF13695 | Zinc-binding domain | |  |  |
| 52.513 | 70.1 | 27 | 5 | 133 | 0.914 | 0.948 | 0.908 | 1.203 | 1.168 | 1.251 | cytoplasm | K17285 | SELENBP1; methanethiol oxidase [EC:1.8.3.4] | map00920 Sulfur metabolism; map01100 Metabolic pathways | | | P | KOG0918 | Selenium-binding protein |
| 47.69 | 27.5 | 10 | 10 | 18 | 1.357 | 1.378 | 1.061 | 0.959 | 0.842 | 0.847 | mitochondria | K18703 | SUGCT; succinate---hydroxymethylglutarate CoA-transferase [EC:2.8.3.13] | | | | I | KOG3957 | Predicted L-carnitine dehydratase/alpha-methylacyl-CoA racemase |
| 36.296 | 6 | 2 | 2 | 4 | 1.254 | 1.38 | 1.18 | 0.678 | 0.87 | 0.645 | plasma membrane | |  |  |  |  | S | KOG4491 | Predicted membrane protein |
| 87.194 | 8.9 | 8 | 8 | 10 | 1.02 | 1.416 | 1.355 | 0.806 | 0.875 | 0.725 | plasma membrane | K10106 | GGCX; vitamin K-dependent gamma-carboxylase [EC:4.1.1.90] | map00130 Ubiquinone and other terpenoid-quinone biosynthesis; map01100 Metabolic pathways | | | | | |
| 86.491 | 9.7 | 6 | 6 | 6 | 1.213 | 1.238 | 1.176 | 0.858 | 0.974 | 0.907 | nucleus | K22415 | ZC3H11; zinc finger CCCH domain-containing protein 11 | | | | S | KOG4791 | Uncharacterized conserved protein |
| 38.255 | 31.6 | 9 | 9 | 19 | 1.36 | 0.946 | 1.257 | 0.859 | 0.806 | 0.891 | mitochondria | K01480 | speB; agmatinase [EC:3.5.3.11] | map00330 Arginine and proline metabolism; map01100 Metabolic pathways | PF00491 | Arginase family | E | KOG2964 | Arginase family protein |
| 32.065 | 24.1 | 4 | 4 | 12 | 1.013 | 1.218 | 1.145 | 0.818 | 0.909 | 0.701 | endoplasmic reticulum | K13249 | SSR1; translocon-associated protein subunit alpha | map04141 Protein processing in endoplasmic reticulum | | | U | KOG1631 | Translocon-associated complex TRAP, alpha subunit |
| 19.587 | 12.2 | 2 | 2 | 2 | 1.11 | 1.07 | 1.205 | 0.857 | 0.843 | 0.734 | mitochondria | |  |  |  |  |  |  |  |
| 9.0788 | 10.1 | 1 | 1 | 2 | 1.366 | 1.557 | 1.438 | 0.692 | 0.83 | 0.61 | plasma membrane | |  |  |  |  | S | KOG4452 | Predicted membrane protein |
| 26.738 | 10.9 | 2 | 2 | 3 | 1.133 | 1.334 | 1.033 | 0.66 | 0.432 | 0.645 | plasma membrane | K17350 | TSPAN9; tetraspanin-9 | |  |  | S | KOG3882 | Tetraspanin family integral membrane protein |
| 42.125 | 2.1 | 1 | 1 | 1 | 1.131 | 1.14 | 1.294 | 0.953 | 0.698 | 0.738 | plasma membrane | K08716 | SLC14A; solute carrier family 14 (urea transporter) | | | | |  |  |
| 54.716 | 10.3 | 3 | 3 | 3 | 1.105 | 1.163 | 1.055 | 0.846 | 0.874 | 0.802 | plasma membrane | K14688 | SLC30A1, ZNT1; solute carrier family 30 (zinc transporter), member 1 | map04978 Mineral absorption | | | P | KOG1483 | Zn2+ transporter ZNT1 and related Cd2+/Zn2+ transporters (cation diffusion facilitator superfamily) |
| 85.965 | 4.6 | 4 | 4 | 4 | 0.708 | 0.922 | 0.848 | 1.126 | 0.963 | 1.155 | nucleus | K17608 | STRN1_3_4; striatin 1/3/4 | map04013 MAPK signaling pathway - fly | | | D | KOG0642 | Cell-cycle nuclear protein, contains WD-40 repeats |
| 38.384 | 34.6 | 9 | 9 | 21 | 0.82 | 0.797 | 0.831 | 1.15 | 1.115 | 1.219 | mitochondria | |  |  |  |  | C | KOG2620 | Prohibitins and stomatins of the PID superfamily |
| 67.083 | 29.4 | 14 | 13 | 25 | 0.789 | 0.819 | 0.772 | 1.347 | 1.086 | 1.289 | cytoplasm | K22182 | TXNRD; thioredoxin reductase (NADPH) [EC:1.8.1.9] | map00450 Selenocompound metabolism; map05200 Pathways in cancer; map05225 Hepatocellular carcinoma | PF07992; PF02852 | Pyridine nucleotide-disulphide oxidoreductase; Pyridine nucleotide-disulphide oxidoreductase, dimerisation domain | O | KOG4716 | Thioredoxin reductase |
| 35.039 | 53.1 | 19 | 19 | 115 | 0.884 | 0.876 | 0.712 | 1.424 | 1.104 | 1.38 | cytoplasm | K00365 | uaZ; urate oxidase [EC:1.7.3.3] | map00230 Purine metabolism; map00232 Caffeine metabolism; map01100 Metabolic pathways | PF01014 | Uricase | Q | KOG1599 | Uricase (urate oxidase) |
| 54.831 | 51.9 | 22 | 22 | 66 | 0.652 | 0.651 | 0.568 | 1.502 | 1.458 | 1.448 | cytoplasm | K00012 | UGDH, ugd; UDPglucose 6-dehydrogenase [EC:1.1.1.22] | map00040 Pentose and glucuronate interconversions; map00053 Ascorbate and aldarate metabolism; map00520 Amino sugar and nucleotide sugar metabolism; map01100 Metabolic pathways | PF03721; PF00984; PF03720 | UDP-glucose/GDP-mannose dehydrogenase family, NAD binding domain; UDP-glucose/GDP-mannose dehydrogenase family, central domain; UDP-glucose/GDP-mannose dehydrogenase family, UDP binding domain | GT | KOG2666 | UDP-glucose/GDP-mannose dehydrogenase |
| 26.146 | 4 | 1 | 1 | 1 | 0.809 | 0.666 | 0.661 | 1.119 | 1.189 | 1.29 | nucleus |  |  |  |  |  |  |  |  |
| 80.731 | 1.4 | 1 | 1 | 1 | 0.864 | 0.733 | 0.859 | 1.149 | 1.02 | 1.098 | nucleus | K04684 | SP1; transcription factor Sp1 | map01522 Endocrine resistance; map04137 Mitophagy - animal; map04350 TGF-beta signaling pathway; map04915 Estrogen signaling pathway; map04927 Cortisol synthesis and secretion; map04928 Parathyroid hormone synthesis, secretion and action; map04934 Cushing syndrome; map05016 Huntington disease; map05163 Human cytomegalovirus infection; map05200 Pathways in cancer; map05202 Transcriptional misregulation in cancer; map05224 Breast cancer; map05231 Choline metabolism in cancer | PF00096 | Zinc finger, C2H2 type | K | KOG1721 | FOG: Zn-finger |
| 53.687 | 60.7 | 30 | 24 | 56 | 0.788 | 0.667 | 0.706 | 1.041 | 1.824 | 1.208 | nucleus | K07606 | VIM; vimentin | map05169 Epstein-Barr virus infection; map05206 MicroRNAs in cancer | | | Z | KOG0977 | Nuclear envelope protein lamin, intermediate filament superfamily |
| 34.278 | 14.2 | 4 | 4 | 4 | 0.834 | 0.881 | 0.833 | 1.22 | 0.999 | 1.308 | cytoplasm | K17766 | TOM34; mitochondrial import receptor subunit TOM34 | | | | S | KOG1124 | FOG: TPR repeat |
| 11.451 | 26.7 | 3 | 3 | 5 | 1.521 | 1.514 | 1.359 | 0.686 | 0.78 | 0.639 | plasma membrane | K08512 | VAMP8; vesicle-associated membrane protein 8 | map04130 SNARE interactions in vesicular transport; map04140 Autophagy - animal; map04611 Platelet activation | | | U | KOG0860 | Synaptobrevin/VAMP-like protein |
| 17.274 | 5.4 | 1 | 1 | 1 | 0.867 | 0.774 | 0.798 | 1.318 | 0.997 | 1.258 | nucleus | K04381 | STMN1; stathmin | map04010 MAPK signaling pathway; map05206 MicroRNAs in cancer | | | Z | KOG1280 | Uncharacterized conserved protein containing ZZ-type Zn-finger |
| 83.056 | 7.7 | 5 | 5 | 6 | 0.731 | 0.72 | 0.77 | 1.104 | 0.967 | 1.043 | cytoplasm | K10885 | XRCC5, KU80, G22P2; ATP-dependent DNA helicase 2 subunit 2 | map03450 Non-homologous end-joining | PF03731; PF02735; PF08785 | Ku70/Ku80 N-terminal alpha/beta domain; Ku70/Ku80 beta-barrel domain; Ku C terminal domain like | L | KOG2326 | DNA-binding subunit of a DNA-dependent protein kinase (Ku80 autoantigen) |
| 36.127 | 49.7 | 15 | 15 | 27 | 0.879 | 0.874 | 0.927 | 1.288 | 1.239 | 0.977 | extracellular | K22267 | STBD1; starch-binding domain-containing protein 1 | | | | |  |  |
| 67.296 | 1.3 | 1 | 1 | 1 | 2.063 | 1.656 | 0.986 | 0.744 | 0.476 | 0.577 | plasma membrane | K10601 | SYVN1, HRD1; E3 ubiquitin-protein ligase synoviolin [EC:2.3.2.27] | map04120 Ubiquitin mediated proteolysis; map04141 Protein processing in endoplasmic reticulum | PF13639 | Ring finger domain | O | KOG0802 | E3 ubiquitin ligase |
| 58.004 | 31.1 | 15 | 15 | 30 | 0.883 | 0.78 | 0.879 | 1.139 | 1.224 | 1.214 | cytoplasm | K09498 | CCT6; T-complex protein 1 subunit zeta | | | | O | KOG0359 | Chaperonin complex component, TCP-1 zeta subunit (CCT6) |
| 46.172 | 5.9 | 3 | 3 | 3 | 0.792 | 0.81 | 0.753 | 1.174 | 0.962 | 1.254 | cytoplasm | K10582 | UBE2Q; ubiquitin-conjugating enzyme E2 Q [EC:2.3.2.23] | map04120 Ubiquitin mediated proteolysis | PF00179 | Ubiquitin-conjugating enzyme | O | KOG0897 | Predicted ubiquitin-conjugating enzyme |
| 122.61 | 3.1 | 3 | 3 | 3 | 0.785 | 0.999 | 0.865 | 1.162 | 1.25 | 1.045 | nucleus | K11839 | USP8, UBP5; ubiquitin carboxyl-terminal hydrolase 8 [EC:3.4.19.12] | map04137 Mitophagy - animal; map04144 Endocytosis; map04934 Cushing syndrome | PF08969; PF00581 | Domain of unknown function (DUF1873); Rhodanese-like domain | O | KOG1868 | Ubiquitin C-terminal hydrolase |
| 67.35 | 14.9 | 6 | 4 | 6 | 0.833 | 0.931 | 0.768 | 1.123 | 1.31 | 1.118 | nucleus | K04523 | UBQLN, DSK2; ubiquilin | map04141 Protein processing in endoplasmic reticulum | PF00240; PF00627 | Ubiquitin family; UBA/TS-N domain | O | KOG0010 | Ubiquitin-like protein |
| 35.24 | 20.1 | 6 | 6 | 8 | 1.374 | 1.368 | 1.418 | 0.893 | 0.594 | 0.743 | nucleus | K15085 | SLC25A42; solute carrier family 25, member 42 | | | | C | KOG0752 | Mitochondrial solute carrier protein |
| 13.77 | 27 | 4 | 3 | 4 | 0.438 | 0.46 | 0.44 | 0.748 | 0.647 | 0.636 | extracellular | K17310 | SAA; serum amyloid A protein | | |  |  |  |  |
| 12.51 | 39.1 | 4 | 4 | 4 | 0.682 | 0.801 | 0.706 | 1.134 | 1.129 | 1.287 | nucleus | K03104 | SRP14; signal recognition particle subunit SRP14 | map03060 Protein export | PF02290 | Signal recognition particle 14kD protein | U | KOG1761 | Signal recognition particle, subunit Srp14 |
| 173.09 | 1.4 | 2 | 2 | 2 | 3.137 | 2.249 | 3.252 | 0.145 | 0.089 | 0.189 | endoplasmic reticulum | K06252 | TN; tenascin | map04151 PI3K-Akt signaling pathway; map04510 Focal adhesion; map04512 ECM-receptor interaction; map05165 Human papillomavirus infection; map05206 MicroRNAs in cancer | PF00041; PF00147 | Fibronectin type III domain; Fibrinogen beta and gamma chains, C-terminal globular domain | T | KOG3544 | Collagens (type IV and type XIII), and related proteins |
| 32.316 | 5.7 | 2 | 1 | 3 | 0.651 | 0.692 | 0.746 | 1.308 | 1.289 | 1.098 | nucleus | K12897 | TRA2; transformer-2 protein | map03040 Spliceosome | PF00076 | RNA recognition motif. (a.k.a. RRM, RBD, or RNP domain) | A | KOG0118 | FOG: RRM domain |
| 31.413 | 2.5 | 1 | 1 | 1 | 0.475 | 0.168 | 0.216 | 1.672 | 0.522 | 1.313 | cytoplasm |  |  |  |  |  |  |  |  |
| 94.495 | 1.8 | 1 | 1 | 1 | 1.229 | 1.162 | 1.344 | 0.769 | 0.746 | 0.856 | plasma membrane | K16896 | TPCN1; two pore calcium channel protein 1 | map04020 Calcium signaling pathway | | | P | KOG2301 | Voltage-gated Ca2+ channels, alpha1 subunits |
| 46.415 | 42.9 | 13 | 13 | 31 | 0.891 | 0.941 | 0.848 | 1.211 | 1.187 | 1.102 | extracellular | K13984 | TXNDC5, ERP46; thioredoxin domain-containing protein 5 | map04141 Protein processing in endoplasmic reticulum | PF00085 | Thioredoxin | O | KOG0191 | Thioredoxin/protein disulfide isomerase |
| 50.051 | 32.3 | 12 | 2 | 43 | 0.702 | 0.826 | 0.81 | 1.148 | 1.121 | 1.184 | cytoskeleton | K07374 | TUBA; tubulin alpha | map04145 Phagosome; map04210 Apoptosis; map04530 Tight junction; map04540 Gap junction; map05130 Pathogenic Escherichia coli infection | PF00091; PF03953 | Tubulin/FtsZ family, GTPase domain; Tubulin C-terminal domain | Z | KOG1376 | Alpha tubulin |
| 32.134 | 5.1 | 1 | 1 | 3 | 0.638 | 0.698 | 0.805 | 1.056 | 1.064 | 1.064 | plasma membrane | K20362 | YIF1; protein transport protein YIF1 | | |  | S | KOG3094 | Predicted membrane protein |
| 22.314 | 20.2 | 5 | 5 | 7 | 1.268 | 1.213 | 1.095 | 0.988 | 0.771 | 0.823 | cytoplasm | K08516 | YKT6; synaptobrevin homolog YKT6 | map04130 SNARE interactions in vesicular transport | PF13774 | Regulated-SNARE-like domain | U | KOG0861 | SNARE protein YKT6, synaptobrevin/VAMP syperfamily |
| 59.69 | 49.7 | 21 | 21 | 83 | 0.829 | 0.999 | 0.77 | 1.118 | 1.154 | 1.339 | nucleus | K00863 | DAK, TKFC; triose/dihydroxyacetone kinase / FAD-AMP lyase (cyclizing) [EC:2.7.1.28 2.7.1.29 4.6.1.15] | map00051 Fructose and mannose metabolism; map00561 Glycerolipid metabolism; map01100 Metabolic pathways; map01200 Carbon metabolism;  map04622 RIG-I-like receptor signaling pathway | | | G | KOG2426 | Dihydroxyacetone kinase/glycerone kinase |
| 26.946 | 50.6 | 15 | 14 | 25 | 1.087 | 1.241 | 1.164 | 0.964 | 0.861 | 0.709 | mitochondria | K10707 | VAPB, ALS8; vesicle-associated membrane protein-associated protein B | map04979 Cholesterol metabolism | PF00635 | MSP (Major sperm protein) domain | U | KOG0439 | VAMP-associated protein involved in inositol metabolism |
| 11.042 | 49.5 | 4 | 4 | 13 | 1.518 | 1.455 | 1.477 | 0.858 | 0.825 | 0.885 | extracellular | K17780 | TIM8; mitochondrial import inner membrane translocase subunit TIM8 | | PF02953 | Tim10/DDP family zinc finger | U | KOG3489 | Mitochondrial import inner membrane translocase, subunit TIM8 |
| 25.488 | 3.3 | 1 | 1 | 2 | 1.182 | 1.19 | 1.178 | 0.759 | 0.742 | 0.752 | cytoplasm |  |  |  |  |  |  |  |  |
| 60.448 | 43 | 22 | 22 | 56 | 0.931 | 0.858 | 0.817 | 1.116 | 1.198 | 1.185 | cytoplasm | K09493 | CCT1, TCP1; T-complex protein 1 subunit alpha | | | | O | KOG0360 | Chaperonin complex component, TCP-1 alpha subunit (CCT1) |
| 56.202 | 4.4 | 2 | 2 | 2 | 1.137 | 1.013 | 1.158 | 0.839 | 0.788 | 0.823 | nucleus | K19944 | TBC1D10; TBC1 domain family member 10 | | | | U | KOG2221 | PDZ-domain interacting protein EPI64, contains TBC domain |
| 52.664 | 21.5 | 7 | 7 | 15 | 0.675 | 0.638 | 0.749 | 1.121 | 1.072 | 1.132 | extracellular |  |  |  | PF00112 | Papain family cysteine protease | S | KOG1544 | Predicted cysteine proteinase TIN-ag |
| 20.132 | 37.6 | 8 | 8 | 17 | 1.191 | 1.178 | 1.108 | 0.817 | 0.764 | 0.908 | extracellular | K22652 | TMEM25; transmembrane protein 25 | | | | T | KOG4555 | TPR repeat-containing protein |
| 27.544 | 2.8 | 1 | 1 | 1 | 0.761 | 0.767 | 0.525 | 1.171 | 1.058 | 0.912 | plasma membrane | K06571 | TSPAN7, TM4SF2, MXS1, CD231; tetraspanin-7 | map05202 Transcriptional misregulation in cancer | | | S | KOG3882 | Tetraspanin family integral membrane protein |
| 77.837 | 6.4 | 4 | 4 | 4 | 0.926 | 0.867 | 0.967 | 1.28 | 1.324 | 1.079 | extracellular | K17283 | LTF; lactotransferrin [EC:3.4.21.-] | | PF00405 | Transferrin |  |  |  |
| 65.444 | 33.4 | 18 | 18 | 31 | 1.122 | 1.375 | 1.202 | 0.614 | 0.706 | 0.993 | nucleus |  |  |  |  |  | S | KOG3783 | Uncharacterized conserved protein |
| 39.787 | 11.4 | 3 | 3 | 3 | 0.689 | 0.976 | 0.89 | 1.233 | 1.216 | 1.138 | cytoplasm,nucleus | K22991 | WDR45, WIPI4, WIPI3; WD repeat-containing protein 45 | | | | S | KOG2111 | Uncharacterized conserved protein, contains WD40 repeats |
| 32.014 | 47.5 | 11 | 11 | 20 | 0.859 | 0.804 | 0.828 | 1.217 | 1.098 | 1.09 | cytoplasm |  |  |  | PF03765; PF00650 | CRAL/TRIO, N-terminal domain; CRAL/TRIO domain | I | KOG1471 | Phosphatidylinositol transfer protein SEC14 and related proteins |
| 97.967 | 0.8 | 1 | 1 | 1 | 0.675 | 0.645 | 0.595 | 0.904 | 0.888 | 1.112 | cytoplasm | K05730 | VAV; guanine nucleotide exchange factor VAV | map04015 Rap1 signaling pathway; map04024 cAMP signaling pathway; map04062 Chemokine signaling pathway; map04510 Focal adhesion; map04650 Natural killer cell mediated cytotoxicity; map04660 T cell receptor signaling pathway; map04662 B cell receptor signaling pathway; map04664 Fc epsilon RI signaling pathway; map04666 Fc gamma R-mediated phagocytosis; map04670 Leukocyte transendothelial migration; map04810 Regulation of actin cytoskeleton; map05135 Yersinia infection; map05205 Proteoglycans in cancer | PF11971; PF00621; PF00169; PF00130; PF07653; PF00017; PF00018 | CAMSAP CH domain; RhoGEF domain; PH domain; Phorbol esters/diacylglycerol binding domain (C1 domain); Variant SH3 domain; SH2 domain; SH3 domain | T | KOG2996 | Rho guanine nucleotide exchange factor VAV3 |
| 42.469 | 3.9 | 1 | 1 | 1 | 1.446 | 1.212 | 1.228 | 0.87 | 0.948 | 0.822 | nucleus |  |  |  |  |  | S | KOG1188 | WD40 repeat protein |
| 102.73 | 1 | 1 | 1 | 1 | 1.268 | 1.318 | 1.105 | 1.041 | 0.816 | 0.803 | nucleus | K11848 | USP20_33; ubiquitin carboxyl-terminal hydrolase 20/33 [EC:3.4.19.12] | | PF02148; PF06337 | Zn-finger in ubiquitin-hydrolases and other protein; DUSP domain | O | KOG1870 | Ubiquitin C-terminal hydrolase |
| 30.752 | 47 | 10 | 10 | 29 | 1.156 | 1.274 | 1.138 | 0.798 | 0.932 | 0.808 | cytoplasm | K15041 | VDAC3; voltage-dependent anion channel protein 3 | map04020 Calcium signaling pathway; map04022 cGMP-PKG signaling pathway; map04216 Ferroptosis; map04217 Necroptosis;  map04218 Cellular senescence; map04621 NOD-like receptor signaling pathway; map04979 Cholesterol metabolism; map05012 Parkinson disease;  map05016 Huntington disease; map05161 Hepatitis B; map05166 Human T-cell leukemia virus 1 infection; map05203 Viral carcinogenesis | | | P | KOG3126 | Porin/voltage-dependent anion-selective channel protein |
| 49.536 | 1.7 | 1 | 1 | 1 | 3.119 | 2.432 | 1.47 | 0.384 | 0.264 | 0.391 | nucleus | K10496 | ZBTB9; zinc finger and BTB domain-containing protein 9 | | PF00651 | BTB/POZ domain | K | KOG1721 | FOG: Zn-finger |
| 49.988 | 8.3 | 4 | 1 | 10 | 0.63 | 0.673 | 0.571 | 1.19 | 1.02 | 1.057 | cytoskeleton | K07374 | TUBA; tubulin alpha | map04145 Phagosome; map04210 Apoptosis; map04530 Tight junction; map04540 Gap junction; map05130 Pathogenic Escherichia coli infection | PF00091; PF03953 | Tubulin/FtsZ family, GTPase domain; Tubulin C-terminal domain | Z | KOG1376 | Alpha tubulin |
| 52.363 | 12.2 | 5 | 5 | 8 | 0.923 | 0.862 | 0.827 | 1.318 | 1.418 | 1.02 | cytoplasm |  |  |  |  |  | S | KOG1398 | Uncharacterized conserved protein |
| 15.815 | 46.9 | 6 | 6 | 11 | 1.098 | 1.269 | 1.24 | 0.886 | 0.874 | 0.766 | cytoplasm | K20057 | SYNJ2BP, OMP25; synaptojanin-2-binding protein | | PF00595 | PDZ domain (Also known as DHR or GLGF) | S | KOG3528 | FOG: PDZ domain |
| 43.953 | 46.2 | 15 | 6 | 72 | 0.833 | 0.737 | 0.664 | 1.763 | 1.542 | 1.519 | mitochondria | K07513 | ACAA1; acetyl-CoA acyltransferase 1 [EC:2.3.1.16] | map00071 Fatty acid degradation; map00280 Valine, leucine and isoleucine degradation; map00592 alpha-Linolenic acid metabolism; map01040 Biosynthesis of unsaturated fatty acids; map01100 Metabolic pathways; map01212 Fatty acid metabolism; map03320 PPAR signaling pathway; map04146 Peroxisome | PF00108; PF02803 | Thiolase, N-terminal domain; Thiolase, C-terminal domain | I | KOG1389 | 3-oxoacyl CoA thiolase |
| 52.904 | 4.1 | 2 | 2 | 2 | 0.484 | 0.577 | 0.747 | 1.265 | 1.237 | 0.807 | cytoplasm | K15430 | TRM11, TRMT11; tRNA (guanine10-N2)-methyltransferase [EC:2.1.1.214] | | PF01170 | Putative RNA methylase family UPF0020 | L | KOG2671 | Putative RNA methylase |
| 12.605 | 42.9 | 5 | 5 | 7 | 1.257 | 1.085 | 1.096 | 0.884 | 0.758 | 0.946 | mitochondria | |  |  | PF00076 | RNA recognition motif. (a.k.a. RRM, RBD, or RNP domain) | A | KOG0118 | FOG: RRM domain |
| 60.597 | 8 | 4 | 4 | 5 | 1.228 | 1.183 | 1.082 | 0.77 | 0.864 | 0.519 | plasma membrane | K00637 | SOAT; sterol O-acyltransferase [EC:2.3.1.26] | map00100 Steroid biosynthesis; map04979 Cholesterol metabolism | | | I | KOG0380 | Sterol O-acyltransferase/Diacylglycerol O-acyltransferase |
| 32.68 | 21.5 | 7 | 2 | 5 | 1.765 | 2.086 | 2.316 | 0.542 | 0.439 | 0.628 | cytoplasm | K10373 | TPM1; tropomyosin 1 | map04260 Cardiac muscle contraction; map04261 Adrenergic signaling in cardiomyocytes; map05206 MicroRNAs in cancer;  map05410 Hypertrophic cardiomyopathy (HCM); map05414 Dilated cardiomyopathy (DCM) | | | Z | KOG1003 | Actin filament-coating protein tropomyosin |
| 15.437 | 13.7 | 2 | 2 | 2 | 1.486 | 1.589 | 1.418 | 0.699 | 0.581 | 0.903 | mitochondria | K02950 | RP-S12, MRPS12, rpsL; small subunit ribosomal protein S12 | map03010 Ribosome | |  | J | KOG1750 | Mitochondrial/chloroplast ribosomal protein S12 |
| 30.381 | 20.8 | 5 | 5 | 9 | 1.274 | 1.252 | 1.248 | 0.756 | 0.996 | 0.687 | plasma membrane | K20369 | ERV29, SURF4; ER-derived vesicles protein | | | | U | KOG3998 | Putative cargo transport protein ERV29 |
| 58.066 | 55.7 | 25 | 25 | 65 | 0.901 | 0.855 | 0.847 | 1.125 | 1.16 | 1.264 | cytoplasm | K09496 | CCT4; T-complex protein 1 subunit delta | | | | O | KOG0358 | Chaperonin complex component, TCP-1 delta subunit (CCT4) |
| 49.583 | 29.4 | 11 | 11 | 15 | 1.144 | 1.148 | 1.192 | 0.784 | 1.007 | 0.693 | extracellular | K23325 | TBL2; transducin beta-like protein 2 | | |  | S | KOG2096 | WD40 repeat protein |
| 16.284 | 18.6 | 3 | 3 | 3 | 1.03 | 1.163 | 1.213 | 0.779 | 0.936 | 0.797 | extracellular | K17770 | TOM20; mitochondrial import receptor subunit TOM20 | | | | U | KOG4056 | Translocase of outer mitochondrial membrane complex, subunit TOM20 |
| 10.458 | 62.1 | 5 | 5 | 10 | 1.756 | 1.562 | 1.704 | 0.686 | 0.664 | 0.718 | extracellular | K17781 | TIM13; mitochondrial import inner membrane translocase subunit TIM13 | | PF02953 | Tim10/DDP family zinc finger | U | KOG1733 | Mitochondrial import inner membrane translocase, subunit TIM13 |
| 145.27 | 8.3 | 9 | 9 | 10 | 0.718 | 0.931 | 0.778 | 1.205 | 0.955 | 1.084 | nucleus | K15542 | PFS2; polyadenylation factor subunit 2 | map03015 mRNA surveillance pathway | | | A | KOG0284 | Polyadenylation factor I complex, subunit PFS2 |
| 19.778 | 15.9 | 3 | 3 | 3 | 2.35 | 2.198 | 2.19 | 0.535 | 0.477 | 0.359 | plasma membrane | K05357 | VKORC1; vitamin-K-epoxide reductase (warfarin-sensitive) [EC:1.17.4.4] | map00130 Ubiquinone and other terpenoid-quinone biosynthesis; map01100 Metabolic pathways | PF07884 | Vitamin K epoxide reductase family | | |  |
| 30.959 | 21.2 | 6 | 6 | 7 | 0.598 | 0.667 | 0.606 | 1.581 | 1.449 | 1.458 | cytoplasm | K00355 | NQO1; NAD(P)H dehydrogenase (quinone) [EC:1.6.5.2] | map00130 Ubiquinone and other terpenoid-quinone biosynthesis; map01100 Metabolic pathways; map05200 Pathways in cancer; map05225 Hepatocellular carcinoma; map05418 Fluid shear stress and atherosclerosis | PF02525 | Flavodoxin-like fold | |  |  |
| 33.228 | 25.8 | 8 | 8 | 12 | 1.181 | 1.268 | 1.363 | 0.736 | 0.809 | 0.76 | cytoplasm,mitochondria | K15110 | SLC25A21, ODC; solute carrier family 25 (mitochondrial 2-oxodicarboxylate transporter), member 21 | | | | C | KOG0754 | Mitochondrial oxodicarboxylate carrier protein |
| 9.4771 | 38.1 | 3 | 1 | 11 | 0.583 | 0.589 | 0.927 | 1.252 | 1.235 | 1.279 | extracellular | K02978 | RP-S27e, RPS27; small subunit ribosomal protein S27e | map03010 Ribosome | |  | J | KOG1779 | 40s ribosomal protein S27 |
| 29.463 | 15.1 | 5 | 5 | 8 | 1.299 | 1.283 | 1.156 | 0.916 | 0.706 | 0.968 | mitochondria | K02956 | RP-S15, MRPS15, rpsO; small subunit ribosomal protein S15 | map03010 Ribosome | PF00312 | Ribosomal protein S15 | J | KOG2815 | Mitochondrial/choloroplast ribosomal protein S15 |
| 45.503 | 4.5 | 2 | 2 | 2 | 0.261 | 0.341 | 0.236 | 2.333 | 1.706 | 1.993 | nucleus |  |  |  |  |  |  |  |  |
| 31.555 | 6.5 | 2 | 2 | 2 | 0.789 | 0.654 | 0.555 | 0.98 | 0.862 | 1.016 | extracellular |  |  |  | PF00789 | UBX domain | T | KOG1363 | Predicted regulator of the ubiquitin pathway (contains UAS and UBX domains) |
| 31.732 | 45.4 | 12 | 12 | 27 | 1.22 | 1.27 | 1.133 | 0.772 | 0.962 | 0.734 | extracellular | K15040 | VDAC2; voltage-dependent anion channel protein 2 | map04020 Calcium signaling pathway; map04022 cGMP-PKG signaling pathway; map04216 Ferroptosis; map04217 Necroptosis;  map04218 Cellular senescence; map04621 NOD-like receptor signaling pathway; map04979 Cholesterol metabolism; map05012 Parkinson disease;  map05016 Huntington disease; map05166 Human T-cell leukemia virus 1 infection | | | P | KOG3126 | Porin/voltage-dependent anion-selective channel protein |
| 19.628 | 41.1 | 7 | 7 | 10 | 1.337 | 1.164 | 1.3 | 1.017 | 0.656 | 0.857 | mitochondria | K18160 | NDUFAF2; NADH dehydrogenase [ubiquinone] 1 alpha subcomplex assembly factor 2 | map04714 Thermogenesis | | |  |  |  |
| 16.815 | 8.1 | 1 | 1 | 3 | 1.439 | 1.633 | 1.493 | 0.59 | 0.741 | 0.57 | plasma membrane | |  |  |  |  | S | KOG3356 | Predicted membrane protein |
| 28.153 | 18.7 | 5 | 5 | 5 | 0.576 | 0.697 | 0.513 | 2.139 | 1.563 | 1.088 | mitochondria | K13351 | PEX11A; peroxin-11A | map04146 Peroxisome | |  | U | KOG4186 | Peroxisomal biogenesis protein (peroxin) |
| 45.58 | 11.8 | 5 | 5 | 6 | 1.401 | 1.186 | 1.265 | 0.885 | 0.996 | 0.878 | plasma membrane | K12275 | SEC62; translocation protein SEC62 | map03060 Protein export; map04141 Protein processing in endoplasmic reticulum | | | U | KOG2927 | Membrane component of ER protein translocation complex |
| 14.149 | 30.9 | 4 | 4 | 5 | 0.498 | 0.387 | 0.411 | 2.237 | 1.735 | 1.852 | cytoplasm,nucleus | K12260 | SRX1; sulfiredoxin [EC:1.8.98.2] | | |  | L | KOG3388 | Predicted transcription regulator/nuclease, contains ParB domain |
| 200.8 | 1.5 | 2 | 2 | 2 | 2.59 | 0.897 | 3.319 | 0.541 | 0.341 | 0.464 | nucleus |  |  |  |  |  | S | KOG0504 | FOG: Ankyrin repeat |
| 43.039 | 15.2 | 4 | 4 | 9 | 1.44 | 1.354 | 1.176 | 0.812 | 0.827 | 0.754 | plasma membrane | K14010 | TRAM1; translocating chain-associated membrane protein 1 | map04141 Protein processing in endoplasmic reticulum | PF03798 | TLC domain | U | KOG1608 | Protein transporter of the TRAM (translocating chain-associating membrane) superfamily |
| 56.613 | 12.2 | 5 | 4 | 5 | 0.855 | 0.835 | 0.808 | 1.074 | 1.49 | 1.199 | cytoplasm,nucleus | K00972 | UAP1; UDP-N-acetylglucosamine/UDP-N-acetylgalactosamine diphosphorylase [EC:2.7.7.23 2.7.7.83] | map00520 Amino sugar and nucleotide sugar metabolism; map01100 Metabolic pathways | | | M | KOG2388 | UDP-N-acetylglucosamine pyrophosphorylase |
| 100.46 | 8 | 6 | 2 | 7 | 0.772 | 0.787 | 0.901 | 1.212 | 1.003 | 1.13 | cytoplasm | K18727 | TNPO2, IPO3, KPNB2B; transportin-2 | | | | UY | KOG2023 | Nuclear transport receptor Karyopherin-beta2/Transportin (importin beta superfamily) |
| 32.994 | 28.4 | 10 | 5 | 17 | 1.294 | 1.39 | 1.548 | 0.72 | 0.806 | 0.942 | cytoplasm | K09290 | TPM3; tropomyosin 3 | map04260 Cardiac muscle contraction; map04261 Adrenergic signaling in cardiomyocytes; map05200 Pathways in cancer; map05216 Thyroid cancer;  map05410 Hypertrophic cardiomyopathy (HCM); map05414 Dilated cardiomyopathy (DCM) | | | Z | KOG1003 | Actin filament-coating protein tropomyosin |
| 72.082 | 3.8 | 2 | 2 | 2 | 0.696 | 0.777 | 0.742 | 0.942 | 1.168 | 1.078 | cytoplasm | K06670 | SCC1, MCD1, RAD21; cohesin complex subunit SCC1 | map04110 Cell cycle | |  | B | KOG1213 | Sister chromatid cohesion complex Cohesin, subunit RAD21/SCC1 |
| 39.706 | 22 | 8 | 6 | 16 | 0.937 | 0.774 | 0.704 | 1.098 | 1.029 | 1.085 | mitochondria | K10839 | RAD23, HR23; UV excision repair protein RAD23 | map03420 Nucleotide excision repair; map04141 Protein processing in endoplasmic reticulum | PF00240; PF00627; PF09280 | Ubiquitin family; UBA/TS-N domain; XPC-binding domain | L | KOG0011 | Nucleotide excision repair factor NEF2, RAD23 component |
| 101.68 | 60.2 | 43 | 43 | 136 | 1.15 | 1.184 | 1.211 | 0.8 | 0.781 | 0.828 | mitochondria | K00314 | SARDH; sarcosine dehydrogenase [EC:1.5.8.3] | map00260 Glycine, serine and threonine metabolism; map01100 Metabolic pathways | PF01266; PF01571; PF08669 | FAD dependent oxidoreductase; Aminomethyltransferase folate-binding domain; Glycine cleavage T-protein C-terminal barrel domain | E | KOG2844 | Dimethylglycine dehydrogenase precursor |
| 31.195 | 6.9 | 2 | 2 | 2 | 1.16 | 1.301 | 1.184 | 0.965 | 0.675 | 0.814 | cytoplasm | K13813 | STX12, STX13; syntaxin 12/13 | map04145 Phagosome | |  | HMU | KOG0811 | SNARE protein PEP12/VAM3/Syntaxin 7/Syntaxin 17 |
| 27.987 | 35 | 10 | 10 | 19 | 1.217 | 1.159 | 1.221 | 0.848 | 0.811 | 0.839 | mitochondria | K11830 | TFAM, MTTFA; transcription factor A, mitochondrial | map04371 Apelin signaling pathway; map05016 Huntington disease | PF00505; PF09011 | HMG (high mobility group) box; Domain of unknown function (DUF1898) | K | KOG0381 | HMG box-containing protein |
| 76.429 | 18.2 | 11 | 11 | 14 | 1.223 | 1.212 | 1.168 | 0.798 | 0.854 | 0.661 | plasma membrane | |  |  |  |  | S | KOG4467 | Uncharacterized conserved protein |
| 49.64 | 10.9 | 4 | 4 | 4 | 0.481 | 0.437 | 0.316 | 1.609 | 0.605 | 1.122 | cytoplasm,nucleus | K12004 | TRIM14; tripartite motif-containing protein 14 | | PF00643 | B-box zinc finger | |  |  |
| 33.931 | 26.7 | 9 | 9 | 23 | 1.3 | 1.233 | 1.269 | 0.816 | 0.816 | 0.691 | cytoplasm | K15100 | SLC25A1, CTP; solute carrier family 25 (mitochondrial citrate transporter), member 1 | | | | C | KOG0756 | Mitochondrial tricarboxylate/dicarboxylate carrier proteins |
| 18.431 | 43.7 | 9 | 9 | 20 | 1.224 | 1.136 | 1.166 | 0.928 | 0.832 | 0.944 | cytoplasm | K02949 | RP-S11e, RPS11; small subunit ribosomal protein S11e | map03010 Ribosome | PF00366 | Ribosomal protein S17 | J | KOG1728 | 40S ribosomal protein S11 |
| 34.732 | 15.8 | 4 | 4 | 4 | 2.264 | 2.347 | 2.166 | 0.379 | 0.506 | 0.417 | cytoplasm,nucleus | K17989 | SDS, SDH, CHA1; L-serine/L-threonine ammonia-lyase [EC:4.3.1.17 4.3.1.19] | map00260 Glycine, serine and threonine metabolism; map00270 Cysteine and methionine metabolism; map00290 Valine, leucine and isoleucine  biosynthesis; map01100 Metabolic pathways; map01200 Carbon metabolism; map01230 Biosynthesis of amino acids | | | E | KOG1250 | Threonine/serine dehydratases |
| 20.313 | 17.8 | 3 | 3 | 9 | 1.078 | 1.154 | 1.186 | 0.78 | 1.034 | 0.766 | extracellular | K12948 | SPCS3, SPC3; signal peptidase complex subunit 3 [EC:3.4.-.-] | map03060 Protein export | | | U | KOG3372 | Signal peptidase complex subunit |
| 32.64 | 20.2 | 4 | 4 | 7 | 1.297 | 1.414 | 1.102 | 0.949 | 0.892 | 0.661 | nucleus |  |  |  |  |  | S | KOG3944 | Uncharacterized conserved protein |
| 17.364 | 5.7 | 1 | 1 | 2 | 1.356 | 1.334 | 1.173 | 0.88 | 0.582 | 0.903 | nucleus | K11095 | SNRPC; U1 small nuclear ribonucleoprotein C | map03040 Spliceosome | PF06220 | U1 zinc finger | A | KOG3454 | U1 snRNP-specific protein C |
| 52.609 | 76.5 | 28 | 6 | 137 | 1.492 | 1.597 | 2.11 | 0.44 | 0.535 | 0.595 | cytoplasm | K17285 | SELENBP1; methanethiol oxidase [EC:1.8.3.4] | map00920 Sulfur metabolism; map01100 Metabolic pathways | | | P | KOG0918 | Selenium-binding protein |
| 36.141 | 14.6 | 5 | 5 | 13 | 1.542 | 1.508 | 1.46 | 0.636 | 0.478 | 0.6 | cytoplasm | K23501 | SFXN2; sideroflexin-2 | |  |  | S | KOG3767 | Sideroflexin |
| 11.186 | 27.8 | 3 | 3 | 5 | 0.609 | 0.505 | 0.701 | 1.084 | 1.068 | 1.411 | cytoplasm | K17274 | S100A10; protein S100-A10 | | PF01023 | S-100/ICaBP type calcium binding domain | | | |
| 40.421 | 2.8 | 1 | 1 | 2 | 1.361 | 1.457 | 1.192 | 0.93 | 0.45 | 0.769 | nucleus |  |  |  |  |  | K | KOG4594 | Sequence-specific single-stranded-DNA-binding protein |
| 73.964 | 4 | 3 | 1 | 4 | 1.236 | 1.704 | 1.334 | 0.624 | 0.362 | 0.504 | plasma membrane | K03460 | SLCO1A; solute carrier organic anion transporter family, member 1A | map04976 Bile secretion | PF07648 | Kazal-type serine protease inhibitor domain | P | KOG3626 | Organic anion transporter |
| 23.574 | 7.3 | 2 | 2 | 2 | 1.067 | 1.15 | 1.351 | 0.896 | 0.964 | 0.822 | cytoplasm |  |  |  | PF05517 | p25-alpha | T | KOG4070 | Putative signal transduction protein p25 |
| 134.07 | 3 | 3 | 3 | 4 | 0.596 | 0.767 | 0.61 | 1.188 | 1.066 | 1.148 | nucleus |  |  |  |  |  | D | KOG2152 | Sister chromatid cohesion protein |
| 17.768 | 12.4 | 2 | 2 | 2 | 1.601 | 1.469 | 1.473 | 0.818 | 0.894 | 0.662 | plasma membrane | K05357 | VKORC1; vitamin-K-epoxide reductase (warfarin-sensitive) [EC:1.17.4.4] | map00130 Ubiquinone and other terpenoid-quinone biosynthesis; map01100 Metabolic pathways | PF07884 | Vitamin K epoxide reductase family | | |  |
| 50.656 | 6.3 | 3 | 3 | 5 | 1.306 | 1.196 | 1.212 | 0.824 | 0.692 | 0.652 | plasma membrane | K14713 | SLC39A7, KE4, ZIP7; solute carrier family 39 (zinc transporter), member 7 | | | | P | KOG2693 | Putative zinc transporter |
| 21.482 | 11.6 | 2 | 2 | 4 | 1.502 | 1.635 | 1.402 | 0.811 | 0.694 | 0.719 | endoplasmic reticulum | K16338 | SDC4; syndecan 4 | map04512 ECM-receptor interaction; map04514 Cell adhesion molecules (CAMs); map05205 Proteoglycans in cancer;  map05418 Fluid shear stress and atherosclerosis | | | | | |
| 10.333 | 24.4 | 3 | 3 | 4 | 1.622 | 1.695 | 1.677 | 0.686 | 0.648 | 0.726 | extracellular | K17778 | TIM10; mitochondrial import inner membrane translocase subunit TIM10 | | PF02953 | Tim10/DDP family zinc finger | U | KOG3480 | Mitochondrial import inner membrane translocase, subunits TIM10/TIM12 |
| 57.091 | 7.4 | 3 | 3 | 4 | 0.515 | 0.657 | 0.643 | 1.463 | 1.496 | 1.12 | plasma membrane | K08069 | VNN; pantetheine hydrolase [EC:3.5.1.92] | map00770 Pantothenate and CoA biosynthesis | | | E | KOG0806 | Carbon-nitrogen hydrolase |
| 55.273 | 2.1 | 1 | 1 | 1 | 0.422 | 0.387 | 0.452 | 0.92 | 0.957 | 1.343 | nucleus |  |  |  | PF00618 | RasGEF N-terminal motif | T | KOG3541 | Predicted guanine nucleotide exchange factor |
| 16.445 | 76 | 16 | 16 | 36 | 1.208 | 1.184 | 1.198 | 0.909 | 0.8 | 0.917 | cytoplasm | K02960 | RP-S16e, RPS16; small subunit ribosomal protein S16e | map03010 Ribosome | |  | J | KOG1753 | 40S ribosomal protein S16 |
| 43.846 | 53.1 | 19 | 19 | 53 | 0.942 | 1.01 | 0.762 | 1.328 | 1.09 | 1.253 | cytoplasm,nucleus | K00306 | PIPOX; sarcosine oxidase / L-pipecolate oxidase [EC:1.5.3.1 1.5.3.7] | map00260 Glycine, serine and threonine metabolism; map00310 Lysine degradation; map01100 Metabolic pathways; map04146 Peroxisome | PF01266 | FAD dependent oxidoreductase | E | KOG2820 | FAD-dependent oxidoreductase |
| 54.748 | 18 | 8 | 8 | 9 | 1.114 | 1.043 | 1.33 | 0.848 | 0.814 | 0.776 | plasma membrane | K10142 | STEAP3, TSAP6; metalloreductase STEAP3 [EC:1.16.1.-] | map04115 p53 signaling pathway; map04216 Ferroptosis | | | | |  |
| 37.829 | 15.6 | 5 | 5 | 9 | 0.795 | 0.81 | 0.816 | 1.377 | 1.265 | 1.08 | extracellular | K22990 | TOR1; torsin-1 | |  |  | O | KOG2170 | ATPase of the AAA+ superfamily |
| 72.349 | 2.9 | 1 | 1 | 1 | 1.491 | 1.283 | 1.52 | 0.909 | 0.866 | 0.894 | mitochondria | K00555 | TRMT1, trm1; tRNA (guanine26-N2/guanine27-N2)-dimethyltransferase [EC:2.1.1.215 2.1.1.216] | | | | J | KOG1253 | tRNA methyltransferase |
| 28.467 | 25.8 | 9 | 7 | 8 | 1.585 | 1.634 | 1.31 | 0.788 | 0.548 | 0.992 | cytoplasm | K10375 | TPM4; tropomyosin 4 | map04260 Cardiac muscle contraction; map04261 Adrenergic signaling in cardiomyocytes; map05410 Hypertrophic cardiomyopathy (HCM);  map05414 Dilated cardiomyopathy (DCM) | | | Z | KOG1003 | Actin filament-coating protein tropomyosin |
| 43.182 | 3 | 1 | 1 | 1 | 1.229 | 1.393 | 1.326 | 0.797 | 0.889 | 0.788 | plasma membrane | |  |  | PF03798 | TLC domain | U | KOG1608 | Protein transporter of the TRAM (translocating chain-associating membrane) superfamily |
| 49.795 | 6.1 | 3 | 3 | 3 | 1.73 | 1.756 | 1.792 | 0.735 | 0.462 | 0.72 | cytoplasm | K14011 | UBXN6, UBXD1; UBX domain-containing protein 6 | map04141 Protein processing in endoplasmic reticulum | PF09409; PF00789 | PUB domain; UBX domain | O | KOG2699 | Predicted ubiquitin regulatory protein |
| 73.118 | 2.6 | 2 | 2 | 2 | 0.419 | 0.397 | 0.375 | 1.877 | 0.547 | 1.306 | nucleus |  |  |  |  |  | S | KOG0772 | Uncharacterized conserved protein, contains WD40 repeat |
| 275.75 | 14.1 | 30 | 23 | 44 | 0.894 | 0.877 | 0.796 | 1.022 | 1.224 | 1.423 | plasma membrane | K01946 | ACACB; acetyl-CoA carboxylase / biotin carboxylase 2 [EC:6.4.1.2 6.3.4.14 2.1.3.15] | map00061 Fatty acid biosynthesis; map00620 Pyruvate metabolism; map00640 Propanoate metabolism; map01100 Metabolic pathways; map04152 AMPK signaling pathway; map04910 Insulin signaling pathway; map04920 Adipocytokine signaling pathway; map04922 Glucagon signaling pathway; map04931 Insulin resistance | PF00289; PF02786; PF02785; PF00364 | Carbamoyl-phosphate synthase L chain, N-terminal domain; Carbamoyl-phosphate synthase L chain, ATP binding domain; Biotin carboxylase C-terminal domain; Biotin-requiring enzyme | I | KOG0368 | Acetyl-CoA carboxylase |
| 46.48 | 23.3 | 8 | 5 | 16 | 0.728 | 0.719 | 0.567 | 2.15 | 1.519 | 1.18 | cytoplasm | K01068 | ACOT1_2_4; acyl-coenzyme A thioesterase 1/2/4 [EC:3.1.2.2] | map00062 Fatty acid elongation; map01040 Biosynthesis of unsaturated fatty acids; map01100 Metabolic pathways | PF08840 | BAAT / Acyl-CoA thioester hydrolase C terminal | | | |
| 19.727 | 19 | 4 | 4 | 4 | 0.837 | 0.695 | 0.733 | 0.936 | 1.268 | 1.178 | cytoplasm |  |  |  | PF01951 | Archease protein family (MTH1598/TM1083) | S | KOG4528 | Uncharacterized conserved protein |
| 9.6963 | 30.7 | 4 | 4 | 8 | 1.979 | 2.219 | 1.956 | 0.633 | 0.516 | 0.574 | extracellular | K22286 | APOC1; apolipoprotein C-I | map04979 Cholesterol metabolism | | |  |  |  |
