# Supplementary material for: Psoraleae Fructus Ethanol Extract Induced Hepatotoxicity via Impaired Lipid Metabolism Caused by Disruption of Fatty Acid β-Oxidation
Source: Oxid Med Cell Longev. 2023 Jan 7;2023:4202861. doi: 10.1155/2023/4202861 (PMC9840557; doi:10.1155/2023/4202861)
Supplement: Supplementary Materials — Figure S1: expressions of 92 metabolites in PFE group. Table S1: chemical composition identification of PFE. Table S2: table of 2-level metabolites information. Table S3: table of differential proteins information. [file 4202861.f1.zip › Table S1.docx]

Tables S1 Chemical composition identification of PFE

| t_R_(/min) | Molecular formula | Detection mode | Theoretical value *m/z* | Measured value *m/z* |  (ppm) | Second-level debris | Compound | Chemical family |
| --- | --- | --- | --- | --- | --- | --- | --- | --- |
| 21.18 | C_11_H_6_O_3_ | + | 186.0317 | 186.0305 | 6.69 | 187.03807，143.04840 | Psoralen | Coumarins |
| 22.58 | C_11_H_6_O_3_ | + | 186.0317 | 186.0304 | 6.75 | 187.03806，131.04849 | Isopsoralen | Coumarins |
| 22.35 | C_20_H_16_O_5_ | - | 336.0998 | 336.0994 | 1.2 | 335.09265 | Psoralidin | Coumarins |
| 13.15 | C_11_H_6_O_4_ | - | 202.0266 | 202.0264 | 1.04 | 201.01921，110.96990, 88.98798 | Xanthotoxol | Coumarins |
| 34.09 | C_21_H_22_O_4_ | - | 338.1518 | 338.1514 | 1.24 | 119.05014 | 8-Geranyloxy psoralen | Coumarins |
| 23.16 | C_20_H_16_O_6_ | - | 352.0947 | 352.0943 | 0.99 | 351.08762，292.03766 | Bavacoumestan B | Coumarins |
| 30.21 | C_20_H_16_O_5_ | - | 336.0998 | 336.0994 | 1.18 | 335.09280，280.03748 | Isopsoralidin | Coumarins |
| 18.48 | C_20_H_16_O_7_ | - | 368.0896 | 368.0893 | 0.91 | 281.04541，367.08191，349.07187 | Corylidin | Coumarins |
| 32.77 | C_20_H_14_O_5_ | - | 334.0841 | 334.084 | 0.34 | 335.08994 | Sophoracoumestan A | Coumarins |
| 28.89 | C_21_H_22_O_5_ | - | 354.1467 | 354.1466 | 0.32 | 119.05012，353.14053 | Epoxybergamottin | Coumarins |
| 21.42 | C_20_H_16_O_6_ | - | 352.0947 | 352.0943 | 1.02 | 351.08734，321.07669，280.03650， | Bavacoumestan A | Coumarins |
| 33.07 | C_20_H_14_O_5_ | - | 334.0841 | 334.0841 | 0.1 | 335.08994 | Plicadin | Coumarins |
| 32.53 | C_25_H_24_O_5_ | - | 404.1624 | 404.1621 | 0.63 | 403.15381，266.05835 | Ficusin A | Coumarins |
| 26.49 | C_21_H_18_O_5_ | - | 350.1154 | 350.1151 | 0.96 | 349.10831，245.11821，305.08191 | 5-(4-Phenoxybutoxy)psoralen | Coumarins |
| 18.19 | C_12_H_8_O_4_ | - | 216.0423 | 216.0423 | -0.09 | 217.04846，202.02504 | 5-Methoxypsoralen | Coumarins |
| 32.98 | C_20_H_20_O_4_ | - | 324.1362 | 324.134 | 6.66 | 202.99443，118.94389,159.06677 | Bavachin | Flavonoids |
| 26.92 | C_20_H_18_O_4_ | - | 322.1205 | 322.12 | 1.49 | 321.11319，265.05072 | Neobavaisoflavone | Flavonoids |
| 37.37 | C_21_H_22_O_4_ | - | 338.1518 | 338.1514 | 1.27 | 119.05014，337.14496 | Bavachinin | Flavonoids |
| 35.09 | C_25_H_26_O_4_ | - | 390.1831 | 390.1827 | 0.95 | 389.17621，333.11340 | Corylifol A | Flavonoids |
| 27.92 | C_20_H_20_O_4_ | - | 324.1362 | 324.1356 | 1.7 | 119.05013，203.07133， 221.08194，323.12897 | Isobavachin | Flavonoids |
| 29.25 | C_20_H_16_O_4_ | - | 320.1049 | 320.1044 | 1.31 | 319.09756 | Corylin | Flavonoids |
| 25.66 | C_20_H_20_O_5_ | - | 340.1311 | 340.1305 | 1.81 | 119.05012，339.12369 | Bakuchalcone | Flavonoids |
| 29.92 | C_20_H_20_O_4_ | - | 324.1362 | 324.1356 | 1.7 | 119.05016，203.07140， 221.08189，323.12903， 159.08153 | Bavachalcone | Flavonoids |
| 25.54 | C_20_H_20_O_4_ | - | 324.1362 | 324.1357 | 1.54 | 119.05013，203.07133， 221.08183，323.12891， 159.08145 | Isobavachalcone | Flavonoids |
| 33.85 | C_21_H_22_O_4_ | - | 338.1518 | 338.1514 | 1.27 | 119.05014，337.14496 | Bavachinin A | Flavonoids |
| 27.35 | C_20_H_18_O_4_ | - | 322.1205 | 322.12 | 1.49 | 321.11319， 65.05072 | Isoneobavaisoflavone | Flavonoids |
| 30.60 | C_20_H_20_O_5_ | - | 340.1311 | 340.1305 | 1.81 | 135.04512，203.07137，221.08186，339.12357 | Bavachromanol | Flavonoids |
| 36.19 | C_20_H_18_O_4_ | - | 322.1205 | 322.12 | 1.52 | 119.05013，201.05559，321.11322 | Isobavachromene | Flavonoids |
| 37.59 | C_21_H_22_O_4_ | - | 338.1518 | 338.1514 | 1.21 | 119.05011 | 4'-O-Methylbroussochalcone B | Flavonoids |
| 23.79 | C_20_H_20_O_6_ | - | 356.126 | 356.1258 | 0.48 | 135.04509，235.06114，355.11887 | Brosimacutin G | Flavonoids |
| 23.80 | C_20_H_18_O_5_ | - | 338.1154 | 338.1152 | 0.75 | 337.10834，282.05304，133.02934 | Psoralenol | Flavonoids |
| 31.57 | C_20_H_20_O_4_ | - | 324.1362 | 324.1356 | 1.64 | 119.05014，203.07133，221.08189，323.12872，159.08148 | Isobavachalcone | Flavonoids |
| 35.09 | C_20_H_18_O_4_ | - | 322.1205 | 322.12 | 1.58 | 119.05014，201.05565，321.11325 | Bavachromene | Flavonoids |
| 26.69 | C_17_H_14_O_5_ | - | 298.0841 | 298.0839 | 0.86 | 119.05013,151.03996, 297.07767 | Neobavachalcone | Flavonoids |
| 19.95 | C_16_H_10_O_5_ | - | 282.0528 | 282.0527 | 0.3 | 281.0455 | Corylinal | Flavonoids |
| 25.26 | C_17_H_14_O_5_ | - | 298.0841 | 298.084 | 0.45 | 119.05013,151.03996, 297.07767 | Isoneobavachalcone | Flavonoids |
| 29.72 | C_22_H_24_O_4_ | - | 352.1675 | 352.1673 | 0.51 | 336.10046，351.12405，281.04535，293.04550，319.09732 | 4'-O-Methylbavachalcone | Flavonoids |
| 10.61 | C_21_H_20_O_10_ | - | 432.1057 | 432.1054 | 0.56 | 268.03778，431.09842 | Isovitexin | Flavonoids |
| 41.47 | C_18_H_24_O | - | 256.1827 | 256.1809 | 7.16 | 172.08931，255.17546 | Bakuchiol | Phenols |
| 37.79 | C_18_H_24_O_2_ | - | 272.1776 | 272.1773 | 1.4 | 188.08417，271.17035 | 3-Hydroxybakuchiol | Phenols |
| 38.02 | C_18_H_24_O_2_ | - | 272.1776 | 272.1773 | 1.4 | 188.08417，271.17035 | 12-Hydroxyisobakuchiol | Phenols |
| 26.65 | C_18_H_24_O_3_ | - | 288.1725 | 288.1725 | 0.26 | 188.08418，287.16516 | Psoracorylifol A | Phenols |
| 28.72 | C_18_H_24_O_3_ | - | 288.1725 | 288.1725 | 0.26 | 287.16534，229.12331，214.09978，150.03232 | Psoracorylifol B | Phenols |
| 31.51 | C_18_H_24_O_3_ | - | 288.1725 | 288.1725 | 0.26 | 149.02431，287.16528，182.02205，93.03448 | Psoracorylifol C | Phenols |
| 15.59 | C_13_H_14_O_4_ | - | 234.0892 | 234.0891 | 0.47 | 163.03996，233.08189，96.96950，135.04506，215.07162 | Isocorylifonol | Phenols |
| 10.32 | C_17_H_18_O_9_ | - | 366.0951 | 366.0944 | 1.79 | 159.04504，203.03500，59.01386 | Psoralenoside | Glycosides |
| 10.84 | C_17_H_18_O_9_ | - | 366.0951 | 366.0944 | 1.87 | 159.04506，203.03500， 59.01384，89.02444，71.01385 | Isopsoralenoside | Glycosides |
| 13.87 | C_21_H_20_O_11_ | - | 448.1006 | 448.1003 | 0.5 | 284.03262，174.95590，447.09344，104.95374， | Astragalin | Glycosides |
| 11.55 | C_27_H_30_O_15_ | - | 594.1585 | 594.1588 | -0.56 | 383.07727，413.08780，473.10883，593.15155，503.11935，533.13062 | Isosaponarin | Glycosides |
| 10.07 | C_27_H_30_O_16_ | - | 610.1534 | 610.1534 | -0.07 | 284.03256, 255.02980 | Lutonarin | Glycosides |
