# Supplementary figures and images for: Psoraleae Fructus Ethanol Extract Induced Hepatotoxicity via Impaired Lipid Metabolism Caused by Disruption of Fatty Acid β-Oxidation
Source: Oxid Med Cell Longev. 2023 Jan 7;2023:4202861. doi: 10.1155/2023/4202861 (PMC9840557; doi:10.1155/2023/4202861)

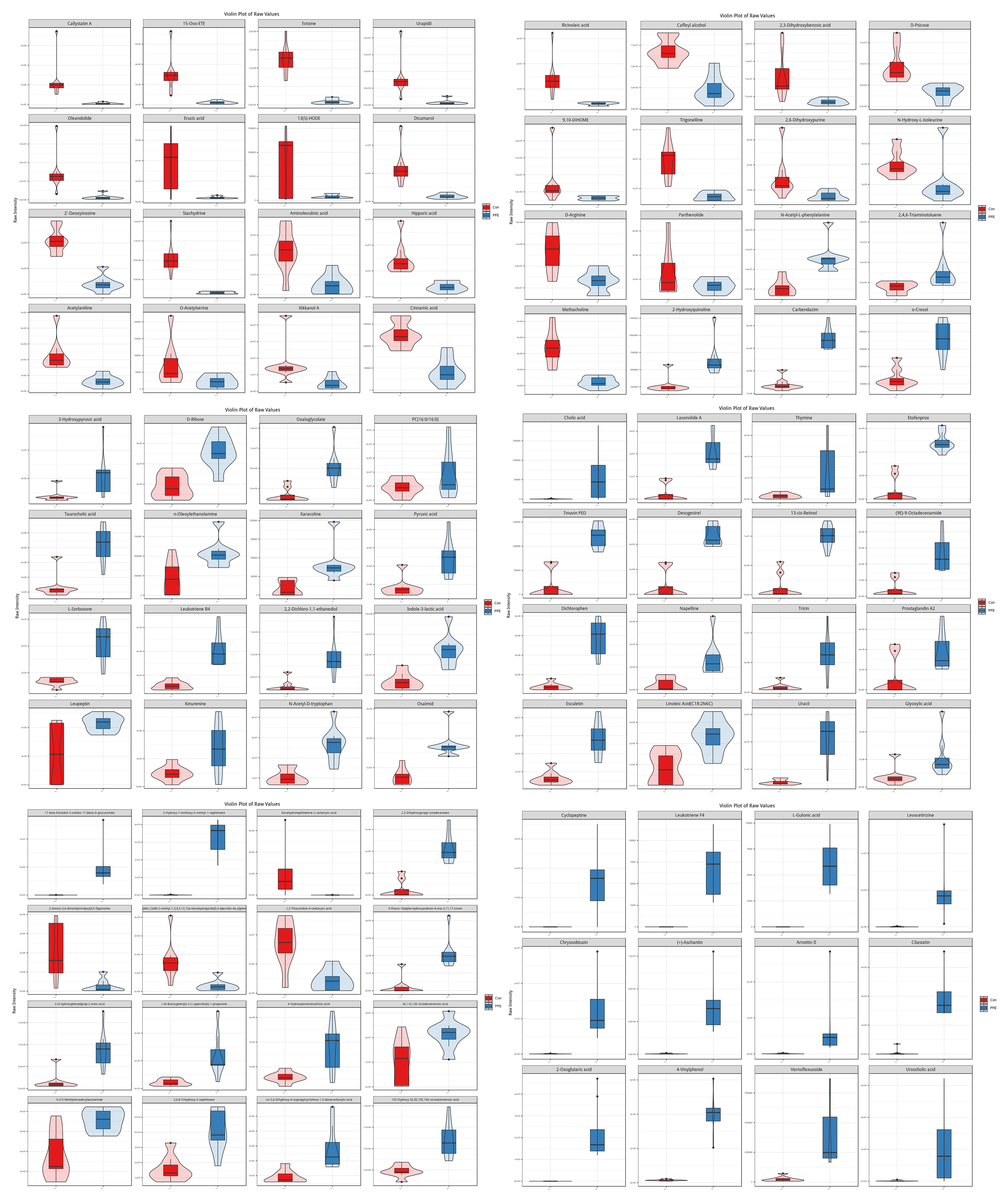

Supplement: Supplementary Materials — Figure S1: expressions of 92 metabolites in PFE group. Table S1: chemical composition identification of PFE. Table S2: table of 2-level metabolites information. Table S3: table of differential proteins information. [file 4202861.f1.zip › Figure S1.jpg]
